# Updated unified phylogenetic classification system and revised nomenclature for Newcastle disease virus

**DOI:** 10.1016/j.meegid.2019.103917

**Published:** 2019-10

**Authors:** Kiril M. Dimitrov, Celia Abolnik, Claudio L. Afonso, Emmanuel Albina, Justin Bahl, Mikael Berg, Francois-Xavier Briand, Ian H. Brown, Kang-Seuk Choi, Ilya Chvala, Diego G. Diel, Peter A. Durr, Helena L. Ferreira, Alice Fusaro, Patricia Gil, Gabriela V. Goujgoulova, Christian Grund, Joseph T. Hicks, Tony M. Joannis, Mia Kim Torchetti, Sergey Kolosov, Bénédicte Lambrecht, Nicola S. Lewis, Haijin Liu, Hualei Liu, Sam McCullough, Patti J. Miller, Isabella Monne, Claude P. Muller, Muhammad Munir, Dilmara Reischak, Mahmoud Sabra, Siba K. Samal, Renata Servan de Almeida, Ismaila Shittu, Chantal J. Snoeck, David L. Suarez, Steven Van Borm, Zhiliang Wang, Frank Y.K. Wong

**Affiliations:** a Exotic and Emerging Avian Viral Disease Research Unit, Southeast Poultry Research Laboratory, US National Poultry Research Center, ARS, USDA, 934 College Station Road, Athens, GA 30605, USA; b Department of Production Studies, Faculty of Veterinary Science, University of Pretoria, Old Soutpan Road, Onderstepoort, Pretoria 0110, South Africa; c CIRAD, UMR ASTRE, F-97170 Petit-Bourg, Guadeloupe, France; d ASTRE CIRAD, INRA, Université de Montpellier, Montpellier, France; e Center for Ecology of Infectious Disease, Department of Infectious Diseases, Department of Epidemiology and Biostatistics, Institute of Bioinformatics, University of Georgia, Athens, GA 30602, USA; f Department of Biomedical Sciences and Veterinary Public Health, Swedish University of Agricultural Sciences, Box 7028, 750 07 Uppsala, Sweden; g ANSES, Avian and Rabbit Virology Immunology and Parasitology Unit, National reference laboratory for avian Influenza and Newcastle disease, BP 53, 22440 Ploufragan, France; h OIE/FAO International Reference Laboratory for Newcastle Disease, Animal and Plant Health Agency (APHA –Weybridge), Addlestone KT15 3NB, UK; i Animal and Plant Quarantine Agency, Ministry of Agriculture, Food and Rural Affairs (MAFRA), 177 Hyeoksin 8-ro, Gimcheon-si, Gyeongsangbuk-do 39660, Republic of Korea; j Federal Governmental Budgetary Institution, Federal Centre for Animal Health, FGI ARRIAH, Vladimir 600901, Russia; k Department of Veterinary and Biomedical Sciences, Animal Disease, Research and Diagnostic Laboratory, South Dakota State University, Brookings, SD, USA; l CSIRO Australian Animal Health Laboratory, Portarlington Road, East Geelong, Victoria 3219, Australia; m University of Sao Paulo, ZMV, FZEA, Pirassununga 13635900, Brazil; n Istituto Zooprofilattico Sperimentale delle Venezie (IZSVe), Viale dell'Università 10, Legnaro 35020, Italy; o CIRAD, UMR ASTRE, F-34398 Montpellier, France; p National Diagnostic and Research Veterinary Medical Institute, 15 Pencho Slaveikov blvd., Sofia 1606, Bulgaria; q Friedrich-Loeffler-Institut, 17493 Greifswald, Insel Riems, Germany; r Regional Laboratory for Animal Influenzas and Transboundary Animal Diseases, National Veterinary Research Institute, Vom, Nigeria; s National Veterinary Services Laboratories, Diagnostics and Biologics, Veterinary Services, Animal and Plant Health Inspection Service, U.S. Department of Agriculture, 1920 Dayton Ave, Ames, IA 50010, USA; t Infectious Diseases in Animals, SCIENSANO, Groeselenberg 99, 1180, Ukkel, Brussels, Belgium; u Royal Veterinary College, University of London, 4 Royal College Street, London NW1 0TU, UK; v College of Veterinary Medicine, Northwest A & F University, Yangling, Shaanxi 712100, PR China; w China Animal Health and Epidemiology Center (CAHEC), 369 Nanjing Road, Qingdao 266032, China; x Department of Population Health, College of Veterinary Medicine, University of Georgia, 953 College Station Road, Athens, GA 30602, USA; y Infectious Diseases Research Unit, Department of Infection and Immunity, Luxembourg Institute of Health, 29, rue Henri Koch, L-4354 Esch-sur-Alzette, Luxembourg; z Division of Biomedical and Life Sciences, Faculty of Health and Medicine, Lancaster University, Lancaster, United Kingdom; aa Ministério da Agricultura, Pecuária e Abastecimento, Laboratório Federal de Defesa Agropecuário, Campinas, SP 13100-105, Brazil; ab Department of Poultry Diseases, Faculty of Veterinary Medicine, South Valley University, Qena 83523, Egypt; ac Virginia-Maryland Regional College of Veterinary Medicine, University of Maryland, College Park, MD, USA

**Keywords:** *Avian paramyxovirus 1* (APMV-1), Newcastle disease virus (NDV), Classification, Nomenclature, Genotype, Phylogenetic analysis

## Abstract

Several *Avian paramyxoviruses 1* (synonymous with Newcastle disease virus or NDV, used hereafter) classification systems have been proposed for strain identification and differentiation. These systems pioneered classification efforts; however, they were based on different approaches and lacked objective criteria for the differentiation of isolates. These differences have created discrepancies among systems, rendering discussions and comparisons across studies difficult. Although a system that used objective classification criteria was proposed by Diel and co-workers in 2012, the ample worldwide circulation and constant evolution of NDV, and utilization of only some of the criteria, led to identical naming and/or incorrect assigning of new sub/genotypes. To address these issues, an international consortium of experts was convened to undertake in-depth analyses of NDV genetic diversity. This consortium generated curated, up-to-date, complete fusion gene class I and class II datasets of all known NDV for public use, performed comprehensive phylogenetic neighbor-Joining, maximum-likelihood, Bayesian and nucleotide distance analyses, and compared these inference methods. An updated NDV classification and nomenclature system that incorporates phylogenetic topology, genetic distances, branch support, and epidemiological independence was developed. This new consensus system maintains two NDV classes and existing genotypes, identifies three new class II genotypes, and reduces the number of sub-genotypes. In order to track the ancestry of viruses, a dichotomous naming system for designating sub-genotypes was introduced. In addition, a pilot dataset and sub-trees rooting guidelines for rapid preliminary genotype identification of new isolates are provided. Guidelines for sequence dataset curation and phylogenetic inference, and a detailed comparison between the updated and previous systems are included. To increase the speed of phylogenetic inference and ensure consistency between laboratories, detailed guidelines for the use of a supercomputer are also provided. The proposed unified classification system will facilitate future studies of NDV evolution and epidemiology, and comparison of results obtained across the world.

## Highlights

• An international consortium phylogenetically studied the diversity of NDV.
• Consensus objective NDV classification and nomenclature system was developed.
• Optimal phylogenetic inference method with guidelines is recommended.
• Curated, up-to-date, complete fusion gene datasets for public use were created.
• Three new NDV genotypes were identified.

## 1 Introduction

The International Committee on Taxonomy of Viruses has recently created three genera, named *Orthoavulavirus*, *Metaavulavirus,* and *Paraavulavirus*, within a new subfamily *Avulavirinae* of the family *Paramyxoviridae* (ICTV, 2019). Viruses of genus *Avian orthoavulavirus 1* (AOAV-1) (formerly designated as *Avian avulavirus 1* (AAvV-1)), commonly known as *Avian paramyxoviruses 1* (APMV-1) or Newcastle disease viruses (NDV, used hereafter for the purposes of this paper), cause infections in a wide range of domestic and wild birds worldwide (Amarasinghe et al., 2018; ICTV, 2019; Miller and Koch, 2013). Newcastle disease (ND), caused by virulent NDV, is highly contagious and can be devastating, particularly in immunologically naïve poultry (Alexander et al., 2012). Virulent strains are defined by the World Organisation for Animal Health (OIE) as viruses that have an intracerebral pathogenicity index of 0.7 or higher (2.0 is maximum) or a fusion cleavage site with multiple basic amino acids and phenylalanine at position 117 (OIE, 2012). Newcastle disease has a global impact and 109 of 200 member countries have reported the disease to the OIE in the last five years (OIE, 2018). Between 2006 and 2009, ND was ranked the 8th most important wildlife disease and the 3rd most significant poultry disease (Anonymous, 2011). The importance of NDV infections to avian health has long been recognized, and the viruses have been the subject of considerable scientific investigations over the past several decades (Alexander et al., 2012; Miller and Koch, 2013). The need for NDV classification has resulted in a variety of methods for isolate identification and differentiation (Alexander et al., 1985; Bankowski and Kinjo, 1965; Pennington, 1978). These early techniques were mainly based on biological properties of the virus, such as pathogenicity, plaque formation, thermostability, analyses of structural polypeptides, and hemagglutination inhibition patterns using monoclonal antibodies (Ballági-Pordany et al., 1996; Russell and Alexander, 1983).

The broad circulation of NDV in poultry populations led to significant genetic diversity of the virus and constant emergence of NDV variants. Given the clinical and economical relevance of NDV to the poultry industry and the broad use of live ND vaccines worldwide, sequencing and phylogenetic analysis became the methods of choice for the characterization of NDV strains circulating in the field. With the incorporation of molecular methods into viral research, the collective knowledge of the NDV genetic makeup has greatly improved. To track NDV evolution and genetic diversity, several NDV molecular classification systems have been developed. In the late 1980s, two related studies based on analyses of the fusion (F) and hemagglutinin-neuraminidase (HN) genes' sequence diversity and HN gene sizes, proposed the existence of three NDV lineages, namely A, B and C (Sakaguchi et al., 1989; Toyoda et al., 1989). In 1996, Ballági-Pordani et al. suggested the classification of NDV isolates into six distinct genotypes (I to VI) based on restriction fragment length polymorphism analyses (Ballági-Pordany et al., 1996). This system was validated and further improved by phylogenetic analysis of partial F gene sequence data, and additional NDV genotypes have since been identified (Herczeg et al., 1999; Lomniczi et al., 1998). A subsequent study based on complete genome sequences identified the existence of three different NDV genome sizes (15,186, 15,192, and 15,198 nucleotides) and separated NDV isolates into two classes – class I and class II (Czeglédi et al., 2006). Each class was further divided into multiple genotypes (Kim et al., 2007a; Maminiaina et al., 2010; Miller et al., 2010b; Tsai et al., 2004). Another classification system, based on partial F gene analysis, was defined by Aldous and colleagues and separated NDV isolates into six distinct lineages (1 to 6) and 13 sub-lineages (Aldous et al., 2003), with an additional lineage 7 proposed later (Cattoli et al., 2010; Snoeck et al., 2009). These systems pioneered the classification efforts for NDV and provided important information on NDV evolution and genetic diversity. However, each system was based on different approaches and lacked objective criteria for the classification of NDV isolates into different genetic groups. These deficiencies created inconsistencies in NDV classification, made discussions and comparisons among studies difficult, and generated confusion in the assignment of existing and new genetic groups.

To overcome these challenges, a unified and objective NDV classification system was proposed in 2012 (Diel et al., 2012a). This system utilized the complete F gene coding sequences and incorporated a number of objective criteria for classification of NDV, including: i) phylogenetic topology; ii) inter-populational evolutionary nucleotide distances; iii) branch support; and iv) epidemiological independence of at least four isolates per sub/genotype. The use of Diel et al. (2012a) system and criteria led to the classification of class I NDV isolates into a single genotype (genotype 1) containing three sub-genotypes, while class II encompassed viruses delineated into 15 genotypes (I to XV) and multiple sub-genotypes. This system was widely adopted, and its use resulted in the identification of three more genotypes (XVI, XVII, and XVIII) (Courtney et al., 2013; Snoeck et al., 2013b). In addition, multiple sub-genotypes have been proposed within many of the existing genotypes (Ahmadi et al., 2016; Byarugaba et al., 2014; Chumbe et al., 2017; Das and Kumar, 2017; Dimitrov et al., 2016b; Esmaelizad et al., 2017; Ganar et al., 2017; He et al., 2018; Miller et al., 2015; Molini et al., 2017; Nath and Kumar, 2017; Ramey et al., 2017; Ramey et al., 2013; Sabouri et al., 2017; Sabra et al., 2017; Servan de Almeida et al., 2013; Snoeck et al., 2013a; Susta et al., 2014; Xue et al., 2017a; Xue et al., 2017b).

While the Diel et al. (2012a) system provided objective criteria for classification of NDV, the ample circulation and constant evolution of NDV led to the almost simultaneous identification and uncoordinated naming of new genotypes. In other instances, new genotypes were created without applying all of the proposed criteria, or the classification was completed using a limited sequence dataset (often using partial F gene sequences) rather than using a curated dataset of all available complete NDV F gene sequences. For these reasons, there are inconsistencies in the naming and classification of some newly proposed genotypes and some sub-genotypes bear identical names while describing different and unrelated viruses (Ahmadi et al., 2016; Barman et al., 2017; Das and Kumar, 2017; Esmaelizad et al., 2017; Ganar et al., 2017; Gowthaman et al., 2018; Nath and Kumar, 2017; Servan de Almeida et al., 2013; Snoeck et al., 2013a; Xue et al., 2017a). Furthermore, with the increased surveillance efforts and the improvement of sequencing technologies, the amount of available sequences has vastly increased, adding to the already complex challenge of studying the molecular evolution of NDV and the relationships among its isolates.

To address these issues, a large group of international scientists and collaborators from 29 laboratories from all continents (except Antarctica) and representing all OIE reference laboratories for ND, was established in early 2014. Members of this consortium have convened during the OFFLU meeting in April 2014 in Paris, France, the 9th International Symposium on Avian Influenza in April 2015 in Athens, Georgia, USA, the 2nd International Avian Respiratory Disease Conference in May 2018 in Athens, Georgia, USA, and through multiple teleconferences. Three working groups were assigned, and the consortium was set out to revise the existing NDV classification systems and to propose updated classification and nomenclature criteria for NDV. The work described here was performed with the aims to: i) establish unified criteria for NDV sequence collection and curation; ii) generate and maintain updated sequence datasets; iii) perform comprehensive phylogenetic analyses using the generated datasets; iv) propose unified criteria for NDV classification; v) propose naming criteria for NDV genotypes; and vi) provide curated complete and partial datasets for future public use. Here, in-depth analyses of the genetic diversity of NDV utilizing all available complete F gene sequences are presented. An updated NDV classification system with new nomenclature criteria is proposed. In addition, curated reference datasets for phylogenetic inference, software recommendations, and guidelines for using high-performance computer clusters are provided.

## 2 Materials and methods

### 2.1 Working groups

Three working groups (WG1 to 3) were formed within the consortium. WG1 was tasked with defining criteria for sequence data quality (e.g. size, coverage, recombination, and identical sequences) and generating the datasets that will be used in the analyses. The focus of WG2 was to make the datasets publically available for future application. The WG3 was tasked to evaluate methods for phylogenetic analyses utilizing the generated datasets and to propose unified NDV classification and nomenclature criteria. This WG was also tasked with selecting representative sequences for a smaller class II dataset for rapid genotype identification of new isolates (named the “pilot” dataset).

### 2.2 Collection of sequences

Due to its variability and biological function (virulence determinant), the F gene is commonly targeted in sequencing efforts. Accordingly, the complete F gene coding nucleotide sequences of all available class I and class II NDV isolates were downloaded from GenBank of the National Center for Biotechnology Information (Benson et al., 2017). Only sequences that represented >99% of the full-length open reading frame of the F gene (≥1645 nucleotides, nt) were selected and aligned using Multiple Alignment with Fast Fourier Transformation (MAFFT v7.221.3) (Katoh and Standley, 2013) as implemented in the Galaxy platform (Goecks et al., 2010). A total of 2966 sequences that had submission dates up to February 21st 2019 were obtained, resulting in two separate initial datasets – class I (n = 431) and class II (n = 2535). Similarly, all available complete genome sequences of class II NDV were downloaded from GenBank and aligned using the same tools (due to the scarcity of class I complete genomes, these were not analyzed). The leader and tail sequences and intergenic regions were trimmed, and the coding sequences for all six genes were concatenated. A total of 651 class II complete genome sequences submitted on or before Feb 21st 2019, were obtained. Metadata like host, year and location of isolation, and isolate name for all sequences were collected from GenBank annotations, where available, or from the respective peer-reviewed publications when the data was not provided with the sequence submission.

### 2.3 Datasets curation

Sequences identified as 100% identical and originating from the same species, country, laboratory, and/or outbreak or having identical names and dates were removed from the datasets (one representative from each duplication pair/group was kept). Each sequence was evaluated and all gaps and insertions that caused alignment shifts were deleted. Sequences that were found to originate from man-made clones, mutant viruses, vaccines, and chimeric viruses were removed from the datasets. Sequences obtained from viruses identified as spillovers or escapes (recent viruses identical or almost identical to vaccine and standard strains originally isolated decades before) were also excluded from further analyses. In addition, all sequences were subjected to recombination analysis using the RDP4 program (Martin et al., 2015) as described previously (Dimitrov et al., 2016a), and sequences with recombination events were removed from the datasets.

### 2.4 RNA extraction and nucleotide sequencing

Three Nigerian and five Bulgarian historical viruses from underrepresented genotypes were obtained from the repository of the Southeast Poultry Research Laboratory of the USDA and were sequenced in this study: (chicken/Nigeria/Kano/N52/899/1973, chicken/Nigeria/Plateau/N53/900/1973, chicken/Nigeria/FLD/N54/901/1973, pullet/Bulgaria/Plovdiv/1153/1959, chicken/Bulgaria/ElovDol/1156/1981, pigeon/Bulgaria/Septemvriitsi/1157/1982, chicken/Bulgaria/Furen/1159/1988, pigeon/Bulgaria/NovoSelo/1161/1995). RNA extraction, next-generation sequencing, and genome assembly were performed as described previously (Dimitrov et al., 2017).

### 2.5 Final datasets and phylogenetic analyses

A total of 1956 complete F gene sequences remained after the curation of the datasets and were used for the phylogenetic analyses – class I (n = 284, Supplemental Table S1) and class II (n = 1672, Supplemental Table S2, “larger” class II dataset). Neighbor-joining (NJ), maximum-likelihood (ML), and Bayesian methods were used to infer phylogenetic relationships and construct phylogenetic trees of both classes. Neighbor-joining trees (Maximum Composite Likelihood model) were constructed using MEGA6 (Tamura et al., 2013) with 1000 bootstrap replicates. Codon positions included were 1st, 2nd, 3rd, and noncoding, and all positions containing gaps and missing data were eliminated. Maximum-likelihood trees based on general time-reversible (GTR) model (Tavaré, 1986) (goodness-of-fit measured by the corrected Akaike information criterion) were constructed by using RaxML version 8.2.11 (Stamatakis, 2014) with 1000 bootstrap replicates and the following command: -s input_filename.phy -n output_tree_name -m GTRGAMMAI -f a -x 123 -N 1000 -p 456. A discrete Gamma distribution (Γ) was used to model evolutionary rate differences among sites and the rate variation model allowed for some sites to be evolutionarily invariable (I). Utilizing the same datasets and parameters, RaxML trees were also constructed through the CIPRES Science Gateway (Miller et al., 2010a). A class II complete genome tree was constructed using the same ML method, utilizing the curated dataset of concatenated coding sequences (n = 491). Class I and class II complete F gene maximum-likelihood trees based on the GTR model were also constructed using MEGA6. Bayesian analyses were performed for comparison using the BEAGLE-enhanced parallel version of MrBayes v3.2 (Altekar et al., 2004; Ayres et al., 2012; Ronquist et al., 2012). For both class I and II datasets, the GTR substitution model with a gamma-distributed variation of rates and a proportion of invariable sites (Yang, 1993) were used. All other parameters were set to the MrBayes default settings. Independent runs of a 10 million generation length Markov Chain Monte Carlo (MCMC) simulation (Geyer, 1991) were performed sampling every 1000 states. Run convergence was assessed using Tracer v1.6 (http://tree.bio.ed.ac.uk/software/tracer) to ensure an effective sampling size larger than 200 for all parameters. After discarding non-convergent runs and removal of at least 10% burn-in, the runs were combined with MrBayes to produce a maximum clade credibility tree and Bayesian posterior probabilities (BPP) were estimated. Trees were visualized using FigTree v1.4.2 (http://tree.bio.ed.ac.uk/software/figtree). Topological congruence between trees was compared through visual inspection for each sub/genotype.

The final class I and class II datasets used for phylogenetic reconstruction were also used to estimate the mean inter-populational distances (nucleotide distances between sub/genotypes). The estimates of average evolutionary distances were inferred using MEGA6 (Tamura et al., 2004). Analyses were conducted using the Maximum Composite Likelihood model. The rate variation among sites was modeled with a gamma distribution (shape parameter = 1).

### 2.6 Pilot tree and individual genotype trees for rapid preliminary identification

After performing the analyses described above and identifying the main NDV genotypes, a smaller (pilot) dataset of class II sequences (n = 125, Supplemental Table S3, “smaller” class II dataset) was parsed from the complete dataset used in the phylogenetic analyses. This pilot dataset included representative sequences from all identified sub/genotypes and was phylogenetically analyzed with the ML method as described above to ensure that the topology inferred using the larger class II dataset would be maintained if fewer isolates were used. To explore the option to use sub-trees for genotype identification of new isolates, sub-trees were built separately, utilizing the ML method and using all sequences in each class II genotype. To ensure congruent topology with the biggest class II tree, these sub-trees were rooted to historical isolates ancestral to the respective genotypes.

### 2.7 Classification criteria

In all analyses, the criteria put forth by Diel et al. (2012a) were used to initially assess the diversity of NDV and served as the foundation to update and propose a consensus classification system. The criteria accepted here, include the previously proposed criteria of clustering of sequences from one sub/genotype into a monophyletic branch, average nucleotide distance among genotypes (above 10%), and the presence in each group of at least four independent viruses from distinct outbreaks and without a direct epidemiologic link. To avoid excessive delineation of sub-genotypes, the cut-off value for nucleotide distance between these was increased to 5% (instead of 3%). In order to increase the reliability and stringency of the phylogenetic clustering, the bootstrap support value cut-off at the nodes that define sub/genotypes was raised to 70% or above. The updated consensus classification criteria are summarized in Table 1. Similar classification criteria have also been used by the World Health Organization/World Organisation for Animal Health/Food and Agriculture Organization H5N1 Evolution Working Group (WHO/OIE/FAO-H5N1-EWG, 2014).

**Table 1:** Criteria for classification of NDV isolates.

Criterion	Description
1	Assignment of viruses into new genotypes and sub-genotypes is done based on complete fusion gene phylogenetic analysis (sequences of at least 1645 nucleotides or longer).
2	Assignment of viruses into new genotypes and sub-genotypes is done only utilizing a complete dataset of sequences from all existing genotypes. All classification criteria listed below need to be fulfilled for naming new genotypes and sub-genotypes.
3	Sub-trees and pilot tree can be used for assigning new isolates to existing sub/genotypes.
4^a^	New genotypes or sub-genotypes are created only when four or more independent isolates, without a direct epidemiologic link (i.e. distinct outbreaks), are available.
5	New genotypes and sub-genotypes are created based on the phylogenetic tree topology (need to cluster into monophyletic branches) using the Maximum Likelihood method and the general time-reversible (GTR) model with gamma distribution (Γ) utilizing RaxML or a comparable tool.
6^a^	The mean nucleotide distance (evolutionary distances) between groups is inferred as the number of base substitutions per site from averaging over all sequence pairs between groups using MEGA v. 5/6/7 software (or a comparable tool) and utilizing the Maximum Composite Likelihood model with rate variation among sites that was modeled with a gamma distribution (shape parameter = 1).
7^a^	Different genotypes have an average distance per site above 10% (0.1).
8	Different sub-genotypes have an average distance per site above 5% (0.05).
9	The bootstrap value at the genotype and sub-genotype defining node is 70% or above (≥70%).
10	Viruses that do not fulfill all classification criteria are assigned to the lower order (closer to the root) sub/genotype (see Table 2 for nomenclature criteria).

a Criteria that were adopted from the Diel et al. (2012a) classification system.

### 2.8 Nomenclature criteria

When possible, names of genotypes were maintained with Arabic numerals used in class I and Roman numerals used in class II. The lowercase Latin letter system used to name sub-genotypes was replaced with a numerical-decimal system using Arabic numerals. Dichotomous splitting was used at every defining node (at which separation into sub-genotypes was done) using the numerals 1 and 2. It is assumed that each node has two immediate descendants (two higher order sub-genotypes). For example, within genotype VII (names of sub/genotypes used in this paragraph do not correspond to the classification proposed in this study and are used solely for demonstration purpose), the first two sub-genotypes will become VII.1 and VII.2 (see Supplemental Fig. S1 for examples). At the next node (closer to the tips or higher order) within sub-genotype VII.1, the sub-genotypes that are one order higher will become VII.1.1 and VII.1.2, and respectively at the next node within sub-genotype VII.2, the two further sub-genotypes will be named VII.2.1 and VII.2.2. The updated nomenclature criteria are presented in Table 2 and examples are provided in Supplemental Fig. S1, Supplemental Fig. S2, and Supplemental Fig. S3.

### 2.9 Accession numbers

The complete F-gene sequences (n = 8) of NDV obtained in this study were submitted to GenBank and are available under the accession numbers MH996897 to MH996904.

**Table 2:** Nomenclature criteria for existing and new NDV genotypes and sub-genotypes^a^.

Criterion	Description
1	All existing genotypes (as per Diel et al., 2012a) in the current classes I and II maintain their Arabic and Roman numerals, respectively.
2	The lowercase Latin letter system to name sub-genotypes is replaced by the numerical-decimal system using Arabic numerals.
3	Dichotomous splitting is used at every defining node (at which separation into sub-genotypes is done) using the numerals 1 and 2.
4	Class I sub/genotypes receive a numerical-decimal address (Arabic numerals separated by periods) that starts with the Arabic numeral of the genotype. Further numeration is made using the dichotomous system at every defining node using numerals 1 and 2. (e.g. 1.1 and 1.2).
5	Class II sub/genotypes receive a numerical-decimal address (Roman-Arabic numerals separated by periods) that starts with the Roman numeral of the genotype. Further numeration is made using the dichotomous system at every defining node (separating sub-genotypes) using numerals 1 and 2. (e.g. VII.1 and VII.2) (example in Supplemental Fig. S1).
6	At the higher order (next defining node closer to the tips) within sub-genotype VII.1, for example, the sub-genotypes that are one order higher will become VII.1.1 and VII.1.2. At the next node within sub-genotype VII.2, the two further sub-genotypes will be named VII.2.1 and VII.2.2, respectively (example in Supplemental Fig. S1).
7	If a branch has unresolved topology, low branch support (i.e. there are polytomies or low bootstrap values), or insufficient number of isolates, the viruses within this branch are not assigned to a higher order, and are assigned the name of the lower order until the topology/support/number of the isolates is resolved and all criteria are fulfilled, regardless of the fact that the distnace criterion is met.
8	Newly identified virus diversity (a group of viruses undescribed before) that meets all classification criteria will be classified as new genotype and will receive a subsequent Roman numeral (e.g. currently XXII is the next available) (example in Supplemental Fig. S2, red color).
9	Existing sub-genotypes that fulfill the genotype criteria will not be designated with a new name in order to maintain the ancestral information in their names (examples in Supplemental Fig. S2, green color).
10	If a new sub-genotype of viruses is identified later, but still falls into a higher order within an existing genotype, this new sub-genotype receives the next consecutive numerical address for the respective level of order in the phylogeny to avoid re-numbering all existing sub-genotypes that are of higher order. For example if a new sub-genotype that outgroups VII.1.1 and VII.1.2 is identified but it is still within VII.1, this new sub-genotype will be named VII.1.3 (example in Supplemental Fig. S3).

a The names of genotypes and sub-genotypes used in this table do not correspond to the names in the phylogenetic trees presented in the current study. The names in this table were used for demonstration purposes only.

## 3 Results and discussion

### 3.1 Dataset curation

A total of 1956 complete F gene sequences remained after the curation of the datasets, including 284 class I sequences and 1672 class II sequences. After utilizing the dataset curation criteria described above, 1010 sequences were removed from the initial datasets (class I: n = 147; class II: n = 863). In the class I dataset, 128 duplicate sequences, 17 recombinant sequences and 2 sequences that resulted from cloning were identified and removed. In the class II dataset, 419 duplicates, 170 recombinant forms, 56 man-made vaccine clones and 218 spillovers or escapes of vaccine/standard viruses were identified and removed. These numbers highlight the importance of carefully assessing the NDV datasets prior to phylogenetic inference. It has been reported, for example, that spillovers of vaccine and of older standard NDV strains can significantly impact analyses, resulting in skewed inferences (Ayala et al., 2016; Dimitrov et al., 2016a; Taylor et al., 2017). In addition, the increasing number of chimeric sequences as identified by the recombination analysis has implications in virus classification and impacts topology of phylogenetic trees. Although recombination has been suggested to play a role in NDV evolution (Han et al., 2008; Qin et al., 2008; Satharasinghe et al., 2016) and increasing numbers of chimeric sequences have been deposited in GenBank, the natural occurrence of recombination events remains disputable (Afonso, 2008; Song et al., 2011). Selection and the inherent error rate of the viral RNA polymerase are believed to be the main forces driving the evolution and diversity of NDV (Diel et al., 2012a). The actual role and contribution of recombination to this process remains to be established. However, to provide a reliable phylogenetic inference based on clearly identified genotypes, we suggest that recombinant sequences always be identified through analysis with RDP (or alternative appropriate tool) and removed from the dataset before performing an analysis and a classification tree construction. A similar approach has already been adopted for porcine circovirus type 2 (Franzo et al., 2015) and infectious bronchitis virus (Valastro et al., 2016).

### 3.2 Phylogenetic analyses and congruence between inference methods

#### 3.2.1 Class I

**Fig. 1:**
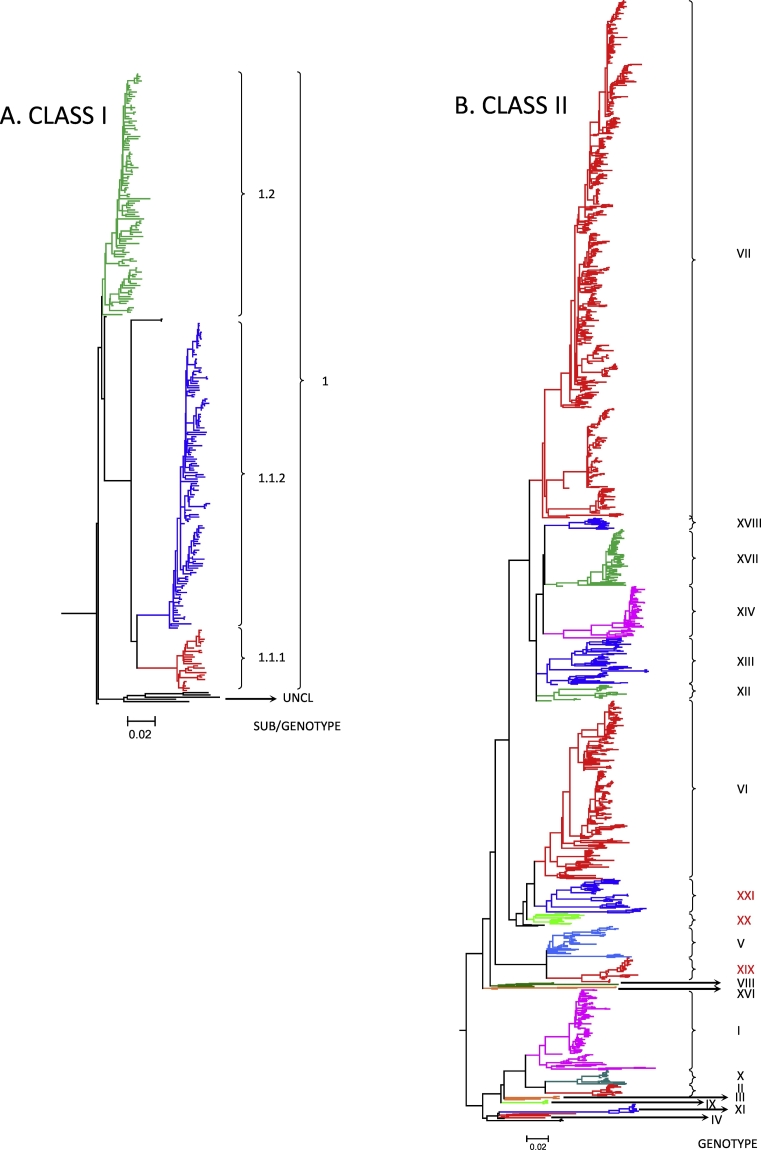
Maximum likelihood phylogenetic trees of class I (A) and class II (B). Phylogenetic analyses are based on the full-length nucleotide sequence of the fusion gene of isolates representing Newcastle disease virus class I (A, n = 284) and class II (B, n = 1672). The evolutionary history was inferred by using RaxML (Stamatakis, 2014) and utilizing the Maximum Likelihood method based on the General Time Reversible model with 1000 bootstrap replicates. The trees with the highest log likelihood (class *I* = −18,683.27, class II = −106,684.34) are shown. A discrete gamma distribution was used to model evolutionary rate differences among sites and the rate variation model allowed for some sites to be evolutionarily invariable. For imaging purposes, the taxa tips are not displayed and colors are randomly assigned to indicate the different sub/genotypes. Three new class II genotypes assigned in the current study are highlighted in red font. The trees are drawn to scale, with branch lengths measured in the number of substitutions per site. (For interpretation of the references to color in this figure legend, the reader is referred to the web version of this article.)

The phylogenetic analyses utilizing the NJ, ML, and Bayesian methods revealed high topology congruence within class I NDV (Supplemental Fig. S4, A-C). All class I trees were rooted to the oldest class I NDV isolate - EF564833/Canada Goose/USA(OH)/78/1987. The existence of a single genotype (i.e. genotype 1) was confirmed as previously determined by Diel et al. (2012a). Minor topological differences among a few unclassified sequences and lack of branch support (<70 bootstrap values) were observed when using the NJ method; however, the existence of a single class I genotype 1 was consistent between the used methods. Based on the ML method (selected due to easier and faster tree construction and because it outperformed NJ), there are three (n = 3) sub-genotypes within genotype 1 of class I (Supplemental Fig. S4B). Utilizing the new naming criteria, these are sub-genotypes 1.1.1 (former 1a), 1.1.2 (former 1b), and 1.2 (former 1c and 1d) (Supplemental Table S1, Table 3). For visualization purposes and to provide an overview of the topology of class I NDV, a condensed ML tree without taxa names is featured in Fig. 1A. The increase of the cut-off for differentiation of sub-genotypes to 5% resulted in the merging of former sub-genotypes 1c and 1d into one single sub-genotype 1.2 (distance between 1c and 1d - 3.68%) (Supplemental Table S4). Of note, there are five unclassified viruses within class I (Supplemental Fig. S4B, UNCL 9 to 13) that cluster together but are genetically distant from each other (9 to 11%) and have the potential to become members of multiple separate genotypes if sequences from additional epidemiologically independent viruses related to them become available. It is also possible that these sequences represent extinct viral lineages, which does not warrant their further classification into genotypes. These observations highlight the importance of having at least four epidemiologically independent sequences in order to classify NDV isolates into a new sub/genotype. Although there is evidence of ongoing evolution among the viruses of class I NDV (Ramey et al., 2017), the evolutionary diversity within this class remains low. The majority of sequenced class I viruses are isolated from wild birds, however, poultry detections are not uncommon (Dimitrov et al., 2016b; Kim et al., 2007a). The circulation of class I viruses mainly in wild birds, which are not vaccinated for ND, thus keeping the immune pressure on these viruses low, may explain the relatively lower genetic diversity of these viruses when compared to class II. While there has been one report of a virulent class I NDV (Collins et al., 1998), all remaining sequences in the class are from viruses of low virulence. It is likely that the low virulence nature of class I viruses in wild birds and poultry and their low incidence in poultry, has negatively influenced efforts to sequence and characterize them. This may explain the restricted spatial distribution of the majority of the available sequences (the U.S. and China, and a few sequences from Japan and Europe). In addition, not all RT-PCR assays are able to detect class I isolates and their circulation may have been missed as they do not commonly cause clinical disease (Fuller et al., 2010; Kim et al., 2007b). Additional sequencing of historical and prospective class I NDV isolates will provide a more detailed characterization of the genetic diversity of this group of viruses.

#### 3.2.2 Class II

The class II phylogenetic trees, constructed using the NJ, ML, and Bayesian methods, presented some significant differences, particularly between NJ (Supplemental Fig. S5A) and the other two methods (ML and Bayesian) (Supplemental Fig. S5B and S5C, respectively). Notably, lack of branch support was observed at many defining nodes of the NJ tree, and the identified monophyletic branches differed compared to the remaining two methods (e.g. in genotypes I, VI, and XIII) (Supplemental Fig. S5, A–C). With the exception of one small branch (former sub-genotype Vd) and low support between two genotype I sub-genotypes, the trees obtained using the ML and the Bayesian approach were generally consistent with well-defined monophyletic branches and strong branch support (≥70 bootstrap and ≥ 0.9 posterior probability values, respectively) at most defining nodes (Supplemental Fig. S5B and S5C, Supplemental Table S2). Since it statistically outperformed NJ, and due to its ease of use, greater speed and wide utilization and accessibility, the ML method was selected as a method of choice for class II NDV phylogenetic inference. Compared to class I, class II is more diverse, contains a range of non-virulent to virulent viruses, and the complete analyses identified at least 20 distinct genotypes (I to XXI, genotype XV that contains only recombinant sequences was excluded from the final analyses). Viruses previously classified as members of sub-genotype Va were separated into a new genotype XIX. Viruses previously classified as five of the sub-genotypes of genotype VI also formed two new genotypes, namely XX and XXI. All class II trees were rooted to the clade of genotype IV, consistent with molecular clock trees. The evolutionary distances between genotypes are presented in Table 4.

Consistent with previous classifications, genotypes I, V, VI, VII, XII, XIII, XIV, and XVIII were confirmed to be further divided into sub-genotypes (Supplemental Fig. S5B, Table 3) (Diel et al., 2012a; Dimitrov et al., 2016b; Snoeck et al., 2013b). The number of existing sub-genotypes in genotypes I, XIV, and XVIII, remained the same (four, two, and two, respectively) (Ramey et al., 2013; Snoeck et al., 2013b), and their new names were assigned based on the updated nomenclature criteria (see Table 5, Supplemental Table S2, Supplemental Fig. S5B). No sub-genotypes were identified in genotypes II, III, IV, VIII, IX, XI, XVI, XIX, and XX. Sequences from eight viruses were not assigned to any of the identified genotypes (“UNCL” 1 to 8, see Supplemental Table S2). For an overview purposes and to provide an overall picture of the topology of class II NDV, a condensed phylogenetic tree without taxa names is presented in Fig. 1B. Utilizing the higher nucleotide distance cut-off of 5%, however, resulted in no sub-genotypes in genotypes X and XVII (distances of 4.83 and 4.59, respectively, Supplemental Table S4).

The use of the updated classification and nomenclature criteria led to the identification of two sub-genotypes in genotype V, designated V.1 (former Vb) and V.2 (former Vc). Previously identified sub-genotype Vd (Byarugaba et al., 2014) lacked branch support and sufficient number of independent isolates and, following the newly proposed classification and naming criteria, was assigned to the lower order (i.e. genotype V). Eight more isolates from Europe and South and North America from the 1970s and 1980s could not be assigned to any of the genotypes (lack of branch support and branches not monophyletic) and were also designated as members of the lower order genotype V (Supplemental Fig. S5B).

Undoubtedly, genotype VI is the most diverse among all NDV genotypes. While the lineage system separated this group into four sub-lineages with multiple further groups (Aldous et al., 2004; Aldous et al., 2003), 14 sub-genotypes were identified based on Diel's system (Diel et al., 2012a). Although viruses classified into two new genotypes (XX and XXI) were taken out of genotype VI, after utilizing the updated criteria suggested here, this genotype was still found to contain seven sub-genotypes (Supplemental Fig. S5B). Former sub-genotypes VIa and VIn (He et al., 2018) were merged into sub-genotype VI.2.1.1.1, as they did not pass the 5% criterion for nucleotide distance between sub-genotypes (3.46%, Supplemental Table S4). Previously identified sub-genotypes VIj and VIk (Xue et al., 2017b) were just above the 5% cut-off (Table 3) and were named VI.2.1.1.2.1 and VI.2.1.1.2.2, respectively. Former sub-genotypes VIb, VIe, VIf, VIh were confirmed and renamed accordingly (Table 5, Supplemental Table S2). Considering that many genotype VI NDV have been part of the panzootic in Columbiform birds that emerged in the Middle East more than four decades ago (Aldous et al., 2004; Alexander, 1988), and is still ongoing (Sabra et al., 2017), it is not surprising that this is the most diverse group of NDV. Unlike any other NDV genotype, genotype VI viruses have been isolated in all continents, except Antarctica (Dimitrov et al., 2016b). Their potential to infect chickens has been demonstrated, but only rarely, and they appear to be highly adapted to some Columbiform birds (Aldous et al., 2014; Ujvari et al., 2003). Viruses that belong to genotype VI are often referred to as pigeon paramyxoviruses 1 (PPMV-1). These viruses can be distinguished as unique antigenic variants of NDV by the patterns produced in a hemagglutination inhibition (HI) assay when tested with a panel of monoclonal antibodies (Collins et al., 1989). This panel, although described in the OIE manual (OIE, 2012), is not widely available and used by NDV reference laboratories. Not all genotype VI viruses have been confirmed to be PPMV-1.

The use of the updated classification criteria significantly impacted genotype VII landscape. As many former sub-genotypes did not fulfill the distance (VIIb, VIId, VIIe, VIIj, VIIl), branch support (VIIh, VIIi, VIIk), and/or number of independent isolates (VIIk) criteria, these were merged into single sub-genotypes, resulting in a total of three genotype VII sub-genotypes (Table 3, Table 5, Supplemental Fig. S5B, Supplemental Table S4). The viruses responsible for the fourth NDV panzootic grouped together, and based on nucleotide distance, were classified into a single genotype (VII.1.1), combining former sub-genotypes VIIb, VIId, VIIe, VIIj, and VIIl. An exception is former sub-genotype VIIf that was classified as a separate sub-genotype, namely VII.1.2. The groups of viruses involved in the fifth NDV panzootic (VIIh and VIIi), that affected Indonesia, Asia, the Middle East, Europe, and Africa (Abolnik et al., 2018; Fuller et al., 2017; Kammon et al., 2018; Mapaco et al., 2016; Miller et al., 2015), lacked branch support, and were merged into a single sub-genotype VII.2. A group of five other sequences from viruses isolated in Namibia in 2016, previously identified as sub-genotype VIIk (Molini et al., 2017), were also assigned to VII.2 due to lack of branch support and insufficient number of independent isolates (Supplemental Fig. S5B). Interestingly, all these sub-genotype VII.2 groups met the nucleotide distance criterion with high genetic distances of 9.40% and 11.17% (Supplemental Table S4); however, all classification criteria need to be fulfilled before a clade is named as a separate sub-genotype. Of note, this branch (VII.2) including these three groups of viruses (former VIIh, VIIi, and VIIk) is almost 10% (9.83%, Table 3) distant from the remaining genotype VII viruses, and with the continuing evolution of NDV, will likely fulfill the criteria for a standalone genotype in the near future.

Genotype XIII was found to consist of four sub-genotypes (Supplemental Fig. S5B). Two previously assigned sub-genotypes (Miller et al., 2015), were further split into two sub-genotypes each – XIIIa into XIII.1.1 and XIII.1.2, and XIIIb into XIII.2.1 and XIII.2.2, respectively (Supplemental Fig. S5B) because of the addition of new sequences from recent studies (Gowthaman et al., 2019; Mayahi and Esmaelizad, 2017). Viruses isolated from different countries in Africa between 1995 and 2015 formed sub-genotype XIII.1.1, while viruses from Sweden, Russia, India, and Iran from 1997 to 2011 were classified in XIII.1.2. Viruses isolated in the last decade from Pakistan and India formed XIII.2.1 and XIII.2.2, respectively. Although very distant from the XIII.2.1 and XIII.2.2 sequences (12.63%), a branch of nine sequences from viruses from India lacked node support and number of independent isolates to be identified as separate sub-genotype and were assigned to the lower order branch (i.e. XIII.2). Similar to the viruses from genotype VII/lineage 5 (to which they were previously assigned), genotype XIII viruses are widely distributed on at least three continents (Cattoli et al., 2010; Munir et al., 2012; Nath et al., 2016; Shabbir et al., 2013). High genetic distances were observed between clades of genotype XIII (Table 3), and the continuous NDV evolution and addition of new genetic data will likely change the classification of this genotype.

Genotype XIX is one of the three newly identified genotypes. This genotype contains all viruses previously classified as sub-genotype Va. Genotype XIX viruses are almost exclusively associated with outbreaks in double-crested cormorants in North America and Canada and additional isolates have been recovered from samples taken from pelicans and gulls in close proximity to cormorants during outbreaks (Diel et al., 2012b; Dimitrov et al., 2016b; Heckert et al., 1996; Rue et al., 2010).

**Table 3:** Estimates of evolutionary distances between class I and class II NDV sub-genotypes^a^, ^b^, ^c^

A. Sub-genotypes within genotype 1 of class I		B. Sub-genotypes within genotype I of class II		C. Sub-genotypes within genotype V of class II		D. Sub-genotypes within genotype VII of class II	
	**1.1 (1a** **+** **1b)**		**I.1 (a** **+** **c** **+** **d)**		**V.1 (b)**		**VII.1 (b** **+** **d** **+** **e** **+** **j** **+** **f** **+** **l)**
**1.2 (1c** **+** **1d)**^d^	7.27	**I.2 (b)**	8.58	**V.2 (c)**	5.86	**VII.2 (h** **+** **i** **+** **k)**	9.91
	**1.1.1 (1a)**		**I.1.1 (a)**				**VII.1.1 (b** **+** **d** **+** **e** **+** **j** **+** **l)**
**1.1.2 (1b)**	7.99	**I.1.2 (c** **+** **d)**	8.52			**VII.1.2 (VIIf)**	5.58
			**I.1.2.1 (c)**				
		**I.1.2.2 (d)**	10.96				

E. Sub-genotypes within genotype VI of class II							
	**VI.1 (b)**		**VI.2.1 (a** **+** **h** **+** **j** **+** **k** **+** **n)**		**VI.2.2.1 (e)**		**VI.2.1.1 (a** **+** **j** **+** **k** **+** **n)**
**VI.2 (a** **+** **e** **+** **f** **+** **h** **+** **j** **+** **k** **+** **n)**	8.05	**VI.2.2 (f** **+** **e)**	8.55	**VI.2.2.2 (f)**	6.14	**VI.2.1.2 (h)**	8.12
							
	**VI.2.1.1.1 (a** **+** **n)**		**VI.2.1.1.2.1 (j)**				
**VI.2.1.1.2 (j** **+** **k)**	6.59	**VI.2.1.1.2.2 (k)**	5.15				


F. Sub-genotypes within genotype XII of class II		G. Sub-genotypes within genotype XIII of class II		H. Sub-genotypes within genotype XIV of class II		I. Sub-genotypes within genotype XVIII of class II	
	**XII.1 (a)**		**XIII.1 (a)**		**XIV.1 (a)**		**XVIII.1 (a)**
**XII.2 (b)**	8.86	**XIII.2 (b)**	9.32	**XIV.2 (b)**	7.55	**XVIII.2 (b)**	6.46
			**XIII.1.1 (a)**				
		**XIII.1.2 (a)**	5.41				
			**XIII.2.1 (b)**				
		**XIII.2.2 (b)**	6.64				

J. Sub-genotypes within genotype XXI of class II							
	**XXI.2.1 (VIg** **+** **VIm)**		**XXI.2.1.1 (VIg)**				
**XXI.2.2 (VIi)**	11.03	**XXI.2.1.2 (VIm)**	9.19				

a Inferred from the complete nucleotide F gene sequences.

b The nucleotide distances were calculated at every defining node.

c The number of base substitutions per site from averaging all sequence pairs between class I and class II sub-genotypes is shown. Analysis was conducted using the Maximum Composite Likelihood model (Tamura et al., 2004). The rate variation among sites was modeled with a gamma distribution (shape parameter = 1). The number of nucleotide sequences in each sub-genotypes is shown in parenthesis. Codon positions included were 1st, 2nd, 3rd and noncoding. All positions containing gaps and missing data were eliminated. Evolutionary analyses were conducted in MEGA6 (Tamura et al., 2013).

d For easier comparison, the former sub-genotype names are provided in parentheses.

**Table 4:** Estimates of evolutionary distances between genotypes of class II NDV.^a^, ^b^

Genotype (number of analyzed sequences)	No. of base substitutions per site^c^																		
	I	II	III	IV	V	VI	VII	VIII	IX	X	XI	XII	XIII	XIV	XVI	XVII	XVIII	XIX	XX
I (n = 120)																			
II (n = 17)	13.06																		
III (n = 6)	11.23	13.76																	
IV (n = 8)	12.79	14.86	10.11																
V (n = 47)	18.06	19.69	16.25	15.03															
VI (n = 265)	19.69	20.64	18.06	16.07	16.06														
VII (n = 772)	18.92	21.63	17.34	15.85	16.12	14.62													
VIII (n = 6)	15.53	16.92	13.78	12.41	12.89	13.35	14.15												
IX (n = 6)	11.44	12.85	***8.62***	10.14	16.05	17.86	17.38	13.50											
X (n = 22)	12.16	11.83	13.52	14.59	19.85	20.85	20.58	17.16	12.88										
XI (n = 14)	20.12	22.60	18.52	15.08	21.76	23.72	24.27	20.56	17.33	22.43									
XII (n = 23)	19.48	22.38	18.14	16.51	15.72	13.81	12.70	14.05	18.23	20.96	24.58								
XIII (n = 70)	18.79	21.28	17.81	16.15	15.87	15.21	12.91	13.98	17.35	20.49	23.45	11.92							
XIV (n = 77)	22.32	25.71	22.18	19.46	18.48	18.29	15.90	16.90	22.44	23.70	28.31	14.47	14.83						
XVI (n = 4)	17.71	20.31	16.63	14.68	15.92	17.22	17.67	13.90	16.56	19.16	23.68	17.38	17.22	20.47					
XVII (n = 85)	17.71	21.30	17.61	16.19	15.90	15.66	13.76	14.67	17.35	21.01	22.75	12.20	12.15	13.59	18.19				
XVIII (n = 17)	18.75	21.55	17.95	16.34	15.92	14.53	13.26	14.46	17.37	20.66	23.27	12.03	11.99	13.95	17.74	10.48			
XIX (n = 38)	20.60	21.49	18.80	17.49	10.12	17.71	17.26	15.00	18.41	21.93	24.47	18.12	17.69	20.57	18.56	17.94	18.03		
XX (n = 17)	16.77	18.83	15.55	13.71	13.69	10.12	12.84	11.41	15.24	19.20	21.85	12.91	13.28	16.78	15.10	13.33	13.10	16.14	
XXI (n = 51)	19.83	21.76	18.52	16.81	16.72	11.88	15.06	14.44	18.08	21.81	24.41	15.46	16.02	18.61	18.23	16.68	15.72	18.51	10.78

a Inferred from the complete nucleotide F gene sequences.

b The sequences from genotype XV are identified as recombinant forms and are not included in this analysis.

c The number of base substitutions per site from averaging all sequence pairs between class II genotypes is shown. Analysis was conducted using the Maximum Composite Likelihood model (Tamura et al., 2004). The rate variation among sites was modeled with a gamma distribution (shape parameter = 1). The analysis involved 1664 nucleotide sequences (8 unclassified sequences not assigned as members of any genotype were excluded from the analysis, UNCL 1–8, see Supplemental Table. S2). Codon positions included were 1st, 2nd, 3rd and noncoding. All positions containing gaps and missing data were eliminated. Evolutionary analyses were conducted in MEGA6 (Tamura et al., 2013). Shaded cell represents inter-genotype nucleotide distance that is lower than 10%.

**Table 5:** Side-by-side comparison between the “lineage” system (Aldous et al., 2003), the “genotype” system (Diel et al., 2012a), and the updated classification system for NDV isolates proposed in this study. The names in bold font represent the sub/genotypes names based on the new classification and nomenclature systems

Sub/lineage Aldous et al., 2003	Sub/genotype Diel et al., 2012a, Diel et al., 2012b	Current study
Class II		
1	I a	**I.1.1**
	I b	**I.2.**
	I c	**I.1.2.1**
	I d	**I.1.2.2**
2	II	**II**
3a, 3f	III	**III**
3b	IV	**IV**
3c	V b	**V; V.1**
	V c	**V.2**
	V d	**V**
*4bii e*	VI a	**VI.2.1.1.1**
	VI n	**VI.2.1.1.1**
4b, *4bi*,	VI b	**VI.1.1**
*4bii d*	VI e	**VI.1.2.2.2**
	VI f	**VI.1.2.2.1**
	VI h	**VI.1.2.1.2**
*4bii f*	VI j	**VI.2.1.1.2.1**
	VI k	**VI.2.1.1.2.2**
4c	–	**–**
5a	VII a – one sequence VIIi	**VII.2**
5d	VII b	**VII.1.1**
	VII b	**VII.1.1**
	VII j	**VII.1.1**
	VII l	**VII.1.1**
5c	VII e	**VII.1.1**
	VII f	**VII.1.2**
–	VII g (RF)	**–**
5a	VII h	**VII.2**
–	VII k	**VII.2**
5e	VII	**VII, VII.2**
3d	VIII	**VIII**
3e	IX	**IX**
1	X a	**X**
	X b	**X**
3 g	XI	**XI**
5b	XII a	**XII.1**
	XII b	**XII.2**
5b	XIII a	**XIII.1.1**
	XIII a	**XIII.1.2**
	XIII b	**XIII.2.1**
	XIII b	**XIII.2.2**
5 h, 7d	XIV	**XIV**
5f, 7c	XIV a	**XIV.1**
	XIV b	**XIV.2**
–	XV (RF)	**XV (RF)**
3d	XVI	**XVI**
5 g, 7a	XVII a	**XVII**
	XVII b	**XVII**
7b	XVIII a	**XVIII.1**
	XVIII b	**XVIII.2**
3c	V a	**XIX**
4a, 4d	VI c	**XX**
–	VI l	**XXI**
–	VI i	**XXI.2**
–	VI g	**XXI.1.1**
–	VI m	**XXI.1.2**

Class I		
6	1a	**1.1.1**
	1b	**1.1.2**
	1c	**1.2**
	1d	**1.2**

Genotype XX contains some of the oldest available NDV isolates, previously identified as members of genotype VI (former sub-genotype VIc). Notably, genotype XX outgroups genotype VI, and all genotype XX viruses have been isolated from chickens. This new genotype XX includes viruses from Asia and Europe isolated between 1985 and 2011 (Umali et al., 2014).

**Fig. 2:**
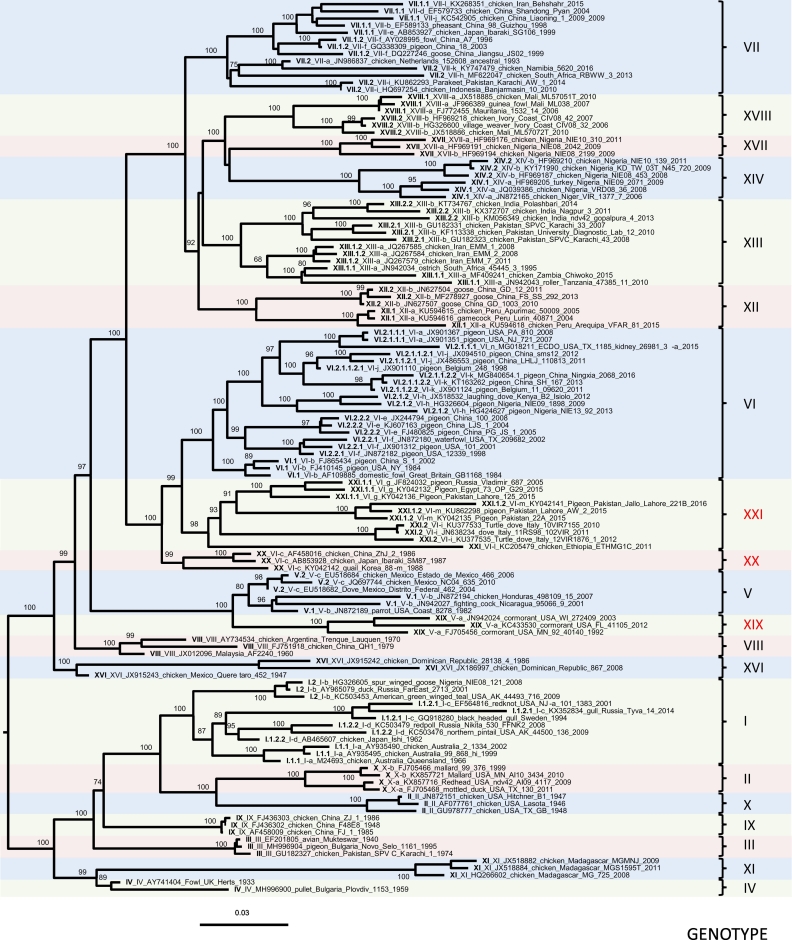
Class II “pilot” tree. Phylogenetic analysis is based on the full-length nucleotide sequence of the fusion gene of selected isolates representing all class II Newcastle disease virus sub/genotypes (n = 125). The evolutionary history was inferred by using RaxML (Stamatakis, 2014) and utilizing the Maximum Likelihood method based on the General Time Reversible model with 1000 bootstrap replicates. The tree with the highest log likelihood (−28,785.59) is shown. A discrete gamma distribution was used to model evolutionary rate differences among sites and the rate variation model allowed for some sites to be evolutionarily invariable. The Roman numerals presented in the taxa names in the phylogenetic tree represent the respective genotype for each isolate. The new (decimal naming) and the old names (alpha-numerical) are provided for easier comparison. The taxa names also include the GenBank identification number, host name, country of isolation, strain designation, and year of isolation. Three new genotypes assigned in the current study are highlighted in red font. The trees are drawn to scale, with branch lengths measured in the number of substitutions per site. (For interpretation of the references to color in this figure legend, the reader is referred to the web version of this article.)

Genotype XXI contains viruses isolated from chickens and pigeons in different Asian, European, and African countries between 2005 and 2016 (Sabra et al., 2017; Snoeck et al., 2013a; Van Borm et al., 2012; Wajid et al., 2016). Similarly to genotype XX, genotype XXI viruses were previously assigned as members of genotype VI. At the root of genotype XXI is a clade of chicken viruses, isolated in Ethiopia between 2011 and 2012, that have previously been assumed to form a separate sub-genotype VIl (Mulisa et al., 2014; Servan de Almeida et al., 2013). However, as the group lacked a sufficient number of independent isolates, these viruses were assigned to the lower order, namely XXI. Three sub-genotypes were identified within genotype XXI – XXI.2, XXI.1.1, and XXI.1.2 corresponding to former sub-genotypes VIi, VIg, and VIm, respectively (see Table 5, Supplemental Fig. S5B).

Construction of the class II phylogenetic trees with any of the used methods and utilizing all available complete fusion gene sequences occasionally resulted in lack of branch support, presence of polytomies, and disruption of monophyletic branches (i.e. previously identified genotypes not grouping into a single branch) (Supplemental Fig. S5 A–C). For example, viruses of genotype IV did not fall into a monophyletic branch, there was lack of branch support between groups in genotype VII, and polytomies were present in different branches. These issues will likely be resolved when additional sequences from these groups become available (Purvis and Garland, 1993). Indeed, the analysis using the concatenated complete genome coding sequences overcame the flaws observed when the complete fusion gene sequences were used for the analyses (Supplemental Fig. S6). Despite the fact that complete genomes provide more reliable phylogenetic inferences (Heath et al., 2008), the lack of complete genome sequences in all NDV sub/genotypes and the complexity of the analysis, the use of complete genome phylogenetic reconstruction to resolve NDV classification issues or for definitive classification, is not warranted at this time. Certainly, with the increasing use of high-throughput sequencing technologies and the development of more powerful computational resources, it is logical to suggest that the availability of complete genomes will increase in the near future. This will probably allow this classification to be revisited with more complete genomic data. In addition, genome size is no longer needed as a criterion for separation of NDV isolates into classes as there are sequences in genotype XIX of class II that have the same genome size as class I NDV (15,198 nucleotides). As the complete fusion gene mean nucleotide distance between the two classes is 59.25% and the individual distances between all class II genotypes and class I is above 40%, a cut-off of 40% nucleotide distance of the F gene between NDV classes is suggested here.

#### 3.2.3 Pilot tree and dataset

Building a phylogenetic tree with a lower number of sequences, that still provides topology consistent to the larger tree, significantly decreases the time for the tree construction. To ensure that the topology of the class II genotypes would be maintained if fewer isolates were used, we performed a ML phylogenetic reconstruction with a smaller “pilot” dataset (n = 125, Supplemental Table S3). The generated tree (named “pilot” tree) is depicted in Fig. 2 and the congruence analysis showed complete consistency with the ML tree inferred using the larger class II fusion gene dataset (Supplementary Fig. S5B). Therefore, this pilot dataset can be utilized for rapid preliminary genotype identification of new isolates. A similar approach has been previously suggested by Aldous et al. (Aldous et al., 2003). However, for naming new genotypes and sub-genotypes, building a tree using the complete curated F gene dataset is required.

#### 3.2.4 Rooted sub-trees for each class II genotype

To further facilitate fast characterization of newly isolated NDVs, sub-trees utilizing all sequences within each genotype of class II that contains sub-genotypes, were constructed (Supplemental Fig. S7 A–I). To maintain consistent topology with the larger class II tree and to provide inference of branch support at the first defining node, each sub-tree was rooted with at least two sequences. A list of sequences to be used for rooting of each genotypes' sub-tree is provided in Supplemental Table S5. Proper sub-tree rooting appeared to be crucial for consistent topology between the larger tree and the sub-trees. Attempts to root the sub-trees with random sequences from different genotypes resulted in topologies largely incongruent to the full tree (data not shown). In addition, lower branch support was observed in some trees and for definitive classification, building a tree with the complete F gene dataset is recommended.

### 3.3 New nomenclature criteria

Assuming that each node has two immediate descendants (partitions into two branches), a dichotomous naming system using the Arabic numerals 1 and 2 was used at every node at which sub-genotypes were separated. Arguably, strict following of the classification and nomenclature criteria proposed here, will prevent duplicate names of future sub-genotypes, as has happened before with the previous systems. In addition, when using this approach, the names of the sub-genotypes of higher order (closer to the tips), bear ancestral information for the sub/genotypes they split from. Of note, the use of the new naming criteria resulted in complex names for two of the genotype VI sub-genotypes – VI.2.1.1.2.1 and VI.2.1.1.2.2. However, the viruses from these two genotypes are not widely distributed, and their names would likely not have negative impact on the global use of the system. Similar decimal systems are used for classification of swine influenza and highly pathogenic H5N1 avian influenza viruses (Anderson et al., 2016; WHO/OIE/FAO-H5N1-EWG, 2008). A detailed comparison between the nomenclature used in the lineage system (Aldous et al., 2003; Cattoli et al., 2010), the genotype system (Courtney et al., 2013; Diel et al., 2012a; Snoeck et al., 2013b) and the nomenclature suggested here, is provided in Table 5.

The worldwide circulation and constant evolution of NDV will, expectedly, lead to emergence of new NDV genetic variants that will form clades fulfilling all criteria for separate genotypes. To ensure consistent naming, specific nomenclature criteria need to be utilized for newly identified genetic diversity. Updated guidelines for naming new genotypes are also outlined in Table 2. Newly identified virus diversity (a group of viruses undescribed before), that meets all classification criteria, will be classified as new genotype and will receive a subsequent Roman numeral (e.g. currently XXII is the next available) (example in Supplemental Fig. S2, red color). If a new sub-genotype of viruses is identified, but still falls into a higher order (not at the first defining node) within an existing genotype, this new sub-genotype receives the next consecutive sub-genotype numerical address for the respective level of order in the phylogeny. This is necessary to avoid re-numbering of all existing sub-genotypes that are of higher order (closer to the tip). For example, if a new sub-genotype that outgroups VII.1.1 and VII.1.2 is identified but it is still within sub-genotype VII.1, this new sub-genotype will be named VII.1.3 (see Supplemental Fig. S3 for an example). Existing sub-genotypes that fulfill the genotype criteria will not be designated with a new genotype name and will continue bearing the name assigned in the current classification (examples in Supplemental Fig. S2, green color).

### 3.4 Software for phylogenetic inference

The increased number of available NDV sequences has led to a higher demand of computational resources. To this end, in addition to the widely used MEGA (Tamura et al., 2013), and PhyML software (Guindon et al., 2010), for example, tools like FastTree (Price et al., 2010), Garli (Bazinet et al., 2014), and RaxML (Stamatakis, 2014), have been extensively used for faster phylogenetic inferences. However, some of these utilize the Shimodaira-Hasegawa test to estimate nodal support (FastTree, PhyML), which requires multiple additional steps to calculate the traditional bootstrap value (Felsenstein, 1985), commonly used in NDV phylogenetic analyses. Based on its increased productivity and reliable results, the RaxML tool (v.8.2.11) was utilized for ML inference in this study. Interestingly, when the invariable sites option is used with RaxML, the tool automatically sets the model optimization precision (in likelihood units) to 0.001 to avoid unfavorable effects caused by simultaneous optimization of gamma distribution and invariable sites. Although the combination GTR+ Γ + I has been widely used, it is suggested that distinct approaches to incorporate rate heterogeneity (e.g. Γ + I) should not be used at the same time and concerns have been raised that this might cause problems during the model parameter optimization process (Stamatakis, 2016). Indeed, GTR+ Γ + I is often estimated as the best-fit model for NDV datasets based on the corrected Akaike information criterion. However, in most cases, trees constructed with this model using the MEGA software lack branch support at established defining nodes or contain multiple polytomies. Disabling the option for invariable sites in MEGA results in better branch support and resolving most polytomies (data not shown). Goodness-of-fit of models has to be established periodically in the future, as adding new isolates to the datasets may result in change of the best-fit model.

The advances in molecular sequencing technologies, the increasing surveillance efforts, and the significantly larger amount of genetic data collected worldwide, have transformed the field of phylogenetic inference into a computational science (Stamatakis et al., 2012). Therefore, faster tools, specifically ones that can be used in high-performance computer (HPC) environments, are needed. In this study, to test the utilization of HPC for NDV phylogenetic inference, the class II ML tree using the bigger complete F gene dataset was built again through the free CIPRES Science Gateway (Miller et al., 2010a) using RaxML-HPC (v.8.2.10) that allows multi-threading by message-passing interface. The use of the supercomputer shortened the construction of a fully congruent tree to less than two hours (compared to two days when using 30 cores, and two weeks when using a single computer). Step-by-step guidelines with all settings needed for ML tree construction through CIPRES (the same settings can be used for any of the freely available HPC) are provided in Supplemental Fig. S8 (provided as screenshots from http://www.phylo.org). Additional guidelines and video tutorials regarding the use of the CIPRES Science Gateway are available online (http://www.phylo.org/index.php/help/).

### 3.5 Discrepancies (exceptions) to the established criteria

The nucleotide distance between genotypes III and IX was estimated to be below 10% (8.62%) (Table 3) with topology not fully resolved. Some sequences, previously identified as members of genotype IV, did not form a monophyletic branch (Supplemental Fig. S5B). However, genotypes III and IV contain historical viruses that are believed to no longer naturally circulate (Dimitrov et al., 2016a). For consistency with the existent literature, the nomenclature and classification of these viruses were maintained. In addition, the genotype IV viruses from Nigeria could have evolved into a separate genotype as they formed a separate branch, but have probably become extinct as similar viruses have not been isolated since 1980.

### 3.6 Dataset availability

To facilitate future analyses, all complete fusion gene datasets, generated in this study, are provided as Supplemental materials – see Supplemental Table S1, Supplemental Table S2, Supplemental Table S3. The resulting alignments are also provided as Supplemental materials (Supplemental Table S1, Supplemental Table S2, Supplemental Table S3). Updated datasets will be deposited at the GitHub repository (https://github.com/NDVconsortium/NDV_Sequence_Datasets). The utilization of these alignments is highly recommended for NDV classification needs.

## 4 Conclusions

The classification and nomenclature system proposed here is a result of the collaboration of 40 experts from 29 institutions on six continents, including all OIE NDV reference laboratories. The revised system includes objective criteria for identification of new NDV isolates and naming NDV sub/genotypes. Curated, up-to-date, complete F gene class I and class II NDV sequence datasets are provided for public use. In addition, a pilot dataset and rooting guidelines that allow rapid preliminary genotype identification of new isolates are included. To increase the speed of phylogenetic inference and to enable the use of consistent methods between laboratories, detailed guidelines for HPC use are also provided. In order to avoid super-delineation, more stringent criteria were proposed, and these resulted in the merging of multiple former sub-genotypes. However, the increased viral diversity due to constant NDV evolution and the significant increase of available sequences led to the naming of three new genotypes. The global adoption of the proposed consensus system will facilitate future studies on NDV evolution and epidemiology, and will make comparisons of results obtained across the world easier. Newcastle disease viruses, as any other RNA viruses, are rapidly evolving, and periodic (e.g. five years) revisiting of the consensus classification and nomenclature system proposed here, led by a working group from this consortium, is warranted.

The following are the supplementary data related to this article.

Supplemental Table S1 Complete fusion gene curated dataset of class I NDV used in this study. The green shading represents congruence between trees. Red font represents lack of branch support at the defining node. The naming in bold font (ML naming) is according to the nomenclature suggested in this study. The dataset contains 284 sequences and was used to build the trees depicted in Fig. 1A and Supplemental Fig. S4 A–C. “UNCL” = unclassified. Supplemental Table S1

Supplemental Table S2 Complete fusion gene curated dataset of class II NDV used in this study. The green shading represents congruence between trees. Red font represents lack of branch support at the defining node. The naming in bold font (ML naming) is according to the nomenclature suggested in this study. The dataset contains 1672 sequences and was used to build the trees depicted in Fig. 1B and Supplemental Fig. S5 A–C. “UNCL” = unclassified. Supplemental Table S2

Supplemental Table S3 Complete fusion gene “pilot” dataset of class II NDV used in this study. The dataset contains 125 sequences and was used to build the tree depicted in Fig. 2. Supplemental Table S3

Supplemental Table S4 Former sub-genotypes within class I and class II that did not pass the updated classification criteria (either one of 5% nucleotide distance, branch support equal or above 70%, at least 4 independent isolates) and were merged. The sub/genotype designation follows the newly proposed nomenclature and the former names are provided in parenthesis. Supplemental Table S4

Supplemental Table S5 Recommended sequences to be used for rooting purposes when building sub-trees for separate analysis of each genotype within class II. The sequence to use for rooting is marked with an asterisk. These sequences were used to root the trees presented in Supplemental Fig. S7 A–I. Supplemental Table S5

Supplemental Fig. S1 Examples of dichotomous naming at nodes at which sub-genotypes are separated. The names of genotypes and sub-genotypes used in this figure do not correspond to the names in the phylogenetic trees presented in the current study. The names in this figure were used for illustration and demonstration purposes. Supplemental Fig. S1

Supplemental Fig. S2 Examples for naming new genotypes when they outgroup or not existing genotypes. The names of genotypes and sub-genotypes used in this figure do not correspond to the names in the phylogenetic trees presented in the current study. The names in this figure were used for illustration and demonstration purposes. Supplemental Fig. S2

Supplemental Fig. S3 Examples for naming new sub-genotypes when they fall into a higher order of an existing genotype. The names of genotypes and sub-genotypes used in this figure do not correspond to the names in the phylogenetic trees presented in the current study. The names in this figure were used for illustration and demonstration purposes. Supplemental Fig. S3

Supplemental Fig. S4 Full phylogenetic trees based on the complete fusion gene sequences of isolates representing Newcastle disease virus class I (n = 284). A. Neighbor-joining tree; B. Maximum Likelihood tree; C. Bayesian tree. The Roman numerals presented in the taxa names in the phylogenetic trees represent the respective genotype for each isolate. The new (decimal naming) and the old names (alpha-numerical) are provided for easier comparison. The numbers after the genotype names represent database ID. The names based on the new system differ for some genotypes in the different trees based on differences in topology. The taxa names also include the GenBank identification number, and when available – host name, country of isolation, strain designation, and year of isolation. The trees are drawn to scale, with branch lengths measured in the number of substitutions per site. Supplemental Fig. S4

Supplemental Fig. S5 Full phylogenetic trees based on the complete fusion gene sequences of isolates representing Newcastle disease virus class II (n = 1672). A. Neighbor-joining tree; B. Maximum Likelihood tree; C. Bayesian tree. The Roman numerals presented in the taxa names in the phylogenetic trees represent the respective genotype for each isolate. The new (decimal naming) and the old names (alpha-numerical) are provided for easier comparison. The numbers after the genotype names represent database ID. The names based on the new system differ for some genotypes in the different trees based on differences in topology. The taxa names also include the GenBank identification number, and when available – host name, country of isolation, strain designation, and year of isolation. The trees are drawn to scale, with branch lengths measured in the number of substitutions per site. Supplemental Fig. S5

Supplemental Fig. S6 Full phylogenetic tree based on the complete genome concatenated coding sequences of isolates representing Newcastle disease virus class II (n = 491). The evolutionary history was inferred by using the Maximum Likelihood method based on the General Time Reversible model with 1000 bootstrap replicates. The tree with the highest log likelihood (−344,411.91) is shown. A discrete gamma distribution was used to model evolutionary rate differences among sites and the rate variation model allowed for some sites to be evolutionarily invariable. The Roman numerals presented in the taxa names in the phylogenetic tree represent the respective genotype for each isolate. The new (decimal naming) and the old names (alpha-numerical) are provided for easier comparison. The names of sub/genotypes are based on the proposed system and as identified by the ML complete fusion gene analysis. The taxa names also include the GenBank identification number, host name, country of isolation, strain designation, and year of isolation. The tree is drawn to scale, with branch lengths measured in the number of substitutions per site. Supplemental Fig. S6

Supplemental Fig. S7 Phylogenetic sub-tree based on the complete fusion gene sequence of isolates representing Newcastle disease virus genotypes. A. Genotype I (n = 122); B. Genotype V (n = 49); C. Genotype VI (n = 267); D. Genotype VII (n = 774); E. XII. Genotype I (n = 25); F. Genotype XIII (n = 74); G. Genotype XIV (n = 79); H. Genotype XVIII (n = 19); I. Genotype XXI (n = 53). The trees were built using all sequences within each NDV genotypes and 2 to 4 additional sequences used for rooting. The sequences recommended for use for rooting are also provided in Supplemental Table S5. The evolutionary history was inferred by using the Maximum Likelihood method based on the General Time Reversible model with 1000 bootstrap replicates. The trees with the highest log likelihood are shown. A discrete gamma distribution was used to model evolutionary rate differences among sites and the rate variation model allowed for some sites to be evolutionarily invariable. The Roman numerals presented in the taxa names in the phylogenetic tree represent the respective genotype for each isolate. The new (decimal naming) and the old names (alpha-numerical) are provided for easier comparison. The taxa names also include the GenBank identification number, and when available - host name, country of isolation, strain designation, and year of isolation. The trees are drawn to scale, with branch lengths measured in the number of substitutions per site. Supplemental Fig. S7

Supplemental Fig. S8 Step-by-step guidelines of settings for building Maximum likelihood trees using the CIPRES Science Gateway (provided as screenshots from http://www.phylo.org). Supplemental Fig. S8

Supplemental Dataset S1 Newcastle disease virus class I curated complete fusion gene dataset. Supplemental Dataset S1

Supplemental Dataset S2 Newcastle disease virus class II curated complete fusion gene dataset. Supplemental Dataset S2

Supplemental Dataset S3 Newcastle disease virus class II pilot complete fusion gene dataset for rapid genotype identification of isolates. Supplemental Dataset S3
