# Supplementary material for: Updated unified phylogenetic classification system and revised nomenclature for Newcastle disease virus
Source: Infect Genet Evol. 2019 Oct;74:103917. doi: 10.1016/j.meegid.2019.103917 (PMC6876278; doi:10.1016/j.meegid.2019.103917)
Supplement: Supplemental Table S1 — Complete fusion gene curated dataset of class I NDV used in this study. The green shading represents congruence between trees. Red font represents lack of branch support at the defining node. The naming in bold font (ML naming) is according to the nomenclature suggested in this study. The dataset contains 284 sequences and was used to build the trees depicted in Fig. 1A and Supplemental Fig. S4 A–C. “UNCL” = unclassified. [file mmc1.pdf]

Supplemental Table S1. Complete fusion gene curated dataset of class I NDV used in this study. The green shading represents congruence between trees. Red font represents lack of branch support at the defining node. The naming in bold font (ML naming) is according to the nomenclature suggested in this study. The dataset contains 284 sequences and was used to build the trees depicted in Supplemental Fig. S4 A-C.

red font = lack of branch support	
**bold font = naming based on the criteria accepted in this study**	
green shading = congruence between trees	

**Based on Diel et al. 2012**	**Sub/genotypes based on the new classification and nomenclature system**								
**Genotype**	**NJ**	**ML**	**BAYES**	**ID**	**Acc. Number**	**Host**	**Country**	**Isolate**	**Year**
1a	1.1.1	**1.1.1**	1.1.1	1	FJ487637	duck	China	08_004	2008
1a	1.1.1	**1.1.1**	1.1.1	2	GQ245777	chicken	China	XZ_35	2007
1a	1.1.1	**1.1.1**	1.1.1	3	FJ597582	duck	China	JS_1	2007
1a	1.1.1	**1.1.1**	1.1.1	4	FJ597583	duck	China	JS_2	2007
1a	1.1.1	**1.1.1**	1.1.1	5	FJ597584	duck	China	JS_16	2005
1a	1.1.1	**1.1.1**	1.1.1	6	FJ597588	duck	China	JS_17	2006
1a	1.1.1	**1.1.1**	1.1.1	7	FJ597589	duck	China	JS_18	2005
1a	1.1.1	**1.1.1**	1.1.1	8	FJ597600	duck	China	ZJ_30	2005
1a	1.1.1	**1.1.1**	1.1.1	9	FJ597601	duck	China	ZJ_31	2005
1a	1.1.1	**1.1.1**	1.1.1	10	HQ398778	chicken	China	08_038	2008
1a	1.1.1	**1.1.1**	1.1.1	11	HQ398794	chicken	China	09_020	2009
1a	1.1.1	**1.1.1**	1.1.1	13	HQ398799	chicken	China	09_031	2009
1a	1.1.1	**1.1.1**	1.1.1	14	HQ398801	duck	China	09_042	2009
1a	1.1.1	**1.1.1**	1.1.1	15	HQ398802	chicken	China	09_043	2009
1a	1.1.1	**1.1.1**	1.1.1	16	HQ398803	chicken	China	09_044	2009
1a	1.1.1	**1.1.1**	1.1.1	18	HQ398805	chicken	China	09_047	2009
1a	1.1.1	**1.1.1**	1.1.1	20	HQ398807.1	duck	China	09_049	2009
1a	1.1.1	**1.1.1**	1.1.1	22	HQ398809	duck	China	09_052	2009
1a	1.1.1	**1.1.1**	1.1.1	23	HQ398810	duck	China	09_053	2009
1a	1.1.1	**1.1.1**	1.1.1	25	HQ398813	duck	China	09_056	2009
1a	1.1.1	**1.1.1**	1.1.1	26	HQ398814	duck	China	09_057	2009
1a	1.1.1	**1.1.1**	1.1.1	27	HQ997380	duck	China	10_011	2010
1a	1.1.1	**1.1.1**	1.1.1	29	HQ997387	chicken	China	10_064	2010
1a	1.1.1	**1.1.1**	1.1.1	30	HQ997388	chicken	China	10_067	2010

1a	1.1.1	**1.1.1**	1.1.1	33	HQ997397	chicken	China	10_089	2010
1a	1.1.1	**1.1.1**	1.1.1	34	JN688862.1	chicken	China	JS_09	2009
1a	1.1.1	**1.1.1**	1.1.1	35	JN688864.1	duck	China	AH_10	2010
1a	1.1.1	**1.1.1**	1.1.1	36	JN688865.1	goose	China	JS_09	2009
1b	1.1.2	**1.1.2**	1.1.2	37	HQ398788	duck	China	09_014	2009
1b	1.1.2	**1.1.2**	1.1.2	43	HQ398795	chicken	China	09_022	2009
1b	1.1.2	**1.1.2**	1.1.2	44	HQ997378	duck	China	10_005	2010
1b	1.1.2	**1.1.2**	1.1.2	45	HQ997379	duck	China	10_006	2010
1b	1.1.2	**1.1.2**	1.1.2	46	HQ997382	chicken	China	10_059	2010
1b	1.1.2	**1.1.2**	1.1.2	47	HQ997383	duck	China	10_060	2010
1b	1.1.2	**1.1.2**	1.1.2	51	HQ997389	chicken	China	10_069	2010
1b	1.1.2	**1.1.2**	1.1.2	52	HQ997390	chicken	China	10_072	2010
1b	1.1.2	**1.1.2**	1.1.2	53	HQ997391	chicken	China	10_079	2010
1b	1.1.2	**1.1.2**	1.1.2	54	HQ997392	chicken	China	10_083	2010
1b	1.1.2	**1.1.2**	1.1.2	55	HQ997393	chicken	China	10_084	2010
1b	1.1.2	**1.1.2**	1.1.2	56	HQ997394	chicken	China	10_085	2010
1b	1.1.2	**1.1.2**	1.1.2	57	HQ997398	chicken	China	10_090	2010
1b	1.1.2	**1.1.2**	1.1.2	58	AB858995.1	duck	China	J17_13	2013
1b	1.1.2	**1.1.2**	1.1.2	59	AB858996.1	duck	China	J72_13	2013
1b	1.1.2	**1.1.2**	1.1.2	60	AB858997.1	duck	China	J55_13	2013
1b	1.1.2	**1.1.2**	1.1.2	61	AB858998.1	duck	China	J77_13	2013
1b	1.1.2	**1.1.2**	1.1.2	62	AB858999.1	duck	China	J80_13	2013
1b	1.1.2	**1.1.2**	1.1.2	63	AB859000.1	chicken	China	J36_13	2013
1b	1.1.2	**1.1.2**	1.1.2	64	AB859001.1	duck	China	J70_13	2013
1b	1.1.2	**1.1.2**	1.1.2	65	AB859002.1	chicken	China	J2_13	2013
1b	1.1.2	**1.1.2**	1.1.2	66	KJ499462.1	Black_Swan	China	LHLJ_120608	2012
1b	1.1.2	**1.1.2**	1.1.2	67	KJ607171.1	peacock	China	LHLJ_131047	2013
1b	1.1.2	**1.1.2**	1.1.2	68	KM669995.1	egret	China	SD21_13	2013
1b	1.1.2	**1.1.2**	1.1.2	69	KM669996.1	heron	China	SD22_13	2013
1b	1.1.2	**1.1.2**	1.1.2	70	KM885150.1	duck	China	LZJ_068	2011
1b	1.1.2	**1.1.2**	1.1.2	71	KM885151.1	duck	China	LJS_215	2011
1b	1.1.2	**1.1.2**	1.1.2	72	KM885152.1	duck	China	LZJ_067	2011
1b	1.1.2	**1.1.2**	1.1.2	73	KM885153.1	duck	China	LJS_016	2011
1b	1.1.2	**1.1.2**	1.1.2	74	KM885155.1	duck	China	LGX_298	2011
1b	1.1.2	**1.1.2**	1.1.2	75	KM885156.1	duck	China	LFJ_048	2011
1b	1.1.2	**1.1.2**	1.1.2	76	KM885157.1	duck	China	LGD_364	2011
1b	1.1.2	**1.1.2**	1.1.2	78	KM885159.1	duck	China	LFJ_104	2011
1b	1.1.2	**1.1.2**	1.1.2	79	KM885160.1	duck	China	LFJ_027	2011
1b	1.1.2	**1.1.2**	1.1.2	80	KM885161.1	duck	China	LHuB_085	2013
1b	1.1.2	**1.1.2**	1.1.2	81	KM885162.1	duck	China	LGX_280	2013
1b	1.1.2	**1.1.2**	1.1.2	82	KM885163.1	duck	China	LFJ_073	2013

1b	1.1.2	**1.1.2**	1.1.2	84	KM885168.1	duck	China	LGD_358	2011
1b	1.1.2	**1.1.2**	1.1.2	85	HQ398812	duck	China	NDV09_055	2009
1b	1.1.2	**1.1.2**	1.1.2	86	KT223818.1	pigeon	China	Qinghai_01	2014
1b	1.1.2	**1.1.2**	1.1.2	88	KT892746.1	chicken	China	JL_CC02	2015
1b	1.1.2	**1.1.2**	1.1.2	89	KT892748.1	chicken	China	JL_CC03	2014
1b	1.1.2	**1.1.2**	1.1.2	94	KT381589.1	chicken	China	Guangdong_GM307	2014
1b	1.1.2	**1.1.2**	1.1.2	95	KT381588.1	chicken	China	Guangdong_GM3	2013
1b	1.1.2	**1.1.2**	1.1.2	96	KT381587.1	pigeon	China	Guangdong_YF1	2014
1b	1.1.2	**1.1.2**	1.1.2	97	KT381586.1	duck	China	Guangdong_YF827	2014
1b	1.1.2	**1.1.2**	1.1.2	99	KX602322.1	Sheldrake_Duck	China	Guizhou_01	2016
1b	1.1.2	**1.1.2**	1.1.2	100	KX602323.1	Sheldrake_Duck	China	Guizhou_02	2016
1b	1.1.2	**1.1.2**	1.1.2	101	KY788675.1.	Oriental_magpie_robin	China	GD_ZS44	2015
1b	1.1.2	**1.1.2**	1.1.2	102	KY788674.1.	Chinese_bulbul	China	GD_ZS23	2015
1b	1.1.2	**1.1.2**	1.1.2	272	KY776595.1	wild_bird	China	LTL130830	
1b	1.1.2	**1.1.2**	1.1.2	274	MF100763.1	duck	China	D_GD_GZ_817_2016	2016
1b	1.1.2	**1.1.2**	1.1.2	276	MF100761.1	pigeon	China	P_GD_HZ_599	2016
1b	1.1.2	**1.1.2**	1.1.2	277	MF100760.1	pigeon	China	P_GD_DG_466	2016
1b	1.1.2	**1.1.2**	1.1.2	278	MF100759.1	pigeon	China	P_GD_GZ_178	2016
1b	1.1.2	**1.1.2**	1.1.2	279	MF100758.1	goose	China	G_GD_DG_571	2016
1b	1.1.2	**1.1.2**	1.1.2	280	MF100757.1	goose	China	G_GD_HZ_460	2016
1b	1.1.2	**1.1.2**	1.1.2	281	MF100756.1	goose	China	G_GD_YF_401	2016
1b	1.1.2	**1.1.2**	1.1.2	282	MF100755.1	goose	China	G_GD_YF_392	2016
1b	1.1.2	**1.1.2**	1.1.2	283	MF100754.1	environment_LBM	China	E_GD_DG_168	2016
1b	1.1.2	**1.1.2**	1.1.2	284	MF100753.1	environment_LBM	China	E_GD_YF_580	2016
1b	1.1.2	**1.1.2**	1.1.2	285	MF100752.1	environment_LBM	China	E_GD_YF_408	2016
1b	1.1.2	**1.1.2**	1.1.2	286	MF100751.1	environment_LBM	China	E_GD_HZ_406	2016
1b	1.1.2	**1.1.2**	1.1.2	287	MF100750.1	environment_LBM	China	E_GD_GZ_402	2016
1b	1.1.2	**1.1.2**	1.1.2	288	MF100749.1	environment_LBM	China	E_GD_DG_12	2016
1b	1.1.2	**1.1.2**	1.1.2	289	MF100748.1	duck	China	D_GD_GZ_825	2016
1b	1.1.2	**1.1.2**	1.1.2	290	MF100747.1	duck	China	D_GD_DG_822	2016
1b	1.1.2	**1.1.2**	1.1.2	291	MF100746.1	duck	China	D_GD_YF_706	2016
1b	1.1.2	**1.1.2**	1.1.2	292	MF100745.1	duck	China	D_GD_YF_458	2016
1b	1.1.2	**1.1.2**	1.1.2	293	MF100744.1	duck	China	D_GD_YF_193	2016
1b	1.1.2	**1.1.2**	1.1.2	294	MF100743.1	duck	China	D_GD_HZ_154	2016
1b	1.1.2	**1.1.2**	1.1.2	295	MF100742.1	chicken	China	C_GD_HZ_140	2016
1b	1.1.2	**1.1.2**	1.1.2	296	MF100741.1	chicken	China	C_GD_GZ_999	2016
1b	1.1.2	**1.1.2**	1.1.2	298	MF100739.1	chicken	China	C_GD_DG_707	2016
1b	1.1.2	**1.1.2**	1.1.2	299	MF100738.1	chicken	China	C_GD_DG_698	2016
1b	1.1.2	**1.1.2**	1.1.2	300	MF100737.1	chicken	China	C_GD_DG_618	2016
1b	1.1.2	**1.1.2**	1.1.2	301	MF100736.1	chicken	China	C_GD_YF_609	2016
1b	1.1.2	**1.1.2**	1.1.2	302	MF100735.1	chicken	China	C_GD_YF_607	2016

1b	1.1.2	**1.1.2**	1.1.2	303	MF100734.1	chicken	China	C_GD_YF_445	2016
1b	1.1.2	**1.1.2**	1.1.2	304	MF100733.1	chicken	China	C_GD_DG_410	2016
1b	1.1.2	**1.1.2**	1.1.2	305	MF100732.1	chicken	China	C_GD_HZ_404	2016
1b	1.1.2	**1.1.2**	1.1.2	306	MF100731.1	chicken	China	C_GD_HZ_398	2016
1b	1.1.2	**1.1.2**	1.1.2	307	MF100730.1	chicken	China	C_GD_HZ_390	2016
1b	1.1.2	**1.1.2**	1.1.2	308	MF100729.1	chicken	China	C_GD_GZ_191	2016
1b	1.1.2	**1.1.2**	1.1.2	309	MF100728.1	chicken	China	C_GD_GZ_150	2016
1b	1.1.2	**1.1.2**	1.1.2	310	MF100727.1	chicken	China	C_GD_GZ_148	2016
1b	1.1.2	**1.1.2**	1.1.2	311	MK122776.1	chicken	China	SC_PT3	2016
1b	1.1.2	**1.1.2**	1.1.2	313	MH274991.1	chicken	China	GX_26	2015
1b	1.1.2	**1.1.2**	1.1.2	314	MH274992.1	chicken	China	GX_65	2015
1b	1.1.2	**1.1.2**	1.1.2	317	MH289953.1	Chicken	China	HN_41M2	2017
1b	1.1.2	**1.1.2**	1.1.2	323	MH289959.1	Chicken	China	HN_53L2	2017
1b	1.1.2	**1.1.2**	1.1.2	324	MH289960.1	Duck	China	JX_56I2	2016
1b	1.1.2	**1.1.2**	1.1.2	327	MH289963.1	Chicken	China	JX_50I2	2016
1b	1.1.2	**1.1.2**	1.1.2	330	MH289966.1	Environment	China	JX_55U	2016
1b	1.1.2	**1.1.2**	1.1.2	334	MH289970.1	Environment	China	JX_43X	2016
1b	1.1.2	**1.1.2**	1.1.2	336	MH289932.1	Chicken	China	HN_6W1	2016
1b	1.1.2	**1.1.2**	1.1.2	348	MH289944.1	Duck	China	JX_69H2	2016
1b	1.1.2	**1.1.2**	1.1.2	350	MH289946.1	Chicken	China	JX_19H2	2016
1b	1.1.2	**1.1.2**	1.1.2	352	MH289948.1	Duck	China	JX_57C2	2016
1b	1.1.2	**1.1.2**	1.1.2	353	MH289949.1	Environment	China	JX_Y94	2016
1b	1.1.2	**1.1.2**	1.1.2	354	MH289950.1	Environment	China	JX_Y95	2016
1b	1.1.2	**1.1.2**	1.1.2	357	MH289913.1	Wild_birds	China	HB_F2B6_2	2017
1b	1.1.2	**1.1.2**	1.1.2	358	MH289914.1	Environment	China	JX_57U	2016
1b	1.1.2	**1.1.2**	1.1.2	361	MH289917.1	Environment	China	JX_13M	2016
1b	1.1.2	**1.1.2**	1.1.2	362	MH289918.1	Environment	China	JX_37U	2016
1b	1.1.2	**1.1.2**	1.1.2	363	MH289919.1	Duck	China	JX_Y82	2016
1b	1.1.2	**1.1.2**	1.1.2	364	MH289920.1	Environment	China	JX_Y98	2016
1b	1.1.2	**1.1.2**	1.1.2	365	MH289921.1	Duck	China	JX_57A2	2016
1b	1.1.2	**1.1.2**	1.1.2	366	MH289922.1	Duck	China	HN_K86	2017
1b	1.1.2	**1.1.2**	1.1.2	374	MH289930.1	Chicken	China	HN_K200	2017
1b	1.1.2	**1.1.2**	1.1.2	377	MH289893.1	Environment	China	HN_79S	2016
1b	1.1.2	**1.1.2**	1.1.2	378	MH289894.1	Chicken	China	HN_Y15	2016
1b	1.1.2	**1.1.2**	1.1.2	382	MH289898.1	Environment	China	HN_K67	2017
1b	1.1.2	**1.1.2**	1.1.2	383	MH289899.1	Environment	China	JX_47X	2016
1b	1.1.2	**1.1.2**	1.1.2	385	MH289901.1	Environment	China	JX_108G2	2017
1b	1.1.2	**1.1.2**	1.1.2	389	MH289905.1	Chicken	China	JX_82G2	2017
1b	1.1.2	**1.1.2**	1.1.2	392	MH289908.1	Chicken	China	JX_30F2	2017
1b	1.1.2	**1.1.2**	1.1.2	394	MH289910.1	Chicken	China	HN_19B2	2016
1b	1.1.2	**1.1.2**	1.1.2	397	MH289873.1	Environment	China	JX_192X	2016

1b	1.1.2	**1.1.2**	1.1.2	398	MH289874.1	Environment	China	HN_74W	2016
1b	1.1.2	**1.1.2**	1.1.2	399	MH289875.1	Chicken	China	HN_15W1	2016
1b	1.1.2	**1.1.2**	1.1.2	400	MH289876.1	Environment	China	HN_54S	2016
1b	1.1.2	**1.1.2**	1.1.2	402	MH289878.1	Chicken	China	HN_73V1	2016
1b	1.1.2	**1.1.2**	1.1.2	403	MH289879.1	Chicken	China	HN_Y10	2016
1b	1.1.2	**1.1.2**	1.1.2	405	MH289881.1	Environment	China	HN_78T	2016
1b	1.1.2	**1.1.2**	1.1.2	406	MH289882.1	Chicken	China	HN_91N	2016
1b	1.1.2	**1.1.2**	1.1.2	409	MH289885.1	Environment	China	HN_49S	2016
1b	1.1.2	**1.1.2**	1.1.2	414	MH289890.1	Environment	China	HN_89S	2016
1b	1.1.2	**1.1.2**	1.1.2	417	MH289856.1	Environment	China	HN_35S	2016
1b	1.1.2	**1.1.2**	1.1.2	418	MH289857.1	Goose	China	HN_20S	2016
1b	1.1.2	**1.1.2**	1.1.2	419	MH289858.1	Chicken	China	HN_50W1	2016
1b	1.1.2	**1.1.2**	1.1.2	422	MH289861.1	Duck	China	HN_67B2	2016
1b	1.1.2	**1.1.2**	1.1.2	423	MH289862.1	Chicken	China	JX_16I2	2016
1b	1.1.2	**1.1.2**	1.1.2	425	MH289864.1	Chicken	China	HN_94N	2016
1b	1.1.2	**1.1.2**	1.1.2	428	MH289867.1	Chicken	China	HN_54L2	2017
1b	1.1.2	**1.1.2**	1.1.2	429	MH289868.1	Chicken	China	JX_28A2	2016
1b	1.1.2	**1.1.2**	1.1.2	430	MH289869.1	Anser_fabalis	China	HN_F2_95_1	2016
1b	1.1.2	**1.1.2**	1.1.2	431	MH289870.1	Chicken	China	HN_6T	2016
1c	1.2	**1.2**	1.2	104	FJ597580	duck	China	ZJ_1	2002
1c	1.2	**1.2**	1.2	105	FJ597581	duck	China	ZJ_3	2002
1c	1.2	**1.2**	1.2	106	FJ597585	duck	China	AH_10	2006
1c	1.2	**1.2**	1.2	107	FJ597586	duck	China	FJ_2	2002
1c	1.2	**1.2**	1.2	108	FJ597587	duck	China	JS_9	2006
1c	1.2	**1.2**	1.2	109	HQ008337	duck	China	JS10	2010
1c	1.2	**1.2**	1.2	110	HQ398777	duck	China	08_037	2008
1c	1.2	**1.2**	1.2	112	HQ398780	duck	China	08_044	2008
1c	1.2	**1.2**	1.2	121	HQ412767	duck	China	08_046	2008
1c	1.2	**1.2**	1.2	122	JF893453	duck	China	JX07	2007
1c	1.2	**1.2**	1.2	123	JX844030.1	mallard	Finland	9147	2010
1c	1.2	**1.2**	1.2	124	AY034801.2	mallard	Finland		1997
1c	1.2	**1.2**	1.2	125	EU493451.2	teal	Finland	12104	2006
1c	1.2	**1.2**	1.2	126	EU493454.2	pochard	Finland	13193	2006
1c	1.2	**1.2**	1.2	127	KC503412.1	duck	Japan	10UO0343	2010
1c	1.2	**1.2**	1.2	128	KC503483.1	Slaty_backed_Gull	Russia	543FFNK15	2007
1c	1.2	**1.2**	1.2	131	KF361507.1	mallard	China	Jilin	2011
1c	1.2	**1.2**	1.2	132	AB871656.1	duck	Japan	Tottori_453	2009
1c	1.2	**1.2**	1.2	133	AB871657.1	duck	Japan	Tottori_481	2009
1c	1.2	**1.2**	1.2	134	KU366517.1	shelduck	Kazakhstan	Chokpak_5717	2013
1c	1.2	**1.2**	1.2	135	DQ097393	duck	Germany	DER49_99	1999
1d	1.2	**1.2**	1.2	136	AY626266	duck	USA	119535_1	2001

1d	1.2	**1.2**	1.2	137	AY626267	duck	USA	154979_1	2001
1d	1.2	**1.2**	1.2	138	AY626268	chicken	USA	101250_2	2001
1d	1.2	**1.2**	1.2	139	EF612277	Northern_Pintail	AK_USA	196	1998
1d	1.2	**1.2**	1.2	140	EF564813	Green_winged_Teal	AK_USA	176	1998
1d	1.2	**1.2**	1.2	143	EF564819	red_knot	DE_USA	2026	2002
1d	1.2	**1.2**	1.2	144	EF564820	mallard	MD_USA	04_125	2004
1d	1.2	**1.2**	1.2	145	EF564822	mallard	MD_USA	04_364	2004
1d	1.2	**1.2**	1.2	146	EF564823	duck	OH_USA	04_612	2004
1d	1.2	**1.2**	1.2	147	EF564825	mallard	MD_USA	02_336	2002
1d	1.2	**1.2**	1.2	148	EF564827	environment	MD_USA	_03_62	2003
1d	1.2	**1.2**	1.2	149	EF564828	mallard	MD_USA	03_808	2003
1d	1.2	**1.2**	1.2	150	EF564829	mallard	MD_USA	01_418	2001
1d	1.2	**1.2**	1.2	151	EF564830	Ruddy_Turnstone	MD_USA	01_523	2001
1d	1.2	**1.2**	1.2	152	JN942004.1		NJ_USA	13890_2	2010
1d	1.2	**1.2**	1.2	153	JN942005.1	muscovy_duck	PA_USA	10145	2010
1d	1.2	**1.2**	1.2	154	JN942006.1		NJ_USA	32796_2	2009
1d	1.2	**1.2**	1.2	155	JN942007.1		PA_USA	33250	2009
1d	1.2	**1.2**	1.2	156	JN942008.1	chicken	FL_USA	31670_4	2009
1d	1.2	**1.2**	1.2	157	JN942009.1	mallard	CT_USA	7847_2	2009
1d	1.2	**1.2**	1.2	158	JN942010.1	Northern_Pintail	MI_USA	28171_16	2009
1d	1.2	**1.2**	1.2	159	JN942011.1	raptor	MN_USA	18575_1	2009
1d	1.2	**1.2**	1.2	160	JN942012.1	mallard	WI_USA	22853	2009
1d	1.2	**1.2**	1.2	161	JN942015.1	mallard	OH_USA	2080_2	2008
1d	1.2	**1.2**	1.2	162	JN941987.1	Northern_Pintail	ID_USA	19663_2	2009
1d	1.2	**1.2**	1.2	163	KC503424.1	Northern_Pintail	AK_USA	44339_630	2007
1d	1.2	**1.2**	1.2	165	KC503426.1	Northern_Pintail	AK_USA	44339_819	2007
1d	1.2	**1.2**	1.2	167	KC503428.1	Northern_Pintail	AK_USA	44339_832	2007
1d	1.2	**1.2**	1.2	168	KC503429.1	Northern_Pintail	AK_USA	44339_835	2007
1d	1.2	**1.2**	1.2	169	KC503430.1	Northern_Pintail	AK_USA	44340_136	2007
1d	1.2	**1.2**	1.2	174	KC503435.1	Great_White_fronted_Goose	AK_USA	44398_192	2008
1d	1.2	**1.2**	1.2	175	KC503436.1	Northern_Pintail	AK_USA	44419_059	2008
1d	1.2	**1.2**	1.2	177	KC503438.1	Northern_Pintail	AK_USA	44420_025	2008
1d	1.2	**1.2**	1.2	178	KC503439.1	Northern_Pintail	AK_USA	44420_048	2008
1d	1.2	**1.2**	1.2	179	KC503440.1	Northern_Pintail	AK_USA	44420_067	2008
1d	1.2	**1.2**	1.2	181	KC503442.1	Northern_Pintail	AK_USA	44420_080	2008
1d	1.2	**1.2**	1.2	182	KC503443.1	Northern_Pintail	AK_USA	44420_088	2008
1d	1.2	**1.2**	1.2	183	KC503444.1	Northern_Pintail	AK_USA	44420_127	2008
1d	1.2	**1.2**	1.2	185	KC503446.1	American_Green_winged_Teal	AK_USA	44493_409	2009
1d	1.2	**1.2**	1.2	186	KC503448.1	American_Green_winged_Teal	AK_USA	44493_503	2009
1d	1.2	**1.2**	1.2	187	KC503449.1	American_Green_winged_Teal	AK_USA	44493_560	2009
1d	1.2	**1.2**	1.2	188	KC503450.1	Northern_Pintail	AK_USA	44493_701	2009

1d	1.2	**1.2**	1.2	190	KC503456.1	American_Green_winged_Teal	AK_USA	44493_736	2009
1d	1.2	**1.2**	1.2	191	KC503457.1	Northern_Pintail	AK_USA	44493_737	2009
1d	1.2	**1.2**	1.2	194	KC503460.1	American_Green_winged_Teal	AK_USA	44493_751	2009
1d	1.2	**1.2**	1.2	195	KC503461.1	Northern_Pintail	AK_USA	44493_762	2009
1d	1.2	**1.2**	1.2	196	KC503462.1	Northern_Pintail	AK_USA	44493_765	2009
1d	1.2	**1.2**	1.2	197	KC503463.1	Northern_Pintail	AK_USA	44493_773	2009
1d	1.2	**1.2**	1.2	198	KC503464.1	Northern_Pintail	AK_USA	44493_779	2009
1d	1.2	**1.2**	1.2	199	KC503468.1	Northern_Pintail	AK_USA	44493_828	2009
1d	1.2	**1.2**	1.2	200	KC503470.1	Northern_Pintail	AK_USA	44493_865	2009
1d	1.2	**1.2**	1.2	201	KC503472.1	Northern_Pintail	AK_USA	44494_659	2009
1d	1.2	**1.2**	1.2	204	KC503475.1	American_Green_winged_Teal	AK_USA	44494_794	2009
1d	1.2	**1.2**	1.2	205	KF444680.1	Mute_Swan	NJ_USA	1180	2012
1d	1.2	**1.2**	1.2	206	KF444681.1	Mute_Swan	MI_USA	780	2012
1d	1.2	**1.2**	1.2	207	KP780877	mallard	ID_USA	ND0001525	2013
1d	1.2	**1.2**	1.2	208	KP780878	American_Green_winged_Teal	AK_USA	ND0007164	2013
1d	1.2	**1.2**	1.2	211	KX857666	Red_Knot	NJ_USA	AI07_1039	2007
1d	1.2	**1.2**	1.2	213	KX857668	mallard	MN_USA	AI07_3115	2007
1d	1.2	**1.2**	1.2	214	KX857669	Herring_Gull	NJ_USA	AI08_1534	2008
1d	1.2	**1.2**	1.2	215	KX857670	mallard	MN_USA	AI08_2152	2008
1d	1.2	**1.2**	1.2	216	KX857671	Ring_billed_Gull	MN_USA	AI08_2203	2008
1d	1.2	**1.2**	1.2	217	KX857672	Blue_winged_Teal	MN_USA	AI08_2490	2008
1d	1.2	**1.2**	1.2	218	KX857673	American_Green_winged_Teal	MN_USA	AI08_4970	2008
1d	1.2	**1.2**	1.2	219	KX857674	mallard	MN_USA	AI08_4984	2008
1d	1.2	**1.2**	1.2	220	KX857675	Herring_Gull	NJ_USA	AI09_209	2009
1d	1.2	**1.2**	1.2	221	KX857676	Ruddy_Turnstone	DE_USA	AI09_619	2009
1d	1.2	**1.2**	1.2	222	KX857677	Franklins_Gull	MN_USA	AI09_1548	2009
1d	1.2	**1.2**	1.2	223	KX857678	mallard	MN_USA	AI09_2529	2009
1d	1.2	**1.2**	1.2	224	KX857679	American_Green_winged_Teal	MN_USA	AI09_3588	2009
1d	1.2	**1.2**	1.2	225	KX857680	Greater_Black_backed_Gull	NJ_USA	AI10_835	2010
1d	1.2	**1.2**	1.2	226	KX857681	Laughing_Gull	NJ_USA	AI10_991	2010
1d	1.2	**1.2**	1.2	227	KX857682	mallard	MN_USA	AI10_3156	2010
1d	1.2	**1.2**	1.2	228	KX857683	Blue_winged_Teal	LA_USA	AI10_3541	2010
1d	1.2	**1.2**	1.2	229	KX857684	American_Green_winged_Teal	MN_USA	AI10_4299	2010
1d	1.2	**1.2**	1.2	230	KX857685	Blue_winged_Teal	TX_USA	AI10_4457	2010
1d	1.2	**1.2**	1.2	231	KX857686	mallard	NJ_USA	AI10_4506	2010
1d	1.2	**1.2**	1.2	232	KX857687	Ruddy_Turnstone	NJ_USA	AI11_982	2011
1d	1.2	**1.2**	1.2	234	KX857689	mallard	MN_USA	AI11_4744	2011
1d	1.2	**1.2**	1.2	235	KX857690	Gadwall	MN_USA	AI12_3182	2012
1d	1.2	**1.2**	1.2	236	KX857691	mallard	MN_USA	AI12_4502	2012
1d	1.2	**1.2**	1.2	237	KX857692	Ruddy_Turnstone	NJ_USA	AI13_2469	2013
1d	1.2	**1.2**	1.2	238	KX857693	mallard	MN_USA	AI13_3303	2013

1d	1.2	**1.2**	1.2	239	KX857694	Blue_winged_Teal	TX_USA	AI13_3994	2013
1d	1.2	**1.2**	1.2	240	KX857695	Blue_winged_Teal	TX_USA	AI13_4042	2013
1d	1.2	**1.2**	1.2	241	KX857696	Blue_winged_Teal	TX_USA	AI14_2572	2014
1d	1.2	**1.2**	1.2	266	KU748779.1	duck	China	Guangxi_1261	2015
UNCL	UNCL9	**UNCL9**	UNCL9	255	EF564815	dunlin	DE_USA	A100_2093	2000
UNCL	UNCL10	**UNCL10**	UNCL10	256	EF564831	Ruddy_Turnstone	DE_USA	401	2004
UNCL	UNCL11	**UNCL11**	UNCL11	257	KC503417.1	Rred_Knot	AK_USA	44308_062	2007
UNCL	UNCL12	**UNCL12**	UNCL12	261	KC503421.1	Black_Brant	AK_USA	44328_651	2007
UNCL	1.1	**1.2**	1.2	262	EF564824	mallard	MD_USA	02_217	2002
UNCL	1.1	**1.1**	1.1	263	AB524405	goose	AK_USA	415	1991
UNCL	1.1	**1.1**	1.1	264	HQ398796	duck	China	09_026	2009
UNCL	1.1	**1.1**	1.1	265	HQ398797	duck	China	09_027	2009
UNCL	UNCL14	**UNCL14**	UNCL14	267	EF564833	Canada_Goose	OH_USA	OH_87_78	1987
UNCL	UNCL_13	**UNCL_13**	UNCL_13	312	KP123431.2	mute_swan	Macedonia	546	2012
