# Supplementary material for: Updated unified phylogenetic classification system and revised nomenclature for Newcastle disease virus
Source: Infect Genet Evol. 2019 Oct;74:103917. doi: 10.1016/j.meegid.2019.103917 (PMC6876278; doi:10.1016/j.meegid.2019.103917)
Supplement: Supplemental Table S2 — Complete fusion gene curated dataset of class II NDV used in this study. The green shading represents congruence between trees. Red font represents lack of branch support at the defining node. The naming in bold font (ML naming) is according to the nomenclature suggested in this study. The dataset contains 1672 sequences and was used to build the trees depicted in Fig. 1B and Supplemental Fig. S5 A–C. “UNCL” = unclassified. [file mmc2.pdf]

Supplemental Table S2. Complete fusion gene curated dataset of class II NDV used in this study. The green shading represents congruence between trees. Red font represents lack of branch support at the defining node. The naming in bold font (ML naming) is according to the nomenclature suggested in this study. The dataset contains 1672 sequences and was used to build the trees depicted in Supplemental Fig. S5 A-C.

red font = lack of branch support
**bold font = naming based on the criteria accepted in this study**
green shading = congruence between trees

**Based on Diel et al. 2012**	**Genotypes based on the new classification and nomenclature system**								
**Genotype**	**NJ**	**ML**	**BAYES**	**ID**	**Acc. Number**	**Host**	**Country**	**Isolate**	**Year**
I_a	I.1.1	**I.1.1**	I.1.1	36	AF217084	chicken	Australia	Queensland_V_4	1966
I_a	I.1.1	**I.1.1**	I.1.1	38	AY935490	chicken	Australia	02_1334	2002
I_a	I.1.1	**I.1.1**	I.1.1	39	AY935491	chicken	Australia	98_1154	1998
I_a	I.1.1	**I.1.1**	I.1.1	40	AY935492	chicken	Australia	98_1249	1998
I_a	I.1.1	**I.1.1**	I.1.1	41	AY935493	chicken	Australia	98_1252	1998
I_a	I.1.1	**I.1.1**	I.1.1	42	AY935494	chicken	Australia	99_0655	1999
I_a	I.1.1	**I.1.1**	I.1.1	43	AY935495	chicken	Australia	99_0868_hi	1999
I_a	I.1.1	**I.1.1**	I.1.1	44	AY935496	chicken	Australia	99_0868_lo	1999
I_a	I.1.1	**I.1.1**	I.1.1	45	AY935497	chicken	Australia	1997PR_32	1999
I_a	I.1.1	**I.1.1**	I.1.1	46	AY935498	chicken	Australia	99_1435	1999
I_a	I.1.1	**I.1.1**	I.1.1	47	GU332645	chicken	Australia	9809_19_1107	1998
I_a	I.1.1	**I.1.1**	I.1.1	54	M24692	chicken	Australia	D26_76	1999
I_a	I.1.1	**I.1.1**	I	55	M24693	chicken	Australia	Queensland	1966
I_a	I.1.1	**I.1.1**	I.1.1	2135	MG869263.1	chicken	Vietnam	NVNvacI2	2016
I_c	I.2	**I.1.2.1**	I.1.2.1	165	EF564816	redknot	NJ_USA	A_101_1383	2001
I_c	I.2	**I.1.2.1**	I.1.2.1	166	GQ918280	black_headed_gull	Sweden		1994
I_c	I.2	**I.1.2.1**	I.1.2.1	167	KC503478	slaty_backedgull	Japan	9KS_0098	2009
I_c	I.2	**I.1.2.1**	I.1.2.1	172	KX352834	gull	Russia	Tyva_14	2014
I_d	I.1.2.2	**I.1.2.2**	I.1.2.2	174	AB465607	chicken	Japan	Ishi	1962
I_d	I.1.2.2	**I.1.2.2**	I.1.2.2	175	EF564817	ruddyturnstone	DE_USA	492	2002
I_d	I.1.2.2	**I.1.2.2**	I.1.2.2	176	KC503476	northern_pintail	AK_USA	44500_136_	2009

I_d	I.1.2.2	**I.1.2.2**	I.1.2.2	177	KC503479	redpoll	Russia	Nikita_530_FFNK2	2008
I_d	I.1.2.2	**I.1.2.2**	I.1.2.2	178	KX857711	American_green_winged_teal	MN_USA	AI09_3831	2009
I_b	I.1.2.1	**I.2**	I.2	71	AB871655	duck	Japan	Tottori_N12	2006
I_b	I.1.2.1	**I.2**	I.2	72	AY965077	Baikal_teal	Russia	FarEast_3658	2002
I_b	I.1.2.1	**I.2**	I.2	73	AY965078	duck	Russia	FarEast_2686	2001
I_b	I.1.2.1	**I.2**	I.2	74	AY965079	duck	Russia	FarEast_2713	2001
I_b	I.1.2.1	**I.2**	I.2	75	AY972101	Baikal_teal	Russia	FarEast_3652	2002
I_b	I.1.2.1	**I.2**	I.2	76	EF564821	mallard	PA_USA	MD_04_204	2004
I_b	I.1.2.1	**I.2**	I.2	77	FJ597603	duck	China	AH_5	2004
I_b	I.1.2.1	**I.2**	I.2	78	FJ597604	duck	China	AH_6	2004
I_b	I.1.2.1	**I.2**	I.2	79	FJ597605	duck	China	AH_13	2004
I_b	I.1.2.1	**I.2**	I.2	80	FJ597606	duck	China	JS_40	2005
I_b	I.1.2.1	**I.2**	I.2	81	FJ597607	duck	China	JS_41	2005
I_b	I.1.2.1	**I.2**	I.2	82	FJ597608	duck	China	JS_42	2005
I_b	I.1.2.1	**I.2**	I.2	83	FJ597609	duck	China	JS_51	2005
I_b	I.1.2.1	**I.2**	I.2	84	FJ597610	duck	China	JS_53	2005
I_b	I.1.2.1	**I.2**	I.2	85	FJ597611	duck	China	JS_14	2005
I_b	I.1.2.1	**I.2**	I.2	86	FJ597612	duck	China	S15	2005
I_b	I.1.2.1	**I.2**	I.2	87	FJ597613	duck	China	ZJ_1	2004
I_b	I.1.2.1	**I.2**	I.2	88	FJ597614	duck	China	ZJ_2	2004
I_b	I.1.2.1	**I.2**	I.2	89	FJ597615	duck	China	ZJ_3	2004
I_b	I.1.2.1	**I.2**	I.2	90	FJ597616	duck	China	ZJ_50	2005
I_b	I.1.2.1	**I.2**	I.2	91	FJ597617	duck	China	HN_34	2005
I_b	I.1.2.1	**I.2**	I.2	92	FJ597618	duck	China	HN_36	2005
I_b	I.1.2.1	**I.2**	I.2	93	FJ597619	duck	China	HN_37	2005
I_b	I.1.2.1	**I.2**	I.2	94	FJ597620	duck	China	JS_39	2005
I_b	I.1.2.1	**I.2**	I.2	95	FJ600539	mule_duck	China	FJ0801	2008
I_b	I.1.2.1	**I.2**	I.2	96	HE972210	duck	Luxembourg	3785	2007
I_b	I.1.2.1	**I.2**	I.2	97	HE972213	mallard	Luxembourg	4178	2008
I_b	I.1.2.1	**I.2**	I.2	98	HG326605	spur_wingedgoose	Nigeria	NIE08_0121	2008
I_b	I.1.2.1	**I.2**	I.2	99	HG326606	wingedgoose	Nigeria	NIE08_0124	2008
I_b	I.1.2.1	**I.2**	I.2	100	HM063422	feralduck	China	D3_Guang_dong	2007
I_b	I.1.2.1	**I.2**	I.2	101	HM125898	duck	China	WDK_Jiangxi_7793	2004
I_b	I.1.2.1	**I.2**	I.2	102	JN653339	Muscovy_duck	China	M4	2007

I_b	I.1.2.1	I.2	I.2	103	JN872169	environment	AK_USA	Pennsylvania_3167	2009
I_b	I.1.2.1	I.2	I.2	104	JQ966077	mallard	South_Korea	WB_KU17_08	2008
I_b	I.1.2.1	I.2	I.2	105	JQ966078	Eurasian_teal	South_Korea	WB_KU22_1	2007
I_b	I.1.2.1	I.2	I.2	106	JQ966082	mallard	South_Korea	WB_KU146	2008
I_b	I.1.2.1	I.2	I.2	107	JQ966083	wild_bird	South_Korea	WB_KU164	2009
I_b	I.1.2.1	I.2	I.2	108	JQ966084	mallard	South_Korea	WB_KU628	2009
I_b	I.1.2.1	I.2	I.2	109	JQ966085	mallard	South_Korea	WB_KU762	2008
I_b	I.1.2.1	I.2	I.2	110	JX193077	duck	China	Guangxi16	2008
I_b	I.1.2.1	I.2	I.2	111	JX193078	duck	China	Guangxi17	2009
I_b	I.1.2.1	I.2	I.2	112	JX193081	duck	China	Guangxi20	2010
I_b	I.1.2.1	I.2	I.2	113	JX193083	duck	China	Guangxi22	2010
I_b	I.1.2.1	I.2	I.2	114	JX401403	mallard	South_Korea	CBU2249	2007
I_b	I.1.2.1	I.2	I.2	115	JX401404	mallard	South_Korea	CBU2179	2007
I_b	I.1.2.1	I.2	I.2	116	JX401405	mallard	South_Korea	CBU2374	2007
I_b	I.1.2.1	I.2	I.2	117	JX518873	Meller_sduck	Madagascar	MGA057C	2009
I_b	I.1.2.1	I.2	I.2	118	JX518874	red_billed_teal	Madagascar	MGA284C	2009
I_b	I.1.2.1	I.2	I.2	119	KC503411	northern_pintail	Japan	10EY0020	2010
I_b	I.1.2.1	I.2	I.2	120	KC503413	duck	Japan	10UO0501	2010
I_b	I.1.2.1	I.2	I.2	121	KC503422	spectacled_eider	AK_USA	AK_44332_723	2007
I_b	I.1.2.1	I.2	I.2	122	KC503447	Amer_green_wingedteal	AK_USA	AK_44493_444	2009
I_b	I.1.2.1	I.2	I.2	123	KC503453	Amer_green_wingedteal	AK_USA	AK_44493_716	2009
I_b	I.1.2.1	I.2	I.2	124	KC503455	northern_pintail	AK_USA	AK_44493_723	2009
I_b	I.1.2.1	I.2	I.2	125	KC503467	northern_pintail	AK_USA	AK_44493_801	2009
I_b	I.1.2.1	I.2	I.2	126	KC503469	northern_pintail	AK_USA	AK_44493_830	2009
I_b	I.1.2.1	I.2	I.2	127	KC503471	american_green_winged_teal	AK_USA	AK_44493_893	2009
I_b	I.1.2.1	I.2	I.2	128	KC503481	coot	Russia	538_FFNK10	2007
I_b	I.1.2.1	I.2	I.2	129	KC503482	slaty_backedgull	Russia	Nikita_539_FFNK11	2007
I_b	I.1.2.1	I.2	I.2	130	KC750151	duck	China	GD_SH	2005
I_b	I.1.2.1	I.2	I.2	131	KC750152	duck	China	GD_SZ	2008
I_b	I.1.2.1	I.2	I.2	132	KC894391	mallard	China	Jilin	2011
I_b	I.1.2.1	I.2	I.2	133	KF851269	teal	Ukraine	Krasnooskilsky_5_11	2009
I_b	I.1.2.1	I.2	I.2	134	KF851270	Ruddy_Shelduck	Ukraine	AN_371502_11	2011
I_b	I.1.2.1	I.2	I.2	135	KJ920203	mallard	Japan	Yakutiya_852	2011
I_b	I.1.2.1	I.2	I.2	136	KM885166	duck	China	LAH_224	2011

I_b	I.1.2.1	**I.2**	I.2	137	KR869090	chicken	China	GX21	2013
I_b	I.1.2.1	**I.2**	I.2	138	KT186351	domestic_duck	South_Korea	DK13	2007
I_b	I.1.2.1	**I.2**	I.2	139	KT380032	chicken	South_Korea	NDRL0901	2010
I_b	I.1.2.1	**I.2**	I.2	140	KT381598	duck	China	Guangdong_GM12	2014
I_b	I.1.2.1	**I.2**	I.2	141	KT381599	duck	China	Guangdong_GZ331	2014
I_b	I.1.2.1	**I.2**	I.2	142	KT892753	swan_goose	China	JL_CC02	2013
I_b	I.1.2.1	**I.2**	I.2	143	KT892754	hooded_crane	China	JL_BC01	2015
I_b	I.1.2.1	**I.2**	I.2	144	KT892755	hooded_crane	China	JL_BC02	2015
I_b	I.1.2.1	**I.2**	I.2	145	KU601398	Common_murre	Russia	Tyuleniy_Island_109	2015
I_b	I.1.2.1	**I.2**	I.2	146	KU662357	Wild_Garganey	Russia	Novosibirsk_27	2014
I_b	I.1.2.1	**I.2**	I.2	147	KX352836	teal	Russia	Novosibirsk_region_320	2010
I_b	I.1.2.1	**I.2**	I.2	148	KX857698	Blue_winged_teal	TX_USA	AI12_3663	2012
I_b	I.1.2.1	**I.2**	I.2	149	KX857699	Mallard	MN_USA	AI12_4078	2012
I_b	I.1.2.1	**I.2**	I.2	150	KX857700	Blue_winged_teal	TX_USA	AI12_4395	2012
I_b	I.1.2.1	**I.2**	I.2	151	KX857701	Blue_winged_teal	LA_USA	AI12_4416	2012
I_b	I.1.2.1	**I.2**	I.2	153	KX857703	Mallard	MN_USA	AI12_4716	2012
I_b	I.1.2.1	**I.2**	I.2	154	KX857704	Mallard	MN_USA	AI12_4867	2012
I_b	I.1.2.1	**I.2**	I.2	155	KX857705	Mallard	MN_USA	AI13_3228	2013
I_b	I.1.2.1	**I.2**	I.2	156	KX857706	Blue_winged_teal	TX_USA	AI13_4119	2013
I_b	I.1.2.1	**I.2**	I.2	157	KX857707	Mallard	MN_USA	AI13_4403	2013
I_b	I.1.2.1	**I.2**	I.2	158	KX857708	Mallard	MN_USA	AI14_3310	2014
I_b	I.1.2.1	**I.2**	I.2	159	KX857709	Mallard	MN_USA	AI14_3452	2014
I_b	I.1.2.1	**I.2**	I.2	160	KX857710	Blue_winged_teal	TX_USA	AI14_3711	2014
I_b	I.1.2.1	**I.2**	I.2	182	AY562991	chicken	Ireland	Ulster	1967
I_b	I.1.2.1	**I.2**	I.2	2064	KX765175.1	duck	China	LH11740	2013
I_b	I.1.2.1	**I.2**	I.2	2065	KX765176.1	duck	China	MH331_7	2011
I_b	I.1.2.1	**I.2**	I.2	2333	MH675922.1	duck	China	D4	2016
I_b	I.1.2.1	**I.2**	I.2	2516	MH289831.1	duck	China	JX_75C2	2016
I_b	I.1.2.1	**I.2**	I.2	2517	MH289832.1	duck	China	JX_2N	2016
I_b	I.1.2.1	**I.2**	I.2	2518	MH289833.1	duck	China	JX_77C2	2016
I_b	I.1.2.1	**I.2**	I.2	2520	MH289835.1	Anser_fabalis	China	HN_F2_110_1	2016
II	II	**II**	II	355	EU289028	turkey	USA	VG_GA	1989
II	II	**II**	II	372	AF077761	chicken	USA	Lasota	1946
II	II	**II**	II	375	GU978777	chicken	USA	TX_GB	1948

II	II	**II**	II	376	JN863121	chicken	USA	New_Jersey_Roakin	1948
II	II	**II**	II	377	JN872151	chicken	USA	Hitchner_B1	1947
II	II	**II**	II	387	KP939087		Mexico	Texcoco_H1_	1950
II	II	**II**	II	388	KP939088	chicken	USA	California_CG_179_H4_	1946
II	II	**II**	II	389	KP939089	chicken	USA	California_11914_FD_H6_	1944
II	II	**II**	II	390	KP939090	chicken	USA	California_119144_NAP_H7_	1944
II	II	**II**	II	391	KP939091	_	USA	Ky_50	1947
II	II	**II**	II	392	KP939092		USA	Ohio_Miller	1948
II	II	**II**	II	393	KP939093		USA	Montana	1946
II	II	**II**	II	394	KP939094		USA	Nebraska_WW1	1955
II	II	**II**	II	395	KP939095	_	USA	Ontario_Berwick	1948
II	II	**II**	II	396	KP939096	_	USA	NJ_KD	1945
II	II	**II**	II	399	KT445901	chicken	Palestine	Komarov	1945
II	II	**II**	II	426	X04719	chicken	USA	Beaudette_C	1945
III	III	**III**	III	428	EF201805	avian		Mukteswar	1940
III	III	**III**	III	432	DQ485259	chicken	china	Guangxi5	2000
III	III	**III**	III	438	GU182327	chicken	Pakistan	SPVC_Karachi_1	1974
III	IX	**III**	III	1668	M24700	_	Australia		1932
III	IX	**III**	III	1669	M24701	chicken	Japan	MIY_51	1951
III	III	**III**	III	1985	MH996904	pigeon	Bulgaria	Novo_Selo_1161	1995
IV	IV	**IV**	IV	444	AY741404	Fowl	UK	Herts	1933
IV	IV	**IV**	IV	445	EU293914	_	Italy	Italien	1944
IV	IV	**IV**	IV	448	MH996898	chicken	Nigeria	Plateau_1973_N53_	1973
IV	IV	**IV**	IV	1969	MH092820	duck	Nigeria	Vom_1980_N55_	1980
IV	IV	**IV**	IV	1977	MH996897	chicken	Nigeria	Kano_N52_899	1973
IV	IV	**IV**	IV	1978	MH996899	chicken	Nigeria	FLD_N54_901	1973
IV	IV	**IV**	IV	1979	MH996900	pullet	Bulgaria	Plovdiv_1153	1959
IV	IV	**IV**	IV	2395	MH996952	chicken	Nigeria	Ibadan_VRD_Ibadan_2	1973
IX	IX	**IX**	IX	1630	AF458009	chicken	China	FJ_1	1985
IX	IX	**IX**	IX	1638	FJ436303	chicken	China	ZJ_1	1986
IX	IX	**IX**	IX	1640	FJ436305	chicken	China	JS_1	1997
IX	IX	**IX**	IX	1661	AY508514	chicken	China	F48E9	1948
IX	IX	**IX**	IX	1663	FJ436302	chicken	China	F48E8	1946_1948
IX	IX	**IX**	IX	1995	MF278924.1	chicken	China	FS_SS_N	1997

UNCL	UNCL1	**UNCL1**	UNCL1	1968	KT987209	chicken	India	TPT_2_AP	2012
UNCL	UNCL2	**UNCL2**	UNCL2	1971	GU187941	chicken	India	Namakkal_Tamil_Nadu	1987
UNCL	UNCL3	**UNCL3**	UNCL3	1972	HQ011508	peacock	India	2K36_Chennai	2008
UNCL	UNCL4	**UNCL4**	UNCL4	1973	KM056353	chicken	India	55_TN_Namakkal	_
VI	UNCL5	**UNCL5**	UNCL5	561	JN872153	chicken	USA	California_1083_Fontana_ancetsral	1971
VI_c	XX	**UNCL6**	UNCL6	680	AB853926	chicken	Japan	Osaka_2440_ancetsral	1969
VI_c	XX	**UNCL7**	UNCL7	681	Z12111	chicken	Great_Britain	Warwick_ancestral	1966
UNCL	UNCL8	**UNCL8**	UNCL8	1963	JX393313	mosquito_pool	Indonesia	JKT1997_ancetsral	1997
V_b	V.2	**V**	V	520	AY562990	Psittacine	USA	Largo	1971
V_b	V.2	**V**	V	521	JN872155	_	USA	Tenn_74_2445_	1974
V_b	V.2	**V**	V	522	JN967789	psittacine	USA	Indiana_29120	1980
V_b	V.2	**V**	V	1982	MH996901	chicken	Bulgaria	ElovDol_1156	1981
V_b	V.2	**V**	V	1983	MH996902	pigeon	Bulgaria	Septemvritsi_1157	1982
V_b	V.2	**V**	V	1984	MH996903	chicken	Bulgaria	Furen_1159	1988
V_b	V.2	**V**	V	526	KF767470	blue_fronted_parrot	Argentina	12567	1976
V_b	V.2	**V**	V	527	KJ123642	chicken	Brazil	SJM_75	1975
V_d	V	**V**	V	551	HG937571	chicken	Uganda	MU013	2011
V_d	V	**V**	V	552	HG937572	chicken	Uganda	MU019	2011
V_d	V	**V**	V	553	HG937573	chicken	Uganda	MU024	2011
V_d	V	**V**	V	554	HG937580	chicken	Uganda	MU040	2011
V_d	V	**V**	V	555	JQ217418	chicken	Kenya	A89	2010
V_d	V	**V**	V	2146	MG988405.1	chicken	Kenya	KE1007	2016
V_b	V.1	**V.1**	V.1	505	AY288999	chicken	Mexico	37821	1996
V_b	V.1	**V.1**	V.1	506	AY562987	gamefowl	USA	CA_211472	2002
V_b	V.1	**V.1**	V.1	507	EF520718	gamefowl	USA_	CA_212676	2002
V_b	V.1	**V.1**	V.1	508	EU518677	chicken	Mexico	Torreon_453	2000
V_b	V.1	**V.1**	V.1	509	EU518680	chicken	Mexico	458	1988
V_b	V.1	**V.1**	V.1	510	EU518681	chicken	Mexico	459	2000
V_b	V.1	**V.1**	V.1	511	JN872189	parrot	USA	Colorado_8278	1982
V_b	V.1	**V.1**	V.1	512	JN872192	chicken	USA	California_211472_4_	2002
V_b	V.1	**V.1**	V.1	513	JN872194	chicken	Honduras	498109_15_	2007
V_b	V.1	**V.1**	V.1	514	JN942028	chicken	Mexico	51735_8_	2000
V_b	V.1	**V.1**	V.1	515	JN942032	Amazon_parrot	USA	Missouri_31378	1996
V_b	V.1	**V.1**	V.1	516	JN942039	Amazon_parrot	USA	California_28936	1988

V_b	V.1	**V.1**	V.1	517	JN942040	parrot	USA	New_Jersey_19674_2	1986
V_b	V.1	**V.1**	V.1	518	JN942042	Amazon_parrot	USA	California_16365	1981
V_b	V.1	**V.1**	V.1	519	JN942045	turkey	Belize	4438_4	2008
V_b	V.1	**V.1**	V.1	528	AY288993	chicken	Honduras	15	2000
V_b	V.1	**V.1**	V.1	529	JN872181	chicken	Honduras	44813	2000
V_b	V.1	**V.1**	V.1	530	JN942027	fighting_cock	Nicaragua	95066_9_	2001
V_c	V.2	**V.2**	V.2	532	EU518682	Dove	Mexico	Distrito_Federal_462	2004
V_c	V.2	**V.2**	V.2	533	EU518683	chicken	Mexico	Estado_de_Mexico_465	2005
V_c	V.2	**V.2**	V.2	534	EU518684	chicken	Mexico	Estado_de_Mexico_466	2006
V_c	V.2	**V.2**	V.2	535	HM117720	chicken	Mexico_Puebla_	NDV_P05	2005
V_c	V.2	**V.2**	V.2	536	JQ697743	chicken	Mexico	MX_NC02_634	2010
V_c	V.2	**V.2**	V.2	537	JQ697744	chicken	Mexico	MX_NC04_635	2010
V_c	V.2	**V.2**	V.2	538	JX974435	chicken	Mexico		2010
V_c	V.2	**V.2**	V.2	539	KC808508	gamefowl	Mexico	616	2008
V_c	V.2	**V.2**	V.2	540	KC808509	gamefowl	Mexico	DF_619	2008
V_c	V.2	**V.2**	V.2	541	KC808510	scarlet_macaw	Mexico	Chiapas_672_ZM12	2009
V_c	V.2	**V.2**	V.2	542	KC808512	quail	Mexico	615	2009
V_c	V.2	**V.2**	V.2	543	KF910963	chicken	Mexico	M08_3313	2008
V_c	V.2	**V.2**	V.2	544	KJ577136	chicken	Mexico	Chimalhuacan	1973
V_c	V.2	**V.2**	V.2	545	KY284094	chicken	Mexico	14_364_14_377	2014
V_c	V.2	**V.2**	V.2	2320	MH392218	chicken	Mexico	NC_23_686	2011
VI_b	VI.1.1	**VI.1**	VI.1	657	AF109885	domestic_fowl	Great_Britain	GB1168	1984
VI_b	VI.1.1	**VI.1**	VI.1	658	AJ880277	pigeon	Italy	IT_227	1982
VI_b	VI.1.1	**VI.1**	VI.1	659	AY734535	pigeon	Argentina	Tigre_6	1999
VI_b	VI.1.1	**VI.1**	VI.1	660	EF520716	pigeon	USA	NY_US	1984
VI_b	VI.1.1	**VI.1**	VI.1	661	FJ410145	pigeon	USA	New_York	1984
VI_b	VI.1.1	**VI.1**	VI.1	662	FJ410147	pigeon	USA	Maryland	1984
VI_b	VI.1.1	**VI.1**	VI.1	663	FJ865434	pigeon	China	S_1	2002
VI_b	VI.1.1	**VI.1**	VI.1	664	JN872173	pigeon	USA	New_York_44407	2007
VI_b	VI.1.1	**VI.1**	VI.1	665	JN967787	pigeon	USA	Illinois_37397	1987
VI_b	VI.1.1	**VI.1**	VI.1	666	JN967788	pigeon	USA	Maryland_11936	1985
VI_b	VI.1.1	**VI.1**	VI.1	667	KU377528	Turtle_Dove	Italy	00VIR4400	2000
VI_b	VI.1.1	**VI.1**	VI.1	668	KU377531	pigeon	Italy	06VIR463_2	2006
VI	VI.2.2	**VI.2.1**	VI.2.1	2188	MH371057.1	pigeon	Israel	PHL137866	2011

VI	VI.2.2	**VI.2.1**	VI.2.1	2222	MH371091.1	pigeon	Israel	PHL133813	2011
VI	VI.2.2	**VI.2.1**	VI.2.1	2235	MH377247.1	pigeon	Israel	PHL37114.2	2008
VI	VI.2.2	**VI.2.1**	VI.2.1	2290	MH377302.1	loughing_dove	Israel	PHL264752	2012
VI	VI.2.2.1	**VI.2.1.1.1**	VI.2.1.1.1	556	AY288995	dove	Italy	2736	2000
VI	VI.2.2.1	**VI.2.1.1.1**	VI.2.1.1.1	558	GQ429292	pigeon	Ireland	AV324	1996
VI	VI.2.2.1	**VI.2.1.1.1**	VI.2.1.1.1	559	GQ429293	Dove	Italy	2736	2000
VI	VI.2.2.1	**VI.2.1.1.1**	VI.2.1.1.1	562	JN872183	pigeon	USA	Maryland_2075	1998
VI	VI.2.2.1	**VI.2.1.1.1**	VI.2.1.1.1	564	KJ736742	pigeon	Germany	R75_98	1998
VI_a	VI.2.2.1	**VI.2.1.1.1**	VI.2.1.1.1	577	EU477189	pigeon	USA_RI_	RI166	2000
VI_a	VI.2.2.1	**VI.2.1.1.1**	VI.2.1.1.1	578	JN872160	pigeon	USA	Minnesota_511296	2007
VI_a	VI.2.2.1	**VI.2.1.1.1**	VI.2.1.1.1	579	JN872167	dove	USA	FL_455682_2_	2006
VI_a	VI.2.2.1	**VI.2.1.1.1**	VI.2.1.1.1	580	JN872172	raptor	USA	Minnesota_18575_4_	2009
VI_a	VI.2.2.1	**VI.2.1.1.1**	VI.2.1.1.1	581	JN872174	environment	USA	New_Jersey_9564_	2010
VI_a	VI.2.2.1	**VI.2.1.1.1**	VI.2.1.1.1	582	JN872175	pigeon	USA	Minnesota_723_	2009
VI_a	VI.2.2.1	**VI.2.1.1.1**	VI.2.1.1.1	583	JN872176	pheasant	USA	Massachusetts_359425_	2005
VI_a	VI.2.2.1	**VI.2.1.1.1**	VI.2.1.1.1	584	JN872177	chicken	USA	Massachusetts_344783_3_	2004
VI_a	VI.2.2.1	**VI.2.1.1.1**	VI.2.1.1.1	585	JN872190	pigeon	USA	South_Dakota_486839	2007
VI_a	VI.2.2.1	**VI.2.1.1.1**	VI.2.1.1.1	586	JN941996	pigeon	USA	Pennsylvania_2062_	2008
VI_a	VI.2.2.1	**VI.2.1.1.1**	VI.2.1.1.1	587	JN941998	pigeon	USA	Pennsylvania_2282	2008
VI_a	VI.2.2.1	**VI.2.1.1.1**	VI.2.1.1.1	588	JN941999	pigeon	USA	Minnesota_77_	2008
VI_a	VI.2.2.1	**VI.2.1.1.1**	VI.2.1.1.1	589	JN942000	dove	USA	Florida_1287_	2008
VI_a	VI.2.2.1	**VI.2.1.1.1**	VI.2.1.1.1	590	JN942100	chukar	USA	New_Jersey_32429_5	2000
VI_a	VI.2.2.1	**VI.2.1.1.1**	VI.2.1.1.1	591	JX901304	_	USA	301	2003
VI_a	VI.2.2.1	**VI.2.1.1.1**	VI.2.1.1.1	592	JX901305	_	USA	302	2003
VI_a	VI.2.2.1	**VI.2.1.1.1**	VI.2.1.1.1	593	JX901313	pigeon	USA	102	2001
VI_a	VI.2.2.1	**VI.2.1.1.1**	VI.2.1.1.1	594	JX901314	dove	USA	103	2001
VI_a	VI.2.2.1	**VI.2.1.1.1**	VI.2.1.1.1	595	JX901317	pigeon	USA	201	2002
VI_a	VI.2.2.1	**VI.2.1.1.1**	VI.2.1.1.1	596	JX901319	pigeon	USA	PA_0501	2005
VI_a	VI.2.2.1	**VI.2.1.1.1**	VI.2.1.1.1	597	JX901320	pigeon	USA	PA_0502	2005
VI_a	VI.2.2.1	**VI.2.1.1.1**	VI.2.1.1.1	598	JX901321	chicken	USA	PA_0503	2005
VI_a	VI.2.2.1	**VI.2.1.1.1**	VI.2.1.1.1	599	JX901323	pigeon	USA	PA_0601	2006
VI_a	VI.2.2.1	**VI.2.1.1.1**	VI.2.1.1.1	600	JX901324	pigeon	USA	PA_0602	2006
VI_a	VI.2.2.1	**VI.2.1.1.1**	VI.2.1.1.1	601	JX901325	pigeon	USA	NY_0603	2006
VI_a	VI.2.2.1	**VI.2.1.1.1**	VI.2.1.1.1	602	JX901326	pigeon	USA	NJ_0604	2006

VI_a	VI.2.2.1	**VI.2.1.1.1**	VI.2.1.1.1	603	JX901327	pigeon	USA	PA_0605	2006
VI_a	VI.2.2.1	**VI.2.1.1.1**	VI.2.1.1.1	604	JX901328	pigeon	USA	NJ_0607	2006
VI_a	VI.2.2.1	**VI.2.1.1.1**	VI.2.1.1.1	605	JX901329	pigeon	USA	PA_0701	2007
VI_a	VI.2.2.1	**VI.2.1.1.1**	VI.2.1.1.1	606	JX901330	pigeon	USA	PA_0702	2007
VI_a	VI.2.2.1	**VI.2.1.1.1**	VI.2.1.1.1	607	JX901332	pigeon	USA	MO_0703	2007
VI_a	VI.2.2.1	**VI.2.1.1.1**	VI.2.1.1.1	608	JX901333	pigeon	USA	PA_0704	2007
VI_a	VI.2.2.1	**VI.2.1.1.1**	VI.2.1.1.1	609	JX901334	pigeon	USA	PA_0705	2007
VI_a	VI.2.2.1	**VI.2.1.1.1**	VI.2.1.1.1	610	JX901335	pigeon	USA	PA_0706	2007
VI_a	VI.2.2.1	**VI.2.1.1.1**	VI.2.1.1.1	611	JX901336	pigeon	USA	NC_0707	2007
VI_a	VI.2.2.1	**VI.2.1.1.1**	VI.2.1.1.1	612	JX901337	pigeon	USA	CT_0708	2007
VI_a	VI.2.2.1	**VI.2.1.1.1**	VI.2.1.1.1	613	JX901338	pigeon	USA	OH_0709	2007
VI_a	VI.2.2.1	**VI.2.1.1.1**	VI.2.1.1.1	614	JX901339	pigeon	USA	NJ_0710	2007
VI_a	VI.2.2.1	**VI.2.1.1.1**	VI.2.1.1.1	615	JX901340	pigeon	USA	PA_0711	2007
VI_a	VI.2.2.1	**VI.2.1.1.1**	VI.2.1.1.1	616	JX901344	pigeon	USA	ME_0714	2007
VI_a	VI.2.2.1	**VI.2.1.1.1**	VI.2.1.1.1	617	JX901346	pigeon	USA	PA_0716	2007
VI_a	VI.2.2.1	**VI.2.1.1.1**	VI.2.1.1.1	618	JX901347	pigeon	USA	NY_0717	2007
VI_a	VI.2.2.1	**VI.2.1.1.1**	VI.2.1.1.1	619	JX901348	pigeon	USA	PA_0718	2007
VI_a	VI.2.2.1	**VI.2.1.1.1**	VI.2.1.1.1	620	JX901349	pigeon	USA	MD_0719	2007
VI_a	VI.2.2.1	**VI.2.1.1.1**	VI.2.1.1.1	621	JX901350	pigeon	USA	PA_0720	2007
VI_a	VI.2.2.1	**VI.2.1.1.1**	VI.2.1.1.1	622	JX901351	pigeon	USA	NJ_0721	2007
VI_a	VI.2.2.1	**VI.2.1.1.1**	VI.2.1.1.1	623	JX901352	pigeon	USA	PA_0723	2007
VI_a	VI.2.2.1	**VI.2.1.1.1**	VI.2.1.1.1	624	JX901353	pigeon	USA	PA_0724	2007
VI_a	VI.2.2.1	**VI.2.1.1.1**	VI.2.1.1.1	625	JX901354	pigeon	USA	PA_0725	2007
VI_a	VI.2.2.1	**VI.2.1.1.1**	VI.2.1.1.1	626	JX901355	pigeon	USA	PA_0726	2007
VI_a	VI.2.2.1	**VI.2.1.1.1**	VI.2.1.1.1	627	JX901356	pigeon	USA	PA_0727	2007
VI_a	VI.2.2.1	**VI.2.1.1.1**	VI.2.1.1.1	628	JX901357	pigeon	USA	PA_0728	2007
VI_a	VI.2.2.1	**VI.2.1.1.1**	VI.2.1.1.1	629	JX901358	pigeon	USA	PA_0801	2008
VI_a	VI.2.2.1	**VI.2.1.1.1**	VI.2.1.1.1	630	JX901359	pigeon	USA	PA_0802	2008
VI_a	VI.2.2.1	**VI.2.1.1.1**	VI.2.1.1.1	631	JX901360	pigeon	USA	PA_0803	2008
VI_a	VI.2.2.1	**VI.2.1.1.1**	VI.2.1.1.1	632	JX901361	pigeon	USA	PA_0804	2008
VI_a	VI.2.2.1	**VI.2.1.1.1**	VI.2.1.1.1	633	JX901362	pigeon	USA	PA_0805	2008
VI_a	VI.2.2.1	**VI.2.1.1.1**	VI.2.1.1.1	634	JX901363	pigeon	USA	PA_0806	2008
VI_a	VI.2.2.1	**VI.2.1.1.1**	VI.2.1.1.1	635	JX901364	pigeon	USA	PA_0807	2008
VI_a	VI.2.2.1	**VI.2.1.1.1**	VI.2.1.1.1	636	JX901365	pigeon	USA	PA_0808	2008

VI_a	VI.2.2.1	**VI.2.1.1.1**	VI.2.1.1.1	637	JX901366	pigeon	USA	PA_0809	2008
VI_a	VI.2.2.1	**VI.2.1.1.1**	VI.2.1.1.1	638	JX901367	pigeon	USA	PA_0810	2008
VI_a	VI.2.2.1	**VI.2.1.1.1**	VI.2.1.1.1	639	JX901368	pigeon	USA	PA_0811	2008
VI_a	VI.2.2.1	**VI.2.1.1.1**	VI.2.1.1.1	640	JX901369	pigeon	USA	NJ_0812	2008
VI_a	VI.2.2.1	**VI.2.1.1.1**	VI.2.1.1.1	641	JX901373	pigeon	USA	PA_0902	2009
VI_a	VI.2.2.1	**VI.2.1.1.1**	VI.2.1.1.1	642	JX901374	pigeon	USA	OH_0903	2009
VI_a	VI.2.2.1	**VI.2.1.1.1**	VI.2.1.1.1	643	JX901375	pigeon	USA	PA_0904	2009
VI_a	VI.2.2.1	**VI.2.1.1.1**	VI.2.1.1.1	644	JX901376	pigeon	USA	PA_0905	2009
VI_a	VI.2.2.1	**VI.2.1.1.1**	VI.2.1.1.1	645	JX901377	pigeon	USA	PA_0906	2009
VI_a	VI.2.2.1	**VI.2.1.1.1**	VI.2.1.1.1	646	KP780870	Rock_pigeon	USA	MD_ND0002270	2013
VI_a	VI.2.2.1	**VI.2.1.1.1**	VI.2.1.1.1	647	KP780871	Rock_pigeon	USA	Allegheny_PA_ND0007190	2013
VI_a	VI.2.2.1	**VI.2.1.1.1**	VI.2.1.1.1	648	KP780872	Rock_pigeon	USA	Allegheny_PA_ND0007186	2013
VI_a	VI.2.2.1	**VI.2.1.1.1**	VI.2.1.1.1	649	KP780873	Rock_pigeon	USA	Allegheny_PA_ND0007187	2013
VI_a	VI.2.2.1	**VI.2.1.1.1**	VI.2.1.1.1	650	KP780874	Rock_pigeon	USA	Allegheny_PA_ND0007199	2013
VI_a	VI.2.2.1	**VI.2.1.1.1**	VI.2.1.1.1	651	KP780875	Rock_pigeon	USA	Ingham_MI_ND0003553	2013
VI_a	VI.2.2.1	**VI.2.1.1.1**	VI.2.1.1.1	652	KP780876	Rock_pigeon	USA	Ingham_MI_ND0003558	2013
VI_a	VI.2.2.1	**VI.2.1.1.1**	VI.2.1.1.1	653	KU059752	Eurasian_collared_dove	USA_TX_	TX3988	2004
VI_a	VI.2.2.1	**VI.2.1.1.1**	VI.2.1.1.1	654	MG018201	RODO	USA_MA	1188_kidney_24998_1G	2014
VI_a	VI.2.2.1	**VI.2.1.1.1**	VI.2.1.1.1	655	MG018202	ECDO	USA_MT	1177_kidney_22919_1_B	2010
VI_a	VI.2.2.1	**VI.2.1.1.1**	VI.2.1.1.1	656	MG018218	RODO	USA_PA	1189_kidney_23844_3_C	2012
VI_n	VI.2.2.1	**VI.2.1.1.1**	VI.2.1.1.1	892	EU477188	Dove	USA_Texas_	TX_B_2580	2004
VI_n	VI.2.2.1	**VI.2.1.1.1**	VI.2.1.1.1	893	EU477190	pigeon	USA_Texas_	TX3503	2004
VI_n	VI.2.2.1	**VI.2.1.1.1**	VI.2.1.1.1	894	EU477191	Eurasian_collared_dove	USA_Texas_	TX3988	2005
VI_n	VI.2.2.1	**VI.2.1.1.1**	VI.2.1.1.1	895	EU477192	Eurasian_collared_dove	USA_Texas_	TX4156	2005
VI_n	VI.2.2.1	**VI.2.1.1.1**	VI.2.1.1.1	896	EU477195	Eurasian_collared_dove	USA	TX6295	2006
VI_n	VI.2.2.1	**VI.2.1.1.1**	VI.2.1.1.1	897	JN872170	pigeon	USA	Texas_5254_12	2010
VI_n	VI.2.2.1	**VI.2.1.1.1**	VI.2.1.1.1	898	JN872178	turkey	USA	Louisiana_331309_	2004
VI_n	VI.2.2.1	**VI.2.1.1.1**	VI.2.1.1.1	899	JN872179	pigeon	USA	Nevada_241851_	2003
VI_n	VI.2.2.1	**VI.2.1.1.1**	VI.2.1.1.1	900	JX901342	pigeon	USA	MN_0713	2007
VI_n	VI.2.2.1	**VI.2.1.1.1**	VI.2.1.1.1	901	MG018205	ECDO	USA_TX	1179_kidney_26041_3_C	2014
VI_n	VI.2.2.1	**VI.2.1.1.1**	VI.2.1.1.1	902	MG018206	ECDO	USA_TX	1180_kidney_26041_4_C	2014
VI_n	VI.2.2.1	**VI.2.1.1.1**	VI.2.1.1.1	903	MG018208	ECDO	USA_UT	1181_spleen_26594_1_A	2015
VI_n	VI.2.2.1	**VI.2.1.1.1**	VI.2.1.1.1	904	MG018209	ECDO	USA_TX	1182_spleen_26877_2_C	2016
VI_n	VI.2.2.1	**VI.2.1.1.1**	VI.2.1.1.1	905	MG018210	ECDO	USA_TX	1184_kidney_26981_2_C	2015

VI_n	VI.2.2.1	**VI.2.1.1.1**	VI.2.1.1.1	906	MG018211	ECDO	USA_TX	1185_kidney_26981_3_A	2015
VI_n	VI.2.2.1	**VI.2.1.1.1**	VI.2.1.1.1	907	MG018213	ECDO	USA_KS	1191_spleen_W16_453_3	2016
VI_j	VI.2.2.2.1	**VI.2.1.1.2.1**	VI.2.1.1.2.1	563	KJ600777	pigeon	China	SD_132	2012
VI_j	VI.2.2.2.1	**VI.2.1.1.2.1**	VI.2.1.1.2.1	786	AY288996	pigeon	Italy	1166	2000
VI_j	VI.2.2.2.1	**VI.2.1.1.2.1**	VI.2.1.1.2.1	787	FJ766527	pigeon	China	JS_16	2007
VI_j	VI.2.2.2.1	**VI.2.1.1.2.1**	VI.2.1.1.2.1	788	GQ281085	pigeon	China	JS_35	2007
VI_j	VI.2.2.2.1	**VI.2.1.1.2.1**	VI.2.1.1.2.1	789	GQ281086	pigeon	China	WX_10	2007
VI_j	VI.2.2.2.1	**VI.2.1.1.2.1**	VI.2.1.1.2.1	790	GQ281087	pigeon	China	YZ_21	2007
VI_j	VI.2.2.2.1	**VI.2.1.1.2.1**	VI.2.1.1.2.1	791	GQ281088	pigeon	China	YZ_23	2007
VI_j	VI.2.2.2.1	**VI.2.1.1.2.1**	VI.2.1.1.2.1	792	GU551934	carrier_pigeon	China	carrier_Guandong	2008
VI_j	VI.2.2.2.1	**VI.2.1.1.2.1**	VI.2.1.1.2.1	793	HE972209	pigeon	Luxemburg	119	2006
VI_j	VI.2.2.2.1	**VI.2.1.1.2.1**	VI.2.1.1.2.1	794	HE972212	pigeon	Luxemburg	3821_1	2007
VI_j	VI.2.2.2.1	**VI.2.1.1.2.1**	VI.2.1.1.2.1	795	HM063423	whitebreasted_water_hen_	China_Guangdong_	W4	2005
VI_j	VI.2.2.2.1	**VI.2.1.1.2.1**	VI.2.1.1.2.1	796	HM063425	wild_pigeon	China_Guangdong_	P4	2003
VI_j	VI.2.2.2.1	**VI.2.1.1.2.1**	VI.2.1.1.2.1	797	HM625835	duck	China	SS	2008
VI_j	VI.2.2.2.1	**VI.2.1.1.2.1**	VI.2.1.1.2.1	798	HM748948	chicken	China	SD5	2008
VI_j	VI.2.2.2.1	**VI.2.1.1.2.1**	VI.2.1.1.2.1	799	JF713701	pigeon	China	SD10	2010
VI_j	VI.2.2.2.1	**VI.2.1.1.2.1**	VI.2.1.1.2.1	800	JN941995	pigeon	USA	New_York_1989_19_	2008
VI_j	VI.2.2.2.1	**VI.2.1.1.2.1**	VI.2.1.1.2.1	802	JN986839	pigeon	Ireland	806	2004
VI_j	VI.2.2.2.1	**VI.2.1.1.2.1**	VI.2.1.1.2.1	803	JQ268609	pigeon	China	Miangyang12505	2011
VI_j	VI.2.2.2.1	**VI.2.1.1.2.1**	VI.2.1.1.2.1	804	JX094510	pigeon	China	sms12	2012
VI_j	VI.2.2.2.1	**VI.2.1.1.2.1**	VI.2.1.1.2.1	805	JX244789	pigeon	China	JM_11	2000
VI_j	VI.2.2.2.1	**VI.2.1.1.2.1**	VI.2.1.1.2.1	806	JX244798	pigeon	China	104	2008
VI_j	VI.2.2.2.1	**VI.2.1.1.2.1**	VI.2.1.1.2.1	807	JX244799	pigeon	China	105	2008
VI_j	VI.2.2.2.1	**VI.2.1.1.2.1**	VI.2.1.1.2.1	808	JX244800	pigeon	China	106	2008
VI_j	VI.2.2.2.1	**VI.2.1.1.2.1**	VI.2.1.1.2.1	809	JX486550	pigeon	China	LGD_110208	2011
VI_j	VI.2.2.2.1	**VI.2.1.1.2.1**	VI.2.1.1.2.1	810	JX486551	pigeon	China	LJL_100605	2010
VI_j	VI.2.2.2.1	**VI.2.1.1.2.1**	VI.2.1.1.2.1	811	JX486553	pigeon	China	LHLJ_110813	2011
VI_j	VI.2.2.2.1	**VI.2.1.1.2.1**	VI.2.1.1.2.1	812	JX486554	pigeon	China	LHLJ_110822	2011
VI_j	VI.2.2.2.1	**VI.2.1.1.2.1**	VI.2.1.1.2.1	813	JX486555	pigeon	China	LGD_110945	2011
VI_j	VI.2.2.2.1	**VI.2.1.1.2.1**	VI.2.1.1.2.1	814	JX486557	pigeon	China	LJL_120404	2012
VI_j	VI.2.2.2.1	**VI.2.1.1.2.1**	VI.2.1.1.2.1	815	JX901109	pigeon	Belgium	238	1998
VI_j	VI.2.2.2.1	**VI.2.1.1.2.1**	VI.2.1.1.2.1	816	JX901110	pigeon	Belgium	248	1998
VI_j	VI.2.2.2.1	**VI.2.1.1.2.1**	VI.2.1.1.2.1	817	JX901111	pigeon	Belgium	321	1998

VI_j	VI.2.2.2.1	**VI.2.1.1.2.1**	VI.2.1.1.2.1	818	JX901119	pigeon	Belgium	1824	2005
VI_j	VI.2.2.2.1	**VI.2.1.1.2.1**	VI.2.1.1.2.1	819	JX901121	pigeon	Belgium	4943	2007
VI_j	VI.2.2.2.1	**VI.2.1.1.2.1**	VI.2.1.1.2.1	820	KJ525672	pigeon	China	SD_JN	2010
VI_j	VI.2.2.2.1	**VI.2.1.1.2.1**	VI.2.1.1.2.1	821	KM374059	pigeon	China	Guangxi_1015	2013
VI_j	VI.2.2.2.1	**VI.2.1.1.2.1**	VI.2.1.1.2.1	822	KT381595	pigeon	China	Guangdong_GM1	2014
VI_j	VI.2.2.2.1	**VI.2.1.1.2.1**	VI.2.1.1.2.1	823	KT381606	pigeon	China	Guangdong_GZ289	2014
VI_j	VI.2.2.2.1	**VI.2.1.1.2.1**	VI.2.1.1.2.1	824	KU377529	Turtle_Dove	Italy	02VIR1875	2002
VI_j	VI.2.2.2.1	**VI.2.1.1.2.1**	VI.2.1.1.2.1	825	KU377530	Turtle_Dove	Italy	04VIR434	2004
VI_j	VI.2.2.2.1	**VI.2.1.1.2.1**	VI.2.1.1.2.1	826	KU377532	Turtle_Dove	Italy	08VIR24	2008
VI_j	VI.2.2.2.1	**VI.2.1.1.2.1**	VI.2.1.1.2.1	827	KU377534	pigeon	Italy	11VIR5795	2011
VI_j	VI.2.2.2.1	**VI.2.1.1.2.1**	VI.2.1.1.2.1	828	KU377537	pigeon	Italy	14VIR8258_2	2014
VI_j	VI.2.2.2.1	**VI.2.1.1.2.1**	VI.2.1.1.2.1	829	KX247376	pigeon	China	SX_01	2015
VI_j	VI.2.2.2.1	**VI.2.1.1.2.1**	VI.2.1.1.2.1	830	KX761865	pigeon	China	HB1103	2011
VI_j	VI.2.2.2.1	**VI.2.1.1.2.1**	VI.2.1.1.2.1	2056	MF681716.1	dove	Namibia	8636	2016
VI_j	VI.2.2.2.1	**VI.2.1.1.2.1**	VI.2.1.1.2.1	2057	MF681715.1	dove	Namibia	8199	2016
VI_j	VI.2.2.2.1	**VI.2.1.1.2.1**	VI.2.1.1.2.1	2059	MF681713.1	dove	Namibia	7808	2016
VI_j	VI.2.2.2.1	**VI.2.1.1.2.1**	VI.2.1.1.2.1	2060	MF681712.1	dove	Namibia	5072	2016
VI_j	VI.2.2.2.1	**VI.2.1.1.2.1**	VI.2.1.1.2.1	2061	MF681711.1	dove	Namibia	4533	2017
VI_k	VI.2.2.2.2	**VI.2.1.1.2.2**	VI.2.1.1.2.2	834	JN872162	rosella	Belgium	4940	2008
VI_k	VI.2.2.2.2	**VI.2.1.1.2.2**	VI.2.1.1.2.2	835	JQ979176	pigeon	China	SDLC	2011
VI_k	VI.2.2.2.2	**VI.2.1.1.2.2**	VI.2.1.1.2.2	836	JQ993431	pigeon	China	SDS	2011
VI_k	VI.2.2.2.2	**VI.2.1.1.2.2**	VI.2.1.1.2.2	837	JX486552	pigeon	China	LLN_110713	2011
VI_k	VI.2.2.2.2	**VI.2.1.1.2.2**	VI.2.1.1.2.2	838	JX901120	pigeon	Belgium	03936_8_	2005
VI_k	VI.2.2.2.2	**VI.2.1.1.2.2**	VI.2.1.1.2.2	839	JX901122	pigeon	Belgium	11_07574	2011
VI_k	VI.2.2.2.2	**VI.2.1.1.2.2**	VI.2.1.1.2.2	840	JX901123	pigeon	Belgium	11_08304	2011
VI_k	VI.2.2.2.2	**VI.2.1.1.2.2**	VI.2.1.1.2.2	841	JX901124	pigeon	Belgium	11_09620	2011
VI_k	VI.2.2.2.2	**VI.2.1.1.2.2**	VI.2.1.1.2.2	842	KF828884.1	pigeon	China	_BJP13	2013
VI_k	VI.2.2.2.2	**VI.2.1.1.2.2**	VI.2.1.1.2.2	843	KJ607165	pigeon	China	LHLJ_131237	2013
VI_k	VI.2.2.2.2	**VI.2.1.1.2.2**	VI.2.1.1.2.2	844	KJ607166	pigeon	China	LHLJ_131011	2013
VI_k	VI.2.2.2.2	**VI.2.1.1.2.2**	VI.2.1.1.2.2	845	KJ782376	pigeon	China	NM_0707	2011
VI_k	VI.2.2.2.2	**VI.2.1.1.2.2**	VI.2.1.1.2.2	846	KJ808819	pigeon	China	BJ2013	2013
VI_k	VI.2.2.2.2	**VI.2.1.1.2.2**	VI.2.1.1.2.2	847	KM374056	pigeon	China	Yunnan_1111	2013
VI_k	VI.2.2.2.2	**VI.2.1.1.2.2**	VI.2.1.1.2.2	848	KM374057	pigeon	China	Anhui_2365	2012
VI_k	VI.2.2.2.2	**VI.2.1.1.2.2**	VI.2.1.1.2.2	849	KM374058	pigeon	China	Anhui_2369	2012

VI_k	VI.2.2.2.2	**VI.2.1.1.2.2**	VI.2.1.1.2.2	850	KM374060	pigeon	China	Shanghai_215	2011
VI_k	VI.2.2.2.2	**VI.2.1.1.2.2**	VI.2.1.1.2.2	851	KM374061	pigeon	China	Zhejiang_2036	2012
VI_k	VI.2.2.2.2	**VI.2.1.1.2.2**	VI.2.1.1.2.2	852	KP861633	pigeon	China	SD069	2012
VI_k	VI.2.2.2.2	**VI.2.1.1.2.2**	VI.2.1.1.2.2	853	KT163262	pigeon	China	SH_0167	2013
VI_k	VI.2.2.2.2	**VI.2.1.1.2.2**	VI.2.1.1.2.2	854	KT381592	pigeon	China	Guangdong_GZ293	2014
VI_k	VI.2.2.2.2	**VI.2.1.1.2.2**	VI.2.1.1.2.2	855	KT381601	pigeon	China	Guangdong_GZ287	2013
VI_k	VI.2.2.2.2	**VI.2.1.1.2.2**	VI.2.1.1.2.2	856	KT381602	pigeon	China	Guangdong_GZ288	2013
VI_k	VI.2.2.2.2	**VI.2.1.1.2.2**	VI.2.1.1.2.2	857	KT381603	pigeon	China	Guangdong_GZ290	2013
VI_k	VI.2.2.2.2	**VI.2.1.1.2.2**	VI.2.1.1.2.2	858	KT381604	pigeon	China	Guangdong_GZ292	2014
VI_k	VI.2.2.2.2	**VI.2.1.1.2.2**	VI.2.1.1.2.2	859	KU522142	pigeon	Egypt	VRLCU	2014
VI_k	VI.2.2.2.2	**VI.2.1.1.2.2**	VI.2.1.1.2.2	860	KU527559	pigeon	China	Jilin_DH09	2015
VI_k	VI.2.2.2.2	**VI.2.1.1.2.2**	VI.2.1.1.2.2	861	KU527560	pigeon	China	Jilin_NG05	2015
VI_k	VI.2.2.2.2	**VI.2.1.1.2.2**	VI.2.1.1.2.2	862	KX710210.1	pigeon	India	D168	2015
VI_k	VI.2.2.2.2	**VI.2.1.1.2.2**	VI.2.1.1.2.2	863	KY788663.1.	Grey_heron	China	GD_GZ333	2015
VI_k	VI.2.2.2.2	**VI.2.1.1.2.2**	VI.2.1.1.2.2	864	KY788667.1.	European_turtle_dove	China	GD_GZ23	2015
VI_k	VI.2.2.2.2	**VI.2.1.1.2.2**	VI.2.1.1.2.2	2085	MF580815.1	pigeon	China	GX0094	2011
VI_k	VI.2.2.2.2	**VI.2.1.1.2.2**	VI.2.1.1.2.2	2086	MF580816.1	pigeon	China	GX0001	2012
VI_k	VI.2.2.2.2	**VI.2.1.1.2.2**	VI.2.1.1.2.2	2087	MF580817.1	pigeon	China	GX0012	2012
VI_k	VI.2.2.2.2	**VI.2.1.1.2.2**	VI.2.1.1.2.2	2088	MF580818.1	pigeon	China	GX0019	2012
VI_k	VI.2.2.2.2	**VI.2.1.1.2.2**	VI.2.1.1.2.2	2089	MF580819.1	pigeon	China	GX0022	2012
VI_k	VI.2.2.2.2	**VI.2.1.1.2.2**	VI.2.1.1.2.2	2090	MF580820.1	pigeon	China	GX0029	2012
VI_k	VI.2.2.2.2	**VI.2.1.1.2.2**	VI.2.1.1.2.2	2092	MF580822.1	pigeon	China	GX0034	2012
VI_k	VI.2.2.2.2	**VI.2.1.1.2.2**	VI.2.1.1.2.2	2093	MF580823.1	pigeon	China	GX0505	2012
VI_k	VI.2.2.2.2	**VI.2.1.1.2.2**	VI.2.1.1.2.2	2094	MF580824.1	pigeon	China	GX0004	2013
VI_k	VI.2.2.2.2	**VI.2.1.1.2.2**	VI.2.1.1.2.2	2095	MF580825.1	pigeon	China	GX0119	2015
VI_k	VI.2.2.2.2	**VI.2.1.1.2.2**	VI.2.1.1.2.2	2096	MF580826.1	pigeon	China	GX0126	2015
VI_k	VI.2.2.2.2	**VI.2.1.1.2.2**	VI.2.1.1.2.2	2097	MF580827.1	pigeon	China	GX1103	2015
VI_k	VI.2.2.2.2	**VI.2.1.1.2.2**	VI.2.1.1.2.2	2472	MG840651.1	environment	China	Hubei_HD240	2017
VI_k	VI.2.2.2.2	**VI.2.1.1.2.2**	VI.2.1.1.2.2	2473	MG840652.1	pigeon	China	Fujian_1233	2014
VI_k	VI.2.2.2.2	**VI.2.1.1.2.2**	VI.2.1.1.2.2	2474	MG840653.1	pigeon	China	Guizhou_1050	2017
VI_k	VI.2.2.2.2	**VI.2.1.1.2.2**	VI.2.1.1.2.2	2475	MG840654.1	pigeon	China	Ningxia_2068	2016
VI_k	VI.2.2.2.2	**VI.2.1.1.2.2**	VI.2.1.1.2.2	2476	MG840655.1	pigeon	China	Qinghai_1325	2017
VI_k	VI.2.2.2.2	**VI.2.1.1.2.2**	VI.2.1.1.2.2	2477	MG840656.1	pigeon	China	Qinghai_1344	2017
VI_k	VI.2.2.2.2	**VI.2.1.1.2.2**	VI.2.1.1.2.2	2478	MG840657.1	pigeon	China	Sichuan_2045	2014

VI_k	VI.2.2.2.2	**VI.2.1.1.2.2**	VI.2.1.1.2.2	2479	MG840658.1	pigeon	China	Yunnan_1205	2015
VI_k	VI.2.2.2.2	**VI.2.1.1.2.2**	VI.2.1.1.2.2	2480	MG840659.1	pigeon	China	Yunnan_1336	2015
VI_k	VI.2.2.2.2	**VI.2.1.1.2.2**	VI.2.1.1.2.2	2481	MG840660.1	pigeon	China	Yunnan_1453	2017
VI_k	VI.2.2.2.2	**VI.2.1.1.2.2**	VI.2.1.1.2.2	2488	MG878084.1	pigeon	China	LN	2017
VI_h	VI.2.1	**VI.2.1.2**	VI.2.1.2	750	HG326601	pigeon	Nigeria	NIE07_061	2007
VI_h	VI.2.1	**VI.2.1.2**	VI.2.1.2	751	HG326602	pigeon	Nigeria	NIE07_062	2007
VI_h	VI.2.1	**VI.2.1.2**	VI.2.1.2	752	HG326603	pigeon	Nigeria	NIE07_063	2007
VI_h	VI.2.1	**VI.2.1.2**	VI.2.1.2	753	HG326604	pigeon	Nigeria	NIE09_1898	2009
VI_h	VI.2.1	**VI.2.1.2**	VI.2.1.2	755	JX518532	laughing_dove	Kenya	B2_Isiolo	2012
VI_h	VI.2.1	**VI.2.1.2**	VI.2.1.2	757	HG424627	pigeon	Nigeria	NIE13_092	2013
VI_h	VI.2.1	**VI.2.1.2**	VI.2.1.2	758	JQ039387	pigeon	Nigeria	VRD08_37BRpe_7_9_	2008
VI_h	VI.2.1	**VI.2.1.2**	VI.2.1.2	759	JQ039391	pigeon	Nigeria	VRD07_231	2007
VI_h	VI.2.1	**VI.2.1.2**	VI.2.1.2	760	AY734536	pigeon	Argentina	Capital_3	1997
VI_h	VI.2.1	**VI.2.1.2**	VI.2.1.2	2021	KX097024.1	pigeon	Brazil	N1065_14	2014
VI_h	VI.2.1	**VI.2.1.2**	VI.2.1.2	2363	MH996920	quail	Nigeria	VRD08_385_N23	2008
VI_h	VI.2.1	**VI.2.1.2**	VI.2.1.2	2435	MH996992	pigeon	Nigeria	Alaba_rago_VRD32_15_32	2015
VI_f	VI.1.2.1	**VI.2.2.1**	VI.2.2.1	706	JN872180	waterfowl	USA	Texas_209682_	2002
VI_f	VI.1.2.1	**VI.2.2.1**	VI.2.2.1	707	JN872182	pigeon	USA	New_York_12339	1998
VI_f	VI.1.2.1	**VI.2.2.1**	VI.2.2.1	708	JN942095	pigeon	USA	Oklahoma_7668_1997_	1997
VI_f	VI.1.2.1	**VI.2.2.1**	VI.2.2.1	709	JN942098	pigeon	USA	North_Carolina_56404_	2000
VI_f	VI.1.2.1	**VI.2.2.1**	VI.2.2.1	710	JN942099	gannet	USA	Pennsylvania_25017_	2000
VI_f	VI.1.2.1	**VI.2.2.1**	VI.2.2.1	711	JN967786	pigeon	USA	New_Jersey_58300_4_	2000
VI_f	VI.1.2.1	**VI.2.2.1**	VI.2.2.1	712	JX901312	pigeon	USA	101	2001
VI_f	VI.1.2.1	**VI.2.2.1**	VI.2.2.1	713	JX901318	duck	USA	PA_0203	2002
VI_f	VI.1.2.1	**VI.2.2.1**	VI.2.2.1	714	JX901341	pigeon	USA	PA_0712	2007
VI_f	VI.1.2.1	**VI.2.2.1**	VI.2.2.1	715	JN872187	pigeon	USA	Illinois_11748	1993
VI_f	VI.1.2.1	**VI.2.2.1**	VI.2.2.1	716	JN872185	pigeon	USA	New_York_32851_2	1984
VI_f	VI.1.2.1	**VI.2.2.1**	VI.2.2.1	717	JN872186	pigeon	USA	Indiana_18002	1991
VI_f	VI.1.2.1	**VI.2.2.1**	VI.2.2.1	718	JN872188	pigeon	USA	Minnesota_2446	1989
VI_f	VI.1.2.1	**VI.2.2.1**	VI.2.2.1	719	FJ410148	pigeon	USA	Texas	1998
VI_f	VI.1.2.1	**VI.2.2.1**	VI.2.2.1	721	JN942022	chicken	USA	Texas_309968	2004
VI_e	VI.1.2.2	**VI.2.2.2**	VI.2.2.2	689	AY325798	pigeon	China	YN_P1	_
VI_e	VI.1.2.2	**VI.2.2.2**	VI.2.2.2	690	DQ417113	pigeon	China	Beijing_PB01_STP	1996
VI_e	VI.1.2.2	**VI.2.2.2**	VI.2.2.2	691	FJ480825	pigeon	China	PG_JS_1	2005

VI_e	VI.1.2.2	**VI.2.2.2**	VI.2.2.2	692	FJ480826	pigeon	China	PG_JS_1	2006
VI_e	VI.1.2.2	**VI.2.2.2**	VI.2.2.2	693	FJ766528	pigeon	China	05_029	2005
VI_e	VI.1.2.2	**VI.2.2.2**	VI.2.2.2	694	GQ338311	_	China	28	2005
VI_e	VI.1.2.2	**VI.2.2.2**	VI.2.2.2	695	JQ290284	pigeon	China	Anhui_1	2009
VI_e	VI.1.2.2	**VI.2.2.2**	VI.2.2.2	696	JX244794	pigeon	China	100	2008
VI_e	VI.1.2.2	**VI.2.2.2**	VI.2.2.2	697	JX244801	pigeon	China	107	2008
VI_e	VI.1.2.2	**VI.2.2.2**	VI.2.2.2	698	JX244803	pigeon	China	110	2008
VI_e	VI.1.2.2	**VI.2.2.2**	VI.2.2.2	699	JX244805	pigeon	China	112	2008
VI_e	VI.1.2.2	**VI.2.2.2**	VI.2.2.2	700	KJ607163	pigeon	China	LJS_1	2004
VI_e	VI.1.2.2	**VI.2.2.2**	VI.2.2.2	701	KJ607164	pigeon	China	LJS_1	2003
VI_e	VI.1.2.2	**VI.2.2.2**	VI.2.2.2	702	KJ808820	pigeon	China	SD	2012
VI_e	VI.1.2.2	**VI.2.2.2**	VI.2.2.2	703	KT381596	pigeon	China	Guangdong_GM8	2013
VI_e	VI.1.2.2	**VI.2.2.2**	VI.2.2.2	704	KX710209.1	pigeon	India	D167	2015
VI_e	VI.1.2.2	**VI.2.2.2**	VI.2.2.2	705	AF358785	pigeon	China	98_1	1998
VII	VII	**VII**	VII	913	U62620	chicken	Taiwan		1995
VII_b	VII.1.1	**VII.1.1**	VII.1.1	933	AF456443	goose	China	JS_9_01_Go	2001
VII_b	VII.1.1	**VII.1.1**	VII.1.1	935	DQ227247	chicken	China	Shandong_SDD	2001
VII_b	VII.1.1	**VII.1.1**	VII.1.1	936	DQ227249	chicken	China	Shandong_SQD	2004
VII_b	VII.1.1	**VII.1.1**	VII.1.1	937	DQ228922	chicken	China	Shandong_SL	2003
VII_b	VII.1.1	**VII.1.1**	VII.1.1	938	DQ363530	chicken	China	Shandong_WHZ	2003
VII_b	VII.1.1	**VII.1.1**	VII.1.1	939	DQ363533	chicken	China	Shandong_SCL	2003
VII_b	VII.1.1	**VII.1.1**	VII.1.1	941	DQ485231	chicken	China	Guangxi_11	2003
VII_b	VII.1.1	**VII.1.1**	VII.1.1	942	DQ485261	chicken	China	Guangxi10	2003
VII_b	VII.1.1	**VII.1.1**	VII.1.1	943	DQ485271	chicken	China	Guangxi14	2005
VII_b	VII.1.1	**VII.1.1**	VII.1.1	944	DQ485274	dove	China	Guangxi15	2005
VII_b	VII.1.1	**VII.1.1**	VII.1.1	945	DQ486859	chicken	China	GM	2001
VII_b	VII.1.1	**VII.1.1**	VII.1.1	946	EF211814	goose	China	JS_8	2006
VII_b	VII.1.1	**VII.1.1**	VII.1.1	947	EF589132	fightcock	China	DQ_Guizhou_	_
VII_b	VII.1.1	**VII.1.1**	VII.1.1	948	EF589133	pheasant	China	98_Guizhou	1998
VII_b	VII.1.1	**VII.1.1**	VII.1.1	949	EF592500	mandarin_duck	China	HLJ_07	2005
VII_b	VII.1.1	**VII.1.1**	VII.1.1	950	EF592501	mandarin_duck	China	HLJ_34	2005
VII_b	VII.1.1	**VII.1.1**	VII.1.1	951	EF592503	mandarin_duck	China	HLJ_42	2006
VII_b	VII.1.1	**VII.1.1**	VII.1.1	952	EF592505	mallard	China	HLJ_50	2006
VII_b	VII.1.1	**VII.1.1**	VII.1.1	953	EF592506	mandarin_duck	China	HLJ_54	2006

VII_b	VII.1.1	**VII.1.1**	VII.1.1	954	EF592507	whitefronted_goose	China	HLJ_75	2006
VII_b	VII.1.1	**VII.1.1**	VII.1.1	955	EF592509	common_teal_	China	HLJ_82	2006
VII_b	VII.1.1	**VII.1.1**	VII.1.1	956	EF592510	mallard	China	HLJ_154	2006
VII_b	VII.1.1	**VII.1.1**	VII.1.1	957	EU258658	chicken	China	20252	2002
VII_b	VII.1.1	**VII.1.1**	VII.1.1	958	EU258661	chicken	China	22433	2002
VII_b	VII.1.1	**VII.1.1**	VII.1.1	959	EU258662	chicken	China	22435	2002
VII_b	VII.1.1	**VII.1.1**	VII.1.1	960	FJ480774	rough_legged_buzzard	China	HLJ009	2006
VII_b	VII.1.1	**VII.1.1**	VII.1.1	961	FJ480775	longearde_owl	China	HLJ012	2006
VII_b	VII.1.1	**VII.1.1**	VII.1.1	962	FJ480776	rough_legged_buzzard	China	HLJ013	2006
VII_b	VII.1.1	**VII.1.1**	VII.1.1	963	FJ480779	Japanese_sparrowhawk	China	AG_HLJ070	2006
VII_b	VII.1.1	**VII.1.1**	VII.1.1	964	FJ480780	mallard	China	HLJ127	2006
VII_b	VII.1.1	**VII.1.1**	VII.1.1	965	FJ480781	mallard	China	HLJ128	2006
VII_b	VII.1.1	**VII.1.1**	VII.1.1	966	FJ480782	mallard	China	HLJ355	2006
VII_b	VII.1.1	**VII.1.1**	VII.1.1	967	FJ480784	mallard	China	HLJ363	2005
VII_b	VII.1.1	**VII.1.1**	VII.1.1	968	FJ480785	mallard	China	HLJ374	2006
VII_b	VII.1.1	**VII.1.1**	VII.1.1	969	FJ480787	mallard	China	HLJ016	2006
VII_b	VII.1.1	**VII.1.1**	VII.1.1	970	FJ480789	chicken	China	GD_1	2005
VII_b	VII.1.1	**VII.1.1**	VII.1.1	971	FJ480796	chicken	China	HN_1	2001
VII_b	VII.1.1	**VII.1.1**	VII.1.1	972	FJ480797	chicken	China	HN_1	2003
VII_b	VII.1.1	**VII.1.1**	VII.1.1	973	FJ480800	chicken	China	JL_1	2003
VII_b	VII.1.1	**VII.1.1**	VII.1.1	974	FJ480801	chicken	China	JL_1	2005
VII_b	VII.1.1	**VII.1.1**	VII.1.1	975	FJ480802	chicken	China	JL_1	2006
VII_b	VII.1.1	**VII.1.1**	VII.1.1	976	FJ480803	chicken	China	JL_2	2003
VII_b	VII.1.1	**VII.1.1**	VII.1.1	977	FJ480804	chicken	China	JL_2	2006
VII_b	VII.1.1	**VII.1.1**	VII.1.1	978	FJ480805	chicken	China	JL_3	2003
VII_b	VII.1.1	**VII.1.1**	VII.1.1	979	FJ480807	chicken	China	JL_5	2003
VII_b	VII.1.1	**VII.1.1**	VII.1.1	980	FJ480820	chicken	China	SHX_1	2006
VII_b	VII.1.1	**VII.1.1**	VII.1.1	981	FJ608335	chicken	China	CJG_Xinjiang_07	2007
VII_b	VII.1.1	**VII.1.1**	VII.1.1	982	FJ608338	chicken	China	SH_Zhejiang	2006
VII_b	VII.1.1	**VII.1.1**	VII.1.1	984	FJ608348	chicken	China	TCQQ_Tianjin	2008
VII_b	VII.1.1	**VII.1.1**	VII.1.1	985	FJ608350	chicken	China	DFGS_Beijing	2008
VII_b	VII.1.1	**VII.1.1**	VII.1.1	986	FJ754271	chicken	China	WF00C	2000
VII_b	VII.1.1	**VII.1.1**	VII.1.1	987	FJ882015	chicken	China	HG_Beijing	2009
VII_b	VII.1.1	**VII.1.1**	VII.1.1	988	GQ245796	chicken	China	AH_2	2008

VII_b	VII.1.1	**VII.1.1**	VII.1.1	989	GQ245797	chicken	China	AN_11	2007
VII_b	VII.1.1	**VII.1.1**	VII.1.1	990	GQ245798	chicken	China	CZ_10	2008
VII_b	VII.1.1	**VII.1.1**	VII.1.1	991	GQ245799	chicken	China	HN_7_06	2006
VII_b	VII.1.1	**VII.1.1**	VII.1.1	992	GQ245800	goose	China	JS_18	2008
VII_b	VII.1.1	**VII.1.1**	VII.1.1	993	GQ245801	pigeon	China	SD_15	2008
VII_b	VII.1.1	**VII.1.1**	VII.1.1	994	GQ245802	chicken	China	SN_6	2008
VII_b	VII.1.1	**VII.1.1**	VII.1.1	995	GQ245803	chicken	China	SN_19	2008
VII_b	VII.1.1	**VII.1.1**	VII.1.1	996	GQ245804	chicken	China	SN_20	2008
VII_b	VII.1.1	**VII.1.1**	VII.1.1	997	GQ245805	chicken	China	SY_1	2008
VII_b	VII.1.1	**VII.1.1**	VII.1.1	998	GQ245806	chicken	China	XY_11	2008
VII_b	VII.1.1	**VII.1.1**	VII.1.1	999	GQ245807	chicken	China	XY_14	2008
VII_b	VII.1.1	**VII.1.1**	VII.1.1	1000	GQ245808	chicken	China	XY_31	2007
VII_b	VII.1.1	**VII.1.1**	VII.1.1	1001	GQ245809	chicken	China	XZ_5	2007
VII_b	VII.1.1	**VII.1.1**	VII.1.1	1002	GQ245810	chicken	China	XZ_7	2008
VII_b	VII.1.1	**VII.1.1**	VII.1.1	1003	GQ245811	chicken	China	XZ_8	2007
VII_b	VII.1.1	**VII.1.1**	VII.1.1	1004	GQ245812	chicken	China	XZ_9	2008
VII_b	VII.1.1	**VII.1.1**	VII.1.1	1005	GQ245813	chicken	China	XZ_12	2007
VII_b	VII.1.1	**VII.1.1**	VII.1.1	1006	GQ245814	chicken	China	XZ_12	2008
VII_b	VII.1.1	**VII.1.1**	VII.1.1	1007	GQ245815	chicken	China	XZ_13	2008
VII_b	VII.1.1	**VII.1.1**	VII.1.1	1008	GQ245816	chicken	China	XZ_16	2007
VII_b	VII.1.1	**VII.1.1**	VII.1.1	1009	GQ245817	chicken	China	XZ_20	2007
VII_b	VII.1.1	**VII.1.1**	VII.1.1	1010	GQ245818	ostrich	China	YZ_22	2007
VII_b	VII.1.1	**VII.1.1**	VII.1.1	1011	GQ245819	chicken	China	ZJ_17	2008
VII_b	VII.1.1	**VII.1.1**	VII.1.1	1013	GU564399	chicken	China	FMW	2006
VII_b	VII.1.1	**VII.1.1**	VII.1.1	1014	HM188398	duck	China	SDFCH	2008
VII_b	VII.1.1	**VII.1.1**	VII.1.1	1015	HM188399	duck	China	SDWF02	2008
VII_b	VII.1.1	**VII.1.1**	VII.1.1	1016	HM188400	duck	China	SDWF03	2008
VII_b	VII.1.1	**VII.1.1**	VII.1.1	1017	HM188401	duck	China	SDWF04	2009
VII_b	VII.1.1	**VII.1.1**	VII.1.1	1018	HM188402	duck	China	SDWF05	2009
VII_b	VII.1.1	**VII.1.1**	VII.1.1	1019	HM748947	chicken	China	SD4	2008
VII_b	VII.1.1	**VII.1.1**	VII.1.1	1020	HQ317395	duck	China	SD09	2009
VII_b	VII.1.1	**VII.1.1**	VII.1.1	1021	HQ717357	duck	China	GD09_1	2009
VII_b	VII.1.1	**VII.1.1**	VII.1.1	1022	JF343539	chicken	China	Guangxi9	2003
VII_b	VII.1.1	**VII.1.1**	VII.1.1	1023	JN400895	duck	China	SD03	2009

VII_b	VII.1.1	**VII.1.1**	VII.1.1	1024	JN400897	chicken	China	SDLY01	2010
VII_b	VII.1.1	**VII.1.1**	VII.1.1	1026	JN631747	goose	China	JS_5	2005
VII_b	VII.1.1	**VII.1.1**	VII.1.1	1027	JQ013855	chicken	China	JS_01	2011
VII_b	VII.1.1	**VII.1.1**	VII.1.1	1028	JQ013857	chicken	China	JS_03	2011
VII_b	VII.1.1	**VII.1.1**	VII.1.1	1029	JQ013858	chicken	China	JS_04	2011
VII_b	VII.1.1	**VII.1.1**	VII.1.1	1030	JQ013859	chicken	China	JS_05	2011
VII_b	VII.1.1	**VII.1.1**	VII.1.1	1031	JQ013863	chicken	China	JS_09	2011
VII_b	VII.1.1	**VII.1.1**	VII.1.1	1032	JQ013866	chicken	China	JS_12	2011
VII_b	VII.1.1	**VII.1.1**	VII.1.1	1033	JQ013867	chicken	China	JS_13	2011
VII_b	VII.1.1	**VII.1.1**	VII.1.1	1034	JQ013869	chicken	China	JS_15	2011
VII_b	VII.1.1	**VII.1.1**	VII.1.1	1035	JQ013874	chicken	China	JS_20	2011
VII_b	VII.1.1	**VII.1.1**	VII.1.1	1036	JQ013876	chicken	China	JS_22	2011
VII_b	VII.1.1	**VII.1.1**	VII.1.1	1037	JQ013879	duck	China	JS_01	2010
VII_b	VII.1.1	**VII.1.1**	VII.1.1	1038	JX193076	chicken	China	Guangxi15	2010
VII_b	VII.1.1	**VII.1.1**	VII.1.1	1039	JX244790	pigeon	China	ZQ_17	2000
VII_b	VII.1.1	**VII.1.1**	VII.1.1	1040	JX244791	pigeon	China	SD_54	2006
VII_b	VII.1.1	**VII.1.1**	VII.1.1	1041	JX244792	pigeon	China	SD_55	2006
VII_b	VII.1.1	**VII.1.1**	VII.1.1	1043	JX482550	seafowl	China	H12	2011
VII_b	VII.1.1	**VII.1.1**	VII.1.1	1044	KC542892	chicken	China	Liaoning_01	2005
VII_b	VII.1.1	**VII.1.1**	VII.1.1	1045	KC542893	chicken	China	Liaoning_02	2005
VII_b	VII.1.1	**VII.1.1**	VII.1.1	1046	KC542899	chicken	China	Jilin_01	2008
VII_b	VII.1.1	**VII.1.1**	VII.1.1	1047	KC542903	chicken	China	Beijing_02	2009
VII_b	VII.1.1	**VII.1.1**	VII.1.1	1048	KC542908	chicken	China	Shandong_01	2011
VII_b	VII.1.1	**VII.1.1**	VII.1.1	1049	KC542909	chicken	China	Shandong_02	2011
VII_b	VII.1.1	**VII.1.1**	VII.1.1	1050	KC750149	duck	China	GD_SD	2006
VII_b	VII.1.1	**VII.1.1**	VII.1.1	1051	KC750156	duck	China	GD_SD	2009
VII_b	VII.1.1	**VII.1.1**	VII.1.1	1052	KC750157	duck	China	GD_SS	2010
VII_b	VII.1.1	**VII.1.1**	VII.1.1	1053	KF219498.1	duck	China	GD_SS	2010
VII_b	VII.1.1	**VII.1.1**	VII.1.1	1054	KJ184574	chicken	China	JS_04	2012
VII_b	VII.1.1	**VII.1.1**	VII.1.1	1055	KJ184591	chicken	China	JS_22	2012
VII_b	VII.1.1	**VII.1.1**	VII.1.1	1056	KJ184600	chicken	China	JS_03	2012
VII_b	VII.1.1	**VII.1.1**	VII.1.1	1057	KJ450977	chicken	China	Jiangxi_07	2009
VII_b	VII.1.1	**VII.1.1**	VII.1.1	1058	KJ525677	goose	China	GD_GD	2008
VII_b	VII.1.1	**VII.1.1**	VII.1.1	1059	KJ525678	duck	China	SD_GM	2008

VII_b	VII.1.1	**VII.1.1**	VII.1.1	1060	KJ525681	duck	China	GD_GDX	2006
VII_b	VII.1.1	**VII.1.1**	VII.1.1	1061	KJ525682	duck	China	GD_GDY	2006
VII_b	VII.1.1	**VII.1.1**	VII.1.1	1062	KJ525683	chicken	China	SX_SX1	2008
VII_b	VII.1.1	**VII.1.1**	VII.1.1	1063	KJ525684	chicken	China	SX_SX2	2008
VII_b	VII.1.1	**VII.1.1**	VII.1.1	1064	KJ525687	chicken	China	SD_XT	2009
VII_b	VII.1.1	**VII.1.1**	VII.1.1	1065	KJ525697	chicken	China	SD_LY2	2008
VII_b	VII.1.1	**VII.1.1**	VII.1.1	1066	KJ525698	chicken	China	SD_LY1	2008
VII_b	VII.1.1	**VII.1.1**	VII.1.1	1067	KJ525709	chicken	China	SD_BD	2010
VII_b	VII.1.1	**VII.1.1**	VII.1.1	1068	KJ525710	chicken	China	GD_ND	2008
VII_b	VII.1.1	**VII.1.1**	VII.1.1	1069	KJ525716	chicken	China	SD_MH	2009
VII_b	VII.1.1	**VII.1.1**	VII.1.1	1070	KJ525718	chicken	China	SD_CQ	2009
VII_b	VII.1.1	**VII.1.1**	VII.1.1	1071	KJ600786	duck	China	SD_134	2009
VII_b	VII.1.1	**VII.1.1**	VII.1.1	1072	KJ607170	quail	China	LJS_101107	2010
VII_b	VII.1.1	**VII.1.1**	VII.1.1	1073	KJ825877	chicken	China	Guangxi_L1	2011
VII_b	VII.1.1	**VII.1.1**	VII.1.1	1074	KP742770	Sheldrake_duck	China	Guizhou_SS1	2014
VII_b	VII.1.1	**VII.1.1**	VII.1.1	1075	KT381593	chicken	China	Guangdong_GM5	2013
VII_b	VII.1.1	**VII.1.1**	VII.1.1	1076	KT381594	duck	China	Guangdong_YF21	2014
VII_b	VII.1.1	**VII.1.1**	VII.1.1	1077	KU200252	goose	China	Jilin_TH01	2015
VII_b	VII.1.1	**VII.1.1**	VII.1.1	1078	KU933951	duck	China	Guizhou_JH	2015
VII_b	VII.1.1	**VII.1.1**	VII.1.1	2005	MF278935.1	white_duck	China	FS_NH_A	2006
VII_d	VII.1.1	**VII.1.1**	VII.1.1	1102	AF358787	fowl	China		1999
VII_d	VII.1.1	**VII.1.1**	VII.1.1	1103	AF358788	fowl	China		2000
VII_d	VII.1.1	**VII.1.1**	VII.1.1	1104	AF364835	fowl	China	98_3	1998
VII_d	VII.1.1	**VII.1.1**	VII.1.1	1105	AF431744	goose	China	ZJ1	2000
VII_d	VII.1.1	**VII.1.1**	VII.1.1	1106	AF456436	goose	China	JS_3	1998
VII_d	VII.1.1	**VII.1.1**	VII.1.1	1107	AF456438	goose	China	ZJ_1	2000
VII_d	VII.1.1	**VII.1.1**	VII.1.1	1109	AF456442	goose	China	JS_5	2001
VII_d	VII.1.1	**VII.1.1**	VII.1.1	1110	AF456444	goose	China	JS_7	2001
VII_d	VII.1.1	**VII.1.1**	VII.1.1	1111	AF473851	goose	China	SF02	2002
VII_d	VII.1.1	**VII.1.1**	VII.1.1	1112	AY253912	parrot	China	YN_PA01	2001
VII_d	VII.1.1	**VII.1.1**	VII.1.1	1113	AY325797	goose	China	GPV_SF02	_
VII_d	VII.1.1	**VII.1.1**	VII.1.1	1115	AY337464	goose	China	_	_
VII_d	VII.1.1	**VII.1.1**	VII.1.1	1116	DQ080015	penguin	China	Beijing	1999
VII_d	VII.1.1	**VII.1.1**	VII.1.1	1117	DQ114478	goose	China	HZ	_

VII_d	VII.1.1	**VII.1.1**	VII.1.1	1118	DQ227248	chicken	China	Shandong_SGM	2001
VII_d	VII.1.1	**VII.1.1**	VII.1.1	1119	DQ227251	chicken	China	Shandong_SKY	2003
VII_d	VII.1.1	**VII.1.1**	VII.1.1	1120	DQ227253	chicken	China	Shandong_SPY	2003
VII_d	VII.1.1	**VII.1.1**	VII.1.1	1121	DQ363531	goose	China	Jiangsu_JS06	2006
VII_d	VII.1.1	**VII.1.1**	VII.1.1	1122	DQ363534	goose	China	Jiangsu_JS03	2003
VII_d	VII.1.1	**VII.1.1**	VII.1.1	1123	DQ363535	chicken	China	Shandong_SF	2002
VII_d	VII.1.1	**VII.1.1**	VII.1.1	1124	DQ363536	chicken	China	Tianjin_TJ	2005
VII_d	VII.1.1	**VII.1.1**	VII.1.1	1125	DQ417110	goose	China	Jiangsu_JS01	2001
VII_d	VII.1.1	**VII.1.1**	VII.1.1	1126	DQ485229	chicken	China	Guangxi_7	2002
VII_d	VII.1.1	**VII.1.1**	VII.1.1	1128	DQ485269	chicken	China	Guangxi13	2004
VII_d	VII.1.1	**VII.1.1**	VII.1.1	1129	DQ839397	chicken	Korea	KBNP_4152	_
VII_d	VII.1.1	**VII.1.1**	VII.1.1	1130	EF128053	wild_duck	China	JS_3	2006
VII_d	VII.1.1	**VII.1.1**	VII.1.1	1131	EF128054	wild_duck	China	JS_1	2006
VII_d	VII.1.1	**VII.1.1**	VII.1.1	1132	EF128055	wild_duck	China	JS_2	2006
VII_d	VII.1.1	**VII.1.1**	VII.1.1	1133	EF175145	muscovy_duck	China	PX2	2003
VII_d	VII.1.1	**VII.1.1**	VII.1.1	1134	EF211807	goose	China	JS_1	2005
VII_d	VII.1.1	**VII.1.1**	VII.1.1	1135	EF211809	goose	China	JS_3	2005
VII_d	VII.1.1	**VII.1.1**	VII.1.1	1136	EF211810	goose	China	JS_4	2005
VII_d	VII.1.1	**VII.1.1**	VII.1.1	1137	EF211811	goose	China	JS_5	2006
VII_d	VII.1.1	**VII.1.1**	VII.1.1	1138	EF211812	goose	China	JS_6	2006
VII_d	VII.1.1	**VII.1.1**	VII.1.1	1139	EF211813	goose	China	JS_7	2006
VII_d	VII.1.1	**VII.1.1**	VII.1.1	1140	EF521889	mallard	China	HLJ_13	2005
VII_d	VII.1.1	**VII.1.1**	VII.1.1	1141	EF540729	goose	China	JG97	_
VII_d	VII.1.1	**VII.1.1**	VII.1.1	1142	EF579731	chicken	China	Shandong_YS	2006
VII_d	VII.1.1	**VII.1.1**	VII.1.1	1143	EF579732	chicken	China	Shandong_DG	2002
VII_d	VII.1.1	**VII.1.1**	VII.1.1	1144	EF579733	chicken	China	Shandong_Pyan	2004
VII_d	VII.1.1	**VII.1.1**	VII.1.1	1145	EF579734	goose	China	Guangdong_GZ	2006
VII_d	VII.1.1	**VII.1.1**	VII.1.1	1146	EF592502	mandarin_duck	China	HLJ_36	2005
VII_d	VII.1.1	**VII.1.1**	VII.1.1	1147	EF592504	bean_goose	China	HLJ_48	2006
VII_d	VII.1.1	**VII.1.1**	VII.1.1	1148	EF592508	mallard	China	HLJ_78	2006
VII_d	VII.1.1	**VII.1.1**	VII.1.1	1149	EU140947		South_Korea	SNU_0202	2000
VII_d	VII.1.1	**VII.1.1**	VII.1.1	1150	EU140948		South_Korea	KBNP_4152	2004
VII_d	VII.1.1	**VII.1.1**	VII.1.1	1151	EU140949		South_Korea	SNU_5070	2005
VII_d	VII.1.1	**VII.1.1**	VII.1.1	1152	EU140950		South_Korea	SNU_5074	2005

VII_d	VII.1.1	**VII.1.1**	VII.1.1	1153	EU258642	chicken	China	7011	2007
VII_d	VII.1.1	**VII.1.1**	VII.1.1	1155	EU583503	chicken	China	Hebei	2004
VII_d	VII.1.1	**VII.1.1**	VII.1.1	1156	EU597811	chicken	China	SHY	2006
VII_d	VII.1.1	**VII.1.1**	VII.1.1	1157	FJ011441	chicken	China	SDCX	2007
VII_d	VII.1.1	**VII.1.1**	VII.1.1	1158	FJ011442	chicken	China	HNDY	2007
VII_d	VII.1.1	**VII.1.1**	VII.1.1	1159	FJ011443	chicken	China	SDMH	2007
VII_d	VII.1.1	**VII.1.1**	VII.1.1	1160	FJ011448	chicken	China	TZ0601	2006
VII_d	VII.1.1	**VII.1.1**	VII.1.1	1161	FJ217665	chicken	China	SC03	2006
VII_d	VII.1.1	**VII.1.1**	VII.1.1	1162	FJ217666	chicken	China	SC05	2006
VII_d	VII.1.1	**VII.1.1**	VII.1.1	1163	FJ240168	goose	China	HLJ_LL01	2008
VII_d	VII.1.1	**VII.1.1**	VII.1.1	1164	FJ426563	chicken	China	SDWF01	2008
VII_d	VII.1.1	**VII.1.1**	VII.1.1	1165	FJ480777	mandarin_duck	China	HLJ028	2006
VII_d	VII.1.1	**VII.1.1**	VII.1.1	1166	FJ480778	whitefronted_goose_	China	HLJ052	2006
VII_d	VII.1.1	**VII.1.1**	VII.1.1	1167	FJ480783	mallard_	China	HLJ361	2005
VII_d	VII.1.1	**VII.1.1**	VII.1.1	1168	FJ480788	wild_bird	China	HLJ001	2006
VII_d	VII.1.1	**VII.1.1**	VII.1.1	1169	FJ480790	chicken	China	HLJ_1	2006
VII_d	VII.1.1	**VII.1.1**	VII.1.1	1170	FJ480791	chicken	China	HLJ_1	2007
VII_d	VII.1.1	**VII.1.1**	VII.1.1	1171	FJ480792	chicken	China	HLJ_2	2006
VII_d	VII.1.1	**VII.1.1**	VII.1.1	1172	FJ480798	chicken	China	HN_1	2004
VII_d	VII.1.1	**VII.1.1**	VII.1.1	1173	FJ480799	chicken	China	HN_1	2007
VII_d	VII.1.1	**VII.1.1**	VII.1.1	1174	FJ480806	chicken	China	JL_4	2003
VII_d	VII.1.1	**VII.1.1**	VII.1.1	1175	FJ480809	chicken	China	LN_1	2006
VII_d	VII.1.1	**VII.1.1**	VII.1.1	1176	FJ480810	chicken	China	LN_2	2006
VII_d	VII.1.1	**VII.1.1**	VII.1.1	1177	FJ480812	chicken	China	LN_4	2006
VII_d	VII.1.1	**VII.1.1**	VII.1.1	1178	FJ480813	chicken	China	NX_1	2005
VII_d	VII.1.1	**VII.1.1**	VII.1.1	1179	FJ480818	chicken	China	NX_5	2006
VII_d	VII.1.1	**VII.1.1**	VII.1.1	1180	FJ480819	chicken	China	SD_1	2006
VII_d	VII.1.1	**VII.1.1**	VII.1.1	1181	FJ480824	mallard_duck	China	GD_1	2005
VII_d	VII.1.1	**VII.1.1**	VII.1.1	1182	FJ608334	chicken	China	WN_Tianin	2003
VII_d	VII.1.1	**VII.1.1**	VII.1.1	1183	FJ608336	chicken	China	ZSM_Hebei	2007
VII_d	VII.1.1	**VII.1.1**	VII.1.1	1184	FJ608343	chicken	China	GM_Shandong	2001
VII_d	VII.1.1	**VII.1.1**	VII.1.1	1185	FJ608345	chicken	China	DFZD_Jilin	2008
VII_d	VII.1.1	**VII.1.1**	VII.1.1	1186	FJ608349	chicken	China	LGQQ_Liaoning	2008
VII_d	VII.1.1	**VII.1.1**	VII.1.1	1187	FJ608351	chicken	China	YZCQ_Liaoning	2008

VII_d	VII.1.1	**VII.1.1**	VII.1.1	1188	FJ754272	duck	China	WF00D	2000
VII_d	VII.1.1	**VII.1.1**	VII.1.1	1189	FJ754273	goose	China	WF00G	2000
VII_d	VII.1.1	**VII.1.1**	VII.1.1	1190	FJ872531	muscovy_duck	China	Fuian_FP1	2002
VII_d	VII.1.1	**VII.1.1**	VII.1.1	1191	GQ245780	chicken	China	AN_9	2007
VII_d	VII.1.1	**VII.1.1**	VII.1.1	1192	GQ245781	chicken	China	CZ_8	2008
VII_d	VII.1.1	**VII.1.1**	VII.1.1	1193	GQ245782	chicken	China	DT_3	2008
VII_d	VII.1.1	**VII.1.1**	VII.1.1	1194	GQ245783	chicken	China	HA_2	2007
VII_d	VII.1.1	**VII.1.1**	VII.1.1	1195	GQ245784	chicken	China	HA_4_08	2008
VII_d	VII.1.1	**VII.1.1**	VII.1.1	1196	GQ245785	chicken	China	HA_9	2006
VII_d	VII.1.1	**VII.1.1**	VII.1.1	1197	GQ245786	chicken	China	HA_13	2007
VII_d	VII.1.1	**VII.1.1**	VII.1.1	1198	GQ245787	chicken	China	HA_14	2007
VII_d	VII.1.1	**VII.1.1**	VII.1.1	1199	GQ245788	chicken	China	HA_15	2007
VII_d	VII.1.1	**VII.1.1**	VII.1.1	1200	GQ245790	chicken	China	HN_6	2007
VII_d	VII.1.1	**VII.1.1**	VII.1.1	1201	GQ245791	chicken	China	SN_5	2008
VII_d	VII.1.1	**VII.1.1**	VII.1.1	1202	GQ245792	chicken	China	SY_17	2007
VII_d	VII.1.1	**VII.1.1**	VII.1.1	1203	GQ245793	goose	China	XZ_18	2007
VII_d	VII.1.1	**VII.1.1**	VII.1.1	1204	GQ245794	goose	China	YZ_19	2007
VII_d	VII.1.1	**VII.1.1**	VII.1.1	1206	GQ255639	goose	China	GPMV_SH	2009
VII_d	VII.1.1	**VII.1.1**	VII.1.1	1207	GQ849007	laying_duck	China	JSD0812	2008
VII_d	VII.1.1	**VII.1.1**	VII.1.1	1208	GU166154	chicken	China	ND_XX	2008
VII_d	VII.1.1	**VII.1.1**	VII.1.1	1209	GU227738	dove	Serbia	Serbia_749	2007
VII_d	VII.1.1	**VII.1.1**	VII.1.1	1210	GU227739	sparrowhawk	Serbia	Serbia_1038	2007
VII_d	VII.1.1	**VII.1.1**	VII.1.1	1211	HM188393	duck	China	SDBZH	2007
VII_d	VII.1.1	**VII.1.1**	VII.1.1	1212	HM188396	duck	China	SDTA	2007
VII_d	VII.1.1	**VII.1.1**	VII.1.1	1213	HM748945	chicken	China	SD2	2008
VII_d	VII.1.1	**VII.1.1**	VII.1.1	1214	HM748946	chicken	China	SD3	2008
VII_d	VII.1.1	**VII.1.1**	VII.1.1	1215	JF340367	goose	China	JSG0210	2002
VII_d	VII.1.1	**VII.1.1**	VII.1.1	1216	JN599167	penguin	China	BP01	1999
VII_d	VII.1.1	**VII.1.1**	VII.1.1	1217	JN872164	chicken	Colombia	440620	2006
VII_d	VII.1.1	**VII.1.1**	VII.1.1	1218	JN986838	chicken	South_Africa	ZA_AL495	2004
VII_d	VII.1.1	**VII.1.1**	VII.1.1	1219	JQ013861	chicken	China	JS_07	2011
VII_d	VII.1.1	**VII.1.1**	VII.1.1	1220	JQ013862	goose	China	JS_08	2011
VII_d	VII.1.1	**VII.1.1**	VII.1.1	1221	JQ013864	chicken	China	JS_10	2011
VII_d	VII.1.1	**VII.1.1**	VII.1.1	1222	JQ319052	chicken	Venezuela	7	2009

VII_d	VII.1.1	**VII.1.1**	VII.1.1	1223	JQ697739	_	Malaysia	1041_632	2009
VII_d	VII.1.1	**VII.1.1**	VII.1.1	1224	JQ894778	chicken	China	HBHD01	2012
VII_d	VII.1.1	**VII.1.1**	VII.1.1	1225	JX519467	chicken	China	JSX1	2010
VII_d	VII.1.1	**VII.1.1**	VII.1.1	1226	KC292514	chicken	China	F72	2010
VII_d	VII.1.1	**VII.1.1**	VII.1.1	1227	KC292517	chicken	China	FH2	2010
VII_d	VII.1.1	**VII.1.1**	VII.1.1	1228	KC461214	chicken	China	TC_9	2011
VII_d	VII.1.1	**VII.1.1**	VII.1.1	1229	KC542895	chicken	China	Hebei_01	2006
VII_d	VII.1.1	**VII.1.1**	VII.1.1	1230	KC542897	chicken	China	Tianjin_01	2007
VII_d	VII.1.1	**VII.1.1**	VII.1.1	1231	KC542898	chicken	China	Tianjin_02	2007
VII_d	VII.1.1	**VII.1.1**	VII.1.1	1232	KC542902	chicken	China	Beijing_01	2012
VII_d	VII.1.1	**VII.1.1**	VII.1.1	1233	KC750150	duck	China	GD_SD	2006
VII_d	VII.1.1	**VII.1.1**	VII.1.1	1234	KC750154	duck	China	GD_JY	2008
VII_d	VII.1.1	**VII.1.1**	VII.1.1	1235	KC853019	crested_ibis	China	Shaanxi	2006
VII_d	VII.1.1	**VII.1.1**	VII.1.1	1236	KF208469	chicken	China	SD883	2013
VII_d	VII.1.1	**VII.1.1**	VII.1.1	1237	KF442615	chicken	South_Africa	8100426	2008
VII_d	VII.1.1	**VII.1.1**	VII.1.1	1238	KF792023	turkey	Israel	832_497	2009
VII_d	VII.1.1	**VII.1.1**	VII.1.1	1240	KJ450979	chicken	China	ShanDong_024	2010
VII_d	VII.1.1	**VII.1.1**	VII.1.1	1241	KJ525676	goose	China	GD_GZ	2006
VII_d	VII.1.1	**VII.1.1**	VII.1.1	1242	KJ525679	duck	China	SD_DZ	2008
VII_d	VII.1.1	**VII.1.1**	VII.1.1	1243	KJ525680	duck	China	SD_LY	2009
VII_d	VII.1.1	**VII.1.1**	VII.1.1	1244	KJ525690	chicken	China	SD_YT	2013
VII_d	VII.1.1	**VII.1.1**	VII.1.1	1245	KJ525702	chicken	China	SD_KD1	2008
VII_d	VII.1.1	**VII.1.1**	VII.1.1	1246	KJ525706	chicken	China	SD_DZ	2007
VII_d	VII.1.1	**VII.1.1**	VII.1.1	1247	KJ525717	chicken	China	SD_MH	2008
VII_d	VII.1.1	**VII.1.1**	VII.1.1	1248	KJ600785	chicken	China	SD_128	2008
VII_d	VII.1.1	**VII.1.1**	VII.1.1	1249	KJ865694	chicken	Colombia	1326_13207	2009
VII_d	VII.1.1	**VII.1.1**	VII.1.1	1250	KJ865699	chicken	Colombia	1326_13563	2009
VII_d	VII.1.1	**VII.1.1**	VII.1.1	1251	KJ865700	chicken	Colombia	1326_13919	2009
VII_d	VII.1.1	**VII.1.1**	VII.1.1	1252	KJ865701	chicken	Colombia	1326_14301	2009
VII_d	VII.1.1	**VII.1.1**	VII.1.1	1253	KJ865706	fighting_rooster	Colombia	2077_26	2009
VII_d	VII.1.1	**VII.1.1**	VII.1.1	1254	KJ865707	chicken	Colombia	2420_06	2010
VII_d	VII.1.1	**VII.1.1**	VII.1.1	1255	KJ865713	chicken	Colombia	5728_17	2009
VII_d	VII.1.1	**VII.1.1**	VII.1.1	1256	KJ865715	chicken	Colombia	12129_14	2009
VII_d	VII.1.1	**VII.1.1**	VII.1.1	1257	KJ865717	chicken	Colombia	14402_17	2008

VII_d	VII.1.1	**VII.1.1**	VII.1.1	1258	KJ914673	chicken	Ukraine	Ivano_Frankivsk_58	2007
VII_d	VII.1.1	**VII.1.1**	VII.1.1	1259	KM885167	mallard	China	LGD_1	2005
VII_d	VII.1.1	**VII.1.1**	VII.1.1	1260	KP189357	mallard	Russia	Adygea_12	2008
VII_d	VII.1.1	**VII.1.1**	VII.1.1	1261	KU140419	chicken	China	ck_LHLJ_1_06	2006
VII_d	VII.1.1	**VII.1.1**	VII.1.1	1262	KU200249	goose	China	Jilin_SP01	2014
VII_d	VII.1.1	**VII.1.1**	VII.1.1	1263	KU200250	goose	China	Jilin_DA05	2015
VII_d	VII.1.1	**VII.1.1**	VII.1.1	1264	KU200251	chicken	China	Heilongjiang_FY06	2015
VII_d	VII.1.1	**VII.1.1**	VII.1.1	1265	KU295450	chicken	Bulgaria	Juper	2006
VII_d	VII.1.1	**VII.1.1**	VII.1.1	1266	KU295451	chicken	Bulgaria	Moravitsa	2007
VII_d	VII.1.1	**VII.1.1**	VII.1.1	1267	KU295452	chicken	Bulgaria	Vidno	2007
VII_d	VII.1.1	**VII.1.1**	VII.1.1	1268	KU295453	chicken	Ukraine	Kharkiv_66_960	2007
VII_d	VII.1.1	**VII.1.1**	VII.1.1	1269	KU295454	chicken	Ukraine	Lyubotyn_961	2003
VII_d	VII.1.1	**VII.1.1**	VII.1.1	1270	KU295455	chicken	Ukraine	Bashtanivske_20_02_962	2013
VII_d	VII.1.1	**VII.1.1**	VII.1.1	1271	KU710269	chicken	Bulgaria	Vodniantsi	2006
VII_d	VII.1.1	**VII.1.1**	VII.1.1	1272	KU710271	chicken	Bulgaria	Kravoder	2007
VII_d	VII.1.1	**VII.1.1**	VII.1.1	1273	KU710272	chicken	Bulgaria	Liliashka_Mogila	2008
VII_d	VII.1.1	**VII.1.1**	VII.1.1	1274	KU710273	chicken	Bulgaria	Kardam	2008
VII_d	VII.1.1	**VII.1.1**	VII.1.1	1275	KU710274	chicken	Bulgaria	Kazatsite	2008
VII_d	VII.1.1	**VII.1.1**	VII.1.1	1276	KU710275	chicken	Bulgaria	Vozhdovo	2009
VII_d	VII.1.1	**VII.1.1**	VII.1.1	1277	KU710276	chicken	Bulgaria	Mamarchevo	2009
VII_d	VII.1.1	**VII.1.1**	VII.1.1	1278	KU710277	pigeon	Ukraine	Simferopol_2_26	2011
VII_d	VII.1.1	**VII.1.1**	VII.1.1	1279	KU710278	chicken	Ukraine	Lypova_Dolyna	2002
VII_d	VII.1.1	**VII.1.1**	VII.1.1	1280	KU710279	chicken	Ukraine	Lugansk	2003
VII_d	VII.1.1	**VII.1.1**	VII.1.1	1281	KU726619	mallard	Russia	Adygea_927	2007
VII_d	VII.1.1	**VII.1.1**	VII.1.1	1284	KY404087	chicken	South_Korea	KR_005_00	2013
VII_e	VII.1.1	**VII.1.1**	VII.1.1	1285	AF162714	goose	China_Guangdong_	GPMY_QY97_1	1997
VII_e	VII.1.1	**VII.1.1**	VII.1.1	1286	AF358786	chicken	Taiwan	TW	2000
VII_e	VII.1.1	**VII.1.1**	VII.1.1	1287	AF456437	goose	China	GD_1	1998
VII_e	VII.1.1	**VII.1.1**	VII.1.1	1288	AY325799	_	_	YN_C1	_
VII_e	VII.1.1	**VII.1.1**	VII.1.1	1289	DQ067447	chicken	China	GX1	2000
VII_e	VII.1.1	**VII.1.1**	VII.1.1	1290	DQ363537	chicken	China	Shandong_Jlan_04	2004
VII_e	VII.1.1	**VII.1.1**	VII.1.1	1291	DQ485256	chicken	China	Guangxi2	2000
VII_e	VII.1.1	**VII.1.1**	VII.1.1	1292	DQ485258	chicken	China	Guangxi4	2000
VII_e	VII.1.1	**VII.1.1**	VII.1.1	1293	EF589131	chicken	China	P1_Guizhou	_

VII_e	VII.1.1	**VII.1.1**	VII.1.1	1295	GU332646	duck	Vietnam	Long_Bien_78	2002
VII_e	VII.1.1	**VII.1.1**	VII.1.1	1296	GU332647	environment	Vietnam	environmental_fecal_480	2002
VII_e	VII.1.1	**VII.1.1**	VII.1.1	1297	JX193075	chicken	China	Guangxi14	2002
VII_e	VII.1.1	**VII.1.1**	VII.1.1	1298	KJ782375	goose	China	GD_QY	1997
VII_e	VII.1.1	**VII.1.1**	VII.1.1	1307	AB853329	chicken	Japan	Fukushima_NYF_3	2002
VII_e	VII.1.1	**VII.1.1**	VII.1.1	1308	AB853927	chicken	Japan	Ibaraki_SG106	1999
VII_e	VII.1.1	**VII.1.1**	VII.1.1	1309	AB853929	chicken	Japan	Miyagi_AGT	2002
VII_e	VII.1.1	**VII.1.1**	VII.1.1	1610	DQ659677	goose	China	NA1	1999
VII_e	VII.1.1	**VII.1.1**	VII.1.1	1611	FJ480822	goose	China	HLJ_1	2006
VII_e	VII.1.1	**VII.1.1**	VII.1.1	1612	KJ528559	goose	China	NA_1M	2014
VII_e	VII.1.1	**VII.1.1**	VII.1.1	1613	KJ607169	goose	China	LHLJ_1	2006
VII_e	VII.1.1	**VII.1.1**	VII.1.1	1614	KU200243	chicken	China	Jilin_LY02	2014
VII_e	VII.1.1	**VII.1.1**	VII.1.1	1615	KU200244	chicken	China	Jilin_CY01	2014
VII_e	VII.1.1	**VII.1.1**	VII.1.1	1616	KU200245	quail	China	Jilin_JY02	2015
VII_e	VII.1.1	**VII.1.1**	VII.1.1	1617	KU200246	chicken	China	Jilin_YJ05	2015
VII_e	VII.1.1	**VII.1.1**	VII.1.1	1618	KU200247	pigeon	China	Heilongjiang_TL03	2015
VII_e	VII.1.1	**VII.1.1**	VII.1.1	1619	KU200248	pigeon	China	Heilongjiang_LS03	2014
VII_e	VII.1.1	**VII.1.1**	VII.1.1	1996	MF278925.1	chicken	China	YF_LD_L	1997
VII_j	VII.1.1	**VII.1.1**	VII.1.1	1478	FJ882014	chicken	China	SG_Liaoning	2009
VII_j	VII.1.1	**VII.1.1**	VII.1.1	1479	JQ013856	chicken	China	JS_02	2011
VII_j	VII.1.1	**VII.1.1**	VII.1.1	1480	JQ013860	chicken	China	JS_06	2011
VII_j	VII.1.1	**VII.1.1**	VII.1.1	1481	JQ013865	chicken	China	JS_11	2011
VII_j	VII.1.1	**VII.1.1**	VII.1.1	1482	JQ013868	chicken	China	JS_14	2011
VII_j	VII.1.1	**VII.1.1**	VII.1.1	1483	JQ013870	chicken	China	JS_16	2011
VII_j	VII.1.1	**VII.1.1**	VII.1.1	1484	JQ013871	chicken	China	JS_17	2011
VII_j	VII.1.1	**VII.1.1**	VII.1.1	1485	JQ013873	chicken	China	JS_19	2011
VII_j	VII.1.1	**VII.1.1**	VII.1.1	1486	JQ013877	chicken	China	SD_01	2011
VII_j	VII.1.1	**VII.1.1**	VII.1.1	1487	JQ013878	chicken	China	SD_02	2011
VII_j	VII.1.1	**VII.1.1**	VII.1.1	1488	JQ015295	chicken	China	SDWF07	2011
VII_j	VII.1.1	**VII.1.1**	VII.1.1	1489	JQ015296	chicken	China	SD04	2011
VII_j	VII.1.1	**VII.1.1**	VII.1.1	1490	JQ015297	chicken	China	SDYT03	2011
VII_j	VII.1.1	**VII.1.1**	VII.1.1	1491	JX840452	chicken	China	SD754	2012
VII_j	VII.1.1	**VII.1.1**	VII.1.1	1492	JX840453	chicken	China	SD755	2012
VII_j	VII.1.1	**VII.1.1**	VII.1.1	1493	JX840454	chicken	China	SD704	2012

VII_j	VII.1.1	**VII.1.1**	VII.1.1	1494	JX840455	chicken	China	SD758	2012
VII_j	VII.1.1	**VII.1.1**	VII.1.1	1495	KC020114	chicken	China	SDS672	2012
VII_j	VII.1.1	**VII.1.1**	VII.1.1	1496	KC292519	chicken	China	SDS672	2010
VII_j	VII.1.1	**VII.1.1**	VII.1.1	1497	KC484655	chicken	Israel	Israel_998	2011
VII_j	VII.1.1	**VII.1.1**	VII.1.1	1498	KC489471	chicken	China	SD834_12	2012
VII_j	VII.1.1	**VII.1.1**	VII.1.1	1499	KC542905	chicken	China	Liaoning_01	2009
VII_j	VII.1.1	**VII.1.1**	VII.1.1	1500	KC542906	chicken	China	Hebei_01	2010
VII_j	VII.1.1	**VII.1.1**	VII.1.1	1501	KC542907	chicken	China	Shandong_02	2010
VII_j	VII.1.1	**VII.1.1**	VII.1.1	1502	KC542910	chicken	China	Hebei_01	2011
VII_j	VII.1.1	**VII.1.1**	VII.1.1	1503	KC542911	chicken	China	Beijing_01	2009
VII_j	VII.1.1	**VII.1.1**	VII.1.1	1504	KC542912	chicken	China	Shandong_01	2012
VII_j	VII.1.1	**VII.1.1**	VII.1.1	1505	KF055273	chicken	China	SD01	2013
VII_j	VII.1.1	**VII.1.1**	VII.1.1	1506	KF306265	wildfowl	China	SF2	2010
VII_j	VII.1.1	**VII.1.1**	VII.1.1	1507	KF771883	duck	China	LC	2012
VII_j	VII.1.1	**VII.1.1**	VII.1.1	1508	KF792022	pheasant	Israel	746_828	2013
VII_j	VII.1.1	**VII.1.1**	VII.1.1	1509	KF935230	chicken	China	YT	2011
VII_j	VII.1.1	**VII.1.1**	VII.1.1	1510	KJ136258	chicken	China	JLJT	2012
VII_j	VII.1.1	**VII.1.1**	VII.1.1	1511	KJ136259	duck	China	JLQG	2013
VII_j	VII.1.1	**VII.1.1**	VII.1.1	1512	KJ136260	goose	China	JLCC	2012
VII_j	VII.1.1	**VII.1.1**	VII.1.1	1513	KJ136261	pigeon	China	JLDH	2013
VII_j	VII.1.1	**VII.1.1**	VII.1.1	1514	KJ184575	chicken	China	JS_05	2012
VII_j	VII.1.1	**VII.1.1**	VII.1.1	1515	KJ184576	chicken	China	JS_06	2012
VII_j	VII.1.1	**VII.1.1**	VII.1.1	1516	KJ184577	chicken	China	JS_07	2012
VII_j	VII.1.1	**VII.1.1**	VII.1.1	1517	KJ184578	chicken	China	JS_08	2012
VII_j	VII.1.1	**VII.1.1**	VII.1.1	1518	KJ184581	chicken	China	JS_11	2012
VII_j	VII.1.1	**VII.1.1**	VII.1.1	1520	KJ184586	chicken	China	JS_17	2012
VII_j	VII.1.1	**VII.1.1**	VII.1.1	1521	KJ184587	chicken	China	JS_18	2012
VII_j	VII.1.1	**VII.1.1**	VII.1.1	1522	KJ184588	chicken	China	JS_19	2012
VII_j	VII.1.1	**VII.1.1**	VII.1.1	1523	KJ184589	chicken	China	JS_20	2012
VII_j	VII.1.1	**VII.1.1**	VII.1.1	1524	KJ184592	chicken	China	JS_23	2012
VII_j	VII.1.1	**VII.1.1**	VII.1.1	1525	KJ184593	chicken	China	JS_24	2012
VII_j	VII.1.1	**VII.1.1**	VII.1.1	1526	KJ184594	chicken	China	SD_01	2012
VII_j	VII.1.1	**VII.1.1**	VII.1.1	1527	KJ184595	chicken	China	SD_02	2012
VII_j	VII.1.1	**VII.1.1**	VII.1.1	1528	KJ184596	chicken	China	SD_03	2012

VII_j	VII.1.1	**VII.1.1**	VII.1.1	1529	KJ184597	chicken	China	AH_02	2012
VII_j	VII.1.1	**VII.1.1**	VII.1.1	1530	KJ450978	chicken	China	Ningxia_S006	2013
VII_j	VII.1.1	**VII.1.1**	VII.1.1	1531	KJ450980	chicken	Chian	Sichuan_1125	2013
VII_j	VII.1.1	**VII.1.1**	VII.1.1	1532	KJ525670	chicken	China	SD_SH1	2011
VII_j	VII.1.1	**VII.1.1**	VII.1.1	1533	KJ525686	chicken	China	SD_YS	2010
VII_j	VII.1.1	**VII.1.1**	VII.1.1	1534	KJ525688	chicken	China	SD_YT	2011
VII_j	VII.1.1	**VII.1.1**	VII.1.1	1535	KJ525689	chicken	China	SD_ZJ	2012
VII_j	VII.1.1	**VII.1.1**	VII.1.1	1536	KJ525691	chicken	China	SD_YS1	2012
VII_j	VII.1.1	**VII.1.1**	VII.1.1	1537	KJ525693	chicken	China	SD_YG	2013
VII_j	VII.1.1	**VII.1.1**	VII.1.1	1538	KJ525694	chicken	China	SD_XT1	2012
VII_j	VII.1.1	**VII.1.1**	VII.1.1	1539	KJ525695	chicken	China	SD_XT	2011
VII_j	VII.1.1	**VII.1.1**	VII.1.1	1540	KJ525696	chicken	China	SD_WF	2012
VII_j	VII.1.1	**VII.1.1**	VII.1.1	1541	KJ525699	chicken	China	SD_LQ	2011
VII_j	VII.1.1	**VII.1.1**	VII.1.1	1542	KJ525704	chicken	China	SD_HZ	2012
VII_j	VII.1.1	**VII.1.1**	VII.1.1	1543	KJ525705	chicken	China	SD_HZ	2011
VII_j	VII.1.1	**VII.1.1**	VII.1.1	1544	KJ525707	chicken	China	SD_XT2	2012
VII_j	VII.1.1	**VII.1.1**	VII.1.1	1545	KJ525708	chicken	China	SD_CP	2012
VII_j	VII.1.1	**VII.1.1**	VII.1.1	1546	KJ525712	chicken	China	SD_MH10	2011
VII_j	VII.1.1	**VII.1.1**	VII.1.1	1547	KJ525714	chicken	China	SD_MH5	2011
VII_j	VII.1.1	**VII.1.1**	VII.1.1	1548	KJ525715	chicken	China	SD_MH4	2011
VII_j	VII.1.1	**VII.1.1**	VII.1.1	1549	KJ567597	chicken	China	SDZB11	2013
VII_j	VII.1.1	**VII.1.1**	VII.1.1	1550	KJ825878	chicken	China	Guangxi_D2	2012
VII_j	VII.1.1	**VII.1.1**	VII.1.1	1551	KM016457	chicken	Israel	159	2014
VII_j	VII.1.1	**VII.1.1**	VII.1.1	1552	KM016458	chicken	Israel	160	2014
VII_j	VII.1.1	**VII.1.1**	VII.1.1	1553	KM016459	chicken	Israel	180	2014
VII_j	VII.1.1	**VII.1.1**	VII.1.1	1554	KP064014	chicken	China	JS_21	2013
VII_j	VII.1.1	**VII.1.1**	VII.1.1	1555	KP064015	chicken	China	JS_22	2013
VII_j	VII.1.1	**VII.1.1**	VII.1.1	1556	KP064016	chicken	China	JS_23	2013
VII_j	VII.1.1	**VII.1.1**	VII.1.1	1557	KP064017	chicken	China	JS_24	2013
VII_j	VII.1.1	**VII.1.1**	VII.1.1	1558	KP064018	chicken	China	JS_27	2013
VII_j	VII.1.1	**VII.1.1**	VII.1.1	1559	KP064019	chicken	China	SD_25	2013
VII_j	VII.1.1	**VII.1.1**	VII.1.1	1560	KP064020	chicken	China	JS_30	2013
VII_j	VII.1.1	**VII.1.1**	VII.1.1	1562	KT804693	chicken	Israel	318_IS	2015
VII_j	VII.1.1	**VII.1.1**	VII.1.1	1563	KU200253	chicken	China	Jilin_YJ06	2015

VII_j	VII.1.1	**VII.1.1**	VII.1.1	1564	KU365650	chicken	Egypt	FU4_NLQP	2014
VII_j	VII.1.1	**VII.1.1**	VII.1.1	1565	KU365651	chicken	Egypt	FU8_NLQP	2014
VII_j	VII.1.1	**VII.1.1**	VII.1.1	1566	KU365652	chicken	Egypt	FU13_NLQP	2014
VII_j	VII.1.1	**VII.1.1**	VII.1.1	1567	KU365653	chicken	Egypt	FU5_NLQP	2014
VII_j	VII.1.1	**VII.1.1**	VII.1.1	1568	KU933948	duck	China	Guizhou_ZY	2014
VII_j	VII.1.1	**VII.1.1**	VII.1.1	1569	KU933949	duck	China	Guizhou_SS2	2015
VII_j	VII.1.1	**VII.1.1**	VII.1.1	1570	KU933950	duck	China	Guizhou_TZ	2015
VII_j	VII.1.1	**VII.1.1**	VII.1.1	1571	KX580300.1	chicken	Israel	1421	2015
VII_j	VII.1.1	**VII.1.1**	VII.1.1	1572	KX686722.1		Egypt	ISM_460F	2013
VII_j	VII.1.1	**VII.1.1**	VII.1.1	1573	KX686723.1		Egypt	KFR_B7	2012
VII_j	VII.1.1	**VII.1.1**	VII.1.1	1574	KX686725.1		Egypt	GZ_339F	2015
VII_j	VII.1.1	**VII.1.1**	VII.1.1	1575	KX686724.1		Egypt	EG_35	2014
VII_j	VII.1.1	**VII.1.1**	VII.1.1	1576	KX686726.1		Egypt	BEH_261F	2014
VII_j	VII.1.1	**VII.1.1**	VII.1.1	1577	KX686727.1		Egypt	GZ_986F	2015
VII_j	VII.1.1	**VII.1.1**	VII.1.1	1578	KX686728.1		Egypt	GHB_328F	2016
VII_j	VII.1.1	**VII.1.1**	VII.1.1	1579	KY073880.1	chicken	Israel	IS_266	2016
VII_j	VII.1.1	**VII.1.1**	VII.1.1	1580	KY075880	chicken	Egypt	Sharkia7	2016
VII_j	VII.1.1	**VII.1.1**	VII.1.1	1581	KY075881	chicken	Egypt	Ismailia8	2016
VII_j	VII.1.1	**VII.1.1**	VII.1.1	1582	KY075882	chicken	Egypt	Damietta9	2016
VII_j	VII.1.1	**VII.1.1**	VII.1.1	1583	KY075883	chicken	Egypt	Sharkia10	2016
VII_j	VII.1.1	**VII.1.1**	VII.1.1	1584	KY075884	chicken	Egypt	Qualyobia11	2016
VII_j	VII.1.1	**VII.1.1**	VII.1.1	1585	KY075885	chicken	Egypt	Qualyobia12	2016
VII_j	VII.1.1	**VII.1.1**	VII.1.1	1586	KY075886	chicken	Egypt	Sharkia14	2016
VII_j	VII.1.1	**VII.1.1**	VII.1.1	1587	KY075887	chicken	Egypt	El_Arish15	2016
VII_j	VII.1.1	**VII.1.1**	VII.1.1	1588	KY075888	chicken	Egypt	El_Arish16	2016
VII_j	VII.1.1	**VII.1.1**	VII.1.1	1589	KY075889	chicken	Egypt	Qualyobia26	2016
VII_j	VII.1.1	**VII.1.1**	VII.1.1	1590	KY075890	chicken	Egypt	Dakahlia27	2016
VII_j	VII.1.1	**VII.1.1**	VII.1.1	1591	KY075891	chicken	Egypt	Dakahlia28	2016
VII_j	VII.1.1	**VII.1.1**	VII.1.1	1592	KY075892	chicken	Egypt	Ismailia29	2016
VII_j	VII.1.1	**VII.1.1**	VII.1.1	1593	KY075895	chicken	Egypt	Ismailia32	2016
VII_j	VII.1.1	**VII.1.1**	VII.1.1	1595	KY510685.1	chicken	Israel	428	2016
VII_j	VII.1.1	**VII.1.1**	VII.1.1	1596	KY510687.1	chicken	Israel	2	2017
VII_j	VII.1.1	**VII.1.1**	VII.1.1	1597	KY510688.1	chicken	Israel	27	2017
VII_j	VII.1.1	**VII.1.1**	VII.1.1	1598	KY510689.1	chicken	Israel	28	2017

VII_j	VII.1.1	**VII.1.1**	VII.1.1	1599	KY968651.1	chicken	Israel	IS_222	2017
VII_j	VII.1.1	**VII.1.1**	VII.1.1	1600	MF049232.1	chicken	Israel	IS_195	2017
VII_j	VII.1.1	**VII.1.1**	VII.1.1	1601	MF686923.1	chicken	Israel	IS_235	2011
VII_l	VII.1.1	**VII.1.1**	VII.1.1	1282	KX268351	chicken	Iran	Behshahr	2015
VII_l	VII.1.1	**VII.1.1**	VII.1.1	1986	KU201408	chicken	Iran	SMV_1	2011
VII_l	VII.1.1	**VII.1.1**	VII.1.1	1987	KU201409	chicken	Iran	SMV_2	2011
VII_l	VII.1.1	**VII.1.1**	VII.1.1	1988	KU201410	chicken	Iran	SMV_3	2011
VII_l	VII.1.1	**VII.1.1**	VII.1.1	1989	KU201411	chicken	Iran	SMV_4	2012
VII_l	VII.1.1	**VII.1.1**	VII.1.1	1990	KU201412	chicken	Iran	SMV_5	2012
VII_l	VII.1.1	**VII.1.1**	VII.1.1	1991	KU201413	chicken	Iran	SMV_6	2012
VII_l	VII.1.1	**VII.1.1**	VII.1.1	1992	KU201414	chicken	Iran	SMV_7	2013
VII_l	VII.1.1	**VII.1.1**	VII.1.1	1993	KU201415	chicken	Iran	SMV_8	2013
VII_d	VII.1.1	**VII.1.1**	VII.1.1	2032	KY776610.1	white_fronted_goose	China	HLJ052	2006
VII_b	VII.1.1	**VII.1.1**	VII.1.1	2033	KY776609.1	mandarin_duck	China	JL01	2007
VII_d	VII.1.1	**VII.1.1**	VII.1.1	2034	KY776608.1	wild_bird	China	HLJ001	2006
VII_b	VII.1.1	**VII.1.1**	VII.1.1	2035	KY776607.1	Long_eared_owl	China	HLJ012	2006
VII_b	VII.1.1	**VII.1.1**	VII.1.1	2036	KY776606.1	Mandarin_duck	China	HLJ016	2006
VII_d	VII.1.1	**VII.1.1**	VII.1.1	2037	KY776605.1	Mandarin_dcuk	China	HLJ028	2006
VII_d	VII.1.1	**VII.1.1**	VII.1.1	2041	KY776601.1	mallard	China	HLJ361	2005
VII_b	VII.1.1	**VII.1.1**	VII.1.1	2042	KY776600.1	mallard	China	HLJ355	2006
VII_b	VII.1.1	**VII.1.1**	VII.1.1	2045	KY776593.1	Rough_legged_Buzzard	China	HLJ013	2006
VII_b	VII.1.1	**VII.1.1**	VII.1.1	2046	KY776592.1	Rough_legged_Buzzard	China	HLJ09	2006
VII_b	VII.1.1	**VII.1.1**	VII.1.1	2047	KY776591.1	Japanese_sparrowhawk	China	HLJ070	2006
VII_d	VII.1.1	**VII.1.1**	VII.1.1	2051	MG867723.1	chicken	China	G7	2008
VII_b	VII.1.1	**VII.1.1**	VII.1.1	2055	MG976930.1	Blyths_reed_warbler	Kazakhstan	Almaty_59	2014
VII_d	VII.1.1	**VII.1.1**	VII.1.1	2066	KX765177.1	duck	China	DU_FJCL117	2011
VII_d	VII.1.1	**VII.1.1**	VII.1.1	2067	KX765178.1	duck	China	DU_JXNC35	2012
VII_b	VII.1.1	**VII.1.1**	VII.1.1	2138	MG869266.1	chicken	Vietnam	NCXCP	2011
VII_b	VII.1.1	**VII.1.1**	VII.1.1	2139	MG869267.1	chicken	Vietnam	NDVHN14	2014
VII_d	VII.1.1	**VII.1.1**	VII.1.1	2153	MH371022.1	chicken	Israel	PHL1818	2007
VII_j	VII.1.1	**VII.1.1**	VII.1.1	2154	MH371023.1	chicken	Israel	PHL120809	2011
VII_j	VII.1.1	**VII.1.1**	VII.1.1	2156	MH371025.1	chicken	Israel	PHL141353	2011
VII_j	VII.1.1	**VII.1.1**	VII.1.1	2159	MH371028.1	chicken	Israel	PHL120423	2011
VII_d	VII.1.1	**VII.1.1**	VII.1.1	2163	MH371032.1	chicken	Israel	PHL11278	2011

VII_j	VII.1.1	**VII.1.1**	VII.1.1	2164	MH371033.1	chicken	Israel	PHL137495	2011
VII_j	VII.1.1	**VII.1.1**	VII.1.1	2169	MH371038.1	chicken	Israel	PHL122509	2011
VII_j	VII.1.1	**VII.1.1**	VII.1.1	2170	MH371039.1	chicken	Israel	PHL137569	2011
VII_d	VII.1.1	**VII.1.1**	VII.1.1	2171	MH371040.1	chicken	Israel	PHL99188	2010
VII_j	VII.1.1	**VII.1.1**	VII.1.1	2175	MH371044.1	chicken	Israel	PHL125877	2011
VII_j	VII.1.1	**VII.1.1**	VII.1.1	2176	MH371045.1	chicken	Israel	PHL118279	2011
VII_d	VII.1.1	**VII.1.1**	VII.1.1	2178	MH371047.1	chicken	Israel	PHL114112	2010
VII_j	VII.1.1	**VII.1.1**	VII.1.1	2179	MH371048.1	chicken	Israel	PHL173849	2013
VII_j	VII.1.1	**VII.1.1**	VII.1.1	2192	MH371061.1	chicken	Israel	PHL116035	2011
VII_j	VII.1.1	**VII.1.1**	VII.1.1	2194	MH371063.1	chicken	Israel	PHL232809	2015
VII_j	VII.1.1	**VII.1.1**	VII.1.1	2195	MH371064.1	chicken	Israel	PHL253633	2016
VII_j	VII.1.1	**VII.1.1**	VII.1.1	2196	MH371065.1	turkey	Israel	PHL245632	2016
VII_j	VII.1.1	**VII.1.1**	VII.1.1	2199	MH371068.1	chicken	Israel	PHL153812	2012
VII_j	VII.1.1	**VII.1.1**	VII.1.1	2200	MH371069.1	chicken	Israel	PHL156916	2012
VII_j	VII.1.1	**VII.1.1**	VII.1.1	2202	MH371071.1	chicken	Israel	PHL169730	2013
VII_j	VII.1.1	**VII.1.1**	VII.1.1	2204	MH371073.1	chicken	Israel	PHL201029	2014
VII_j	VII.1.1	**VII.1.1**	VII.1.1	2206	MH371075.1	chicken	Israel	PHL203762	2014
VII_j	VII.1.1	**VII.1.1**	VII.1.1	2209	MH371078.1	chicken	Israel	PHL207436	2014
VII_j	VII.1.1	**VII.1.1**	VII.1.1	2210	MH371079.1	chicken	Israel	PHL209108	2014
VII_j	VII.1.1	**VII.1.1**	VII.1.1	2212	MH371081.1	chicken	Israel	PHL218637	2014
VII_j	VII.1.1	**VII.1.1**	VII.1.1	2215	MH371084.1	chicken	Israel	PHL223759	2015
VII_j	VII.1.1	**VII.1.1**	VII.1.1	2231	MH371100.1	chicken	Israel	PHL208243	2014
VII_d	VII.1.1	**VII.1.1**	VII.1.1	2234	MH377246.1	chicken	Israel	PHL34617	2008
VII_d	VII.1.1	**VII.1.1**	VII.1.1	2238	MH377250.1	pigeon	Israel	PHL76558	2009
VII_d	VII.1.1	**VII.1.1**	VII.1.1	2239	MH377251.1	chicken	Israel	PHL87809	2010
VII_d	VII.1.1	**VII.1.1**	VII.1.1	2240	MH377252.1	chicken	Israel	PHL88629	2010
VII_d	VII.1.1	**VII.1.1**	VII.1.1	2241	MH377253.1	chicken	Israel	PHL95252	2010
VII_d	VII.1.1	**VII.1.1**	VII.1.1	2242	MH377254.1		Israel	PHL95493	2010
VII_d	VII.1.1	**VII.1.1**	VII.1.1	2243	MH377255.1		Israel	PHL97202	2010
VII_j	VII.1.1	**VII.1.1**	VII.1.1	2245	MH377257.1	chicken	Israel	PHL117244	2011
VII_j	VII.1.1	**VII.1.1**	VII.1.1	2246	MH377258.1	chicken	Israel	PHL119228	2011
VII_j	VII.1.1	**VII.1.1**	VII.1.1	2247	MH377259.1	chicken	Israel	PHL121082	2011
VII_j	VII.1.1	**VII.1.1**	VII.1.1	2248	MH377260.1	chicken	Israel	PHL121551	2011
VII_j	VII.1.1	**VII.1.1**	VII.1.1	2249	MH377261.1	chicken	Israel	PHL125051	2011

VII_j	VII.1.1	**VII.1.1**	VII.1.1	2250	MH377262.1	chicken	Israel	PHL141932	2012
VII_j	VII.1.1	**VII.1.1**	VII.1.1	2252	MH377264.1	chicken	Israel	PHL145002	2012
VII_j	VII.1.1	**VII.1.1**	VII.1.1	2253	MH377265.1	turkey	Israel	PHL149777	2012
VII_j	VII.1.1	**VII.1.1**	VII.1.1	2256	MH377268.1	turkey	Israel	PHL173633	2013
VII_d	VII.1.1	**VII.1.1**	VII.1.1	2258	MH377270.1	chicken	Israel	PHL2041	2007
VII_d	VII.1.1	**VII.1.1**	VII.1.1	2259	MH377271.1	chicken	Israel	PHL5869	2007
VII_d	VII.1.1	**VII.1.1**	VII.1.1	2260	MH377272.1	chicken	Israel	PHL9273	2007
VII_d	VII.1.1	**VII.1.1**	VII.1.1	2261	MH377273.1	chicken	Israel	PHL11365	2007
VII_j	VII.1.1	**VII.1.1**	VII.1.1	2262	MH377274.1	chicken	Israel	PHL124027	2011
VII_d	VII.1.1	**VII.1.1**	VII.1.1	2263	MH377275.1	chicken	Israel	PHL23536	2007
VII_d	VII.1.1	**VII.1.1**	VII.1.1	2264	MH377276.1	turkey	Israel	PHL24317	2007
VII_d	VII.1.1	**VII.1.1**	VII.1.1	2265	MH377277.1	chicken	Israel	PHL24639	2007
VII_d	VII.1.1	**VII.1.1**	VII.1.1	2267	MH377279.1	chicken	Israel	PHL27571	2007
VII_d	VII.1.1	**VII.1.1**	VII.1.1	2268	MH377280.1	chicken	Israel	PHL17222	2007
VII_d	VII.1.1	**VII.1.1**	VII.1.1	2271	MH377283.1	chicken	Israel	PHL24993	2007
VII_j	VII.1.1	**VII.1.1**	VII.1.1	2272	MH377284.1	chicken	Israel	PHL151347	2012
VII_j	VII.1.1	**VII.1.1**	VII.1.1	2274	MH377286.1	chicken	Israel	PHL223397	2015
VII_j	VII.1.1	**VII.1.1**	VII.1.1	2275	MH377287.1	chicken	Israel	PHL238651	2015
VII_j	VII.1.1	**VII.1.1**	VII.1.1	2276	MH377288.1		Israel	PHL204642	2014
VII_j	VII.1.1	**VII.1.1**	VII.1.1	2279	MH377291.1	chicken	Israel	PHL251453	2016
VII_j	VII.1.1	**VII.1.1**	VII.1.1	2281	MH377293.1	chicken	Israel	PHL251747	2016
VII_j	VII.1.1	**VII.1.1**	VII.1.1	2282	MH377294.1	chicken	Israel	PHL253817	2016
VII_j	VII.1.1	**VII.1.1**	VII.1.1	2284	MH377296.1	chicken	Israel	PHL256987	2016
VII_j	VII.1.1	**VII.1.1**	VII.1.1	2285	MH377297.1	turkey	Israel	PHL257758	2016
VII_j	VII.1.1	**VII.1.1**	VII.1.1	2289	MH377301.1	long_legged_buzzard	Israel	PHL264751	2011
VII_j	VII.1.1	**VII.1.1**	VII.1.1	2293	MH377305.1	common_kestrel	Israel	PHL264758	2015
VII_j	VII.1.1	**VII.1.1**	VII.1.1	2294	MH377306.1	chicken	Israel	PHL246332	2016
VII_j	VII.1.1	**VII.1.1**	VII.1.1	2295	MH377307.1	chicken	Israel	PHL249275	2016
VII_j	VII.1.1	**VII.1.1**	VII.1.1	2296	MH377308.1	chicken	Israel	PHL251542	2016
VII_j	VII.1.1	**VII.1.1**	VII.1.1	2297	MH377309.1	turkey	Israel	PHL255913	2016
VII_d	VII.1.1	**VII.1.1**	VII.1.1	2298	MH377310.1		Israel	PHL104477	2010
VII_j	VII.1.1	**VII.1.1**	VII.1.1	2300	MH377312.1	chicken	Israel	PHL265941	2016
VII_j	VII.1.1	**VII.1.1**	VII.1.1	2301	MH377313.1	chicken	Israel	PHL265559	2016
VII_j	VII.1.1	**VII.1.1**	VII.1.1	2302	MH377314.1	chicken	Israel	PHL266018	2016

VII_j	VII.1.1	**VII.1.1**	VII.1.1	2303	MH377315.1	lesser_kestrel	Israel	PHL264759	2012
VII_j	VII.1.1	**VII.1.1**	VII.1.1	2305	MH377317.1	chicken	Israel	PHL142551	2012
VII_j	VII.1.1	**VII.1.1**	VII.1.1	2313	MH377325.1	chicken	Israel	PHL203308	2014
VII_j	VII.1.1	**VII.1.1**	VII.1.1	2439	MK005975	chicken	Egypt	Sohag_1_1012	2011
VII_j	VII.1.1	**VII.1.1**	VII.1.1	2440	MK005976	chicken	Egypt	Sohag_13_1015	2011
VII_j	VII.1.1	**VII.1.1**	VII.1.1	2443	MK005979	chicken	Egypt	Sohag_21_1023	2011
VII_j	VII.1.1	**VII.1.1**	VII.1.1	2444	MK005980	chicken	Egypt	Sohag_61_1027	2011
VII_j	VII.1.1	**VII.1.1**	VII.1.1	2445	MK005981	chicken	Egypt	Sohag_66_1030	2011
VII_j	VII.1.1	**VII.1.1**	VII.1.1	2446	MK005982	chicken	Egypt	Sohag_67_1031	2011
VII_j	VII.1.1	**VII.1.1**	VII.1.1	2447	MK005983	chicken	Egypt	Sohag_68_1032	2011
VII_j	VII.1.1	**VII.1.1**	VII.1.1	2449	MK005985	chicken	Egypt	Luxor_83_1035	2011
VII_j	VII.1.1	**VII.1.1**	VII.1.1	2450	MK005986	chicken	Egypt	Qena_101_1036	2012
VII_j	VII.1.1	**VII.1.1**	VII.1.1	2451	MK005987	chicken	Egypt	Qena_103_1037	2012
VII_j	VII.1.1	**VII.1.1**	VII.1.1	2452	MK005988	chicken	Egypt	Qena_105_1039	2012
VII_j	VII.1.1	**VII.1.1**	VII.1.1	2453	MK005989	chicken	Egypt	Qena_108_1041	2012
VII_j	VII.1.1	**VII.1.1**	VII.1.1	2455	MK005991	chicken	Egypt	Qena_140_1048	2012
VII_j	VII.1.1	**VII.1.1**	VII.1.1	2456	MK005992	chicken	Egypt	Qena_141_1049	2012
VII_j	VII.1.1	**VII.1.1**	VII.1.1	2457	MK005993	chicken	Egypt	Qena_144_1052	2012
VII_j	VII.1.1	**VII.1.1**	VII.1.1	2458	MK005995	pigeon	Egypt	Souqal_Cairo_19_CL_G3_1090	2015
VII_j	VII.1.1	**VII.1.1**	VII.1.1	2459	MK005996	pigeon	Egypt	Souqal_Cairo_27_OP_G9_1091	2015
VII_j	VII.1.1	**VII.1.1**	VII.1.1	2460	MK005997	pigeon	Egypt	Souqal_Cairo_28_OP_G27_1109	2015
VII_j	VII.1.1	**VII.1.1**	VII.1.1	2461	MK005998	pigeon	Egypt	Giza_15_CL_PG_1095	2015
VII_j	VII.1.1	**VII.1.1**	VII.1.1	2483	MG717683.1	quail	Egypt	SDU_2	2016
VII_j	VII.1.1	**VII.1.1**	VII.1.1	2484	MG717684.1	teal	Egypt	SDU_3	2016
VII_j	VII.1.1	**VII.1.1**	VII.1.1	2486	MG717686.1	teal	Egypt	SDU_4	2016
VII_b	VII.1.1	**VII.1.1**	VII.1.1	2487	MH445410.1	chicken	Egypt	18	2015
VII_l	VII.1.1	**VII.1.1**	VII.1.1	2490	MG519856.1		Iran	MSH_2	2015
VII_l	VII.1.1	**VII.1.1**	VII.1.1	2491	MG519857.1		Iran	MSH_3	2015
VII_l	VII.1.1	**VII.1.1**	VII.1.1	2497	MF417546.1	chicken	Iran	Beh	2011
VII_j	VII.1.1	**VII.1.1**	VII.1.1	2499	MH105251.1	chicken	China	Jiangsu_1816	2014
VII_d	VII.1.1	**VII.1.1**	VII.1.1	2500	MH105250.1	duck	China	Shandong_142	2015
VII_l	VII.1.1	**VII.1.1**	VII.1.1	2506	MH247187.1	chicken	Iran	MAM55	2017
VII_l	VII.1.1	**VII.1.1**	VII.1.1	2507	MH247186.1	chicken	Iran	MAM52	2017
VII_l	VII.1.1	**VII.1.1**	VII.1.1	2508	MH247185.1	chicken	Iran	MAM31	2017

VII_l	VII.1.1	**VII.1.1**	VII.1.1	2509	MH247184.1	chicken	Iran	MAM19	2017
VII_l	VII.1.1	**VII.1.1**	VII.1.1	2510	MH481363.1	chicken	Iran	MAM81	2018
VII_l	VII.1.1	**VII.1.1**	VII.1.1	2511	MH481362.1	chicken	Iran	MAM72	2018
VII_l	VII.1.1	**VII.1.1**	VII.1.1	2512	MH481361.1	chicken	Iran	MAM68	2017
VII_f	VII.1.2	**VII.1.2**	VII.1.2	1311	AF458010	chicken	China	JS_3	2000
VII_f	VII.1.2	**VII.1.2**	VII.1.2	1313	AY028995	fowl	China	A7	1996
VII_f	VII.1.2	**VII.1.2**	VII.1.2	1314	GQ338309	pigeon	China	18	2003
VII_f	VII.1.2	**VII.1.2**	VII.1.2	1315	GQ338310	pigeon	China	44	2003
VII_f	VII.1.2	**VII.1.2**	VII.1.2	1316	DQ227246	goose	China	Jiangsu_JS02	1999
VII_f	VII.1.2	**VII.1.2**	VII.1.2	1317	DQ227254	chicken	China	Broiler_Shandong_SWS_03	2003
VII_f	VII.1.2	**VII.1.2**	VII.1.2	1318	DQ858357	goose	China	Jiangsu_YG	2003
VII_a	VII.2	**VII.2**	VII.2	916	JN986837	chicken	Netherlands	152608_ancestral	1993
VII_h	VII.2	**VII.2**	VII.2	1329	HQ697255	chicken	Indonesia	Sukorejo_019	2010
VII_h	VII.2	**VII.2**	VII.2	1330	HQ697256	chicken	Indonesia	Makassar_003	2009
VII_h	VII.2	**VII.2**	VII.2	1331	HQ697261	chicken	Bali	bali_020_10	2010
VII_h	VII.2	**VII.2**	VII.2	1332	JX193074	egret	China	egret_Guangxi	2011
VII_h	VII.2	**VII.2**	VII.2	1333	KF026013	chicken	Malaysia	UPM_IBS_002	2011
VII_h	VII.2	**VII.2**	VII.2	1334	KR074404	chicken	Malaysia	IBS002_11	2011
VII_h	VII.2	**VII.2**	VII.2	1335	KR074405	chicken	Malaysia	IBS005_11	2011
VII_h	VII.2	**VII.2**	VII.2	1336	KR815908	turkey	South_Africa	N2057	2013
VII_h	VII.2	**VII.2**	VII.2	1337	KT760568	chicken	China	Guizhou_1032	2012
VII_h	VII.2	**VII.2**	VII.2	1338	KT760569	goose	China	Yunnan_1200	2013
VII_h	VII.2	**VII.2**	VII.2	1339	KU175230	chicken	China	Guizhou_1031	2012
VII_h	VII.2	**VII.2**	VII.2	1340	KU175232	chicken	China	Guizhou_1176	2012
VII_h	VII.2	**VII.2**	VII.2	1341	KU523524	chicken	Mozambique	466	2012
VII_h	VII.2	**VII.2**	VII.2	1342	KU523526	chicken	Mozambique	491	2012
VII_h	VII.2	**VII.2**	VII.2	1343	KU523528	chicken	Mozambique	658	2012
VII_h	VII.2	**VII.2**	VII.2	1344	KU523529	chicken	Mozambique	584	2013
VII_h	VII.2	**VII.2**	VII.2	1345	KU523531	chicken	Mozambique	494	2014
VII_h	VII.2	**VII.2**	VII.2	1346	KU523533	chicken	Mozambique	622	2014
VII_h	VII.2	**VII.2**	VII.2	1347	KX231366	chicken	Mozambique	1205	2011
VII_h	VII.2	**VII.2**	VII.2	1348	KX231368	chicken	Mozambique	192A	2016
VII_h	VII.2	**VII.2**	VII.2	2007	MF622034.1	chicken	South_Africa	32995	2015
VII_h	VII.2	**VII.2**	VII.2	2008	MF622035.1	chicken	South_Africa	230665	2013

VII_h	VII.2	**VII.2**	VII.2	2009	MF622036.1	chicken	Zimbabwe	235280	2013
VII_h	VII.2	**VII.2**	VII.2	2010	MF622037.1	chicken	South_Africa	239391	2013
VII_h	VII.2	**VII.2**	VII.2	2011	MF622038.1	chicken	Zambia	Chadiza	2015
VII_h	VII.2	**VII.2**	VII.2	2012	MF622039.1	chicken	South_Africa	H14973	2014
VII_h	VII.2	**VII.2**	VII.2	2013	MF622040.1	chicken	South_Africa	Inchanga	2013
VII_h	VII.2	**VII.2**	VII.2	2014	MF622041.1	chicken	Zambia	Katete	2015
VII_h	VII.2	**VII.2**	VII.2	2015	MF622042.1	chicken	Zambia	Mbeweka	2015
VII_h	VII.2	**VII.2**	VII.2	2016	MF622043.1	chicken	South_Africa	N2117	2015
VII_h	VII.2	**VII.2**	VII.2	2017	MF622044.1	chicken	South_Africa	N2683	2015
VII_h	VII.2	**VII.2**	VII.2	2018	MF622045.1	chicken	South_Africa	RBNW_1	2013
VII_h	VII.2	**VII.2**	VII.2	2020	MF622047.1	chicken	South_Africa	RBWW_3	2013
VII_i	VII.2	**VII.2**	VII.2	1403	HQ697254	chicken	Indonesia	Banjarmasin_010	2010
VII_i	VII.2	**VII.2**	VII.2	1404	HQ697257	chicken	Indonesia	Gianyar_013	2010
VII_i	VII.2	**VII.2**	VII.2	1407	KF113339	chicken	Pakistan	Lahore_30	2011
VII_i	VII.2	**VII.2**	VII.2	1408	KF113341	chicken	Pakistan	Lahore_43	2011
VII_i	VII.2	**VII.2**	VII.2	1409	KF113342	chicken	Pakistan	Lahore_50	2011
VII_i	VII.2	**VII.2**	VII.2	1410	KF113343	chicken	Pakistan	Gujranwala_56	2011
VII_i	VII.2	**VII.2**	VII.2	1411	KF113345	chicken	Pakistan	Khyber_Pukhtun_Khawa_117	2011
VII_i	VII.2	**VII.2**	VII.2	1412	KF113349	chicken	Pakistan	Kasure_191	2012
VII_i	VII.2	**VII.2**	VII.2	1413	KF113350	chicken	Pakistan	Lahore_200	2012
VII_i	VII.2	**VII.2**	VII.2	1414	KF113351	chicken	Pakistan	University_Vet_Animal_Sci_211	2012
VII_i	VII.2	**VII.2**	VII.2	1415	KF792019	chicken	Israel	KY50_826	2013
VII_i	VII.2	**VII.2**	VII.2	1416	KF792021	chicken	Israel	BT_120_827	2013
VII_i	VII.2	**VII.2**	VII.2	1417	KM670337	chicken	Pakistan	SFR_611_13	2013
VII_i	VII.2	**VII.2**	VII.2	1418	KP719224	chicken	Libya	13VIR_7225_1	2013
VII_i	VII.2	**VII.2**	VII.2	1419	KP776462	chicken	Pakistan	AW_14	2014
VII_i	VII.2	**VII.2**	VII.2	1420	KP780878	chicken	Pakistan	Gujranwala_649	2013
VII_i	VII.2	**VII.2**	VII.2	1423	KR676389	chicken	Pakistan	Kohat_122	2011
VII_i	VII.2	**VII.2**	VII.2	1424	KR676391	chicken	Pakistan	Narowal_329	2013
VII_i	VII.2	**VII.2**	VII.2	1425	KR676392	chicken	Pakistan	Waziabbad_431	2013
VII_i	VII.2	**VII.2**	VII.2	1426	KR676396	chicken	Pakistan	Lahore_736	2014
VII_i	VII.2	**VII.2**	VII.2	1427	KR676397	chicken	Pakistan	Attock_7_411	2014
VII_i	VII.2	**VII.2**	VII.2	1428	KR676398	chicken	Pakistan	Multan_5_125	2014
VII_i	VII.2	**VII.2**	VII.2	1429	KR676402	chicken	Pakistan	Lahore_12	2015

VII_i	VII.2	**VII.2**	VII.2	1430	KR676403	chicken	Pakistan	Lahore_24	2015
VII_i	VII.2	**VII.2**	VII.2	1431	KR676404	chicken	Pakistan	Gujranwala_2	2015
VII_i	VII.2	**VII.2**	VII.2	1434	KU862287	Peacock	Pakistan	Lahore_AW_7	2013
VII_i	VII.2	**VII.2**	VII.2	1435	KU862288	Peacock	Pakistan	Lahore_AW_3	2014
VII_i	VII.2	**VII.2**	VII.2	1436	KU862291	Peacock	Pakistan	Pathoki_AW_1	2015
VII_i	VII.2	**VII.2**	VII.2	1437	KU862292	Peacock	Pakistan	Kamoki_AW_2	2015
VII_i	VII.2	**VII.2**	VII.2	1438	KU862293	Parakeet	Pakistan	Karachi_AW_1	2014
VII_i	VII.2	**VII.2**	VII.2	1439	KU862294	Parakeet	Pakistan	Lahore_AW_2	2014
VII_i	VII.2	**VII.2**	VII.2	1440	KU862295	Parakeet	Pakistan	Karachi_AW_3	2015
VII_i	VII.2	**VII.2**	VII.2	1441	KU862296	Black_Swan	Pakistan	Lahore_AW_1	2015
VII_i	VII.2	**VII.2**	VII.2	1442	KX268689	parrot	Pakistan	Lahore_SFR_129	2015
VII_i	VII.2	**VII.2**	VII.2	1443	KX496962	wild_pigeon	Pakistan	Lahore_20A_996	2015
VII_i	VII.2	**VII.2**	VII.2	1444	KX496963	pigeon	Pakistan	Lahore_22A_1001	2015
VII_i	VII.2	**VII.2**	VII.2	1445	KX496964	pigeon	Pakistan	Lahore_23A_997	2015
VII_i	VII.2	**VII.2**	VII.2	1446	KX496967	pigeon	Pakistan	Lahore_1085	2015
VII_i	VII.2	**VII.2**	VII.2	1447	KX791183	parakeet	Pakistan	R_Pindi_SFR_16	2016
VII_i	VII.2	**VII.2**	VII.2	1448	KX791184	backyard	Pakistan	Lahore_SFR_144A	2016
VII_i	VII.2	**VII.2**	VII.2	1449	KX791185	backyard	Pakistan	Lahore_SFR_144B	2016
VII_i	VII.2	**VII.2**	VII.2	1450	KX791186	backyard	Pakistan	Lahore_SFR_144C	2016
VII_i	VII.2	**VII.2**	VII.2	1451	KX791187	backyard	Pakistan	Lahore_SFR_144D	2016
VII_i	VII.2	**VII.2**	VII.2	1452	KY076031	chicken	Pakistan	Wazirabad_15A_995	2015
VII_i	VII.2	**VII.2**	VII.2	1453	KY076032	chicken	Pakistan	Kasur_26A_998	2011
VII_i	VII.2	**VII.2**	VII.2	1454	KY076035	chicken	Pakistan	Pattoki_1A_1002	2015
VII_i	VII.2	**VII.2**	VII.2	1455	KY076039	chicken	Pakistan	Gujranwala_13A_1009	2015
VII_k	VII.2	**VII.2**	VII.2	1604	KY747479.1	chicken	Namibia	5620	2016
VII_k	VII.2	**VII.2**	VII.2	1605	KY747480.1	chicken	Namibia	6195	2016
VII_k	VII.2	**VII.2**	VII.2	1606	KY747481.1	chicken	Namibia	6196	2016
VII_k	VII.2	**VII.2**	VII.2	1607	KY747482.1	chicken	Namibia	6403	2016
VII_k	VII.2	**VII.2**	VII.2	1608	KY747483.1	chicken	Namibia	6762	2016
VII_i	VII.2	**VII.2**	VII.2	2053	KY967611.1	mallard	Pakistan	I_UVAS	2016
VII_i	VII.2	**VII.2**	VII.2	2084	MF437287.1	Anas_carolinenses	Pakistan	II_UVAS	2015
VII_i	VII.2	**VII.2**	VII.2	2098	MG200021	chicken	Pakistan	Lahore_965	2015
VII_i	VII.2	**VII.2**	VII.2	2099	MG200022	chicken	Pakistan	Kassur_966	2015
VII_i	VII.2	**VII.2**	VII.2	2101	MG200024	chicken	Pakistan	Sheikhupura_969	2015

VII_i	VII.2	**VII.2**	VII.2	2102	MG200025	chicken	Pakistan	Lahore_972	2015
VII_i	VII.2	**VII.2**	VII.2	2103	MG200026	chicken	Pakistan	Gujranwala_978	2015
VII_i	VII.2	**VII.2**	VII.2	2113	MG686589.1	goose	Pakistan	SKHP_AW_101	2015
VII_i	VII.2	**VII.2**	VII.2	2114	MG686590.1	duck	Pakistan	GUJR_AW_51	2015
VII_i	VII.2	**VII.2**	VII.2	2115	MG686591.1	duck	Pakistan	GUJR_AW_52	2015
VII_i	VII.2	**VII.2**	VII.2	2116	MG686592.1	duck	Pakistan	ISLM_AW_1	2015
VII_i	VII.2	**VII.2**	VII.2	2117	MG686593.1	goose	Pakistan	SLK_AW_16	2015
VII_i	VII.2	**VII.2**	VII.2	2118	MG686594.1	goose	Pakistan	SKHP_AW_102	2015
VII_i	VII.2	**VII.2**	VII.2	2119	MG686595.1	goose	Pakistan	SKHP_AW_103	2015
VII_i	VII.2	**VII.2**	VII.2	2120	MG686596.1	goose	Pakistan	SKHP_AW_104	2015
VII_i	VII.2	**VII.2**	VII.2	2121	MG686597.1	duck	Pakistan	GUJR_AW_53	2015
VII_i	VII.2	**VII.2**	VII.2	2122	MG686598.1	goose	Pakistan	GUJR_AW_55	2015
VII_i	VII.2	**VII.2**	VII.2	2123	MG686599.1	goose	Pakistan	GUJR_AW_54	2015
VII_i	VII.2	**VII.2**	VII.2	2124	MG686600.1	duck	Pakistan	GUJR_AW_56	2015
VII_i	VII.2	**VII.2**	VII.2	2125	MG686601.1	duck	Pakistan	Lahore_AW_4	2017
VII_i	VII.2	**VII.2**	VII.2	2127	MG686603.1	duck	Pakistan	KAR_AW_2	2014
VII_i	VII.2	**VII.2**	VII.2	2128	MG686604.1	duck	Pakistan	Lahore_AW_6	2016
VII_i	VII.2	**VII.2**	VII.2	2129	MG686605.1	duck	Pakistan	SLK_AW_15	2016
VII_i	VII.2	**VII.2**	VII.2	2130	MG686606.1	duck	Pakistan	SLK_AW_14	2016
VII_i	VII.2	**VII.2**	VII.2	2132	MG686608.1	black_swan	Pakistan	Lahore_AW_8	2016
VII_i	VII.2	**VII.2**	VII.2	2133	MG686609.1	goose	Pakistan	Lahore_AW_3	2017
VII_h	VII.2	**VII.2**	VII.2	2140	MG869268.1	chicken	Vietnam	15A1	2015
VII_h	VII.2	**VII.2**	VII.2	2141	MG869269.1	chicken	Vietnam	LC15	2015
VII_i	VII.2	**VII.2**	VII.2	2145	MG871466.1	chicken	Iran	PCR_UT	2017
VII_i	VII.2	**VII.2**	VII.2	2152	MH120424.1	chicken	Pakistan	NIAB	2017
VII_i	VII.2	**VII.2**	VII.2	2167	MH371036.1	turkey	Israel	PHL138692	2011
VII_i	VII.2	**VII.2**	VII.2	2168	MH371037.1	chicken	Israel	PHL137592	2011
VII_i	VII.2	**VII.2**	VII.2	2181	MH371050.1	chicken	Israel	PHL168757	2013
VII_i	VII.2	**VII.2**	VII.2	2197	MH371066.1	chicken	Israel	PHL149165	2012
VII_i	VII.2	**VII.2**	VII.2	2201	MH371070.1	chicken	Israel	PHL159057	2012
VII_i	VII.2	**VII.2**	VII.2	2203	MH371072.1	chicken	Israel	PHL175571	2013
VII_i	VII.2	**VII.2**	VII.2	2228	MH371097.1	chicken	Israel	PHL140339	2011
VII_i	VII.2	**VII.2**	VII.2	2251	MH377263.1	turkey	Israel	PHL143483	2012
VII_i	VII.2	**VII.2**	VII.2	2254	MH377266.1	chicken	Israel	PHL167788	2012

VII_i	VII.2	**VII.2**	VII.2	2255	MH377267.1	chicken	Israel	PHL169208	2013
VII_i	VII.2	**VII.2**	VII.2	2277	MH377289.1	chicken	Israel	PHL174621	2013
VII_i	VII.2	**VII.2**	VII.2	2278	MH377290.1	turkey	Israel	PHL176533	2013
VII_i	VII.2	**VII.2**	VII.2	2291	MH377303.1	common_kestrel	Israel	PHL264753	2012
VII_i	VII.2	**VII.2**	VII.2	2304	MH377316.1	chicken	Israel	PHL141759	2011
VII_i	VII.2	**VII.2**	VII.2	2306	MH377318.1	chicken	Israel	PHL142626	2012
VII_i	VII.2	**VII.2**	VII.2	2307	MH377319.1	turkey	Israel	PHL142784	2012
VII_i	VII.2	**VII.2**	VII.2	2308	MH377320.1	chicken	Israel	PHL143114	2012
VII_i	VII.2	**VII.2**	VII.2	2309	MH377321.1	chicken	Israel	PHL143696	2012
VII_i	VII.2	**VII.2**	VII.2	2311	MH377323.1	chicken	Israel	PHL147004	2012
VII_i	VII.2	**VII.2**	VII.2	2331	MH432252.1	bassette_chicken	Belgium	4096	2018
VII_i	VII.2	**VII.2**	VII.2	2332	MH614933.1	chicken	Jordan	J11_Spleen	2018
VII_i	VII.2	**VII.2**	VII.2	2334	MH717055.1	chicken	Pakistan	AJK_AW_c21	2017
VII_i	VII.2	**VII.2**	VII.2	2335	MH717056.1	chicken	Pakistan	AJK_AW_c22	2017
VII_i	VII.2	**VII.2**	VII.2	2336	MH717057.1	chicken	Pakistan	AJK_AW_c23	2017
VII_i	VII.2	**VII.2**	VII.2	2337	MH717058.1	chicken	Pakistan	AJK_AW_c24	2017
VII_i	VII.2	**VII.2**	VII.2	2338	MH717059.1	chicken	Pakistan	AJK_AW_c25	2018
VII_i	VII.2	**VII.2**	VII.2	2339	MH717060.1	chicken	Pakistan	AJK_AW_c26	2018
VII_i	VII.2	**VII.2**	VII.2	2340	MH717061.1	chicken	Pakistan	AJK_AW_by31	2017
VII_i	VII.2	**VII.2**	VII.2	2341	MH717062.1	chicken	Pakistan	AJK_AW_by32	2017
VII_i	VII.2	**VII.2**	VII.2	2342	MH717063.1	chicken	Pakistan	AJK_AW_by33	2017
VII_i	VII.2	**VII.2**	VII.2	2343	MH717064.1	chicken	Pakistan	AJK_AW_by34	2018
VII_i	VII.2	**VII.2**	VII.2	2344	MH717065.1	chicken	Pakistan	AJK_AW_by35	2018
VII_i	VII.2	**VII.2**	VII.2	2345	MH717066.1	duck	Pakistan	AJK_AW_d41	2018
VII_i	VII.2	**VII.2**	VII.2	2346	MH717067.1	duck	Pakistan	AJK_AW_d42	2018
VII_i	VII.2	**VII.2**	VII.2	2347	MH717068.1	duck	Pakistan	AJK_AW_d43	2018
VII_i	VII.2	**VII.2**	VII.2	2348	MH717069.1	peacock	Pakistan	AJK_AW_pc45	2018
VII_i	VII.2	**VII.2**	VII.2	2463	MK006009	chicken	Pakistan	Sargohda_9CCS	2015
VII_i	VII.2	**VII.2**	VII.2	2464	MK006010	chicken	Pakistan	Sargohda_10CCS	2015
VII_i	VII.2	**VII.2**	VII.2	2465	MK006011	chicken	Pakistan	MirpurKhas_3EOS	2015
VII_i	VII.2	**VII.2**	VII.2	2468	MK006014	chicken	Pakistan	BankaCheema_PF_1H	2015
VII_i	VII.2	**VII.2**	VII.2	2470	MK006016	chicken	Pakistan	BankaCheema_2HOS	2015
VII_i	VII.2	**VII.2**	VII.2	2471	MK006017	chicken	Pakistan	WadanaKasur_1FOS	2015
VII_h	VII.2	**VII.2**	VII.2	2501	MH105249.1	duck	China	Yunnan	2017

VII_h	VII.2	**VII.2**	VII.2	2502	MH105247.1	chicken	China	Yunnan_1113	2017
VII_i	VII.2	**VII.2**	VII.2	2503	MK069429.1	chicken	China	Kulonprogo_4171317	2017
VII_i	VII.2	**VII.2**	VII.2	2504	MK069428.1	chicken	China	Belitung_3150041	2015
VII_i	VII.2	**VII.2**	VII.2	1422	KR074407	chicken	Malaysia	MB128_04	2004
VIII	VIII	**VIII**	VIII	1623	AF048763	chicken	Malaysia	AF2240	1960
VIII	VIII	**VIII**	VIII	1624	AY734534	chicken	Argentina	Trenque_Lauquen	1970
VIII	VIII	**VIII**	VIII	1625	FJ751918	chicken	China	QH1	1979
VIII	VIII	**VIII**	VIII	1626	FJ751919	chicken	China	QH4	1985
VIII	VIII	**VIII**	VIII	1627	JX012096	_	Malaysia	AF2240_I	1960s
VIII	VIII	**VIII**	VIII	2505	MH715892.1	game_fowl	China	GXGB	2011
X	X	**X**	X	1672	AY727881	duck	Argentina	32C_T_98	1998
X	X	**X**	X	1673	AY727882	swan	Argentina	126C_00	2000
X_a	X	**X**	X	1677	EF564826	northern_pintail	USA	US_OH_87_486	1987
X_a	X	**X**	X	1678	FJ705468	mottled_duck	USA	US_TX_TX01_130	2001
X_a	X	**X**	X	1679	JN872171	turkey	USA	Minnesota_17531_3_	2010
X_a	X	**X**	X	1680	KX857713	Mallard	MN_USA	AI09_1832	2009
X_a	X	**X**	X	1681	KX857714	Blue_winged_teal	ND_USA	AI09_2902	2009
X_a	X	**X**	X	1682	KX857715	Blue_winged_teal	ND_USA	AI09_3678	2009
X_a	X	**X**	X	1683	KX857716	Redhead	ND_USA	AI09_4117	2009
X_a	X	**X**	X	1684	KX857717	Mallard	MN_USA	AI13_4156	2013
X_b	X	**X**	X	1685	EF564832	mallard	USA	US_OH_86_233	1986
X_b	X	**X**	X	1686	FJ705464	mallard	USA	US_OH_04_411	2004
X_b	X	**X**	X	1687	FJ705465	mallard	USA	US_MN_03_632	2003
X_b	X	**X**	X	1688	FJ705466	mallard	USA	US_MN_99_376	1999
X_b	X	**X**	X	1689	FJ705467	mallard	USA	US_MN_MN00_32	2000
X_b	X	**X**	X	1690	FJ705469	mallard	USA	US_MN_MN00_39	2000
X_b	X	**X**	X	1691	KX857718	American_black_duck	MN_USA	AI09_3836	2009
X_b	X	**X**	X	1692	KX857719	Mallard	MN_USA	AI10_2806	2010
X_b	X	**X**	X	1693	KX857720	Mallard	MN_USA	AI10_2962	2010
X_b	X	**X**	X	1694	KX857721	Mallard	MN_USA	AI10_3434	2010
X_b	X	**X**	X	1696	KX857723	Blue_winged_teal	MN_USA	AI08_4957	2008
X_b	X	**X**	X	2323	MH392221	mallard	USA_MN_	99_376_163	1999
XI	XI	**XI**	XI	1697	HQ266602	chicken	Madagascar	MG_725	2008
XI	XI	**XI**	XI	1698	HQ266603	chicken	Madagascar	MG_1992	2008

XI	XI	**XI**	XI	1699	HQ266604	chicken	Madagascar	MG_Meola	2008
XI	XI	**XI**	XI	1700	HQ266605	chicken	Madagascar	MG_39_4	2008
XI	XI	**XI**	XI	1701	JX518875	chicken	Madagascar	MGBBS_	2009
XI	XI	**XI**	XI	1702	JX518876	chicken	Madagascar	MGF003C_	2010
XI	XI	**XI**	XI	1703	JX518877	chicken	Madagascar	MGF015C_	2011
XI	XI	**XI**	XI	1704	JX518878	chicken	Madagascar	MGF082T_	2010
XI	XI	**XI**	XI	1705	JX518879	chicken	Madagascar	MGF120T_	2010
XI	XI	**XI**	XI	1706	JX518880	duck	Madagascar	MGF166_	2010
XI	XI	**XI**	XI	1707	JX518881	chicken	Madagascar	MGF192C_	2010
XI	XI	**XI**	XI	1708	JX518882	chicken	Madagascar	MGMNJ_	2009
XI	XI	**XI**	XI	1709	JX518883	chicken	Madagascar	MGS1130T_	2011
XI	XI	**XI**	XI	1710	JX518884	chicken	Madagascar	MGS1595T_	2011
XII_d	XII	**XII**	XII	2142	MG869270.1	chicken	Vietnam	NDVQG	2008
XII_d	XII	**XII**	XII	2143	MG869271.1	chicken	Vietnam	NCXKH	2011
XII_d	XII	**XII**	XII	2144	MG869272.1	chicken	Vietnam	NCXMT	2014
XII_a	XII.1	**XII.1**	XII.1	1712	JN800306	chicken	Peru	1918_03	2008
XII_a	XII.1	**XII.1**	XII.1	1713	KJ865695	chicken	Colombia	1326_13285	2009
XII_a	XII.1	**XII.1**	XII.1	1714	KJ865696	chicken	Colombia	1326_13286	2009
XII_a	XII.1	**XII.1**	XII.1	1715	KR732614	peacock	Peru		2011
XII_a	XII.1	**XII.1**	XII.1	1716	KU594613	gamecock	Peru	Lima_40931	2004
XII_a	XII.1	**XII.1**	XII.1	1717	KU594614	gamecock	Peru	Lima_40785	2004
XII_a	XII.1	**XII.1**	XII.1	1718	KU594615	chicken	Peru	Apurimac_50009	2005
XII_a	XII.1	**XII.1**	XII.1	1719	KU594616	gamecock	Peru	Lurin_40871	2004
XII_a	XII.1	**XII.1**	XII.1	1720	KU594617	chicken	Peru	Piura_60087	2006
XII_a	XII.1	**XII.1**	XII.1	1721	KU594618	chicken	Peru	Arequipa_VFAR_81	2015
XII_b	XII.2	**XII.2**	XII.2	1724	JN627504	goose	China	GD_12	2011
XII_b	XII.2	**XII.2**	XII.2	1725	JN627507	goose	China	GD_1003	2010
XII_b	XII.2	**XII.2**	XII.2	1726	JN627508	goose	China	GD_450	2011
XII_b	XII.2	**XII.2**	XII.2	1727	KC551967	goose	China	Guangdong	2010
XII_b	XII.2	**XII.2**	XII.2	1997	MF278926.1	goose	China	FS_GM_4	2011
XII_b	XII.2	**XII.2**	XII.2	1998	MF278927.1	goose	China	FS_SS_292	2013
XII_b	XII.2	**XII.2**	XII.2	1999	MF278928.1	goose	China	ZQ_JL_12	2011
XII_b	XII.2	**XII.2**	XII.2	2000	MF278929.1	goose	China	ZQ_XG_17	2011
XII_b	XII.2	**XII.2**	XII.2	2001	MF278930.1	goose	China	ZQ_XG_502	2014

XII_b	XII.2	**XII.2**	XII.2	2003	MF278933.1	muscovy_duck	China	MM_GZ_884	2016
XIII	XIII.1	**XIII.1**	XIII.1	1728	JN942041	cockatoo	India	7847_ancestral	1982
XIII_a	XIII.1.1	**XIII.1.2**	XIII.1.1	1730	AY865652	Sterna_albifrons	Russia	Astr_2755	2001
XIII_a	XIII.1.1	**XIII.1.2**	XIII.1.1	1733	GU585905	chicken	Sweden		1997
XIII_a	XIII.1.1	**XIII.1.2**	XIII.1.1	1734	HQ589257	chicken	India	Bareilly	1997
XIII_a	XIII.1.1	**XIII.1.2**	XIII.1.1	1738	KF727980	chicken	India	Bareilly	2006
XIII_a	XIII.1.1	**XIII.1.2**	XIII.1.1	1739	KJ577585	chicken	India	Bareilly_01	2010
XIII_a	XIII.1.1	**XIII.1.1**	XIII.1.1	1731	FJ772491	chicken	Burundi	4132_12	2008
XIII_a	XIII.1.1	**XIII.1.1**	XIII.1.1	1732	FJ772494	chicken	Burundi	4132_20	2008
XIII_a	XIII.1.1	**XIII.1.1**	XIII.1.1	1735	JN942034	ostrich	South_Africa	45445_3	1995
XIII_a	XIII.1.1	**XIII.1.1**	XIII.1.1	1736	JN942043	roller	Tanzania	47385_11	2010
XIII_a	XIII.1.1	**XIII.1.1**	XIII.1.1	1737	JN942044	roller	Tanzania	47387_6	2010
XIII_a	XIII.1.1	**XIII.1.1**	XIII.1.1	1740	MF409241.1	chicken	Zambia	Chiwoko	2015
XIII_a	XIII.1.2.1	**XIII.1.2**	XIII.1.2	1778	JQ267579	chicken	Iran	EMM_7	2011
XIII_a	XIII.1.2.1	**XIII.1.2**	XIII.1.2	1779	JQ267580	chicken	Iran	EMM_6	2011
XIII_a	XIII.1.2.1	**XIII.1.2**	XIII.1.2	1780	JQ267581	chicken	Iran	EMM_5	2011
XIII_a	XIII.1.2.1	**XIII.1.2**	XIII.1.2	1781	JQ267582	chicken	Iran	EMM_4	2010
XIII_a	XIII.1.2.1	**XIII.1.2**	XIII.1.2	1782	JQ267583	chicken	Iran	EMM_3	2009
XIII_a	XIII.1.2.1	**XIII.1.2**	XIII.1.2	1783	JQ267584	chicken	Iran	EMM_2	2008
XIII_a	XIII.1.2.1	**XIII.1.2**	XIII.1.2	1784	JQ267585	chicken	Iran	EMM_1	2008
XIII_b	XIII.1	**XIII.2**	XIII.2	1729	KT734766	chicken	India	Pandu	2015
XIII_b	XIII.1	**XIII.2**	XIII.2	2022	KY828161.1	chicken	India	MZ_7	2016
XIII_b	XIII.1	**XIII.2**	XIII.2	2023	KY828160.1	chicken	India	MZ_6	2016
XIII_b	XIII.1	**XIII.2**	XIII.2	2024	KY828159.1	chicken	India	MZ_5	2016
XIII_b	XIII.1	**XIII.2**	XIII.2	2025	KY828158.1	chicken	India	MZ_4	2016
XIII_b	XIII.1	**XIII.2**	XIII.2	2026	KY828157.1	chicken	India	MZ_3	2016
XIII_b	XIII.1	**XIII.2**	XIII.2	2027	KY828156.1	chicken	India	MZ_2	2016
XIII_b	XIII.1	**XIII.2**	XIII.2	2028	KY828155.1	chicken	India	MZ_1	2016
XIII_b	XIII.1	**XIII.2**	XIII.2	2076	MF422123.1	chicken	India	110_13A	2013
XIII_b	XIII.1.2.2.2	**XIII.2.1**	XIII.2.1	1745	GU182323	chicken	Pakistan	SPVC_Karachi_43	2008
XIII_b	XIII.1.2.2.1	**XIII.2.1**	XIII.2.1	1747	GU182331	chicken	Pakistan	SPVC_Karachi_33	2007
XIII_b	XIII.1.2.2.1	**XIII.2.1**	XIII.2.1	1748	JN682184	chicken	Pakistan	BYP_Lahore	2010
XIII_b	XIII.1.2.2.1	**XIII.2.1**	XIII.2.1	1749	JN682186	chicken	Pakistan	CP_Islamabad1	2010
XIII_b	XIII.1.2.2.1	**XIII.2.1**	XIII.2.1	1750	JN682187	chicken	Pakistan	BYP_Rawalpindi	2010
XIII_b	XIII.1.2.2.1	**XIII.2.1**	XIII.2.1	1752	JN682189	chicken	Pakistan	CP_Rawalpindi2	2010
XIII_b	XIII.1.2.2.1	**XIII.2.1**	XIII.2.1	1753	JN682190	chicken	Pakistan	CP_Islamabad2	2010
XIII_b	XIII.1.2.2.1	**XIII.2.1**	XIII.2.1	1754	JN682191	chicken	Pakistan	CP_Islamabad3	2010

XIII_b	XIII.1.2.2.1	**XIII.2.1**	XIII.2.1	1755	JN682211	chicken	Pakistan	CP	2010
XIII_b	XIII.1.2.2.1	**XIII.2.1**	XIII.2.1	1756	JQ517285	chicken	Pakistan	Pakistan_UDL8	2011
XIII_b	XIII.1.2.2.1	**XIII.2.1**	XIII.2.1	1757	JX436344	chicken	Pakistan	MM14_Lahore	2010
XIII_b	XIII.1.2.2.1	**XIII.2.1**	XIII.2.1	1758	KF113338	chicken	Pakistan	University_Diagnostic_Lab_12	2010
XIII_b	XIII.1.2.2.1	**XIII.2.1**	XIII.2.1	2050	MH019281.1	chicken	Pakistan	PK1	2015
XIII_b	XIII.1.2.2.1	**XIII.2.1**	XIII.2.1	2324	MH392222	chicken	Pakistan	SPVC_Karachi_27_558	2007
XIII_b	XIII.1.2.2.1	**XIII.2.1**	XIII.2.1	2325	MH392223	chicken	Pakistan	SPVC_Karachi_33_556	2007
XIII_b	XIII.1.2.2.2	**XIII.2.2**	XIII.2.2	1760	KF740478	Japanese_quail	India	NDV2K35_TN	2003
XIII_b	XIII.1.2.2.2	**XIII.2.2**	XIII.2.2	1761	KM056344	chicken	India	ndv16_Godhra_03	2013
XIII_b	XIII.1.2.2.2	**XIII.2.2**	XIII.2.2	1762	KM056345	chicken	India	ndv20_Navli_03	2013
XIII_b	XIII.1.2.2.2	**XIII.2.2**	XIII.2.2	1763	KM056346	chicken	India	ndv26_navli_03	2013
XIII_b	XIII.1.2.2.2	**XIII.2.2**	XIII.2.2	1764	KM056347	chicken	India	ndv32_vaherakhadi_04	2013
XIII_b	XIII.1.2.2.2	**XIII.2.2**	XIII.2.2	1765	KM056348	chicken	India	ndv40_sarsa_04	2013
XIII_b	XIII.1.2.2.2	**XIII.2.2**	XIII.2.2	1766	KM056349	chicken	India	ndv42_gopalpura_04	2013
XIII_b	XIII.1.2.2.2	**XIII.2.2**	XIII.2.2	1767	KM056350	chicken	India	ndv52_Sarsa	_
XIII_b	XIII.1.2.2.2	**XIII.2.2**	XIII.2.2	1768	KM056352	chicken	India	ndv54_Hyderabad	_
XIII_b	XIII.1.2.2.2	**XIII.2.2**	XIII.2.2	1769	KP089979	chicken	India	Nagpur	2012
XIII_b	XIII.1.2.2.2	**XIII.2.2**	XIII.2.2	1770	KT734767	chicken	India	Polashbari	2014
XIII_b	XIII.1.2.2.2	**XIII.2.2**	XIII.2.2	1771	KX061544	Red_lori	India	CHN	2013
XIII_b	XIII.1.2.2.2	**XIII.2.2**	XIII.2.2	1772	KX242342	chicken	India	D162	2013
XIII_b	XIII.1.2.2.2	**XIII.2.2**	XIII.2.2	1773	KX372707	chicken	India	Nagpur_03	2011
XIII_b	XIII.1.2.2.2	**XIII.2.2**	XIII.2.2	1774	KX372708	chicken	India	Nagpur_04	2011
XIII_b	XIII.1.2.2.2	**XIII.2.2**	XIII.2.2	1785	KT734765	chicken	India	Hajo	2014
XIII_b	XIII.1.2.2.2	**XIII.2.2**	XIII.2.2	2070	MF362983.1	chicken	India	PDDSL_2	2015
XIII_b	XIII.1.2.2.2	**XIII.2.2**	XIII.2.2	2071	MF362984.1	chicken	India	PDDSL_3	2015
XIII_b	XIII.1.2.2.2	**XIII.2.2**	XIII.2.2	2072	MF362985.1	chicken	India	PDDSL_4	2015
XIII_b	XIII.1.2.2.2	**XIII.2.2**	XIII.2.2	2073	MF362986.1	chicken	India	PDDSL_5	2015
XIII_b	XIII.1.2.2.2	**XIII.2.2**	XIII.2.2	2075	MF362988.1	chicken	India	PDDSL_7	2016
XIII_b	XIII.1.2.2.2	**XIII.2.2**	XIII.2.2	2077	MF422124.1	chicken	India	229_13B	2013
XIII_b	XIII.1.2.2.2	**XIII.2.2**	XIII.2.2	2078	MF422125.1	chicken	India	96_15	2015
XIII_b	XIII.1.2.2.2	**XIII.2.2**	XIII.2.2	2079	MF422126.1	chicken	India	478_13A	2013
XIII_b	XIII.1.2.2.2	**XIII.2.2**	XIII.2.2	2080	MF422127.1	chicken	India	805_13B	2013
XIII_b	XIII.1.2.2.2	**XIII.2.2**	XIII.2.2	2081	MF422128.1	chicken	India	248_13A2	2013
XIII_b	XIII.1.2.2.2	**XIII.2.2**	XIII.2.2	2082	MF422129.1	chicken	India	410_16A	2016
XIV	XIV	**XIV**	XIV	1786	JF966386	chicken	Mali	ML029	2007
XIV_a	XIV.1	**XIV.1**	XIV.1	1790	FJ772452	chicken	Nigeria	1377_8	2006
XIV_a	XIV.1	**XIV.1**	XIV.1	1791	HF969131	chicken	Nigeria	NIE08_2117	2009
XIV_a	XIV.1	**XIV.1**	XIV.1	1792	HF969136	chicken	Nigeria	NIE08_2194	2009
XIV_a	XIV.1	**XIV.1**	XIV.1	1793	HF969139	chicken	Nigeria	NIE08_2280	2009
XIV_a	XIV.1	**XIV.1**	XIV.1	1794	HF969144	chicken	Nigeria	NIE09_2009	2009
XIV_a	XIV.1	**XIV.1**	XIV.1	1795	HF969150	chicken	Nigeria	NIE09_2044	2009

XIV_a	XIV.1	**XIV.1**	XIV.1	1796	HF969155	chicken	Nigeria	NIE09_2087	2009
XIV_a	XIV.1	**XIV.1**	XIV.1	1797	HF969167	turkey	Nigeria	NIE10_082	2011
XIV_a	XIV.1	**XIV.1**	XIV.1	1798	HF969186	chicken	Nigeria	NIE07_125	2007
XIV_a	XIV.1	**XIV.1**	XIV.1	1799	HF969193	chicken	Nigeria	NIE08_2150	2009
XIV_a	XIV.1	**XIV.1**	XIV.1	1800	HF969200	chicken	Nigeria	NIE08_2362	2009
XIV_a	XIV.1	**XIV.1**	XIV.1	1801	HF969205	turkey	Nigeria	NIE09_2071	2009
XIV_a	XIV.1	**XIV.1**	XIV.1	1802	JN872165	chicken	Niger	VIR_1377_7_	2006
XIV_a	XIV.1	**XIV.1**	XIV.1	1803	JQ039386	chicken	Nigeria	VRD08_36	2008
XIV_b	XIV.2	**XIV.2**	XIV.2	1807	HF969133	chicken	Nigeria	NIE08_2159	2009
XIV_b	XIV.2	**XIV.2**	XIV.2	1808	HF969141	chicken	Nigeria	NIE08_2359	2009
XIV_b	XIV.2	**XIV.2**	XIV.2	1809	HF969142	chicken	Nigeria	NIE09_1596	2009
XIV_b	XIV.2	**XIV.2**	XIV.2	1810	HF969143	chicken	Nigeria	NIE09_1597	2009
XIV_b	XIV.2	**XIV.2**	XIV.2	1811	HF969145	chicken	Nigeria	NIE09_2014	2009
XIV_b	XIV.2	**XIV.2**	XIV.2	1812	HF969146	chicken	Nigeria	NIE09_2017	2009
XIV_b	XIV.2	**XIV.2**	XIV.2	1813	HF969149	chicken	Nigeria	NIE09_2041	2009
XIV_b	XIV.2	**XIV.2**	XIV.2	1814	HF969151	chicken	Nigeria	NIE09_2053	2009
XIV_b	XIV.2	**XIV.2**	XIV.2	1815	HF969157	chicken	Nigeria	NIE09_2166	2009
XIV_b	XIV.2	**XIV.2**	XIV.2	1816	HF969161	chicken	Nigeria	NIE10_024	2011
XIV_b	XIV.2	**XIV.2**	XIV.2	1817	HF969162	chicken	Nigeria	NIE10_032	2011
XIV_b	XIV.2	**XIV.2**	XIV.2	1818	HF969163	chicken	Nigeria	NIE10_034	2011
XIV_b	XIV.2	**XIV.2**	XIV.2	1819	HF969164	chicken	Nigeria	NIE10_041	2011
XIV_b	XIV.2	**XIV.2**	XIV.2	1820	HF969165	chicken	Nigeria	NIE10_043	2011
XIV_b	XIV.2	**XIV.2**	XIV.2	1821	HF969166	chicken	Nigeria	NIE10_076	2011
XIV_b	XIV.2	**XIV.2**	XIV.2	1822	HF969169	chicken	Nigeria	NIE10_150	2011
XIV_b	XIV.2	**XIV.2**	XIV.2	1823	HF969170	chicken	Nigeria	NIE10_160	2011
XIV_b	XIV.2	**XIV.2**	XIV.2	1824	HF969172	chicken	Nigeria	NIE10_258	2011
XIV_b	XIV.2	**XIV.2**	XIV.2	1825	HF969173	chicken	Nigeria	NIE10_302	2011
XIV_b	XIV.2	**XIV.2**	XIV.2	1826	HF969177	chicken	Nigeria	NIE10_409	2011
XIV_b	XIV.2	**XIV.2**	XIV.2	1827	HF969178	chicken	Nigeria	NIE08_2270	2009
XIV_b	XIV.2	**XIV.2**	XIV.2	1828	HF969187	chicken	Nigeria	NIE08_0453	2008
XIV_b	XIV.2	**XIV.2**	XIV.2	1829	HF969190	chicken	Nigeria	NIE08_2032	2009
XIV_b	XIV.2	**XIV.2**	XIV.2	1830	HF969198	chicken	Nigeria	NIE08_2279	2009
XIV_b	XIV.2	**XIV.2**	XIV.2	1831	HF969202	chicken	Nigeria	NIE09_2013	2009
XIV_b	XIV.2	**XIV.2**	XIV.2	1832	HF969203	turkey	Nigeria	NIE09_2021	2009
XIV_b	XIV.2	**XIV.2**	XIV.2	1833	HF969210	chicken	Nigeria	NIE10_139	2011
XIV_b	XIV.2	**XIV.2**	XIV.2	1834	HF969211	chicken	Nigeria	NIE10_263	2011
XIV_b	XIV.2	**XIV.2**	XIV.2	1835	HF969212	chicken	Nigeria	NIE10_318	2011
XIV_b	XIV.2	**XIV.2**	XIV.2	1836	HF969213	chicken	Nigeria	NIE10_325	2011
XIV_b	XIV.2	**XIV.2**	XIV.2	1837	HF969214	chicken	Nigeria	NIE10_333	2011
XIV_b	XIV.2	**XIV.2**	XIV.2	1838	JQ039390	chicken	Nigeria	VRD07_233	2007
XIV_b	XIV.2	**XIV.2**	XIV.2	1839	JX546245	chicken	Benin	463MT	2009

XIV_b	XIV.2	**XIV.2**	XIV.2	1840	KC568206	guinea_fowl	Nigeria	NG_706_JG_KZ_14T	2009
XIV_b	XIV.2	**XIV.2**	XIV.2	1841	KT948996	duck	Nigeria	NG_695_KG_LOM_11_16	2009
XIV_b	XIV.2	**XIV.2**	XIV.2	1842	KY171989	chicken	Nigeria	VRD10_143_N68_913	2010
XIV_b	XIV.2	**XIV.2**	XIV.2	1843	KY171990	chicken	Nigeria	KD_TW_03T_N45_720	2009
XIV_b	XIV.2	**XIV.2**	XIV.2	1844	KY171993	chicken	Nigeria	VRD09_031_N23_715	2009
XIV_b	XIV.2	**XIV.2**	XIV.2	1845	KY171994	chicken	Nigeria	VRD09_001_N19_714	2009
XIV_b	XIV.2	**XIV.2**	XIV.2	2362	MH996919	Turkey	Nigeria	JG_DT_30_31T_N18	2009
XIV_b	XIV.2	**XIV.2**	XIV.2	2365	MH996922	pigeon	Nigeria	KT_MSH_15C_N2	2009
XIV_b	XIV.2	**XIV.2**	XIV.2	2366	MH996923	Wild_bird_eagle	Nigeria	VRD09_546_N4	2009
XIV_b	XIV.2	**XIV.2**	XIV.2	2370	MH996927	chicken	Nigeria	VRD09_340_N50	2009
XIV_b	XIV.2	**XIV.2**	XIV.2	2373	MH996930	chicken	Nigeria	JN_469_N44	2009
XIV_b	XIV.2	**XIV.2**	XIV.2	2381	MH996938	domestic_duck	Nigeria	JG_SH_47C_N15	2009
XIV_b	XIV.2	**XIV.2**	XIV.2	2384	MH996941	chicken	Nigeria	NS_KF_6_09C_N46	2009
XIV_b	XIV.2	**XIV.2**	XIV.2	2385	MH996942	domestic_duck	Nigeria	GM_GMM_17_18T_N14	2009
XIV_b	XIV.2	**XIV.2**	XIV.2	2386	MH996943	pigeon	Nigeria	YB_GSH1_4_6T_N3	2009
XIV_b	XIV.2	**XIV.2**	XIV.2	2388	MH996945	chicken	Nigeria	VRD09_25_N21	2009
XIV_b	XIV.2	**XIV.2**	XIV.2	2389	MH996946	chicken	Nigeria	KT_JBY_09T_N40	2009
XIV_b	XIV.2	**XIV.2**	XIV.2	2397	MH996954	chicken	Nigeria	Dundubus_VRD255_62	2010
XIV_b	XIV.2	**XIV.2**	XIV.2	2398	MH996955	chicken	Nigeria	VRD158A_70	2013
XIV_b	XIV.2	**XIV.2**	XIV.2	2399	MH996956	chicken	Nigeria	Kaduna_VRD401_17	2006
XIV_b	XIV.2	**XIV.2**	XIV.2	2400	MH996957	chicken	Nigeria	Kurmi_VRD033_23	2007
XIV_b	XIV.2	**XIV.2**	XIV.2	2401	MH996958	chicken	Nigeria	Kwara_VRD415_57	2009
XIV_b	XIV.2	**XIV.2**	XIV.2	2402	MH996959	chicken	Nigeria	Vom_VRD216_73	2013
XIV_b	XIV.2	**XIV.2**	XIV.2	2403	MH996960	chicken	Nigeria	Katsina_KT_CH_C18_22_74	2008
XIV_b	XIV.2	**XIV.2**	XIV.2	2423	MH996980	chicken	Nigeria	Vwang_VRD97_67	2013
XIV_b	XIV.2	**XIV.2**	XIV.2	2424	MH996981	chicken	Nigeria	VRD798_XIV_15_798	2015
XIV_b	XIV.2	**XIV.2**	XIV.2	2429	MH996986	chicken	Nigeria	VRD599_59	2009
XIV_b	XIV.2	**XIV.2**	XIV.2	2430	MH996987	chicken	Nigeria	Kogi_VRD578_60	2009
XIV_b	XIV.2	**XIV.2**	XIV.2	2432	MH996989	chicken	Nigeria	Dutse_JG_DT_C32_36_102	2010
V_a	XIX	**XIX**	XIX	463	FJ705456	cormorant	USA_MN_	MN_92_40140	1992
V_a	XIX	**XIX**	XIX	464	FJ705457	cormorant	USA	CA_97_23071	1997
V_a	XIX	**XIX**	XIX	465	FJ705458	cormorant	USA_CA_	CA_D9704285	1997
V_a	XIX	**XIX**	XIX	466	FJ705459	cormorant	Canada	98CNN3_V1125	1998
V_a	XIX	**XIX**	XIX	467	FJ705460	cormorant	Canada	95DC02150	1995
V_a	XIX	**XIX**	XIX	468	FJ705461	cormorant	Canada	95DC2345	1995
V_a	XIX	**XIX**	XIX	469	FJ705463	cormorant	USA_	NV_19529_04	2005
V_a	XIX	**XIX**	XIX	470	GU332655	cormorant	USA_	496	2008
V_a	XIX	**XIX**	XIX	471	GU332662	cormorant	USA_	503	2008
V_a	XIX	**XIX**	XIX	472	GU332663	cormorant	USA_	506	2008
V_a	XIX	**XIX**	XIX	473	JN255775	cormorant	USA	MA_651	2010
V_a	XIX	**XIX**	XIX	474	JN255777	Great_Cormorant	USA	HN_654	2010

V_a	XIX	**XIX**	XIX	475	JN255778	great_black_backed_gull	USA	655	2010
V_a	XIX	**XIX**	XIX	476	JN255779	herring_gull	USA	656	2010
V_a	XIX	**XIX**	XIX	477	JN255781	cormorant	USA	ME_658_	2010
V_a	XIX	**XIX**	XIX	478	JN255782	cormorant	USA	ME_659	2010
V_a	XIX	**XIX**	XIX	479	JN255784	cormorant	USA	MN_648	2010
V_a	XIX	**XIX**	XIX	480	JN255785	cormorant	USA	HN_652	2010
V_a	XIX	**XIX**	XIX	481	JN872159	cormorant	USA	Wisconsin_451287_11_06_	2006
V_a	XIX	**XIX**	XIX	482	JN872161	cormorant	USA	Wisconsin_498260_2_07_	1997
V_a	XIX	**XIX**	XIX	483	JN941988	cormorant	USA	Florida_66638_	2010
V_a	XIX	**XIX**	XIX	484	JN941991	cormorant	USA	Wisconsin_1718_	2008
V_a	XIX	**XIX**	XIX	485	JN941993	pelican	USA	Wisconsin_2415_02_	2008
V_a	XIX	**XIX**	XIX	486	JN942020	cormorant	USA	Oregon_399448_	2005
V_a	XIX	**XIX**	XIX	487	JN942023	cormorant	USA	Oregon_276236_7_	2003
V_a	XIX	**XIX**	XIX	488	JN942024	cormorant	USA	Wisconsin_272409_	2003
V_a	XIX	**XIX**	XIX	489	JN942026	Cormorant	USA	New_York_268898_7	2003
V_a	XIX	**XIX**	XIX	490	JN942029	cormorant	USA	Wisconsin_248	1998
V_a	XIX	**XIX**	XIX	491	JN942030	cormorant	USA	Wisconsin_28547	1997
V_a	XIX	**XIX**	XIX	492	JN942035	cormorant	USA	North_Dakota_43888_6	1992
V_a	XIX	**XIX**	XIX	493	JN942036	turkey	USA	North_Dakota_43084_25	1992
V_a	XIX	**XIX**	XIX	494	JN942038	cormorant	USA	Michigan_38819_5	1992
V_a	XIX	**XIX**	XIX	495	JN942097	cormorant	USA	Wisconsin_1926_	2008
V_a	XIX	**XIX**	XIX	496	KC130083	cormorant	USA	Delaware_39840_2	2010
V_a	XIX	**XIX**	XIX	497	KC433530	cormorant	USA	Florida_41105	2012
V_a	XIX	**XIX**	XIX	498	KC433532	cormorant	USA	Florida_42884	2012
V_a	XIX	**XIX**	XIX	2318	MH392216	cormorant	USA_MN_	92_40140_250	1992
XVI	XVI	**XVI**	XVI	1854	JX119193	chicken	Dominican_Republic	499_31	2008
XVI	XVI	**XVI**	XVI	1855	JX186997	chicken	Dominican_Republic	867	2008
XVI	XVI	**XVI**	XVI	1856	JX915242	chicken	Dominican_Republic	28138_4	1986
XVI	XVI	**XVI**	XVI	1857	JX915243	chicken	Mexico	Queretaro_452_1947	1947
XVII	XVII	**XVII**	XVII	1860	HF969125	chicken	CAR	CAF09_014	2008
XVII	XVII	**XVII**	XVII	1861	HF969180	_	Cameroon	CAE08_318	2009
XVII	XVII	**XVII**	XVII	1862	HF969182	chicken	CAR	CAF09_016	2009
XVII	XVII	**XVII**	XVII	1865	KU058680	chicken	Nigeria	Kudu_113_N56_	1992
XVII_a	XVII	**XVII**	XVII	1869	FJ772449	_	Nigeria	913_33	2006
XVII_a	XVII	**XVII**	XVII	1870	FJ772458	chicken	Burkina_Faso	2415_361	2008
XVII_a	XVII	**XVII**	XVII	1871	FJ772463	chicken	Burkina_Faso	2415_580	2008
XVII_a	XVII	**XVII**	XVII	1872	FJ772469	chicken	Niger	2602_348	2008
XVII_a	XVII	**XVII**	XVII	1873	FJ772472	chicken	Niger	2602_468	2008
XVII_a	XVII	**XVII**	XVII	1874	FJ772475	chicken	Nigeri	2602_605	2008
XVII_a	XVII	**XVII**	XVII	1875	FJ772478	chicken	Cameroon	3490_149	2008
XVII_a	XVII	**XVII**	XVII	1876	FJ772481	chicken	Niger	2602_625	2008

XVII_a	XVII	**XVII**	XVII	1877	FJ772484	chicken	Cameroon	_3490_147	2008
XVII_a	XVII	**XVII**	XVII	1878	FJ772486	_	Nigeria	3724_6	2008
XVII_a	XVII	**XVII**	XVII	1879	HF969129	chicken	Nigeria	NIE08_1363	2008
XVII_a	XVII	**XVII**	XVII	1880	HF969130	guinea_fowl	Nigeria	NIE08_2004	2009
XVII_a	XVII	**XVII**	XVII	1881	HF969132	chicken	Nigeria	NIE08_2149	2009
XVII_a	XVII	**XVII**	XVII	1882	HF969134	chicken	Nigeria	NIE08_2168	2009
XVII_a	XVII	**XVII**	XVII	1883	HF969135	chicken	Nigeria	NIE08_2187	2009
XVII_a	XVII	**XVII**	XVII	1884	HF969138	chicken	Nigeria	NIE08_2224	2009
XVII_a	XVII	**XVII**	XVII	1885	HF969140	chicken	Nigeria	NIE08_2340	2009
XVII_a	XVII	**XVII**	XVII	1886	HF969148	chicken	Nigeria	NIE09_2034	2009
XVII_a	XVII	**XVII**	XVII	1887	HF969152	chicken	Nigeria	NIE09_2072	2009
XVII_a	XVII	**XVII**	XVII	1888	HF969168	chicken	Nigeria	NIE10_124	2011
XVII_a	XVII	**XVII**	XVII	1889	HF969174	chicken	Nigeria	NIE10_304	2011
XVII_a	XVII	**XVII**	XVII	1890	HF969175	chicken	Nigeria	NIE10_306	2011
XVII_a	XVII	**XVII**	XVII	1891	HF969176	chicken	Nigeria	NIE10_310	2011
XVII_a	XVII	**XVII**	XVII	1892	HF969184	chicken	Ivory_Coast	CIV08_103	2007
XVII_a	XVII	**XVII**	XVII	1893	HF969185	chicken	Ivory_Coast	CIV08_104	2007
XVII_a	XVII	**XVII**	XVII	1894	HF969188	chicken	Nigeria	NIE08_1365	2008
XVII_a	XVII	**XVII**	XVII	1895	HF969191	chicken	Nigeria	NIE08_2042	2009
XVII_a	XVII	**XVII**	XVII	1896	HF969192	chicken	Nigeria	NIE08_2119	2009
XVII_a	XVII	**XVII**	XVII	1897	HF969195	chicken	Nigeria	NIE08_2247	2009
XVII_a	XVII	**XVII**	XVII	1898	HF969197	chicken	Nigeria	NIE08_2267	2009
XVII_a	XVII	**XVII**	XVII	1899	HF969199	chicken	Nigeria	NIE08_2349	2009
XVII_a	XVII	**XVII**	XVII	1900	HF969204	chicken	Nigeria	NIE09_2028	2009
XVII_a	XVII	**XVII**	XVII	1901	HF969207	_	Nigeria	NIE09_2167	2009
XVII_a	XVII	**XVII**	XVII	1902	HF969209	chicken	Nigeria	NIE10_123	2011
XVII_a	XVII	**XVII**	XVII	1903	HF969215	chicken	Nigeria	NIE10_335	2011
XVII_a	XVII	**XVII**	XVII	1904	JF966385	chicken	Mali	ML007	2008
XVII_a	XVII	**XVII**	XVII	1905	JQ039393	chicken	Nigeria	VRD07_141	2007
XVII_a	XVII	**XVII**	XVII	1906	JQ039394	chicken	Nigeria	VRD07_410	2007
XVII_a	XVII	**XVII**	XVII	1909	JX546247	chicken	Benin	488MT	2009
XVII_a	XVII	**XVII**	XVII	1910	KC568204	pigeon	Nigeria	ZM_KN_PG01_N1_688	2009
XVII_a	XVII	**XVII**	XVII	1911	KC568208	chicken	Nigeria	NG_710_GM_PLBM_10_12T	2009
XVII_a	XVII	**XVII**	XVII	1912	KY171991	quail	Nigeria	VRD17_04_N2_861	2004
XVII_a	XVII	**XVII**	XVII	1913	KY171992	chicken	Nigeria	JN_469_N44_892	2009
XVII_a	XVII	**XVII**	XVII	1914	KY171995	chicken	Nigeria	VRD124_06_N11_867	2006
XVII_a	XVII	**XVII**	XVII	1915	KY292310	quail	Nigeria	VRD08_385_N23	2009
XVII_b	XVII	**XVII**	XVII	1935	FJ772446	_	Nigeria	913_1	2006
XVII_b	XVII	**XVII**	XVII	1936	HF969128	_	Nigeria	NIE07_216	2007
XVII_b	XVII	**XVII**	XVII	1937	HF969171	chicken	Nigeria	NIE10_182	2011
XVII_b	XVII	**XVII**	XVII	1938	HF969194	chicken	Nigeria	NIE08_2199	2009

XVII_b	XVII	**XVII**	XVII	1939	HF969196	chicken	Nigeria	NIE08_2261	2009
XVII_b	XVII	**XVII**	XVII	1940	KF442614	chicken	Nigeria	228_7	2006
XVII_a	XVII	**XVII**	XVII	2151	MH092810	duck	Kanam	Nigeria_KN_399_N26	2009
XVII_a	XVII	**XVII**	XVII	2361	MH996918	domestic_duck	Nigeria	BA_BAU_R_07T_N17	2009
XVII_a	XVII	**XVII**	XVII	2368	MH996925	chicken	Nigeria	BA_TFB_14C_N38	2009
XVII_a	XVII	**XVII**	XVII	2369	MH996926	chicken	Nigeria	BO_MMC_AGN_6_07T_N42	2009
XVII_a	XVII	**XVII**	XVII	2374	MH996931	Guinea_fowl	Nigeria	KT_MA_5_6C_N7	2009
XVII_a	XVII	**XVII**	XVII	2375	MH996932	Vulture	Nigeria	PL038_N47	2002_2003
XVII_a	XVII	**XVII**	XVII	2376	MH996933	chicken	Nigeria	LTS_11T_N38	2009
XVII_a	XVII	**XVII**	XVII	2378	MH996935	duck	Nigeria	YB_GSHI_07T_N5	2009
XVII_a	XVII	**XVII**	XVII	2382	MH996939	domestic_duck	Nigeria	JG_SH_47C_N15	2009
XVII_a	XVII	**XVII**	XVII	2404	MH996961	chicken	Nigeria	Jos_VRD234_7	2002
XVII_a	XVII	**XVII**	XVII	2406	MH996963	chicken	Nigeria	VRD21_63	2011
XVII_a	XVII	**XVII**	XVII	2407	MH996964	chicken	Nigeria	VRD64_66	2012
XVII_a	XVII	**XVII**	XVII	2408	MH996965	duck	Nigeria	Keffi_NS_KR_DK56_57_93	2010
XVII_a	XVII	**XVII**	XVII	2409	MH996966	chicken	Nigeria	Azare_BA_AZR_C6_7_96	2010
XVII_a	XVII	**XVII**	XVII	2412	MH996969	chicken	Nigeria	Bauchi_VRD154_14	2005
XVII_a	XVII	**XVII**	XVII	2413	MH996970	chicken	Nigeria	Zaria_VRD646_19	2006
XVII_a	XVII	**XVII**	XVII	2414	MH996971	chicken	Nigeria	T_Wada_VRD647_20	2006
XVII_a	XVII	**XVII**	XVII	2416	MH996973	chicken	Nigeria	Katsina_VRD221_40	2007
XVII_a	XVII	**XVII**	XVII	2417	MH996974	chicken	Nigeria	Fune_VRD284_44	2007
XVII_a	XVII	**XVII**	XVII	2418	MH996975	chicken	Nigeria	P_Harcourt_VRD289_46	2007
XVII_a	XVII	**XVII**	XVII	2419	MH996976	chicken	Nigeria	Gombe_VRD316_56	2008
XVII_a	XVII	**XVII**	XVII	2420	MH996977	chicken	Nigeria	Jigawa_JG_BR_T15_101	2010
XVII_a	XVII	**XVII**	XVII	2421	MH996978	chicken	Nigeria	Jos_VRD152_9	2003
XVII_a	XVII	**XVII**	XVII	2425	MH996982	chicken	Nigeria	Owerri_west_VRD798_XVII_15_798	2015
XVII_a	XVII	**XVII**	XVII	2426	MH996983	chicken	Nigeria	Nassarawa_VRD235_15_235	2015
XVII_a	XVII	**XVII**	XVII	2427	MH996984	chicken	Nigeria	Katsina_KT_MG_C2_3_75	2011
XVII_a	XVII	**XVII**	XVII	2428	MH996985	chicken	Nigeria	VRD25_12	2005
XVII_a	XVII	**XVII**	XVII	2431	MH996988	chicken	Nigeria	Keffu_NS_KF_C13_17_90	2010
XVII_a	XVII	**XVII**	XVII	2433	MH996990	chicken	Nigeria	Dutse_JG_DT_C32_36_102	2010
XVII_a	XVII	**XVII**	XVII	2434	MH996991	chicken	Nigeria	Katsina_KT_MDWT3_4_104	2011
XVIII_a	XVIII.1	**XVIII.1**	XVIII.1	1942	FJ772455	_	Mauritania	1532_14	2006
XVIII_a	XVIII.1	**XVIII.1**	XVIII.1	1943	HF969179	chicken	Ivory_Coast	CIV08_026	2007
XVIII_a	XVIII.1	**XVIII.1**	XVIII.1	1944	JF966387	chicken	Mali	ML008	2009
XVIII_a	XVIII.1	**XVIII.1**	XVIII.1	1945	JF966388	chicken	Mali	ML225	2008
XVIII_a	XVIII.1	**XVIII.1**	XVIII.1	1946	JF966389	guinea_fowl	Mali	ML038	2007
XVIII_a	XVIII.1	**XVIII.1**	XVIII.1	1947	JN872157	Green_Wood_Hoope	EH	5801_22	2010
XVIII_a	XVIII.1	**XVIII.1**	XVIII.1	1948	JX518885	chicken	Mali	ML57051T	2010
XVIII_b	XVIII.2	**XVIII.2**	XVIII.2	1950	FJ772466	chicken	IvoryCoast	2601	2008
XVIII_b	XVIII.2	**XVIII.2**	XVIII.2	1951	HF969126	duck	Ivory_Coast	CIV08_062	2006

XVIII_b	XVIII.2	**XVIII.2**	XVIII.2	1952	HF969216	chicken	Nigeria	NIE11_1286	2011
XVIII_b	XVIII.2	**XVIII.2**	XVIII.2	1953	HF969217	chicken	Nigeria	NIE10_171	2011
XVIII_b	XVIII.2	**XVIII.2**	XVIII.2	1954	HF969218	chicken	Ivory_Coast	CIV08_042	2007
XVIII_b	XVIII.2	**XVIII.2**	XVIII.2	1955	HG326600	village_weaver	Ivory_Coast	CIV08_032	2006
XVIII_b	XVIII.2	**XVIII.2**	XVIII.2	1956	JN942101	finch	EH	FinEastern_Hemisphere_1409_12	2008
XVIII_b	XVIII.2	**XVIII.2**	XVIII.2	1957	JX518886	chicken	Mali	ML57072T	2010
XVIII_b	XVIII.2	**XVIII.2**	XVIII.2	1958	JX546248	chicken	Togo	AKO18	2009
XVIII_b	XVIII.2	**XVIII.2**	XVIII.2	2329	MH392227	chicken	Nigeria	OOT_4_1_N69_914	2009
VI_c	XX	**XX**	XX	669	AB465606	chicken	Japan	Ibaraki	1985
VI_c	XX	**XX**	XX	670	AB853928	chicken	Japan	Ibaraki_SM87	1987
VI_c	XX	**XX**	XX	671	AF458015	chicken	China	ZhJ_3	1997
VI_c	XX	**XX**	XX	672	AF458016	chicken	China	ZhJ_2	1986
VI_c	XX	**XX**	XX	673	AF458017	chicken	China	Sh_2	1998
VI_c	XX	**XX**	XX	674	AF458018	chicken	China	Sh_1	1997
VI_c	XX	**XX**	XX	675	EF589137	pigeon	China	GZ_Guizhou	_
VI_c	XX	**XX**	XX	676	HQ839733	chicken	Sweden		1995
VI_c	XX	**XX**	XX	677	KC853020	crested_ibis	China	Shaanxi10	2010
VI_c	XX	**XX**	XX	678	KU373026	ostrich	China	SX_O1	2006
VI_c	XX	**XX**	XX	679	KY042125	chicken	Bulgaria	DolnoLinevo	1992
VI_c	XX	**XX**	XX	683	AF458020	chicken	China	ZJ_1	1991
VI_c	XX	**XX**	XX	684	AF458021	chicken	China	JX_1	1994
VI_c	XX	**XX**	XX	685	GQ507801	chicken	South_Korea	Kr_102	1989
VI_c	XX	**XX**	XX	686	KY042142	quail_	Korea	88_M	1988
VI_c	XX	**XX**	XX	687	KY042143	chicken	Korea	93_58GG	1993
VI_c	XX	**XX**	XX	2004	MF278934.1	pigeon	China	GZ_HD_PN	2011
VI_l	XXI	**XXI**	XXI	865	KC205475	chicken	Ethiopia	ETH10065	2011
VI_l	XXI	**XXI**	XXI	866	KC205476	chicken	Ethiopia	_ETH_10073	2011
VI_l	XXI	**XXI**	XXI	867	KC205477	chicken	Ethiopia	ETH8755	2011
VI_l	XXI	**XXI**	XXI	868	KC205478	chicken	Ethiopia	ETHAN01	2011
VI_l	XXI	**XXI**	XXI	869	KC205479	chicken	Ethiopia	ETHMG1C	2011
VI_l	XXI	**XXI**	XXI	870	KJ958913	chicken	Ethiopia	13VIR3936_1	2012
VI_l	XXI	**XXI**	XXI	871	KJ958914	chicken	Ethiopia	13VIR3936_27	2012
VI_g	XXI.1.1	**XXI.1.1**	XXI.1.1	728	JF824013	pigeon	Russia	Kemerovo_0267	2009
VI_g	XXI.1.1	**XXI.1.1**	XXI.1.1	729	JF824032	pigeon	Russia	Vladimir_687	2005
VI_g	XXI.1.1	**XXI.1.1**	XXI.1.1	731	KJ914671	pigeon	Ukraine	Dnipropetrovsk_1_18_11	2011
VI_g	XXI.1.1	**XXI.1.1**	XXI.1.1	732	KJ914672	pigeon	Ukraine	Ukromne_3_26_11	2011
VI_g	XXI.1.1	**XXI.1.1**	XXI.1.1	733	KT962979	pigeon	Russia	Altai_777	2010
VI_g	XXI.1.1	**XXI.1.1**	XXI.1.1	734	KT965727	pigeon	Kazakhstan	EKO_15	2014
VI_g	XXI.1.1	**XXI.1.1**	XXI.1.1	735	KT965728	pigeon	Kazakhstan	Zhambyl_32	2014
VI_g	XXI.1.1	**XXI.1.1**	XXI.1.1	736	KX352835	mallard	Russia	Amur_264	2009
VI_g	XXI.1.1	**XXI.1.1**	XXI.1.1	737	KY042127	pigeon	Ukraine	Kharkiv_23_01_967	2013

VI_g	XXI.1.1	**XXI.1.1**	XXI.1.1	738	KY042128	pigeon	Ukraine	Doneck_3	2007
VI_g	XXI.1.1	**XXI.1.1**	XXI.1.1	739	KY042129	pigeon	Egypt	11_CL_G1_	2015
VI_g	XXI.1.1	**XXI.1.1**	XXI.1.1	740	KY042130	pigeon	Egypt	44_CL_G24_	2015
VI_g	XXI.1.1	**XXI.1.1**	XXI.1.1	741	KY042131	pigeon	Egypt	56_CL_G25_	2015
VI_g	XXI.1.1	**XXI.1.1**	XXI.1.1	742	KY042132	pigeon	Egypt	73_OP_G29_	2015
VI_g	XXI.1.1	**XXI.1.1**	XXI.1.1	743	KY042134	pigeon	Egypt	84_OP_G31_	2015
VI_g	XXI.1.1	**XXI.1.1**	XXI.1.1	744	KY042136	pigeon	Pakistan	Lahore_125	2015
VI_g	XXI.1.1	**XXI.1.1**	XXI.1.1	745	KY042137	pigeon	Pakistan	Jhang_115	2015
VI_g	XXI.1.1	**XXI.1.1**	XXI.1.1	746	KY042138	pigeon	Pakistan	Lahore_126	2015
VI_g	XXI.1.1	**XXI.1.1**	XXI.1.1	747	KY042139	pigeon	Pakistan	Lahore_146	2016
VI_g	XXI.1.1	**XXI.1.1**	XXI.1.1	748	JQ039385	dove	Nigeria	dove_VRD07_163	2007
VI_g	XXI.1.1	**XXI.1.1**	XXI.1.1	2349	MH717070.1	pigeon	Pakistan	AJK_AW_p54	2018
VI_g	XXI.1.1	**XXI.1.1**	XXI.1.1	2396	MH996953	pigeon	Nigeria	Kazaure_VRD231_42	2007
VI_g	XXI.1.1	**XXI.1.1**	XXI.1.1	2437	MK005973	pigeon	Egypt	Souqal_Cairo_39_L_G23_1105	2015
VI_m	XXI.1.2	**XXI.1.2**	XXI.1.2	873	KU862297	pigeon	Pakistan	Lahore_AW_1	2014
VI_m	XXI.1.2	**XXI.1.2**	XXI.1.2	874	KU862298	pigeon	Pakistan	Lahore_AW_2	2015
VI_m	XXI.1.2	**XXI.1.2**	XXI.1.2	875	KU885949	pigeon	Pakistan	MZS_UVAS	2014
VI_m	XXI.1.2	**XXI.1.2**	XXI.1.2	876	KX236100	pigeon	Pakistan	21A	2015
VI_m	XXI.1.2	**XXI.1.2**	XXI.1.2	877	KX236101	pigeon	Pakistan	25A	2015
VI_m	XXI.1.2	**XXI.1.2**	XXI.1.2	878	KY042135	pigeon	Pakistan	22A	2015
VI_m	XXI.1.2	**XXI.1.2**	XXI.1.2	879	KY042140	pigeon	Pakistan	Jallo_Lahore_221A	2016
VI_m	XXI.1.2	**XXI.1.2**	XXI.1.2	880	KY042141	pigeon	Pakistan	Jallo_Lahore_221B	2016
VI_m	XXI.1.2	**XXI.1.2**	XXI.1.2	2350	MH717071.1	pigeon	Pakistan	AJK_AW_p51	2018
VI_m	XXI.1.2	**XXI.1.2**	XXI.1.2	2351	MH717072.1	pigeon	Pakistan	AJK_AW_p52	2018
VI_m	XXI.1.2	**XXI.1.2**	XXI.1.2	2352	MH717073.1	pigeon	Pakistan	AJK_AW_p53	2018
VI_i	XXI.2	**XXI.2**	XXI.2	762	HG424625	pigeon	Nigeria	NIE13_005	2013
VI_i	XXI.2	**XXI.2**	XXI.2	763	JN638234	dove	Italy	11RS98_102VIR	2011
VI_i	XXI.2	**XXI.2**	XXI.2	764	JN638235	dove	Italy	11RS100_104VIR	2011
VI_i	XXI.2	**XXI.2**	XXI.2	765	JN638236	dove	Italy	10RS6171_7154VIR	2010
VI_i	XXI.2	**XXI.2**	XXI.2	766	KU377533	Turtle_Dove	Italy	10VIR7155	2010
VI_i	XXI.2	**XXI.2**	XXI.2	767	KU377535	Turtle_Dove	Italy	12VIR1876_1	2012
VI_i	XXI.2	**XXI.2**	XXI.2	768	KU377536	Turtle_Dove	Italy	12VIR604	2012
VI_i	XXI.2	**XXI.2**	XXI.2	2104	MG456676.1	collared_dove	Iran		2014
VI_i	XXI.2	**XXI.2**	XXI.2	2148	MH044693.1	pigeon	Iran	Konarak_Barin	2017
VI_i	XXI.2	**XXI.2**	XXI.2	2286	MH377298.1	eurasian_collared_dove	Israel	PHL264746	2010
