# Supplementary material for: Updated unified phylogenetic classification system and revised nomenclature for Newcastle disease virus
Source: Infect Genet Evol. 2019 Oct;74:103917. doi: 10.1016/j.meegid.2019.103917 (PMC6876278; doi:10.1016/j.meegid.2019.103917)
Supplement: Supplemental Table S3 — Complete fusion gene “pilot” dataset of class II NDV used in this study. The dataset contains 125 sequences and was used to build the tree depicted in Fig. 2. [file mmc3.pdf]

Supplemental Table S3. Complete fusion gene “pilot” dataset of class II NDV used in this study. The dataset contains 125 sequences and was used to build the trees depicted in Figure 2.

Genotypes based on the new classification and nomenclature system	Based on Diel et al. 2012	Acc. Number	Host	Country	Isolate	Year
I.1.1	I-a	AY935490	chicken	Australia	2_1334	2002
I.1.1	I-a	AY935495	chicken	Australia	99_868_hi	1999
I.1.1	I-a	M24693	chicken	Australia	Queensland	1966
I.1.2.1	I-c	EF564816	redknot	USA	NJ_A_101_1383	2001
I.1.2.1	I-c	GQ918280	black_headed_gull	Sweden		1994
I.1.2.1	I-c	KX352834	gull	Russia	Tyva_14	2014
I.1.2.2	I-d	AB465607	chicken	Japan	Ishi	1962
I.1.2.2	I-d	KC503476	northern_pintail	USA	AK_44500_136	2009
I.1.2.2	I-d	KC503479	redpoll	Russia	Nikita_530_FFNK2	2008
I.2	I-b	AY965079	duck	Russia	FarEast_2713	2001
I.2	I-b	HG326605	spur_winged_goose	Nigeria	NIE08_121	2008
I.2	I-b	KC503453	American_green_winged_teal	USA	AK_44493_716	2009
II	II	AF077761	chicken	USA	Lasota	1946
II	II	GU978777	chicken	USA	TX_GB	1948
II	II	JN872151	chicken	USA	Hitchner_B1	1947
III	III	EF201805	avian		Mukteswar	1940
III	III	GU182327	chicken	Pakistan	SPVC_Karachi_1	1974
III	III	MH996904	pigeon	Bulgaria	Novo_Selo_1161	1995
IV	IV	AY741404	Fowl	UK	Herts	1933
IV	IV	MH996900	pullet	Bulgaria	Plovdiv_1153	1959
V.1	V-b	JN872189	parrot	USA	Coast_8278	1982
V.1	V-b	JN872194	chicken	Honduras	498109_15	2007
V.1	V-b	JN942027	fighting_cock	Nicaragua	95066_9	2001
V.2	V-c	EU518682	Dove	Mexico	Distrito_Federal_462	2004
V.2	V-c	EU518684	chicken	Mexico	Estado_de_Mexico_466	2006
V.2	V-c	JQ697744	chicken	Mexico	NC04_635	2010
VI.2.1.1.1	VI-a	JX901367	pigeon	USA	PA_810	2008
VI.2.1.1.1	VI-a	JX901351	pigeon	USA	NJ_721	2007

VI.2.1.1.1	VI-n	MG018211	ECDO	USA	TX_1185_kidney_26981_3_A	2015
VI.2.1.1.2.1	VI-j	JX094510	pigeon	China	sms12	2012
VI.2.1.1.2.1	VI-j	JX901110	pigeon	Belgium	248_	1998
VI.2.1.1.2.1	VI-j	JX486553	pigeon	China	LHLJ_110813	2011
VI.2.1.1.2.2	VI-k	KT163262	pigeon	China	SH_167	2013
VI.2.1.1.2.2	VI-k	JX901124	pigeon	Belgium	11_09620	2011
VI.2.1.1.2.2	VI-k	MG840654.1	pigeon	China	Ningxia_2068	2016
VI.1	VI-b	AF109885	domestic_fowl	Great_Britain	GB1168	1984
VI.1	VI-b	FJ410145	pigeon	USA	NY	1984
VI.1	VI-b	FJ865434	pigeon	China	S_1	2002
VI.2.2.2	VI-e	FJ480825	pigeon	China	PG_JS_1	2005
VI.2.2.2	VI-e	JX244794	pigeon	China	100	2008
VI.2.2.2	VI-e	KJ607163	pigeon	China	LJS_1	2004
VI.2.2.1	VI-f	JN872180	waterfowl	USA	TX_209682	2002
VI.2.2.1	VI-f	JN872182	pigeon	USA	12339	1998
VI.2.2.1	VI-f	JX901312	pigeon	USA	101	2001
VI.2.1.2	VI-h	HG326604	pigeon	Nigeria	NIE09_1898	2009
VI.2.1.2	VI-h	JX518532	laughing_dove	Kenya	B2_Isiolo	2012
VI.2.1.2	VI-h	HG424627	pigeon	Nigeria	NIE13_92	2013
VII.1.1	VII-b	EF589133	pheasant	China	98_Guizhou	1998
VII.1.1	VII-d	EF579733	chicken	China	Shandong_Pyan	2004
VII.1.1	VII-e	AB853927	chicken	Japan	Ibaraki_SG106	1999
VII.1.1	VII-j	KC542905	chicken	China	Liaoning_1_2009	2009
VII.1.1	VII-l	KX268351	chicken	Iran	Behshahr	2015
VII.1.2	VII-f	AY028995	fowl	China	A7	1996
VII.1.2	VII-f	GQ338309	pigeon	China	18	2003
VII.1.2	VII-f	DQ227246	goose	China	Jiangsu_JS02	1999
VII.2	VII-h	MF622047	chicken	South_Africa	RBWW_3	2013
VII.2	VII-i	KU862293	Parakeet	Pakistan	Karachi_AW_1	2014
VII.2	VII-i	HQ697254	chicken	Indonesia	Banjarmasin_10	2010
VII.2	VII-k	KY747479	chicken	Namibia	5620	2016
VII.2	VII-a	JN986837	chicken	Netherlands	152608_ancestral	1993
VIII	VIII	AY734534	chicken	Argentina	Trenque_Lauquen	1970
VIII	VIII	FJ751918	chicken	China	QH1	1979

VIII	VIII	JX012096		Malaysia	AF2240	1960
IX	IX	AF458009	chicken	China	FJ_1	1985
IX	IX	FJ436303	chicken	China	ZJ_1	1986
IX	IX	FJ436302	chicken	China	F48E8	1948
X	X-a	FJ705468	mottled_duck	USA	TX_130	2011
X	X-a	KX857716	Redhead	USA	ndv42_AI09_4117	2009
X	X-b	FJ705466	mallard		99_376	1999
X	X-b	KX857721	Mallard	USA	MN_AI10_3434	2010
XI	XI	HQ266602	chicken	Madagascar	MG_725	2008
XI	XI	JX518882	chicken	Madagascar	MGMNJ	2009
XI	XI	JX518884	chicken	Madagascar	MGS1595T	2011
XII.1	XII-a	KU594615	chicken	Peru	Apurimac_50009	2005
XII.1	XII-a	KU594616	gamecock	Peru	Lurin_40871	2004
XII.1	XII-a	KU594618	chicken	Peru	Arequipa_VFAR_81	2015
XII.2	XII-b	JN627504	goose	China	GD_12	2011
XII.2	XII-b	JN627507	goose	China	GD_1003	2010
XII.2	XII-b	MF278927	goose	China	FS_SS_292	2013
XIII.1.1	XIII-a	JN942034	ostrich	South_Africa	45445_3	1995
XIII.1.1	XIII-a	JN942043	roller	Tanzania	47385_11	2010
XIII.1.1	XIII-a	MF409241	chicken	Zambia	Chiwoko	2015
XIII.2.1	XIII-b	GU182323	chicken	Pakistan	SPVC_Karachi_43	2008
XIII.2.1	XIII-b	GU182331	chicken	Pakistan	SPVC_Karachi_33_	2007
XIII.2.1	XIII-b	KF113338	chicken	Pakistan	University_Diagnostic_Lab_12	2010
XIII.2.2	XIII-b	KM056349	chicken	India	ndv42_gopalpura_4	2013
XIII.2.2	XIII-b	KT734767	chicken	India	Polashbari	2014
XIII.2.2	XIII-b	KX372707	chicken	India	Nagpur_3	2011
XIII.1.2	XIII-a	JQ267579	chicken	Iran	EMM_7	2011
XIII.1.2	XIII-a	JQ267584	chicken	Iran	EMM_2	2008
XIII.1.2	XIII-a	JQ267585	chicken	Iran	EMM_1	2008
XIV.1	XIV-a	HF969205	turkey	Nigeria	NIE09_2071	2009
XIV.1	XIV-a	JN872165	chicken	Niger	VIR_1377_7	2006
XIV.1	XIV-a	JQ039386	chicken	Nigeria	VRD08_36	2008
XIV.2	XIV-b	HF969187	chicken	Nigeria	NIE08_453	2008
XIV.2	XIV-b	HF969210	chicken	Nigeria	NIE10_139	2011

XIV.2	XIV-b	KY171990	chicken	Nigeria	KD_TW_03T_N45_720	2009
XVI	XVI	JX915242	chicken	Dominican_Republic	28138_4	1986
XVI	XVI	JX915243	chicken	Mexico	Queretaro_452_1947	1947
XVI	XVI	JX186997	chicken	Dominican_Republic	867	2008
XVII	XVII-a	HF969176	chicken	Nigeria	NIE10_310	2011
XVII	XVII-a	HF969191	chicken	Nigeria	NIE08_2042	2009
XVII	XVII-b	HF969194	chicken	Nigeria	NIE08_2199	2009
XVIII.1	XVIII-a	FJ772455		Mauritania	1532_14	2006
XVIII.1	XVIII-a	JF966389	guinea_fowl	Mali	ML038	2007
XVIII.1	XVIII-a	JX518885	chicken	Mali	ML57051T	2010
XVIII.2	XVIII-b	HF969218	chicken	Ivory_Coast	CIV08_42	2007
XVIII.2	XVIII-b	HG326600	village_weaver	Ivory_Coast	CIV08_32	2006
XVIII.2	XVIII-b	JX518886	chicken	Mali	ML57072T	2010
XIX	V-a	FJ705456	cormorant	USA	MN_92_40140	1992
XIX	V-a	JN942024	cormorant	USA	WI_272409	2003
XIX	V-a	KC433530	cormorant	USA	FL_41105	2012
XX	VI-c	AB853928	chicken	Japan	Ibaraki_SM87	1987
XX	VI-c	AF458016	chicken	China	ZhJ_2	1986
XX	VI-c	KY042142	quail	Korea	88_M	1988
XXI	VI-l	KC205479	chicken	Ethiopia	ETHMG1C	2011
XXI.2	VI-i	JN638234	dove	Italy	11RS98_102VIR	2011
XXI.2	VI-i	KU377533	Turtle_dove	Italy	10VIR7155	2010
XXI.2	VI-i	KU377535	Turtle_dove	Italy	12VIR1876_1	2012
XXI.1.2	VI-m	KU862298	pigeon	Pakistan	Lahore_AW_2	2015
XXI.1.2	VI-m	KY042135	Pigeon	Pakistan	22A	2015
XXI.1.2	VI-m	KY042141	Pigeon	Pakistan	Jallo_Lahore_221B	2016
XXI.1.1	VI-g	JF824032	pigeon	Russia	Vladimir_687	2005
XXI.1.1	VI-g	KY042136	Pigeon	Pakistan	Lahore_125	2015
XXI.1.1	VI-g	KY042132	Pigeon	Egypt	73_OP_G29	2015
