# Supplementary material for: Updated unified phylogenetic classification system and revised nomenclature for Newcastle disease virus
Source: Infect Genet Evol. 2019 Oct;74:103917. doi: 10.1016/j.meegid.2019.103917 (PMC6876278; doi:10.1016/j.meegid.2019.103917)
Supplement: Supplemental Table S4 — Former sub-genotypes within class I and class II that did not pass the updated classification criteria (either one of 5% nucleotide distance, branch support equal or above 70%, at least 4 independent isolates) and were merged. The sub/genotype designation follows the newly proposed nomenclature and the former names are provided in parenthesis. [file mmc4.pdf]

Supplemental Table S4. Former sub-genotypes within class I and class II that did not pass the updated classification criteria (either one of 5% nucleotide distance, branch support equal or above 70%, at least 4 independent isolates) and were merged. The sub/genotype designation follows the newly proposed nomenclature and the former names are provided in parenthesis.

Distance between groups is below 5%

Class I	
	**1.2 (1c)**
**1.2 (1d)**	3.68

Class II genotype VI	
	**VI.1.2.1.1.1 (VIn)**
**VI.1.2.1.1.1 (VIa)**	3.46

Class II genotype X	
	**X (Xa)**
**X (Xb)**	4.83

Class II genotype XVII	
	**XVII (XVIIb)**
**XVII (XVIIa)**	4.59

Class II genotype VII	
	**VII.1.1 (VIIe)**
**VII.1.1 (b+j+d+ℓ)**	4.21

Class II genotype VII	
	**VII.1.1 (VIId+VIIℓ)**
**VII.1.1 (VIIb+VIIj)**	4.68

Class II genotype VII	
	**VII.1.1 (VIIj)**
**VII.1.1 (VIIb)**	3.31

Lack of branch support or sufficient number of epidemiologically independent isolates

Class II genotype VII	
	**VII.2 (VIIi)**
**VII.2 (VIIh+VIIk)**	9.61

Class II genotype VII	
	**VII.2 (VIIk)**
**VII.2 (VIIh)**	11.10

Class II genotype V	
	**V (Vd)**
**V.1 + V.2 (Vb+Vc)**	9.96

Class II genotype XXI	
	**XXI (VI ℓ)**
**XXI.1+XXI.2 (VIg+m+i)**	13.24
