# Supplementary material for: Updated unified phylogenetic classification system and revised nomenclature for Newcastle disease virus
Source: Infect Genet Evol. 2019 Oct;74:103917. doi: 10.1016/j.meegid.2019.103917 (PMC6876278; doi:10.1016/j.meegid.2019.103917)
Supplement: Supplemental Table S5 — Recommended sequences to be used for rooting purposes when building sub-trees for separate analysis of each genotype within class II. The sequence to use for rooting is marked with an asterisk. These sequences were used to root the trees presented in Supplemental Fig. S7 A–I. [file mmc5.pdf]

Supplemental Table S5. Recommended sequences to be used for rooting purposes when building sub-trees for separate analysis of each genotype within class II. The sequence to use for rooting is marked with an asterisk. These sequences were used to root the trees presented in Supplemental Fig. S7 A-I.

Genotype	Root with (GenBank number)
I, II, VIII, IX, X, XI, XVI	AY741404* and EF201805
III	AY741404* and EU293914*
IV	EF201805* and M24700*
V, VI, VII, XIV, XVII, XVIII, XIX, XX	JX915243* and AF048763
XII	JX915243* and JX393313
XIII	AY741404*, EF201805, JX915243 and AF048763
XXI	JX915243* and Z12111
