# Supplementary material for: Updated unified phylogenetic classification system and revised nomenclature for Newcastle disease virus
Source: Infect Genet Evol. 2019 Oct;74:103917. doi: 10.1016/j.meegid.2019.103917 (PMC6876278; doi:10.1016/j.meegid.2019.103917)

Supplemental Fig. S2. Examples for naming new genotypes when they outgroup or not existing genotypes. The names of genotypes and sub-genotypes used in this figure do not correspond to the names in the phylogenetic trees presented in the current study. The names in this figure were used for illustration and demonstration purposes.

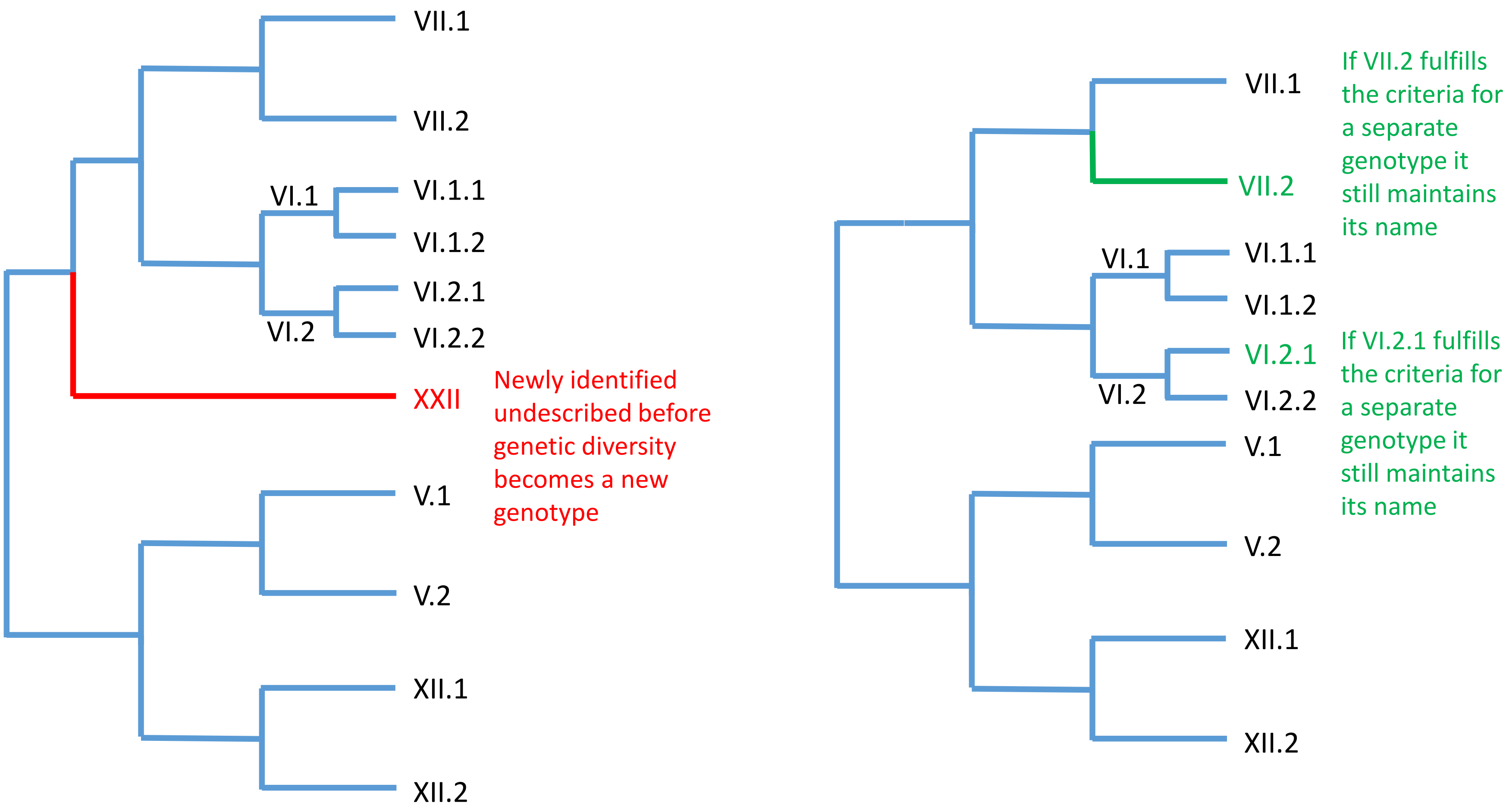

Supplement: Supplemental Fig. S2 — Examples for naming new genotypes when they outgroup or not existing genotypes. The names of genotypes and sub-genotypes used in this figure do not correspond to the names in the phylogenetic trees presented in the current study. The names in this figure were used for illustration and demonstration purposes. [file mmc7.pdf]
