# Supplementary material for: Updated unified phylogenetic classification system and revised nomenclature for Newcastle disease virus
Source: Infect Genet Evol. 2019 Oct;74:103917. doi: 10.1016/j.meegid.2019.103917 (PMC6876278; doi:10.1016/j.meegid.2019.103917)

Supplemental Fig. S5. Full phylogenetic trees based on the complete fusion gene sequences of isolates representing Newcastle disease virus class II (n = 1672). A. Neighbor-joining tree; B. Maximum Likelihood tree; C. Bayesian tree. The Roman numerals presented in the taxa names in the phylogenetic trees represent the respective genotype for each isolate. The new (decimal naming) and the old names (alpha-numerical) are provided for easier comparison. The numbers after the genotype names represent database ID. The names based on the new system differ for some genotypes in the different trees based on differences in topology. The taxa names also include the GenBank identification number, and when available – host name, country of isolation, strain designation, and year of isolation. The trees are drawn to scale, with branch lengths measured in the number of substitutions per site.

Supplemental Fig. S5A
CLASS II
NEIGHBOR-JOINING TREE

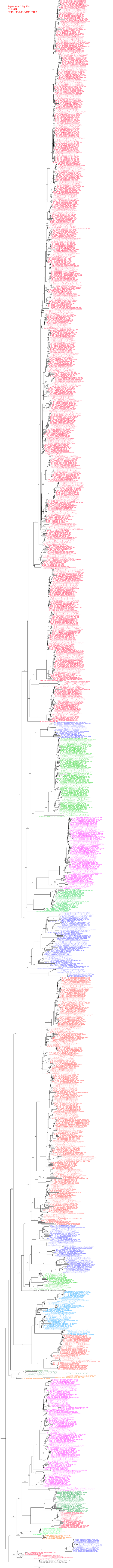

Supplemental Fig. S5B
CLASS II
MAXIMUM-LIKELIHOOD TREE

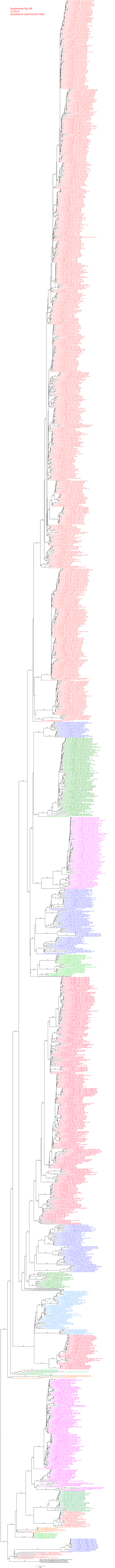

Supplemental Fig. S5C
CLASS II
BAYESIAN TREE

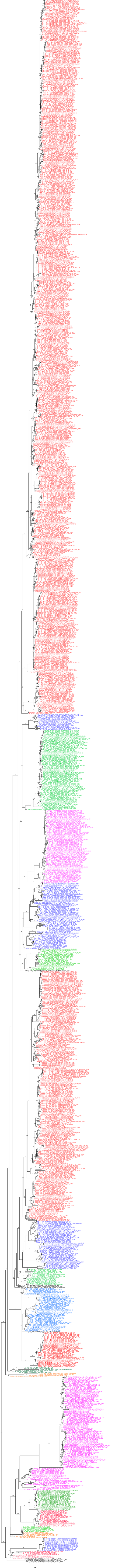

Supplement: Supplemental Fig. S5 — Full phylogenetic trees based on the complete fusion gene sequences of isolates representing Newcastle disease virus class II (n = 1672). A. Neighbor-joining tree; B. Maximum Likelihood tree; C. Bayesian tree. The Roman numerals presented in the taxa names in the phylogenetic trees represent the respective genotype for each isolate. The new (decimal naming) and the old names (alpha-numerical) are provided for easier comparison. The numbers after the genotype names represent database ID. The names based on the new system differ for some genotypes in the different trees based on differences in topology. The taxa names also include the GenBank identification number, and when available – host name, country of isolation, strain designation, and year of isolation. The trees are drawn to scale, with branch lengths measured in the number of substitutions per site. [file mmc10.pdf]
