# Supplementary material for: Updated unified phylogenetic classification system and revised nomenclature for Newcastle disease virus
Source: Infect Genet Evol. 2019 Oct;74:103917. doi: 10.1016/j.meegid.2019.103917 (PMC6876278; doi:10.1016/j.meegid.2019.103917)

Supplemental Fig. S6. Full phylogenetic tree based on the complete genome concatenated coding sequences of isolates representing Newcastle disease virus class II (n = 491). The evolutionary history was inferred by using the Maximum Likelihood method based on the General Time Reversible model with 1000 bootstrap replicates. The tree with the highest log likelihood (-344411.91) is shown. A discrete gamma distribution was used to model evolutionary rate differences among sites and the rate variation model allowed for some sites to be evolutionarily invariable. The Roman numerals presented in the taxa names in the phylogenetic tree represent the respective genotype for each isolate. The new (decimal naming) and the old names (alpha-numerical) are provided for easier comparison. The names of sub/genotypes are based on the proposed system and as identified by the ML complete fusion gene analysis. The taxa names also include the GenBank identification number, host name, country of isolation, strain designation, and year of isolation. The tree is drawn to scale, with branch lengths measured in the number of substitutions per site.

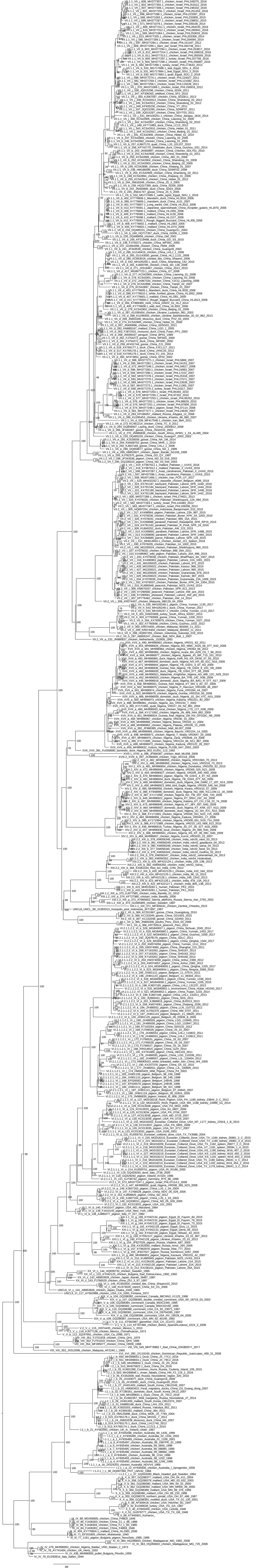

Supplement: Supplemental Fig. S6 — Full phylogenetic tree based on the complete genome concatenated coding sequences of isolates representing Newcastle disease virus class II (n = 491). The evolutionary history was inferred by using the Maximum Likelihood method based on the General Time Reversible model with 1000 bootstrap replicates. The tree with the highest log likelihood (−344,411.91) is shown. A discrete gamma distribution was used to model evolutionary rate differences among sites and the rate variation model allowed for some sites to be evolutionarily invariable. The Roman numerals presented in the taxa names in the phylogenetic tree represent the respective genotype for each isolate. The new (decimal naming) and the old names (alpha-numerical) are provided for easier comparison. The names of sub/genotypes are based on the proposed system and as identified by the ML complete fusion gene analysis. The taxa names also include the GenBank identification number, host name, country of isolation, strain designation, and year of isolation. The tree is drawn to scale, with branch lengths measured in the number of substitutions per site. [file mmc11.pdf]
