# Supplementary material for: Updated unified phylogenetic classification system and revised nomenclature for Newcastle disease virus
Source: Infect Genet Evol. 2019 Oct;74:103917. doi: 10.1016/j.meegid.2019.103917 (PMC6876278; doi:10.1016/j.meegid.2019.103917)
Supplement: Supplemental Fig. S7 — Phylogenetic sub-tree based on the complete fusion gene sequence of isolates representing Newcastle disease virus genotypes. A. Genotype I (n = 122); B. Genotype V (n = 49); C. Genotype VI (n = 267); D. Genotype VII (n = 774); E. XII. Genotype I (n = 25); F. Genotype XIII (n = 74); G. Genotype XIV (n = 79); H. Genotype XVIII (n = 19); I. Genotype XXI (n = 53). The trees were built using all sequences within each NDV genotypes and 2 to 4 additional sequences used for rooting. The sequences recommended for use for rooting are also provided in Supplemental Table S5. The evolutionary history was inferred by using the Maximum Likelihood method based on the General Time Reversible model with 1000 bootstrap replicates. The trees with the highest log likelihood are shown. A discrete gamma distribution was used to model evolutionary rate differences among sites and the rate variation model allowed for some sites to be evolutionarily invariable. The Roman numerals presented in the taxa names in the phylogenetic tree represent the respective genotype for each isolate. The new (decimal naming) and the old names (alpha-numerical) are provided for easier comparison. The taxa names also include the GenBank identification number, and when available - host name, country of isolation, strain designation, and year of isolation. The trees are drawn to scale, with branch lengths measured in the number of substitutions per site. [file mmc12.pdf]

Supplemental Fig. S7. Phylogenetic sub-tree based on the complete fusion gene sequence of isolates representing Newcastle disease virus genotypes. A. Genotype I (n = 122); B. Genotype V (n = 49); C. Genotype VI (n = 267); D. Genotype VII (n = 774); E. XII. Genotype I (n = 25); F. Genotype XIII (n = 74); G. Genotype XIV (n = 79); H. Genotype XVIII (n = 19); I. Genotype XXI (n = 53). The trees were built using all sequences within each NDV genotypes and 2 to 4 additional sequences used for rooting. The sequences recommended for use for rooting are also provided in Supplemental Table S5. The evolutionary history was inferred by using the Maximum Likelihood method based on the General Time Reversible model with 1000 bootstrap replicates. The trees with the highest log likelihood are shown. A discrete gamma distribution was used to model evolutionary rate differences among sites and the rate variation model allowed for some sites to be evolutionarily invariable. The Roman numerals presented in the taxa names in the phylogenetic tree represent the respective genotype for each isolate. The new (decimal naming) and the old names (alpha-numerical) are provided for easier comparison. The taxa names also include the GenBank identification number, and when available - host name, country of isolation, strain designation, and year of isolation. The trees are drawn to scale, with branch lengths measured in the number of substitutions per site.

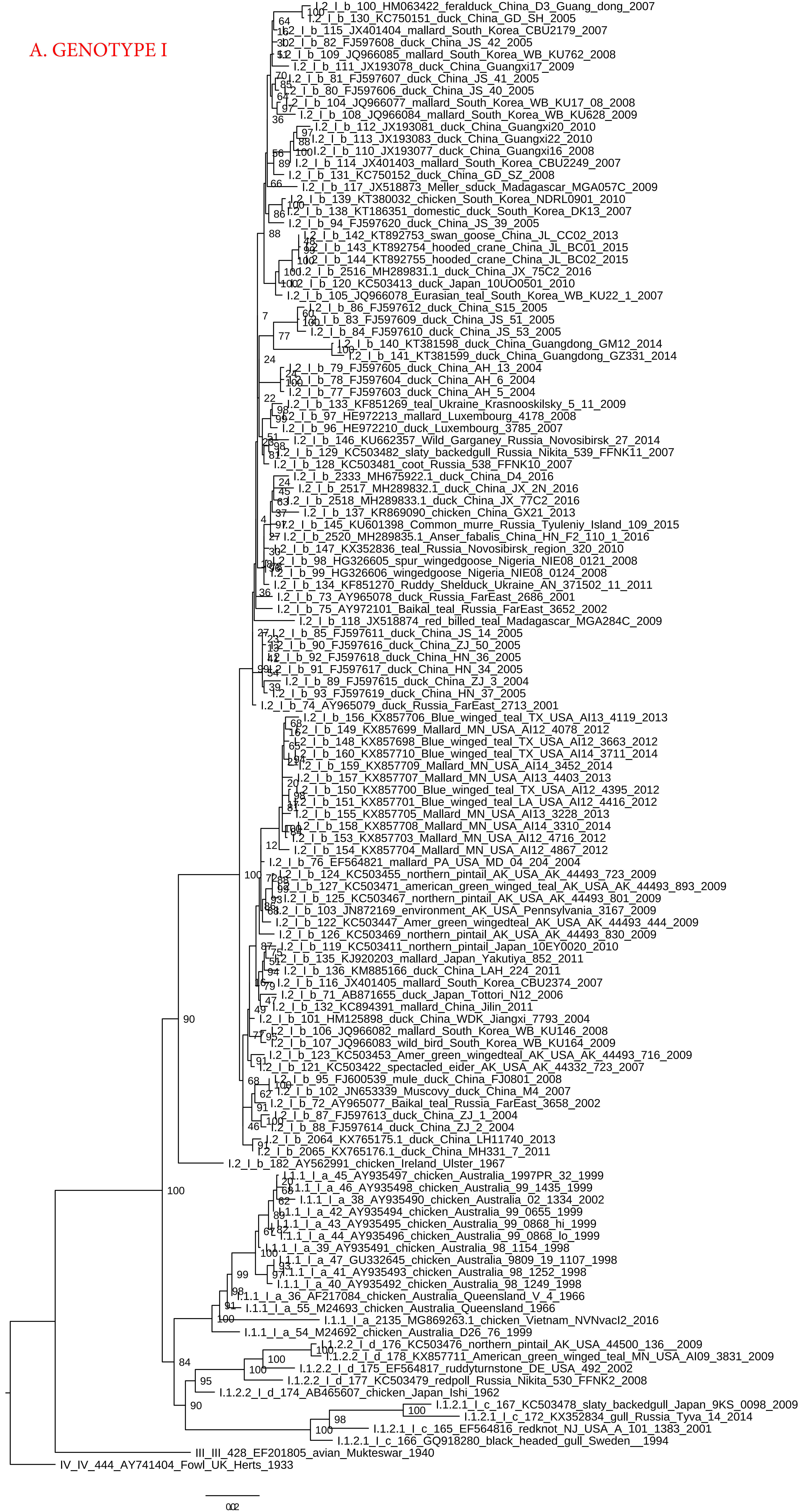
A. GENOTYPE I
I.2_I_b_100_HM063422_feralduck_China_D3_Guang_dong_2007
I.2_I_b_130_KC750151_duck_China_GD_SH_2005
I.2_I_b_115_JX401404_mallard_South_Korea_CBU2179_2007
I.2_I_b_82_FJ597608_duck_China_JS_42_2005
I.2_I_b_109_JQ966085_mallard_South_Korea_WB_KU762_2008
I.2_I_b_111_JX193078_duck_China_Guangxi17_2009
I.2_I_b_81_FJ597607_duck_China_JS_41_2005
I.2_I_b_80_FJ597606_duck_China_JS_40_2005
I.2_I_b_104_JQ966077_mallard_South_Korea_WB_KU17_08_2008
I.2_I_b_108_JQ966084_mallard_South_Korea_WB_KU628_2009
I.2_I_b_112_JX193081_duck_China_Guangxi20_2010
I.2_I_b_113_JX193083_duck_China_Guangxi22_2010
I.2_I_b_110_JX193077_duck_China_Guangxi16_2008
I.2_I_b_114_JX401403_mallard_South_Korea_CBU2249_2007
I.2_I_b_131_KC750152_duck_China_GD_SZ_2008
I.2_I_b_117_JX518873_Meller_sduck_Madagascar_MGA057C_2009
I.2_I_b_139_KT380032_chicken_South_Korea_NDRL0901_2010
I.2_I_b_138_KT186351_domestic_duck_South_Korea_DK13_2007
I.2_I_b_94_FJ597620_duck_China_JS_39_2005
I.2_I_b_142_KT892753_swan_goose_China_JL_CC02_2013
I.2_I_b_143_KT892754_hooded_crane_China_JL_BC01_2015
I.2_I_b_144_KT892755_hooded_crane_China_JL_BC02_2015
I.2_I_b_2516_MH289831.1_duck_China_JX_75C2_2016
I.2_I_b_120_KC503413_duck_Japan_10UO0501_2010
I.2_I_b_105_JQ966078_Eurasian_teal_South_Korea_WB_KU22_1_2007
I.2_I_b_86_FJ597612_duck_China_S15_2005
I.2_I_b_83_FJ597609_duck_China_JS_51_2005
I.2_I_b_84_FJ597610_duck_China_JS_53_2005
I.2_I_b_140_KT381598_duck_China_Guangdong_GM12_2014
I.2_I_b_141_KT381599_duck_China_Guangdong_GZ331_2014
I.2_I_b_79_FJ597605_duck_China_AH_13_2004
I.2_I_b_78_FJ597604_duck_China_AH_6_2004
I.2_I_b_77_FJ597603_duck_China_AH_5_2004
I.2_I_b_133_KF851269_teal_Ukraine_Krasnooskilsky_5_11_2009
I.2_I_b_97_HE972213_mallard_Luxembourg_4178_2008
I.2_I_b_96_HE972210_duck_Luxembourg_3785_2007
I.2_I_b_146_KU662357_Wild_Garganey_Russia_Novosibirsk_27_2014
I.2_I_b_129_KC503482_slaty_backedgull_Russia_Nikita_539_FFNK11_2007
I.2_I_b_128_KC503481_coot_Russia_538_FFNK10_2007
I.2_I_b_2333_MH675922.1_duck_China_D4_2016
I.2_I_b_2517_MH289832.1_duck_China_JX_2N_2016
I.2_I_b_2518_MH289833.1_duck_China_JX_77C2_2016
I.2_I_b_137_KR869090_chicken_China_GX21_2013
I.2_I_b_145_KU601398_Common_murre_Russia_Tyuleniy_Island_109_2015
I.2_I_b_2520_MH289835.1_Anser_fabalis_China_HN_F2_110_1_2016
I.2_I_b_147_KX352836_teal_Russia_Novosibirsk_region_320_2010
I.2_I_b_98_HG326605_spur_wingedgoose_Nigeria_NIE08_0121_2008
I.2_I_b_99_HG326606_wingedgoose_Nigeria_NIE08_0124_2008
I.2_I_b_134_KF851270_Ruddy_Shelduck_Ukraine_AN_371502_11_2011
I.2_I_b_73_AY965078_duck_Russia_FarEast_2686_2001
I.2_I_b_75_AY972101_Baikal_teal_Russia_FarEast_3652_2002
I.2_I_b_118_JX518874_red_billed_teal_Madagascar_MGA284C_2009
I.2_I_b_85_FJ597611_duck_China_JS_14_2005
I.2_I_b_90_FJ597616_duck_China_ZJ_50_2005
I.2_I_b_92_FJ597618_duck_China_HN_36_2005
I.2_I_b_91_FJ597617_duck_China_HN_34_2005
I.2_I_b_89_FJ597615_duck_China_ZJ_3_2004
I.2_I_b_93_FJ597619_duck_China_HN_37_2005
I.2_I_b_74_AY965079_duck_Russia_FarEast_2713_2001
I.2_I_b_156_KX857706_Blue_winged_teal_TX_USA_AI13_4119_2013
I.2_I_b_149_KX857699_Mallard_MN_USA_AI12_4078_2012
I.2_I_b_148_KX857698_Blue_winged_teal_TX_USA_AI12_3663_2012
I.2_I_b_160_KX857710_Blue_winged_teal_TX_USA_AI14_3711_2014
I.2_I_b_159_KX857709_Mallard_MN_USA_AI14_3452_2014
I.2_I_b_157_KX857707_Mallard_MN_USA_AI13_4403_2013
I.2_I_b_150_KX857700_Blue_winged_teal_TX_USA_AI12_4395_2012
I.2_I_b_151_KX857701_Blue_winged_teal_LA_USA_AI12_4416_2012
I.2_I_b_155_KX857705_Mallard_MN_USA_AI13_3228_2013
I.2_I_b_158_KX857708_Mallard_MN_USA_AI14_3310_2014
I.2_I_b_153_KX857703_Mallard_MN_USA_AI12_4716_2012
I.2_I_b_154_KX857704_Mallard_MN_USA_AI12_4867_2012
I.2_I_b_76_EF564821_mallard_PA_USA_MD_04_204_2004
I.2_I_b_124_KC503455_northern_pintail_AK_USA_AK_44493_723_2009
I.2_I_b_127_KC503471_american_green_winged_teal_AK_USA_AK_44493_893_2009
I.2_I_b_125_KC503467_northern_pintail_AK_USA_AK_44493_801_2009
I.2_I_b_103_JN872169_environment_AK_USA_Pennsylvania_3167_2009
I.2_I_b_122_KC503447_Amer_green_wingedteal_AK_USA_AK_44493_444_2009
I.2_I_b_126_KC503469_northern_pintail_AK_USA_AK_44493_830_2009
I.2_I_b_119_KC503411_northern_pintail_Japan_10EY0020_2010
I.2_I_b_135_KJ920203_mallard_Japan_Yakutiya_852_2011
I.2_I_b_136_KM885166_duck_China_LAH_224_2011
I.2_I_b_116_JX401405_mallard_South_Korea_CBU2374_2007
I.2_I_b_71_AB871655_duck_Japan_Tottori_N12_2006
I.2_I_b_132_KC894391_mallard_China_Jilin_2011
I.2_I_b_101_HM125898_duck_China_WDK_Jiangxi_7793_2004
I.2_I_b_106_JQ966082_mallard_South_Korea_WB_KU146_2008
I.2_I_b_107_JQ966083_wild_bird_South_Korea_WB_KU164_2009
I.2_I_b_123_KC503453_Amer_green_wingedteal_AK_USA_AK_44493_716_2009
I.2_I_b_121_KC503422_spectacled_eider_AK_USA_AK_44332_723_2007
I.2_I_b_95_FJ600539_mule_duck_China_FJ0801_2008
I.2_I_b_102_JN653339_Muscovy_duck_China_M4_2007
I.2_I_b_72_AY965077_Baikal_teal_Russia_FarEast_3658_2002
I.2_I_b_87_FJ597613_duck_China_ZJ_1_2004
I.2_I_b_88_FJ597614_duck_China_ZJ_2_2004
I.2_I_b_2064_KX765175.1_duck_China_LH11740_2013
I.2_I_b_2065_KX765176.1_duck_China_MH331_7_2011
I.2_I_b_182_AY562991_chicken_Ireland_Ulster_1967
I.1.1_I_a_45_AY935497_chicken_Australia_1997PR_32_1999
I.1.1_I_a_46_AY935498_chicken_Australia_99_1435_1999
I.1.1_I_a_38_AY935490_chicken_Australia_02_1334_2002
I.1.1_I_a_42_AY935494_chicken_Australia_99_0655_1999
I.1.1_I_a_43_AY935495_chicken_Australia_99_0868_hi_1999
I.1.1_I_a_44_AY935496_chicken_Australia_99_0868_lo_1999
I.1.1_I_a_39_AY935491_chicken_Australia_98_1154_1998
I.1.1_I_a_47_GU332645_chicken_Australia_9809_19_1107_1998
I.1.1_I_a_41_AY935493_chicken_Australia_98_1252_1998
I.1.1_I_a_40_AY935492_chicken_Australia_98_1249_1998
I.1.1_I_a_36_AF217084_chicken_Australia_Queensland_V_4_1966
I.1.1_I_a_55_M24693_chicken_Australia_Queensland_1966
I.1.1_I_a_2135_MG869263.1_chicken_Vietnam_NVNvacI2_2016
I.1.1_I_a_54_M24692_chicken_Australia_D26_76_1999
I.1.2.2_I_d_176_KC503476_northern_pintail_AK_USA_44500_136__2009
I.1.2.2_I_d_178_KX857711_American_green_winged_teal_MN_USA_AI09_3831_2009
I.1.2.2_I_d_175_EF564817_ruddyturnstone_DE_USA_492_2002
I.1.2.2_I_d_177_KC503479_redpoll_Russia_Nikita_530_FFNK2_2008
I.1.2.2_I_d_174_AB465607_chicken_Japan_Ishi_1962
I.1.2.1_I_c_167_KC503478_slaty_backedgull_Japan_9KS_0098_2009
I.1.2.1_I_c_172_KX352834_gull_Russia_Tyva_14_2014
I.1.2.1_I_c_165_EF564816_redknot_NJ_USA_A_101_1383_2001
I.1.2.1_I_c_166_GQ918280_black_headed_gull_Sweden__1994
III_III_428_EF201805_avian_Mukteswar_1940
IV_IV_444_AY741404_Fowl_UK_Herts_1933

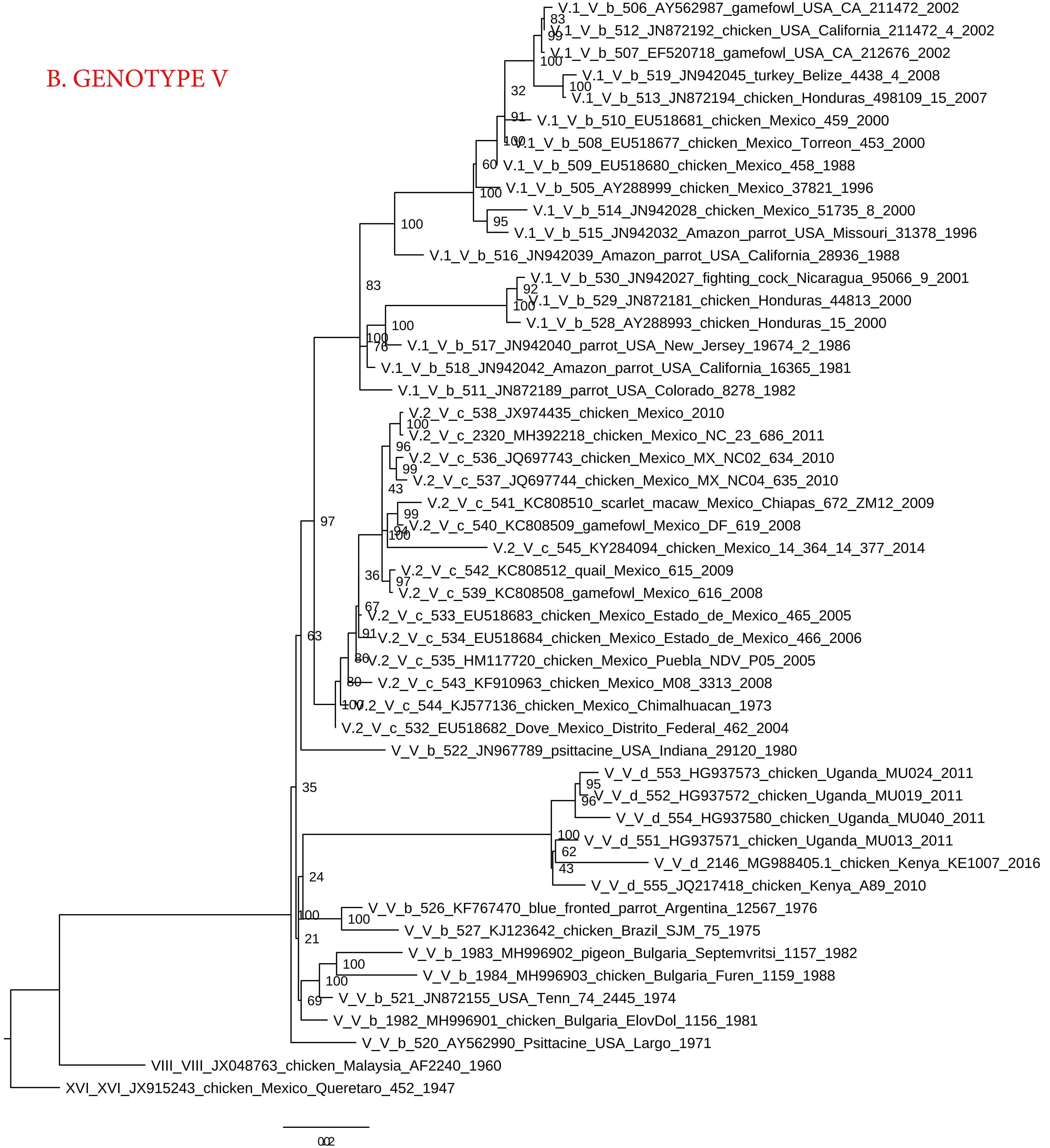
B. GENOTYPE V
V.1_V_b_506_AY562987_gamefowl_USA_CA_211472_2002
V.1_V_b_512_JN872192_chicken_USA_California_211472_4_2002
V.1_V_b_507_EF520718_gamefowl_USA_CA_212676_2002
V.1_V_b_519_JN942045_turkey_Belize_4438_4_2008
V.1_V_b_513_JN872194_chicken_Honduras_498109_15_2007
V.1_V_b_510_EU518681_chicken_Mexico_459_2000
V.1_V_b_508_EU518677_chicken_Mexico_Torreon_453_2000
V.1_V_b_509_EU518680_chicken_Mexico_458_1988
V.1_V_b_505_AY288999_chicken_Mexico_37821_1996
V.1_V_b_514_JN942028_chicken_Mexico_51735_8_2000
V.1_V_b_515_JN942032_Amazon_parrot_USA_Missouri_31378_1996
V.1_V_b_516_JN942039_Amazon_parrot_USA_California_28936_1988
V.1_V_b_530_JN942027_fighting_cock_Nicaragua_95066_9_2001
V.1_V_b_529_JN872181_chicken_Honduras_44813_2000
V.1_V_b_528_AY288993_chicken_Honduras_15_2000
V.1_V_b_517_JN942040_parrot_USA_New_Jersey_19674_2_1986
V.1_V_b_518_JN942042_Amazon_parrot_USA_California_16365_1981
V.1_V_b_511_JN872189_parrot_USA_Colorado_8278_1982
V.2_V_c_538_JX974435_chicken_Mexico_2010
V.2_V_c_2320_MH392218_chicken_Mexico_NC_23_686_2011
V.2_V_c_536_JQ697743_chicken_Mexico_MX_NC02_634_2010
V.2_V_c_537_JQ697744_chicken_Mexico_MX_NC04_635_2010
V.2_V_c_541_KC808510_scarlet_macaw_Mexico_Chiapas_672_ZM12_2009
V.2_V_c_540_KC808509_gamefowl_Mexico_DF_619_2008
V.2_V_c_545_KY284094_chicken_Mexico_14_364_14_377_2014
V.2_V_c_542_KC808512_quail_Mexico_615_2009
V.2_V_c_539_KC808508_gamefowl_Mexico_616_2008
V.2_V_c_533_EU518683_chicken_Mexico_Estado_de_Mexico_465_2005
V.2_V_c_534_EU518684_chicken_Mexico_Estado_de_Mexico_466_2006
V.2_V_c_535_HM117720_chicken_Mexico_Puebla_NDV_P05_2005
V.2_V_c_543_KF910963_chicken_Mexico_M08_3313_2008
V.2_V_c_544_KJ577136_chicken_Mexico_Chimalhuacan_1973
V.2_V_c_532_EU518682_Dove_Mexico_Distrito_Federal_462_2004
V_V_b_522_JN967789_psittacine_USA_Indiana_29120_1980
V_V_d_553_HG937573_chicken_Uganda_MU024_2011
V_V_d_552_HG937572_chicken_Uganda_MU019_2011
V_V_d_554_HG937580_chicken_Uganda_MU040_2011
V_V_d_551_HG937571_chicken_Uganda_MU013_2011
V_V_d_2146_MG988405.1_chicken_Kenya_KE1007_2016
V_V_d_555_JQ217418_chicken_Kenya_A89_2010
V_V_b_526_KF767470_blue_fronted_parrot_Argentina_12567_1976
V_V_b_527_KJ123642_chicken_Brazil_SJM_75_1975
V_V_b_1983_MH996902_pigeon_Bulgaria_Septemvritsi_1157_1982
V_V_b_1984_MH996903_chicken_Bulgaria_Furen_1159_1988
V_V_b_521_JN872155_USA_Tenn_74_2445_1974
V_V_b_1982_MH996901_chicken_Bulgaria_ElovDol_1156_1981
V_V_b_520_AY562990_Psittacine_USA_Largo_1971
VIII_VIII_JX048763_chicken_Malaysia_AF2240_1960
XVI_XVI_JX915243_chicken_Mexico_Queretaro_452_1947
0.02

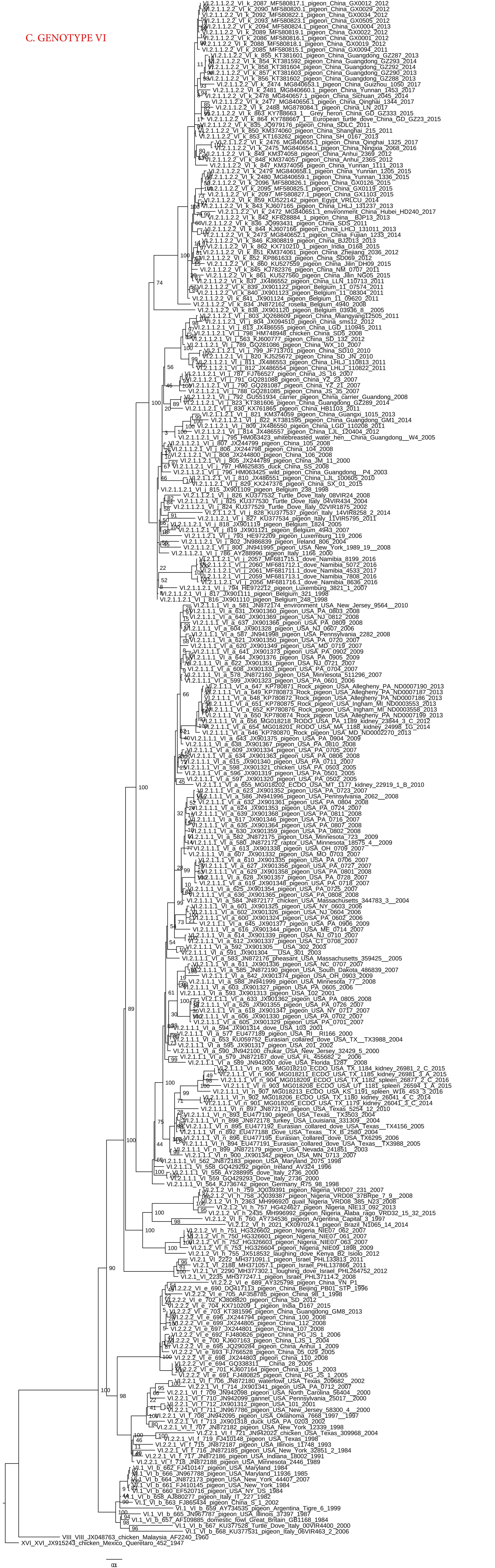
C. GENOTYPE VI

D. GENOTYPE VII

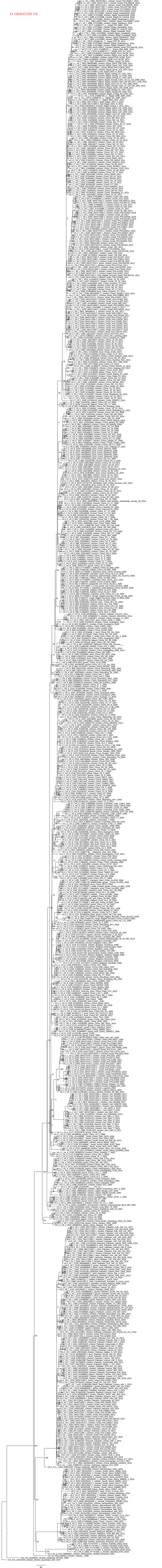

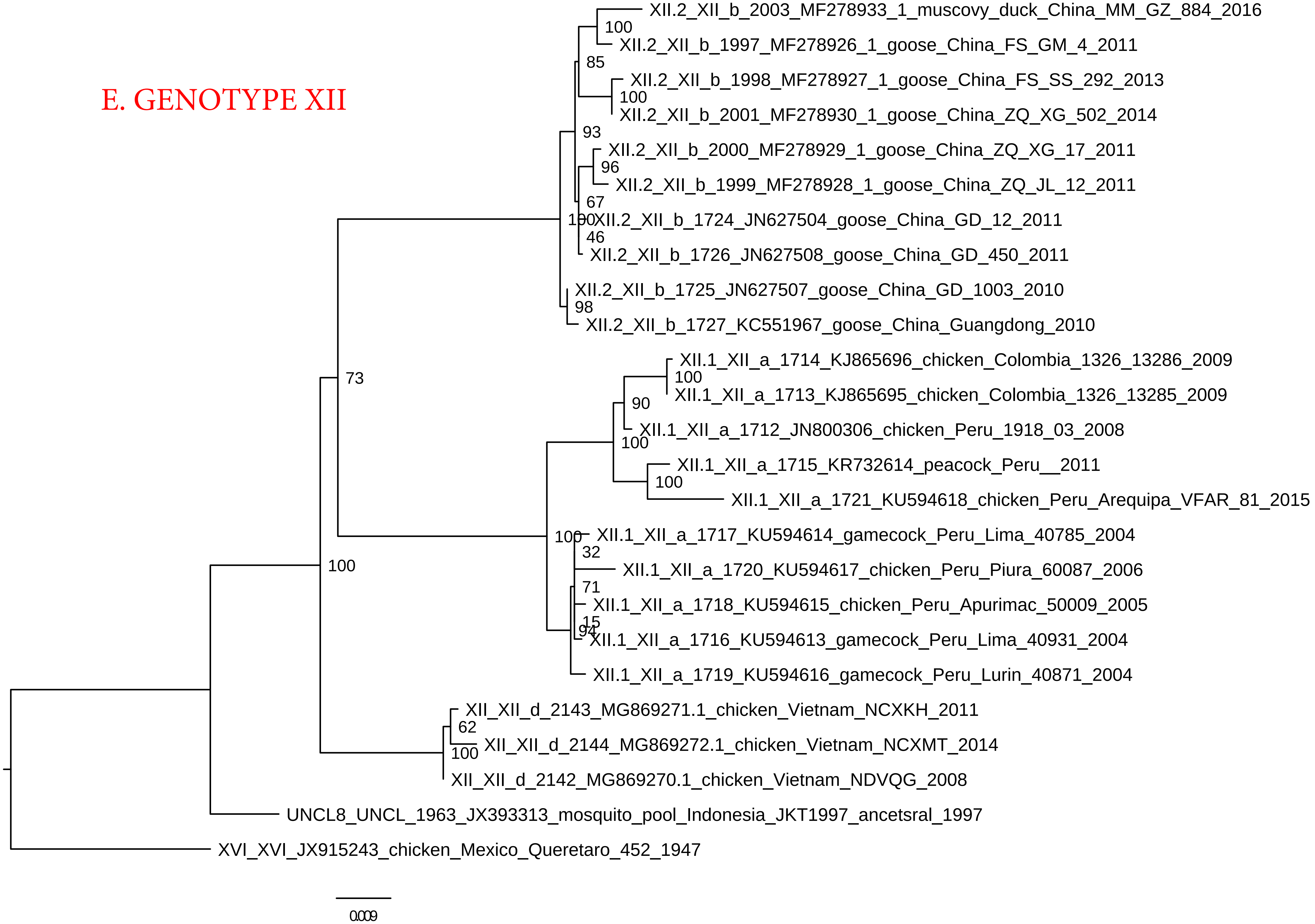
E. GENOTYPE XII
XII.2_XII_b_2003_MF278933_1_muscovy_duck_China_MM_GZ_884_2016
100
XII.2_XII_b_1997_MF278926_1_goose_China_FS_GM_4_2011
85
XII.2_XII_b_1998_MF278927_1_goose_China_FS_SS_292_2013
100
XII.2_XII_b_2001_MF278930_1_goose_China_ZQ_XG_502_2014
93
XII.2_XII_b_2000_MF278929_1_goose_China_ZQ_XG_17_2011
96
XII.2_XII_b_1999_MF278928_1_goose_China_ZQ_JL_12_2011
67
XII.2_XII_b_1724_JN627504_goose_China_GD_12_2011
46
XII.2_XII_b_1726_JN627508_goose_China_GD_450_2011
XII.2_XII_b_1725_JN627507_goose_China_GD_1003_2010
98
XII.2_XII_b_1727_KC551967_goose_China_Guangdong_2010
73
XII.1_XII_a_1714_KJ865696_chicken_Colombia_1326_13286_2009
100
XII.1_XII_a_1713_KJ865695_chicken_Colombia_1326_13285_2009
90
XII.1_XII_a_1712_JN800306_chicken_Peru_1918_03_2008
100
XII.1_XII_a_1715_KR732614_peacock_Peru__2011
100
XII.1_XII_a_1721_KU594618_chicken_Peru_Arequipa_VFAR_81_2015
XII.1_XII_a_1717_KU594614_gamecock_Peru_Lima_40785_2004
32
XII.1_XII_a_1720_KU594617_chicken_Peru_Piura_60087_2006
71
XII.1_XII_a_1718_KU594615_chicken_Peru_Apurimac_50009_2005
15
94
XII.1_XII_a_1716_KU594613_gamecock_Peru_Lima_40931_2004
XII.1_XII_a_1719_KU594616_gamecock_Peru_Lurin_40871_2004
100
XII_XII_d_2143_MG869271.1_chicken_Vietnam_NCXKH_2011
62
XII_XII_d_2144_MG869272.1_chicken_Vietnam_NCXMT_2014
100
XII_XII_d_2142_MG869270.1_chicken_Vietnam_NDVQG_2008
UNCL8_UNCL_1963_JX393313_mosquito_pool_Indonesia_JKT1997_ancetsral_1997
XVI_XVI_JX915243_chicken_Mexico_Queretaro_452_1947
0.009

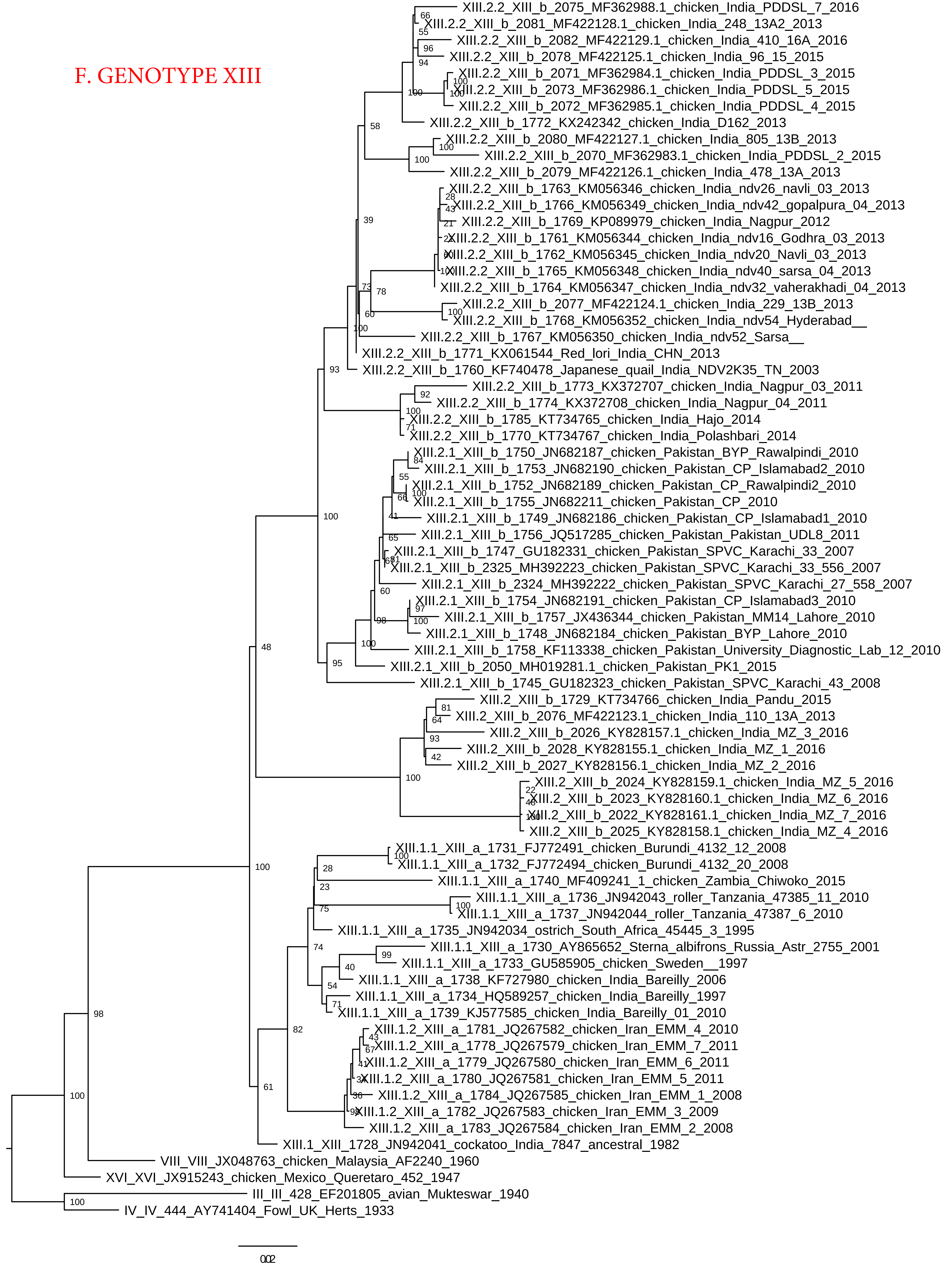
F. GENOTYPE XIII
XIII.2.2_XIII_b_2075_MF362988.1_chicken_India_PDDSL_7_2016
XIII.2.2_XIII_b_2081_MF422128.1_chicken_India_248_13A2_2013
XIII.2.2_XIII_b_2082_MF422129.1_chicken_India_410_16A_2016
XIII.2.2_XIII_b_2078_MF422125.1_chicken_India_96_15_2015
XIII.2.2_XIII_b_2071_MF362984.1_chicken_India_PDDSL_3_2015
XIII.2.2_XIII_b_2073_MF362986.1_chicken_India_PDDSL_5_2015
XIII.2.2_XIII_b_2072_MF362985.1_chicken_India_PDDSL_4_2015
XIII.2.2_XIII_b_1772_KX242342_chicken_India_D162_2013
XIII.2.2_XIII_b_2080_MF422127.1_chicken_India_805_13B_2013
XIII.2.2_XIII_b_2070_MF362983.1_chicken_India_PDDSL_2_2015
XIII.2.2_XIII_b_2079_MF422126.1_chicken_India_478_13A_2013
XIII.2.2_XIII_b_1763_KM056346_chicken_India_ndv26_navli_03_2013
XIII.2.2_XIII_b_1766_KM056349_chicken_India_ndv42_gopalpura_04_2013
XIII.2.2_XIII_b_1769_KP089979_chicken_India_Nagpur_2012
XIII.2.2_XIII_b_1761_KM056344_chicken_India_ndv16_Godhra_03_2013
XIII.2.2_XIII_b_1762_KM056345_chicken_India_ndv20_Navli_03_2013
XIII.2.2_XIII_b_1765_KM056348_chicken_India_ndv40_sarsa_04_2013
XIII.2.2_XIII_b_1764_KM056347_chicken_India_ndv32_vaherakhadi_04_2013
XIII.2.2_XIII_b_2077_MF422124.1_chicken_India_229_13B_2013
XIII.2.2_XIII_b_1768_KM056352_chicken_India_ndv54_Hyderabad__
XIII.2.2_XIII_b_1767_KM056350_chicken_India_ndv52_Sarsa__
XIII.2.2_XIII_b_1771_KX061544_Red_lori_India_CHN_2013
XIII.2.2_XIII_b_1760_KF740478_Japanese_quail_India_NDV2K35_TN_2003
XIII.2.2_XIII_b_1773_KX372707_chicken_India_Nagpur_03_2011
XIII.2.2_XIII_b_1774_KX372708_chicken_India_Nagpur_04_2011
XIII.2.2_XIII_b_1785_KT734765_chicken_India_Hajo_2014
XIII.2.2_XIII_b_1770_KT734767_chicken_India_Polashbari_2014
XIII.2.1_XIII_b_1750_JN682187_chicken_Pakistan_BYP_Rawalpindi_2010
XIII.2.1_XIII_b_1753_JN682190_chicken_Pakistan_CP_Islamabad2_2010
XIII.2.1_XIII_b_1752_JN682189_chicken_Pakistan_CP_Rawalpindi2_2010
XIII.2.1_XIII_b_1755_JN682211_chicken_Pakistan_CP_2010
XIII.2.1_XIII_b_1749_JN682186_chicken_Pakistan_CP_Islamabad1_2010
XIII.2.1_XIII_b_1756_JQ517285_chicken_Pakistan_Pakistan_UDL8_2011
XIII.2.1_XIII_b_1747_GU182331_chicken_Pakistan_SPVC_Karachi_33_2007
XIII.2.1_XIII_b_2325_MH392223_chicken_Pakistan_SPVC_Karachi_33_556_2007
XIII.2.1_XIII_b_2324_MH392222_chicken_Pakistan_SPVC_Karachi_27_558_2007
XIII.2.1_XIII_b_1754_JN682191_chicken_Pakistan_CP_Islamabad3_2010
XIII.2.1_XIII_b_1757_JX436344_chicken_Pakistan_MM14_Lahore_2010
XIII.2.1_XIII_b_1748_JN682184_chicken_Pakistan_BYP_Lahore_2010
XIII.2.1_XIII_b_1758_KF113338_chicken_Pakistan_University_Diagnostic_Lab_12_2010
XIII.2.1_XIII_b_2050_MH019281.1_chicken_Pakistan_PK1_2015
XIII.2.1_XIII_b_1745_GU182323_chicken_Pakistan_SPVC_Karachi_43_2008
XIII.2_XIII_b_1729_KT734766_chicken_India_Pandu_2015
XIII.2_XIII_b_2076_MF422123.1_chicken_India_110_13A_2013
XIII.2_XIII_b_2026_KY828157.1_chicken_India_MZ_3_2016
XIII.2_XIII_b_2028_KY828155.1_chicken_India_MZ_1_2016
XIII.2_XIII_b_2027_KY828156.1_chicken_India_MZ_2_2016
XIII.2_XIII_b_2024_KY828159.1_chicken_India_MZ_5_2016
XIII.2_XIII_b_2023_KY828160.1_chicken_India_MZ_6_2016
XIII.2_XIII_b_2022_KY828161.1_chicken_India_MZ_7_2016
XIII.2_XIII_b_2025_KY828158.1_chicken_India_MZ_4_2016
XIII.1.1_XIII_a_1731_FJ772491_chicken_Burundi_4132_12_2008
XIII.1.1_XIII_a_1732_FJ772494_chicken_Burundi_4132_20_2008
XIII.1.1_XIII_a_1740_MF409241_1_chicken_Zambia_Chiwoko_2015
XIII.1.1_XIII_a_1736_JN942043_roller_Tanzania_47385_11_2010
XIII.1.1_XIII_a_1737_JN942044_roller_Tanzania_47387_6_2010
XIII.1.1_XIII_a_1735_JN942034_ostrich_South_Africa_45445_3_1995
XIII.1.1_XIII_a_1730_AY865652_Sterna_albifrons_Russia_Astr_2755_2001
XIII.1.1_XIII_a_1733_GU585905_chicken_Sweden__1997
XIII.1.1_XIII_a_1738_KF727980_chicken_India_Bareilly_2006
XIII.1.1_XIII_a_1734_HQ589257_chicken_India_Bareilly_1997
XIII.1.1_XIII_a_1739_KJ577585_chicken_India_Bareilly_01_2010
XIII.1.2_XIII_a_1781_JQ267582_chicken_Iran_EMM_4_2010
XIII.1.2_XIII_a_1778_JQ267579_chicken_Iran_EMM_7_2011
XIII.1.2_XIII_a_1779_JQ267580_chicken_Iran_EMM_6_2011
XIII.1.2_XIII_a_1780_JQ267581_chicken_Iran_EMM_5_2011
XIII.1.2_XIII_a_1784_JQ267585_chicken_Iran_EMM_1_2008
XIII.1.2_XIII_a_1782_JQ267583_chicken_Iran_EMM_3_2009
XIII.1.2_XIII_a_1783_JQ267584_chicken_Iran_EMM_2_2008
XIII.1_XIII_1728_JN942041_cockatoo_India_7847_ancestral_1982
VIII_VIII_JX048763_chicken_Malaysia_AF2240_1960
XVI_XVI_JX915243_chicken_Mexico_Queretaro_452_1947
III_III_428_EF201805_avian_Mukteswar_1940
IV_IV_444_AY741404_Fowl_UK_Herts_1933

G. GENOTYPE XIV

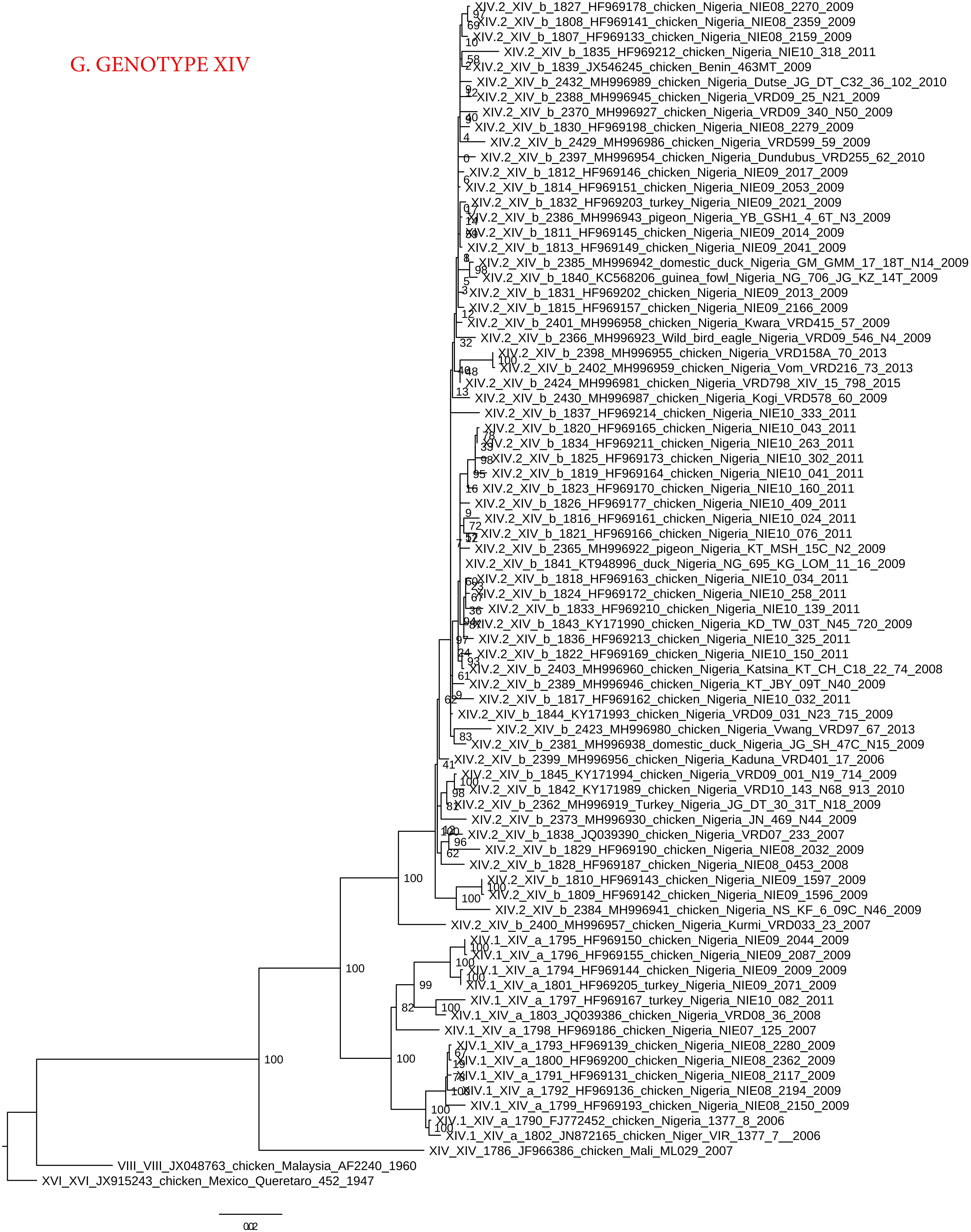

H. GENOTYPE XVIII

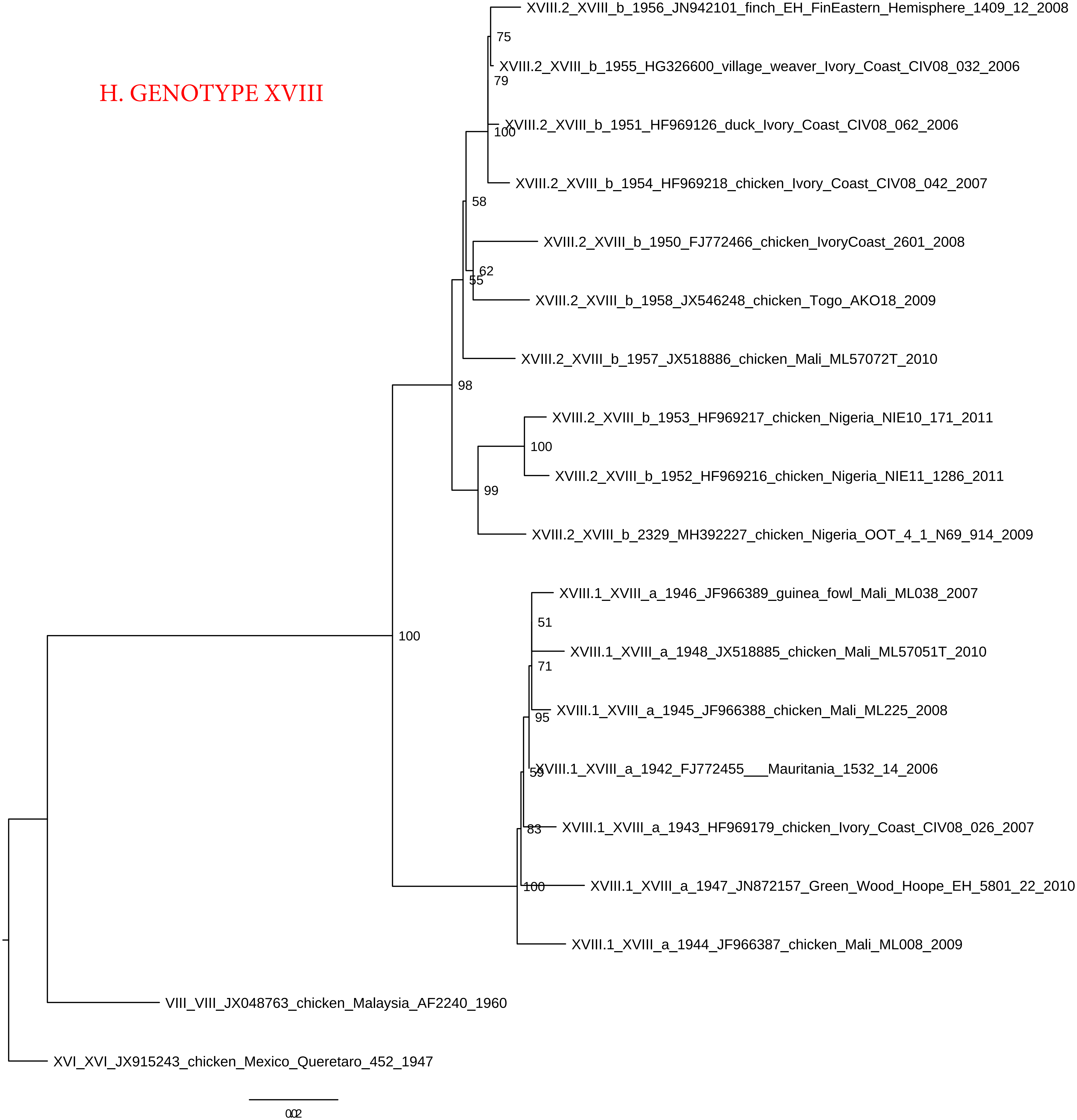

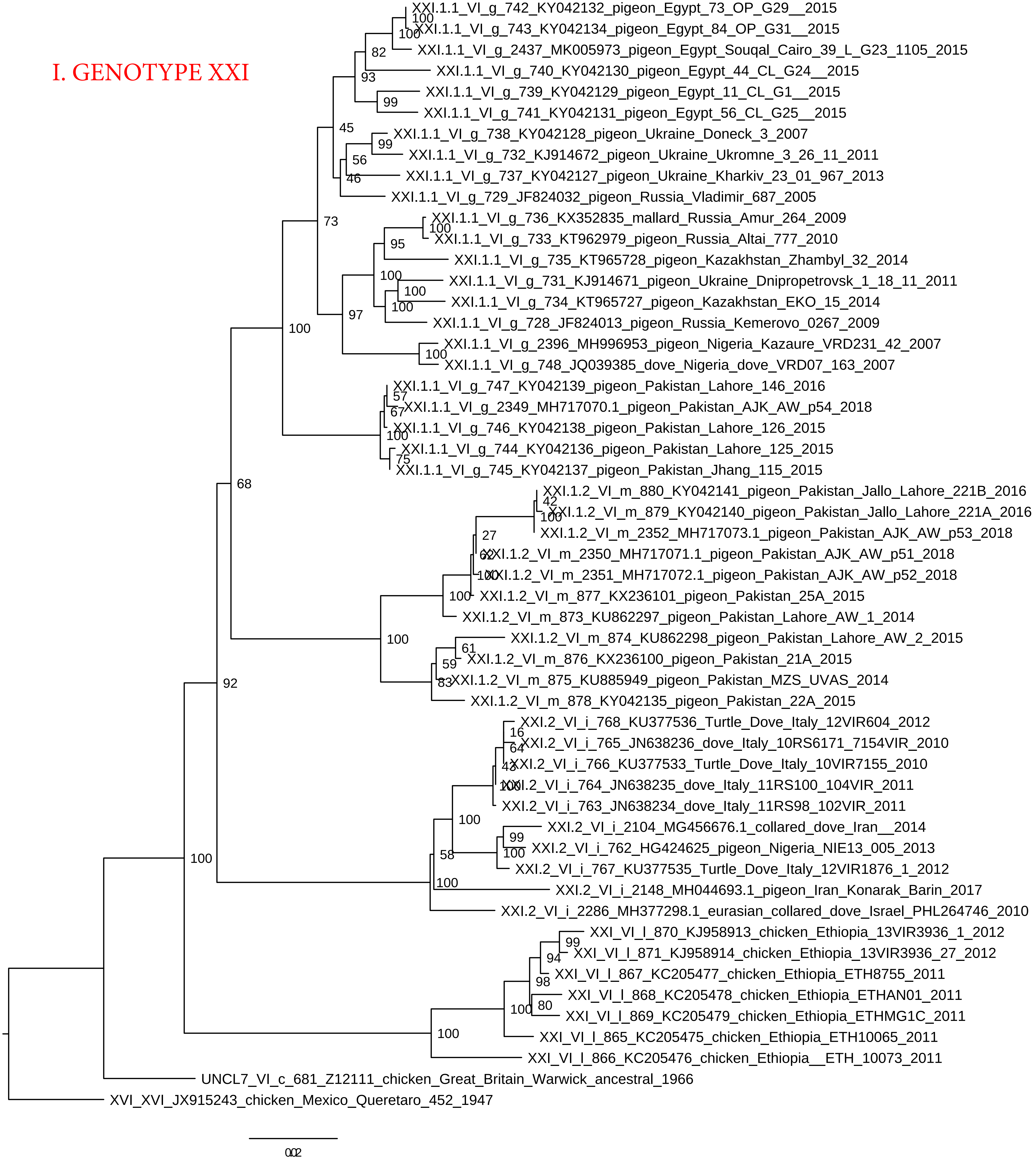
I. GENOTYPE XXI
XXI.1.1_VI_g_742_KY042132_pigeon_Egypt_73_OP_G29__2015
XXI.1.1_VI_g_743_KY042134_pigeon_Egypt_84_OP_G31__2015
XXI.1.1_VI_g_2437_MK005973_pigeon_Egypt_Souqal_Cairo_39_L_G23_1105_2015
XXI.1.1_VI_g_740_KY042130_pigeon_Egypt_44_CL_G24__2015
XXI.1.1_VI_g_739_KY042129_pigeon_Egypt_11_CL_G1__2015
XXI.1.1_VI_g_741_KY042131_pigeon_Egypt_56_CL_G25__2015
XXI.1.1_VI_g_738_KY042128_pigeon_Ukraine_Doneck_3_2007
XXI.1.1_VI_g_732_KJ914672_pigeon_Ukraine_Ukromne_3_26_11_2011
XXI.1.1_VI_g_737_KY042127_pigeon_Ukraine_Kharkiv_23_01_967_2013
XXI.1.1_VI_g_729_JF824032_pigeon_Russia_Vladimir_687_2005
XXI.1.1_VI_g_736_KX352835_mallard_Russia_Amur_264_2009
XXI.1.1_VI_g_733_KT962979_pigeon_Russia_Altai_777_2010
XXI.1.1_VI_g_735_KT965728_pigeon_Kazakhstan_Zhambyl_32_2014
XXI.1.1_VI_g_731_KJ914671_pigeon_Ukraine_Dnipropetrovsk_1_18_11_2011
XXI.1.1_VI_g_734_KT965727_pigeon_Kazakhstan_EKO_15_2014
XXI.1.1_VI_g_728_JF824013_pigeon_Russia_Kemerovo_0267_2009
XXI.1.1_VI_g_2396_MH996953_pigeon_Nigeria_Kazaure_VRD231_42_2007
XXI.1.1_VI_g_748_JQ039385_dove_Nigeria_dove_VRD07_163_2007
XXI.1.1_VI_g_747_KY042139_pigeon_Pakistan_Lahore_146_2016
XXI.1.1_VI_g_2349_MH717070.1_pigeon_Pakistan_AJK_AW_p54_2018
XXI.1.1_VI_g_746_KY042138_pigeon_Pakistan_Lahore_126_2015
XXI.1.1_VI_g_744_KY042136_pigeon_Pakistan_Lahore_125_2015
XXI.1.1_VI_g_745_KY042137_pigeon_Pakistan_Jhang_115_2015
XXI.1.2_VI_m_880_KY042141_pigeon_Pakistan_Jallo_Lahore_221B_2016
XXI.1.2_VI_m_879_KY042140_pigeon_Pakistan_Jallo_Lahore_221A_2016
XXI.1.2_VI_m_2352_MH717073.1_pigeon_Pakistan_AJK_AW_p53_2018
XXI.1.2_VI_m_2350_MH717071.1_pigeon_Pakistan_AJK_AW_p51_2018
XXI.1.2_VI_m_2351_MH717072.1_pigeon_Pakistan_AJK_AW_p52_2018
XXI.1.2_VI_m_877_KX236101_pigeon_Pakistan_25A_2015
XXI.1.2_VI_m_873_KU862297_pigeon_Pakistan_Lahore_AW_1_2014
XXI.1.2_VI_m_874_KU862298_pigeon_Pakistan_Lahore_AW_2_2015
XXI.1.2_VI_m_876_KX236100_pigeon_Pakistan_21A_2015
XXI.1.2_VI_m_875_KU885949_pigeon_Pakistan_MZS_UVAS_2014
XXI.1.2_VI_m_878_KY042135_pigeon_Pakistan_22A_2015
XXI.2_VI_i_768_KU377536_Turtle_Dove_Italy_12VIR604_2012
XXI.2_VI_i_765_JN638236_dove_Italy_10RS6171_7154VIR_2010
XXI.2_VI_i_766_KU377533_Turtle_Dove_Italy_10VIR7155_2010
XXI.2_VI_i_764_JN638235_dove_Italy_11RS100_104VIR_2011
XXI.2_VI_i_763_JN638234_dove_Italy_11RS98_102VIR_2011
XXI.2_VI_i_2104_MG456676.1_collared_dove_Iran__2014
XXI.2_VI_i_762_HG424625_pigeon_Nigeria_NIE13_005_2013
XXI.2_VI_i_767_KU377535_Turtle_Dove_Italy_12VIR1876_1_2012
XXI.2_VI_i_2148_MH044693.1_pigeon_Iran_Konarak_Barin_2017
XXI.2_VI_i_2286_MH377298.1_eurasian_collared_dove_Israel_PHL264746_2010
XXI_VI_I_870_KJ958913_chicken_Ethiopia_13VIR3936_1_2012
XXI_VI_I_871_KJ958914_chicken_Ethiopia_13VIR3936_27_2012
XXI_VI_I_867_KC205477_chicken_Ethiopia_ETH8755_2011
XXI_VI_I_868_KC205478_chicken_Ethiopia_ETHAN01_2011
XXI_VI_I_869_KC205479_chicken_Ethiopia_ETHMG1C_2011
XXI_VI_I_865_KC205475_chicken_Ethiopia_ETH10065_2011
XXI_VI_I_866_KC205476_chicken_Ethiopia__ETH_10073_2011
UNCL7_VI_c_681_Z12111_chicken_Great_Britain_Warwick_ancestral_1966
XVI_XVI_JX915243_chicken_Mexico_Queretaro_452_1947
0.02
