# Supplementary material for: Updated unified phylogenetic classification system and revised nomenclature for Newcastle disease virus
Source: Infect Genet Evol. 2019 Oct;74:103917. doi: 10.1016/j.meegid.2019.103917 (PMC6876278; doi:10.1016/j.meegid.2019.103917)
Supplement: Supplemental Fig. S8 — Step-by-step guidelines of settings for building Maximum likelihood trees using the CIPRES Science Gateway (provided as screenshots from http://www.phylo.org). [file mmc13.pdf]

Supplemental Fig. S8. Step-by-step guidelines of settings for building Maximum likelihood trees using the CIPRES Science Gateway (provided as screenshots from http://www.phylo.org).

Folders

Total Storage: 62 MB

CLASS II
- **Data (3)**
- **Tasks (5)**

Create new task

Task Summary | Select Data | Select Tool | Set Parameters

RAxML-HPC v.8 on XSEDE: NEW Interface! Phylogenetic tree inference using maximum likelihood/rapid bootstrapping run on XSEDE (Alexandros Stamatakis)

Simple Parameters

Maximum Hours to Run (click here for help setting this correctly) * 5
Set a name for output files (-n) tree
Enable ML searches under CAT (-F) ☐
Outgroup (-o) ()one or more comma-separated outgroups, see comment for syntax)
Specify the number of distinct rate categories (-c) *
Disable Rate Heterogeneity (-V) * ☐
Supply a tree (Not available when doing rapid bootstrapping, -x) (-t)
Specify a random seed value for parsimony inferences (-p) * ☑
Enter a random seed value for parsimony inferences (-p "value" gives reproducible results from random starting tree) * 456
Specify an initial rearrangement setting (-i) * ☐
Specify the distance from original pruning point (-i) *
Constraint (-g)
Binary Backbone (-r)
Use a mixed/partitioned model? (-q)
Estimate individual per-partition branch lengths (-M) * ☐
Estimate proportion of invariable sites (GTRGAMMA + I) ○ **no** ◉ **yes**
Choose an input file that excludes the range of positions specifed in this file (-E)
Weight characters as specifed in this file (-a)
Disable checking for sequences with no values (-O) ☐
Print output files that can be parsed by Mesquite. (-mesquite) ☐

Advanced Parameters

Please select the Data Type * Nucleotide

Nucleic Acid Options

Choose model for bootstrapping phase ○ **[Not Mandatory]** ○ **GTRCAT** ◉ **GTRGAMMA**

Evaluate DNA partitions only under this model ◉ **[Not Mandatory]** ○ **HKY85** ○ **K80** ○ **JC69**

Protein Analysis Options

Choose GAMMA or CAT model: + ○ **Protein GAMMA** ○ **Protein CAT**

Protein Substitution Matrix + DAYHOFF

Upload a Custom Protein Substitution Matrix (-P)

Use a Partition file that specifies AA Matrices ☐

Select the First Protein Substitution Matrix Called in Your Partition File

Select the Second Protein Substitution Matrix Called in Your Partition File

Select the Third Protein Substitution Matrix Called in Your Partition File

Select the Fourth Protein Substitution Matrix Called in Your Partition File

Select the Fifth Protein Substitution Matrix Called in Your Partition File

Use empirical frequencies? [F] ◉ **No** ○ **Yes**

Make an ML estimate of frequencies [X] ◉ **No** ○ **Yes**

RNA Secondary Structure Options

Upload a Secondary Structure File (-S)

Use an RNA Secondary Structure Substitution Model (-A) + Six State Model A

Binary Matrix Options

Binary data model (-m) + ○ **Binary CAT** ○ **Binary GAMMA**

Multiple State Morphological Matrix Options

Multiple State Data Model (-m) + ○ **Multi-state CAT** ○ **Multi-state GAMMA**

Select a Multiple state data model (-K) + ○ **Ordered** ○ **MK** ○ **GTR**

Configure the Analysis

Select the Analysis Type * [Rapid bootstrap analysis / search for best-scoring ML tree (-f a)]

Specify the number alternative runs on distinct starting trees? (-N) + ☐

Enter number of number alternative runs (-N) + [ ]

File with topologies for bipartitions (-z) [ ]

Don't use BFGS searching algorithm (--no-bfgs) ☐

Write intermediate tree files to a file (-j) ☐

Use ML search convergence criterion. (-D) ☐

Specify majority rule consensus tree (-J) technique + [Majority rule]

Ascertainment Bias Confguration

Correct for Ascertainment bias (ASC_) ◉ **no** ○ **yes**

Ascertainment bias correction type (--asc-corr) ◉ **[Not Mandatory]** ○ **Lewis** ○ **Felsenstein** ○ **Stamatakis**

Choose Ascertainment bias correction file 1 (will be named p1.txt) + [ ]

Choose Ascertainment bias correction file 2 (will be named p2.txt) + [ ]

Choose Ascertainment bias correction file 3 (will be named p3.txt) + [ ]

Choose Ascertainment bias correction file 4 (will be named p4.txt) + [ ]

Choose Ascertainment bias correction file 5 (will be named p5.txt) + [ ]

Choose Ascertainment bias correction file 6 (will be named p6.txt) + [ ]

Configure more memory

I have a data set that may require more than 20 GB of memory + ☐

Enter the number of patterns in your dataset

Enter the number of taxa in your dataset

Configure Bootstrapping

Choose a Bootstrapping Type ○**No Bootstrapping** ○**Non-parametric Boostrapping (-b)** ◉**Rapid Bootstrapping (-x)**

Enter a random seed value for bootstrapping + 12345

Print branch lengths (-k) ☐

Specify bootstrap protocol + ◉**Specify an explicit number of bootstraps** ○**Let RaxML halt bootstrapping automatically**

Bootstrap iterations (-N) + 1000

Select Bootstopping Criterion: (autoMRE is recommended)

Select the criterion for a posteriori bootstopping analysis (-l) + autoFC

File with topologies for a posteriori bootstopping (-z)

Save Parameters | Reset | Cancel

Advanced Help
